# Total Synthesis and Structure Confirmation of (*E*) and (*Z*)-Ocellenyne

**DOI:** 10.1021/acs.orglett.2c03524

**Published:** 2022-12-12

**Authors:** Harry B. Hicks, Daniel S. Brown, Hau Sun Sam Chan, Bruno A. Sousa, Kirsten E. Christensen, Jonathan W. Burton

**Affiliations:** † Chemistry Research Laboratory, University of Oxford, Mansfield Road, Oxford, OX1 3TA, U.K.; ‡ Vertex Pharmaceuticals, 86-88 Jubilee Avenue, Milton Park, Abingdon, OX14 4RW, U.K.

## Abstract

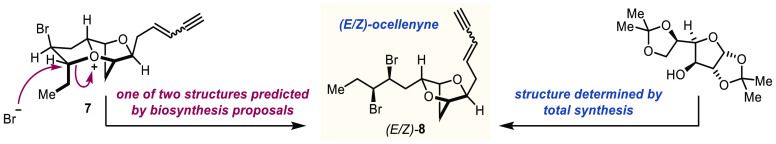

The (*E*/*Z*)-ocellenynes are C_15_ dibrominated *Laurencia* natural products whose structures have been subject to several reassignments on the basis of extensive NMR analysis, biosynthetic postulates, and DFT calculations. Herein, we report the synthesis of both (*E*)- and (*Z*)-ocellenyne, which, in combination with single crystal X-ray diffraction studies, allows their absolute configuration to be established and defines the configuration of the *syn*-12,13-dibromide as being (*S*, *S*) in keeping with their proposed biogenesis from the (6*S*, 7*S*)-laurediols.

The (*E/Z*)-ocellenynes were isolated by Scheuer and co-workers from the digestive glands of the sea hare *Aplysia oculifera* collected off the Hawaiian island of Oahu.^1^ On the basis of spectroscopic and chemical degradation studies, the structures were proposed to be dibrominated C_15_*Laurencia* natural products possessing a novel (6*S*,7*R*) or (6*R*,7*S*)-6,10-*syn*-dioxabicyclo[2.2.1]heptane core and a 12,13-dibromide (Figure 1B, **1**); the relative configuration of the *syn*-12,13-dibromide was ascertained via a zinc-mediated reduction to give a (*Z*)-alkene.^2^ The ocellenynes belong to an ever-growing family of C_15_ acetogenin-derived halogenated natural products isolated from *Laurencia* spp.^3^ The biogenesis of a number of these natural products has been proposed to arise from the laurediols **2** via a sequence of bromonium ion induced etherification reactions, followed by formation of complex tricyclic oxonium ions, which are opened at any of three positions, to give a range of natural products.^4−6^ One of the first proposals of oxonium ions as intermediates in the biosynthesis of *Laurencia* (*L*.) natural products came from T. Suzuki and M. Suzuki et al.,^5^ who proposed that the oxonium ions **4** are formed from bromoetherification reactions of (*R*,*R*)-laurediol [(*R*,*R*)-**2**] to give the prelaurefucins **3**, which on transannular displacement of bromide would give **4** (Figure 1A). Opening of the oxonium ions **4** with bromide at C-13 would give the ocellenynes **5** having a (6*R*,7*R*,12*S*,13*S*)-6,10-*anti* configuration—the Suzuki ocellenynes.^7,8^ In relation to the synthesis and structure determination of elatenyne (not shown), diastereomeric oxonium ions **7** derived from (*S*,*S*)-laurediols [(*S*,*S*)-**2**] via the bromofucins **6** were proposed as key biosynthetic intermediates in the biogenesis of a number of other C_15_*L*. natural products.^6^ Opening of the oxonium ions **7** with bromide at C-13, first proposed by Kim et al.,^9,10^ would give rise to the ocellenynes **8** with a (6*S*,7*S*,12*S*,13*S*)-6,10-*anti* configuration (Figure 1A).^11^ The relative configuration of (*E*)-**8** is the most likely configuration of (*E*)-ocellenyne, as predicted from DFT NMR chemical shift calculations by Kim and Paton et al. (85% DP4 metric),^9^ compared with the Scheuer structure (*E*)-**1** and the Suzuki structure (*E*)-**5**, with the Suzuki structure (*E*)-**5** having a 15% DP4 metric.^12^ Furthermore, ^1^H NMR coupling constant analysis by Kim et al. of model dioxabicyclo[2.2.1]heptane cores with the Scheuer and Suzuki/Kim and Paton relative configurations, were in keeping with the structure of the actual ocellenynes having a 6,10-*anti*-configuration, i.e., being of the Suzuki or Kim and Paton type (structures **5** or **8**).^9^

**Figure 1:**
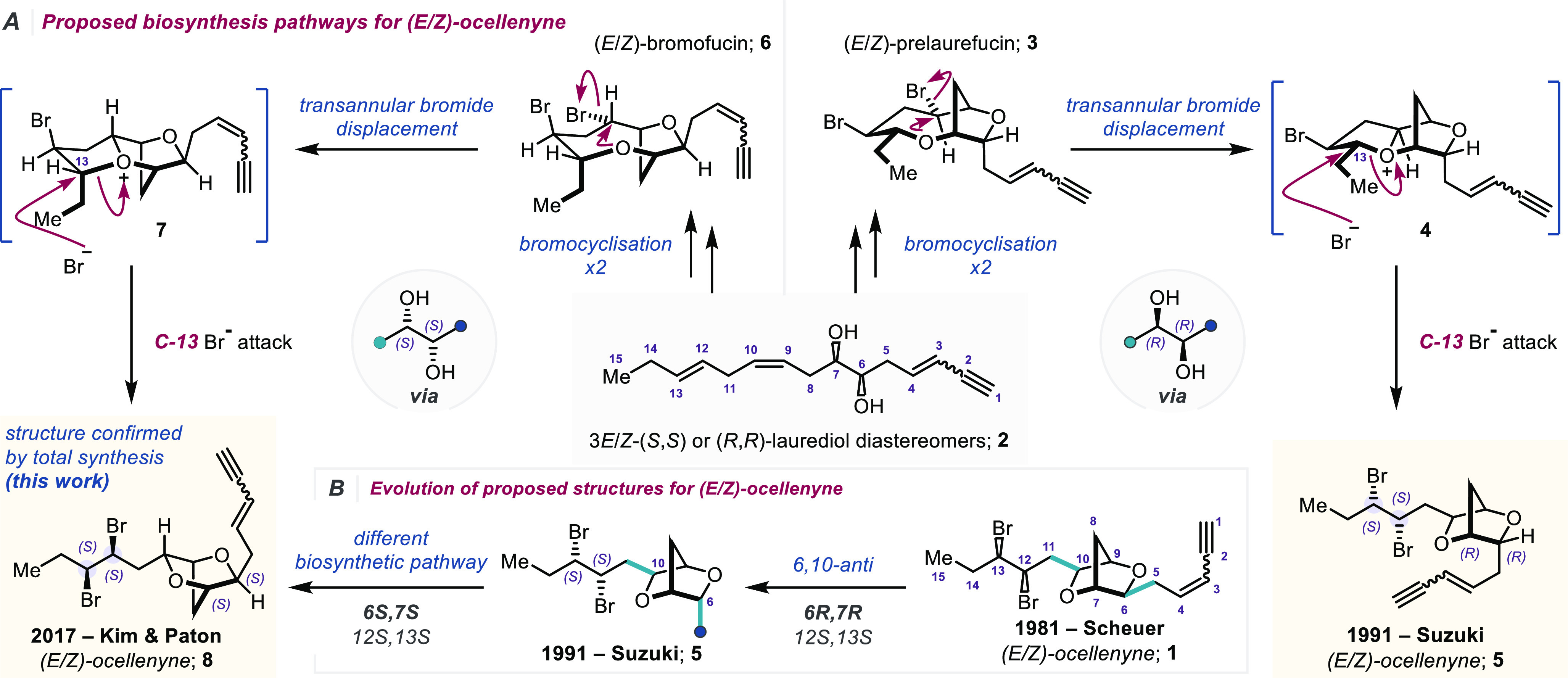
(A) Proposed biosynthesis pathways for the ocellenynes.^2^ (B) Evolution of proposed structures for the ocellenynes.

We have long been fascinated by the wealth of architectural diversity in the *L*. natural product family that could arise biosynthetically by opening of the oxonium ions **4** and **7** with various nucleophiles. We have characterized the four oxonium ions **4** and **7** and studied their opening with a variety of nucleophiles, as well as completing the synthesis of numerous C_15_*Laurencia* natural products.^6,10,13,14^

Interestingly, exposure of the oxonium ions **4** and **7** (carrying aluminate counter anions) to bromide did not result in any opening at C-13, and we were, therefore, unable to confirm whether the reassigned structures of the ocellenynes (**5** or **8**) were the actual structures of the natural products.^10^ Herein, we delineate a 10-step synthesis of both biosynthetically plausible ocellenyne diastereomers, namely the Kim and Paton ocellenynes **8** and the enantiomers of the Suzuki ocellenynes (i.e., *ent*-**5**) from known epoxide **9** (Scheme 1).^10,15^ The data for our synthetic (*E*)- and (*Z*)-**8** is in good agreement with the data for the natural products, and on the basis of these data, we assign/confirm the structures and absolute configurations of the ocellenyne natural products as represented by structures **8**.

**Scheme 1:**
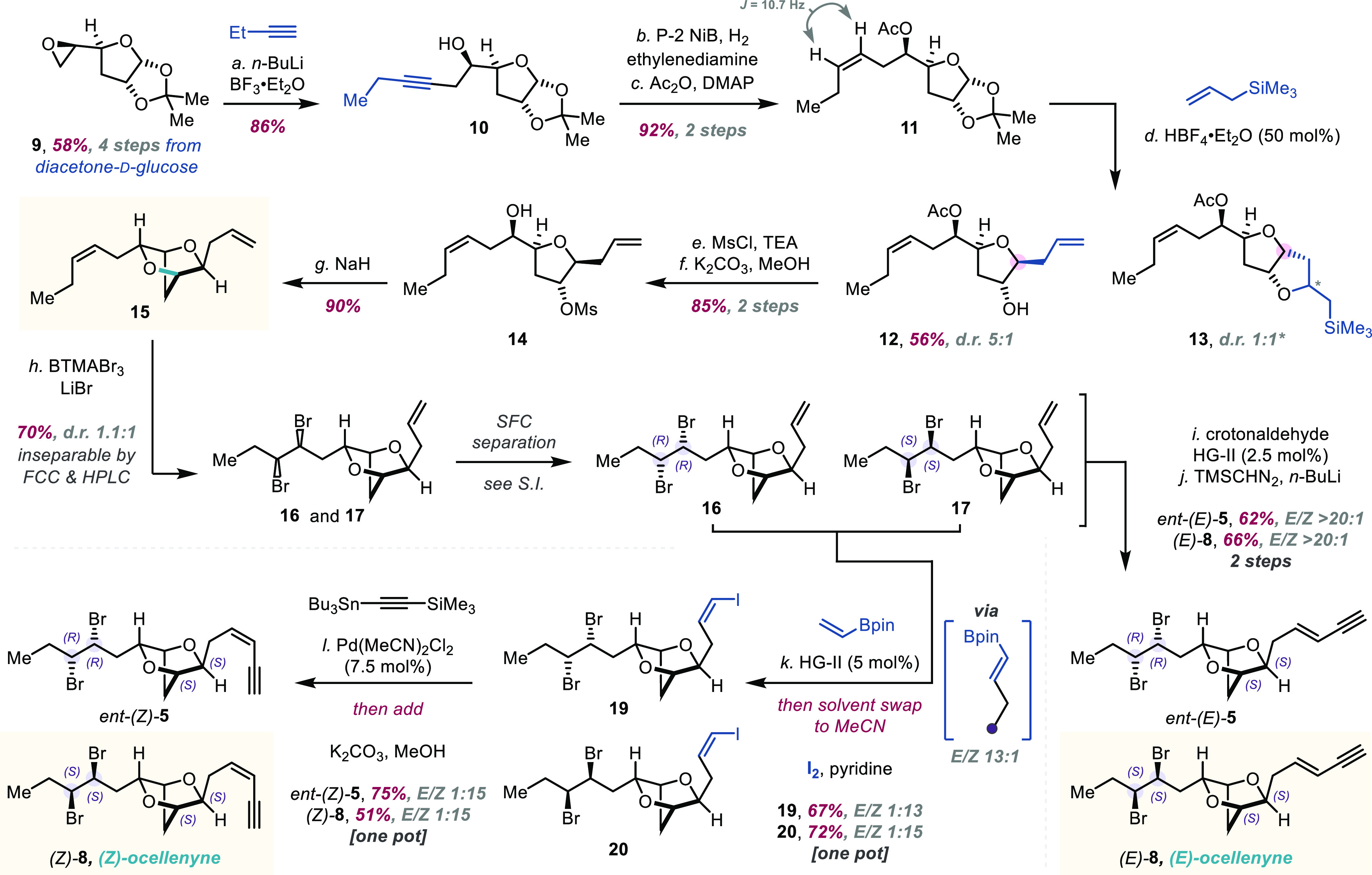
Total Synthesis of (*E*)- and (*Z*)-Ocellenyne Reagents and conditions: (*a*) 1-butyne (1.9 equiv), *n*-BuLi (1.9 equiv), BF_3_·Et_2_O (1.9 equiv), THF, −78 °C, 1 h, 86%. (*b*) Ni(OAc)_2_·4H_2_O (20 mol %), NaBH_4_ (20 mol %), ethylenediamine (40 mol %), H_2_ atmosphere, EtOH, rt, 18 h, 99%. (*c*) Ac_2_O (3.0 equiv), DMAP (3.0 equiv), CH_2_Cl_2_, rt, 2 h, 93%. (*d*) Allyltrimethylsilane (7.0 equiv), HBF_4_·Et_2_O (50 mol %), CH_2_Cl_2_, reflux, 4 h, 56% d.r. 5:1. (*e*) MsCl (1.2 equiv), TEA (1.2 equiv), CH_2_Cl_2_, 0 °C to rt, 2 h. (*f*) K_2_CO_3_ (2.0 equiv), MeOH/H_2_O (7:1), rt, 2 h, 85% over 2 steps. (*g*) NaH (5.0 equiv), DMF, 0 °C to rt, 18 h, 90%. (*h*) benzyltrimethylammonium tribromide (1.0 equiv), LiBr (10.0 equiv), MeCN, 0 °C, 1 h, 70% yield (inseparable mixture of diastereomers) d.r. **16**/**17**, 1.1:1. (*i*) Crotonaldehyde (5.0 equiv), HG-II (2.5 mol %), CH_2_Cl_2_, reflux, 3 h. (*j*) TMSCHN_2_ (1.3 equiv), *n*-BuLi (1.2 equiv), THF, −78 °C for 1 h then 0 °C for 10 min, *ent-*(*E*)*-***5** 62% over 2 steps, *E*/*Z* > 20:1; (*E*)*-***8** 66% over 2 steps, *E*/*Z* > 20:1. (*k*) Vinylboronic acid pinacol ester (1.5 equiv), HG-II (5 mol %), CH_2_Cl_2_, reflux, 3 h, then solvent swap to MeCN, pyridine (5.0 equiv), I_2_ (10.0 equiv), 25 °C, 5 h, **19** 67% *E*/*Z* 1:13; **20** 72% *E*/*Z* 1:15. (*l*) Trimethyl[(tributylstannyl)ethynyl]silane (2.0 equiv), Pd(MeCN)_2_Cl_2_ (7.5 mol %), DMF, 25 °C, 18 h, then add K_2_CO_3_ (3.0 equiv), MeOH, rt, 15 min, *ent*-(*Z*)*-***5** 75% *E*/*Z* 1:15; (*Z*)*-***8** 51% *E*/*Z* 1:15.

Inspired by the Scheuer et al. bromination of an ocellenyne derivative,^1^ we reasoned that selective bromination of diene **15** would yield both desired diastereomers of the *syn*-vicinal-dibromide (see **16** and **17**) en route to *ent-(E/Z)-***5** and (*E/Z*)*-***8**, with the *syn*-stereochemistry being set by the *cis*-olefin geometry of **15** (Scheme 1). This diene **15** could ultimately be obtained from diacetone-d-glucose, which utilizes chemistry previously developed by our group.^10,14^ We started from known epoxide **9**(10,15) and added lithiated 1-butyne to the terminal epoxide position using the method by Yamaguchi and Hirao^16^ to yield homopropargylic alcohol **10** (Scheme 1).^17^ In the presence of P-2 nickel boride catalyst,^18^ hydrogen, and ethylenediamine,^19^ the alkyne underwent stereospecific reduction to give the corresponding (*Z*)-homoallylic alcohol, which upon acetylation, gave the acetate **11**. Conditions previously employed using boron trifluoride etherate to enable Hosomi–Sakurai allylation^20^ at the anomeric position of **11** resulted in a low diastereomeric ratio (d.r.) (2:1) of the desired bishomoallylic alcohol **12** (major diastereomer shown).^10,21^ Interestingly, exposure of the acetate **11** to substoichiometric tetrafluoroboric acid diethyl ether complex in the presence of excess allyltrimethylsilane^22^ gave the allylated product **12** with an improved d.r. (5:1) in favor of the desired product, with the side product **13** being readily separated (see Supporting Information for further detail). Mesylation of **12**, followed by acetate cleavage, gave the alcohol **14**. Exposure of **14** to sodium hydride promoted intramolecular S_N_2 cyclization, which gave the key 6,10-*anti*-2,5-dioxabicyclo[2.2.1]heptane **15** in 90% yield. Initial bromination studies with **15** showed good selectivity for the internal olefin;^23^ however, significant degradation was observed, potentially from reaction of the pendant cyclic ether with the intermediate bromonium ion.^10,14,24^ This problem was ameliorated by addition of excess lithium bromide, which delivered the pair of *syn*-12,13-dibromide diastereomers **16** and **17** (d.r. = 1.1:1), which were separated by supercritical fluid chromatography (SFC).^25^ Derivatization of **17** by Lemieux–Johnson oxidation,^26^ followed by addition of 2,4-dinitrophenylhydrazine, gave the 2,4-dinitrophenylhydrazone **18** (Figure 2). Subsequent single crystal X-ray diffraction study^27^ of **18** allowed determination of the absolute configuration of the *syn*-12,13-dibromide as (12*S*,13*S*) (Figure 2), as well as confirmation of the 6,10-*anti* configuration of the two side chains; the absolute configuration of the derivatives of **17** follows from the absolute configuration of **17**. The separated dibromides **16** and **17** were transformed into their respective (*E*)-enynes *ent-*(*E*)-**5** and (*E*)-**8** via cross-metathesis^28^ with crotonaldehyde and Hoveyda–Grubbs second generation catalyst (HG-II),^29^ followed by Colvin–Ohira alkyne homologation.^30^ Installation of the (*Z*)-enynes proved more challenging, with both the *syn*-12,13-dibromide moiety and aldehyde resulting from oxidative cleavage of **16** degrading under a variety of basic conditions (see Supporting Information). Keen to exploit our substrate’s compatibility with cross-metathesis, we adapted Grubbs’ one-pot procedure to form (*Z*)-vinyl halides via (*E*)-selective cross-metathesis of separated **16** and **17** with vinylboronic acid pinacol ester, followed by an *anti*-diiodination, *anti*-deboronoiodination sequence to generate (*Z*)-vinyl iodides **19** and **20** respectively.^31^ Finally, phosphine-free Stille cross-coupling of **19** and **20**,^32^ followed by *in situ* silyl group deprotection yielded the (*Z*)-enynes *ent*-(*Z*)-**5** and (*Z*)-**8**; attempted Sonogashira reactions with **19** and **20** resulted in degradation of the vicinal dibromide.^33^

**Figure 2:**
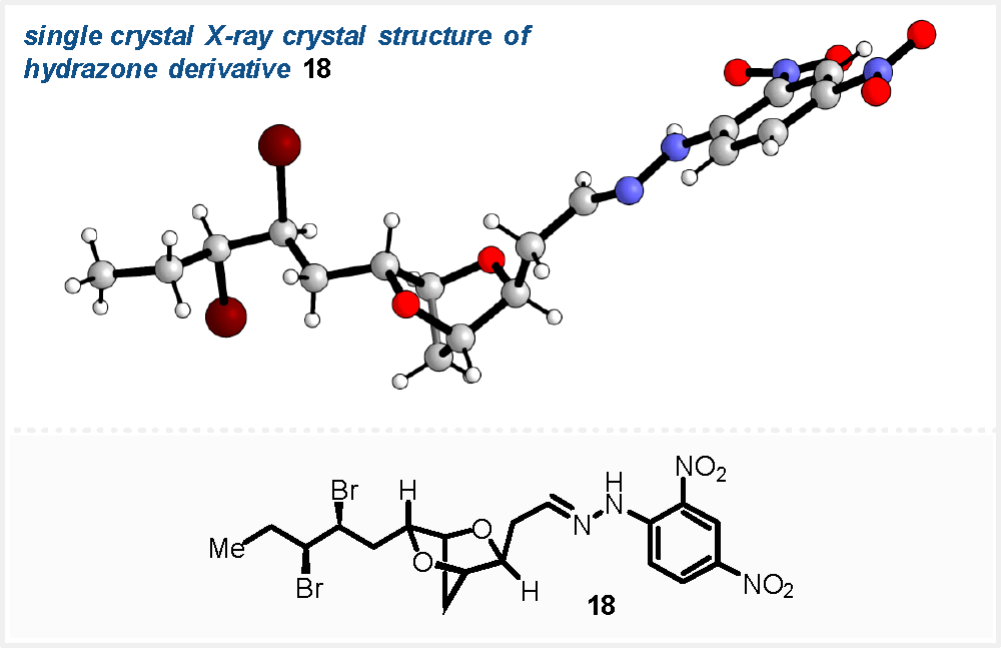
Single crystal X-ray crystal structure of 2,4-dinitrophenylhydrazone **18**.

The spectroscopic data of our synthetic *ent-*(*E*)- and *ent-*(*Z*)-**5** were not a good match with the spectroscopic data of the ocellenyne natural products listed in the isolation paper,^1^ which indicated that the natural ocellenynes do not have the structure (*E*/*Z*)-**5**. However, our spectroscopic data for (*E*)- and (*Z*)-**8** were a good match with the natural product data, albeit with some minor discrepancies (see Supporting Information for data comparison and discussion). On this basis, we assign the structures of the (*E*/*Z*)-ocellenynes as (*E*/*Z*)-**8**, which is in keeping with their proposed biosynthesis from (*S*,*S*)-laurediols (*S*,*S*)-**2** and with the Kim and Paton DFT prediction.

In conclusion, we have completed the total synthesis of both (*E*)- and (*Z*)-ocellenyne *(E/Z)-***8** and assigned the configuration of these natural products as (6*S*,7*S*,9*S*,10*R*,12*S*,13*S*), in keeping with the proposed biosynthetic pathway for the ocellenynes starting from the (*S*,*S*)-laurediols (*S*,*S*)-**2**.

## Data Availability

The data underlying this study are available in the published article and its online Supporting Information.

## Supporting Information Available

Experimental procedures and spectroscopic and analytical data for all new compounds, including copies of the NMR spectra (PDF)

## References

[ref1] Schulte G. R.; Chung M. C. H.; Scheuer P. J. Two bicyclic C15 enynes from the sea hare *Aplysia oculifera*. J. Org. Chem. 1981, 46, 3870–3873. 10.1021/jo00332a022.

[ref2] In structures **1** and **2**, wedge outlines are used to represent either the configuration shown or its mirror image, i.e., the diol in **2** has either the (*R*,*R*) or (*S,S*) configuration, as does the dibromide in **1**, see: Maehr H. A proposed new convention for graphic presentation of molecular geometry and topography. J. Chem. Educ. 1985, 62, 114–120. 10.1021/ed062p114.

[ref3] aWang B. G.; Gloer J. B.; Ji N. Y.; Zhao J. C. Halogenated organic molecules of Rhodomelaceae origin: chemistry and biology. Chem. Rev. 2013, 113, 3632–85. 10.1021/cr9002215. 23448097

[ref4] aFukuzawa A.; Aye M.; Nakamura M.; Tanura M.; Murai A. Structure elucidation of laureoxanyne, a new nonisoprenoid C-15-enyne, using lactoperoxidase. Tetrahedron Lett. 1990, 31, 4895–4898. 10.1016/S0040-4039(00)97762-1.

[ref5] Kikuchi H.; Suzuki T.; Kurosawa E.; Suzuki M. The Structure Of Notoryne, A Halogenated C15 Nonterpenoid With A Novel Carbon Skeleton From The Red Alga *Laurencia Nipponica* Yamada. Bull. Chem. Soc. Jpn. 1991, 64, 1763–1775. 10.1246/bcsj.64.1763.

[ref6] Dyson B. S.; Burton J. W.; Sohn T. I.; Kim B.; Bae H.; Kim D. Total synthesis and structure confirmation of elatenyne: success of computational methods for NMR prediction with highly flexible diastereomers. J. Am. Chem. Soc. 2012, 134, 11781–11790. 10.1021/ja304554e. 22758928

[ref7] In ref (9), Kim et al. highlight that the ocellenynes formed from the opening of **4** with bromide were erroneously drawn with 6,10-*syn* stereochemistry (**1**) instead of with 6,10-*anti* stereochemistry (**5**) in Scheme 4 of the Suzuki et al. paper (ref 5).

[ref8] The structures of the ocellenynes proposed by Suzuki et al. (structures **5**) were based purely on a reasonable biosynthetic postulate involving the oxonium ions **4**.

[ref9] Jeong D.; Sohn T. I.; Kim J. Y.; Kim G.; Kim D.; Paton R. S. Construction of 6,10-*syn*- and -*anti*-2,5-dioxabicyclo[2.2.1]heptane skeletons via oxonium ion formation/fragmentation: prediction of structure of (*E*)-ocellenyne by NMR calculation. Org. Lett. 2017, 19, 6252–6255. 10.1021/acs.orglett.7b03226. 29112433

[ref10] Chan H. S. S.; Nguyen Q. N. N.; Paton R. S.; Burton J. W. Synthesis, Characterization, and Reactivity of Complex Tricyclic Oxonium Ions, Proposed Intermediates in Natural Product Biosynthesis. J. Am. Chem. Soc. 2019, 141, 15951–15962. 10.1021/jacs.9b07438. 31560524

[ref11] In footnote 16 of ref 9, Kim et al. note that the ocellenynes may have the absolute configuration represented by **8** on the basis of plausible biosynthetic arguments (see p S177 of the Supporting Information of ref 9).

[ref12] Smith S. G.; Goodman J. M. Assigning Stereochemistry to Single Diastereoisomers by GIAO NMR Calculation: The DP4 Probability. J. Am. Chem. Soc. 2010, 132, 12946–12959. 10.1021/ja105035r. 20795713

[ref13] aShepherd D. J.; Broadwith P. A.; Dyson B. S.; Paton R. S.; Burton J. W. Structure Reassignment of Laurefurenynes A and B by Computation and Total Synthesis. Chem. Eur. J. 2013, 19, 12644–12648. 10.1002/chem.201302349. 23963665 PMC4280896

[ref14] Chan H. S. S.; Thompson A. L.; Christensen K. E.; Burton J. W. Forwards and backwards – synthesis of Laurencia natural products using a biomimetic and retrobiomimetic strategy incorporating structural reassignment of laurefurenynes C–F. Chem. Sci. 2020, 11, 11592–11600. 10.1039/D0SC04120C. 34094406 PMC8162873

[ref15] Rauter A. P.; Figueiredo J.; Ismael M.; Canda T.; Font J.; Figueredo M. Efficient synthesis of α,β-unsaturated γ-lactones linked to sugars. Tetrahedron: Asymmetry 2001, 12, 1131–1146. 10.1016/S0957-4166(01)00197-5.

[ref16] Yamaguchi M.; Hirao I. An efficient method for the alkynylation of oxiranes using alkynyl boranes. Tetrahedron Lett. 1983, 24, 391–394. 10.1016/S0040-4039(00)81416-1.

[ref17] Das S.; Ramana C. V. A formal total synthesis of (−)-kumausallene. Tetrahedron 2015, 71, 8577–8584. 10.1016/j.tet.2015.09.027.

[ref18] aBrown H. C.; Brown C. A. The Reaction of Sodium Borohydride with Nickel Acetate in Ethanol Solution--A Highly Selective Nickel Hydrogenation Catalyst. J. Am. Chem. Soc. 1963, 85, 1005–1006. 10.1021/ja00890a041.

[ref19] Brown C. A.; Ahuja V. K. P-2 “nickel” catalyst with ethylenediamine, a novel system for highly stereospecific reduction of alkynes to cis-olefins. J. Chem. Soc., Chem. Commun. 1973, 553–554. 10.1039/C39730000553.

[ref20] Akira H.; Masahiko E.; Hideki S. Allylsilanes as synthetic intermediates. II. Synthesis of homoallyl ethers from allylsilanes and acetals promoted by titanium tetrachloride. Chem. Lett. 1976, 5, 941–942. 10.1246/cl.1976.941.

[ref21] García-Tellado F.; de Armas P.; Marrero-Tellado J. J. Highly 1,2-trans Stereoselective Allylations of 1,2-O-Isopropylidene-Protected Glycofuranosides. Angew. Chem., Int. Ed. 2000, 39, 2727–2729. 10.1002/1521-3773(20000804)39:15<2727::AID-ANIE2727>3.0.CO;2-I. 10934407

[ref22] The reaction of HBF_4_·OEt_2_ with allyltrimethylsilane most likely generates BF_3_·OEt_2_. For the reaction of allyltrimethylsilane with trifluoromethane sulfonic acid, see: Olah G. A.; Husain A.; Gupta B. G. B.; Salem G. F.; Narang S. C. Synthetic methods and reactions 104. Silylations with in situ generated trimethylsilyl triflate reagent systems. J. Org. Chem. 1981, 46, 5212–5214. 10.1021/jo00338a030.

[ref23] Ashtekar K. D.; Marzijarani N. S.; Jaganathan A.; Holmes D.; Jackson J. E.; Borhan B. A new tool to guide halofunctionalization reactions: the halenium affinity (HalA) scale. J. Am. Chem. Soc. 2014, 136, 13355–62. 10.1021/ja506889c. 25152188 PMC4183602

[ref24] aSnyder S. A.; Treitler D. S.; Brucks A. P.; Sattler W. A general strategy for the stereocontrolled preparation of diverse 8- and 9-membered *Laurencia*-type bromoethers. J. Am. Chem. Soc. 2011, 133, 15898–15901. 10.1021/ja2069449. 21919540

[ref25] The mixture of dibromides **16** and **17** were inseparable by flash column chromatography (FCC) and by high performance liquid chromatography (HPLC) but could be separated by supercritical fluid chromatography (SFC).

[ref26] Yu W.; Mei Y.; Kang Y.; Hua Z.; Jin Z. Improved Procedure for the Oxidative Cleavage of Olefins by OsO4–NaIO4. Org. Lett. 2004, 6, 3217–3219. 10.1021/ol0400342. 15355016

[ref27] aPalatinus L.; Chapuis G. SUPERFLIP - a computer program for the solution of crystal structures by charge flipping in arbitrary dimensions. J. Appl. Crystallogr. 2007, 40, 786–790. 10.1107/S0021889807029238.

[ref28] aChatterjee A. K.; Choi T. L.; Sanders D. P.; Grubbs R. H. A general model for selectivity in olefin cross metathesis. J. Am. Chem. Soc. 2003, 125, 11360–11370. 10.1021/ja0214882. 16220959

[ref29] aGarber S. B.; Kingsbury J. S.; Gray B. L.; Hoveyda A. H. Efficient and recyclable monomeric and dendritic Ru-based metathesis catalysts. J. Am. Chem. Soc. 2000, 122, 8168–8179. 10.1021/ja001179g.

[ref30] aColvin E. W.; Hamill B. J. One-step conversion of carbonyl compounds into acetylenes. J. Chem. Soc., Chem. Commun. 1973, 151–152. 10.1039/c39730000151.

[ref31] aMorrill C.; Grubbs R. H. Synthesis of Functionalized Vinyl Boronates via Ruthenium-Catalyzed Olefin Cross-Metathesis and Subsequent Conversion to Vinyl Halides. J. Org. Chem. 2003, 68, 6031–6034. 10.1021/jo0345345. 12868943

[ref32] Stille J. K.; Simpson J. H. Stereospecific palladium-catalyzed coupling reactions of vinyl iodides with acetylenic tin reagents. J. Am. Chem. Soc. 1987, 109, 2138–2152. 10.1021/ja00241a035.

[ref33] Sonogashira K. Development of Pd–Cu catalyzed cross-coupling of terminal acetylenes with sp2-carbon halides. J. Organomet. Chem. 2002, 653, 46–49. 10.1016/S0022-328X(02)01158-0.

