# Supplementary material for: Total Synthesis and Structure Confirmation of (E) and (Z)-Ocellenyne
Source: Org Lett. 2022 Dec 12;24(50):9174–8. doi: 10.1021/acs.orglett.2c03524 (PMC9791679; doi:10.1021/acs.orglett.2c03524)

Supplementary Information

for

Total Synthesis and Structure Confirmation of (*E*) and (*Z*)-Ocellenyne

Harry B. Hicks,† Daniel S. Brown,† Hau Sun Sam Chan,† Bruno A. Sousa‡, Kirsten E. Christensen† and Jonathan W. Burton*,†

†Chemistry Research Laboratory, University of Oxford, Mansfield Road, Oxford, OX1 3TA, U.K.
‡Vertex Pharmaceuticals, 86-88 Jubilee Avenue, Milton Park, Abingdon, OX14 4RW, U. K.

Email: jonathan.burton@chem.ox.ac.uk

1. General Experimental 3
2. Proposed Biosynthesis Pathway for (*E*/*Z*)-Ocellenyne 4
3. Synthesis and Characterization of Novel Compounds/Natural Products 5
   3.1. Synthesis of key 6,10-*anti*-2,5-dioxabicyclo[2.2.1]heptane diene 15 5
   3.2. Bromination, aldehyde degradation and determination of stereochemistry 15
   3.3. Enyne syntheses to generate (*E*)- and (*Z*)-ocellenynes (8) and the enantiomers of the Suzuki (*E*)- and (*Z*)-ocellenynes (5) 24
4. Comparison of Synthetic and Natural Ocellenyne Data 39
5. Comparison of ^{1}H NMR Spectra for Synthetic vs Simulated Natural Ocellenyne 46
6. References 52
7. NMR Spectra for Novel Compounds 53

1. General Experimental

^{1}H and ^{13}C NMR spectra were recorded on Bruker AV 600 (600/151 MHz), Bruker AV 500 (500/125 MHz) and Bruker AV 400 (400/100 MHz) spectrometers. Proton and carbon chemical shifts are quoted in ppm and referenced to residual protonated solvent. Resonances are described as s (singlet), d (doublet), t (triplet), q (quartet), m (multiplet), br (broad), dd (double doublet) and so on. Coupling constants (J) are given in Hz and are rounded to the nearest 0.1 Hz. Low resolution mass spectra (LRMS) were recorded on a Fisons Platform spectrometer (ESI). High resolution mass spectra (HRMS) were recorded by the mass spectrometry staff at the Chemistry Research Laboratory, University of Oxford, using a Bruker Daltronics microTOF spectrometer (ESI) or a Micromass GCT (FI). m/z values are reported in Daltons. High resolution values are calculated to four decimal places from the molecular formula, all found values being within a tolerance of 5.0 ppm. Infrared (IR) spectra were recorded on a Bruker Tensor 27 Fourier Transform spectrometer using diamond ATR. Absorption maxima (ν_{max}) are described as strong, medium, weak and broad and are quoted in wavenumbers (cm^{-1}). Optical rotations were measured using a Perkin-Elmer 241 polarimeter in a cell of 1.0 dm path length (l). TLC was performed on Merck DCAlufolien 60 F254 0.2 mm precoated plates and visualised using an acidic vanillin or basic potassium permanganate dip. Retention factors (R_f) are reported with the solvent system used in parentheses. Flash column chromatography was performed on Merck 60 silica (particle size 40–63 µm, pore diameter 60 Å) and the solvent system used is recorded in parentheses. Supercritical fluid chromatography (SFC) was performed on either a Waters SFC Prep150 or Waters UPCC instrument using an appropriate chiral stationary phase column, specified in the individual experiment. All non-aqueous reactions were carried out in flame-dried glassware under an inert atmosphere of nitrogen or argon and employing standard techniques for handling air sensitive materials. Solvents and commercially available reagents were dried and purified before use, as appropriate. For reactions that require heating, DrySyn® heating blocks were used as the heat source. Compounds were named using ChemDraw and the assignments of NMR spectra were conducted on MestReNova, using 2D NMR experiments including ^{1}H –^{1}H–COSY, ^{1}H–^{13}C–HSQC, ^{1}H–^{13}C–HMBC and ^{1}H –^{1}H–NOESY when necessary. The numbering system for NMR assignments of compounds does not follow IUPAC rules. Low temperature[1] single crystal X-ray diffraction data were collected using a (Rigaku) Oxford Diffraction SuperNova diffractometer. Raw frame data were reduced using CrysAlisPro and the structures were solved using 'Superflip'[2] before refinement with CRYSTALS[3] as per the SI (CIF). Full refinement details are given in the Supporting Information (CIF); Crystallographic data have been deposited with the Cambridge Crystallographic Data Centre (CCDC 2208812) and can be obtained via www.ccdc.cam.ac.uk/data_request/cif.

2. Proposed Biosynthesis Pathway for (E/Z)-Ocellenyne

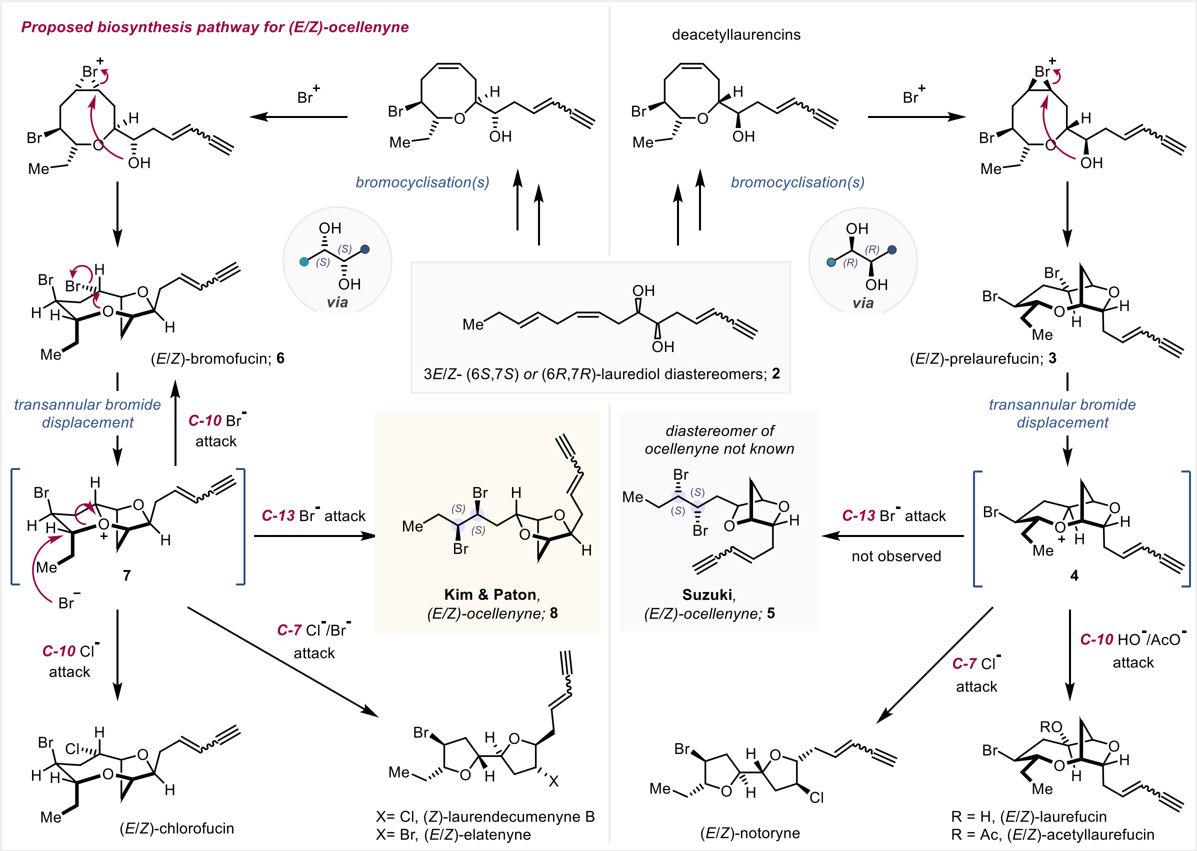

Figure S1. Proposed biosynthesis of (E/Z)-ocellenyne 8 from 3E/Z, S,S-laurediol (S,S)-2 and of the Suzuki (E/Z)-ocellenynes 5 from 3E/Z, R,R-laurediol (R,R)-2.

3. Synthesis and Characterization of Novel Compounds/Natural Products

All ^{1}H and ^{13}C NMR assignments are numbered in accordance with the numbering system used for ocellenyne and other C15-acetogenin *Laurencia* natural products.[4]

(*R*)-1-((3a*R*,5*S*,6a*R*)-2,2-dimethyltetrahydrofuro[2,3-*d*][1,3]dioxol-5-yl)ethane-1,2-diol was prepared previously in the referenced publication[5].

3.1. Synthesis of key 6,10-*anti*-2,5-dioxabicyclo[2.2.1]heptane diene 15

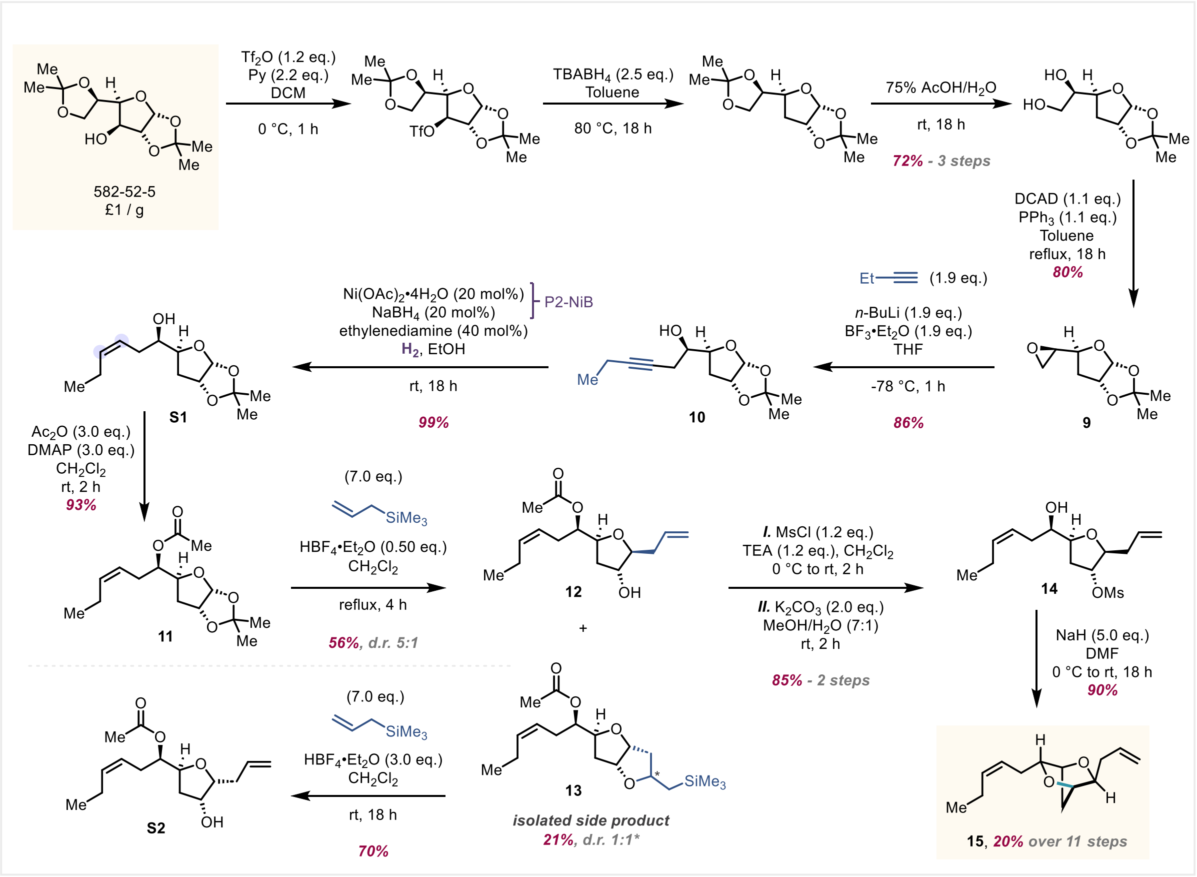

Scheme S1. 11 Step synthesis of key diene 15 from diacetone-D-glucose.

(3a*R*,5*S*,6a*R*)-2,2-Dimethyl-5-((*R*)-oxiran-2-yl)tetrahydrofuro[2,3-*d*][1,3]dioxole (9)

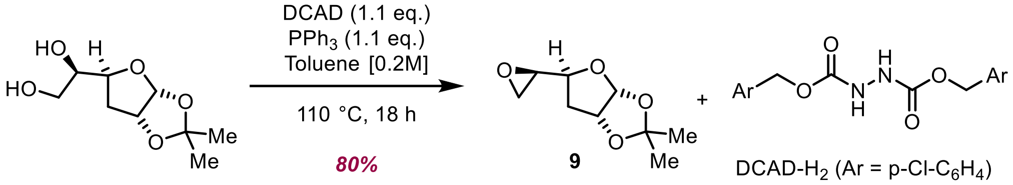

According to the modified procedures of Hanaya and Chan[6]: (*R*)-1-((3a*R*,5*S*,6a*R*)-2,2-dimethyltetrahydrofuro[2,3-d][1,3]dioxol-5-yl)ethane-1,2-diol (5.5 g, 27.0 mmol) and triphenylphosphine (7.8 g, 29.7 mmol, 1.1 eq.) were dissolved in toluene (150 mL). Di-(4-chlorobenzyl)azodicarboxylate (DCAD) (10.9 g, 29.7 mmol, 1.1 eq.) was added to the reaction mixture. The reaction mixture was then stirred and heated at 110 °C for 18 h. TLC analysis (30% acetone/petroleum ether 40-60) at this stage indicated completion of reaction. The reaction mixture was cooled to 0 °C, filtered and washed on the filter with ice-cold toluene (2 x 40 mL). The solid white filter cake (DCAD-H_2) was dried on the filter and recycled to give DCAD according to the procedure of Lipshutz.[7] The filtrate was then concentrated under reduced pressure to yield a syrup. The syrup was diluted with diethyl ether (50 mL) and sonicated. The sonication brought about the precipitation of the triphenylphosphine oxide by-product. The mixture was then filtered, the solids were washed with ice cold diethyl ether (3 x 15 mL) and the filtrate was concentrated and purified by flash column chromatography (250 g silica, CV = 500 mL, 5-12% acetone/petroleum ether 40-60) to give the desired compound 9 as a colourless oil (4.0 g, 21.7 mmol, 80%).

R_f = 0.38 (30% EtOAc/petroleum ether 40-60).

^{1}H NMR (400 MHz, $CDCl_3$) δ 5.84 (d, *J* = 3.6 Hz, 1H), 4.75 (dd, *J* = 4.7, 3.7 Hz, 1H), 4.21 (dt, *J* = 10.7, 4.4 Hz, 1H), 3.14 (td, *J* = 4.2, 2.6 Hz, 1H), 2.83 (dd, *J* = 5.0, 4.1 Hz, 1H), 2.61 (dd, *J* = 4.9, 2.7 Hz, 1H), 2.07 (dd, *J* = 13.3, 4.5 Hz, 1H), 1.72 (ddd, *J* = 13.3, 10.7, 4.8 Hz, 1H), 1.50 (s, 3H), 1.32 (s, 3H).

LRMS (ESI) $[M+Na]^+$: *m/z* 209.1.

$[\alpha]_D^{25}$ = -17.0 (c=1.0, $CHCl_3$). lit. $[\alpha]_D^{25}$ = −22.2 (c=0.1, $CHCl_3$)[5].

Spectroscopic and physical data are in accordance with literature data.[5]

(*R*)-1-((3a*R*,5S,6a*R*)-2,2-Dimethyltetrahydrofuro[2,3-*d*][1,3]dioxol-5-yl)hex-3-yn-1-ol (10)

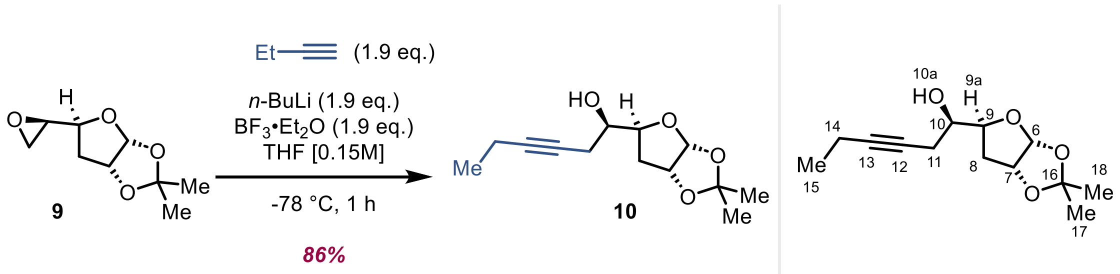

According to the modified procedure of Ramana:[8] Butyne (2.7 g, 50 mmol, 1.9 eq.) was quickly taken out of a -24 °C freezer and charged immediately into a vessel at 0 °C. The reaction mixture was then cooled to -78 °C and stirred before THF (110 ml) was added slowly. *n*-BuLi (2.5 M in hexanes, 26 mL, 50 mmol, 1.9 eq.) was added dropwise before the reaction mixture was warmed to 0 °C and stirred for 30 mins. After this time, the solution was cooled to -78 °C and boron trifluoride diethyl etherate (6.0 mL, 50 mmol, 1.9 eq.) was added. Compound 9 (5.5 g, 26.3 mmol, 1.0 eq.) was dissolved in THF (20 mL) and then added to the reaction mixture, which was then stirred at -78 °C for 1 h. The reaction mixture was quenched at -78 °C via addition of sat. aq. NH_4Cl solution and then warmed to rt. The organics were extracted with EtOAc (3 × 75 mL) and then combined, dried ($MgSO_4$), filtered and the solvent removed in vacuo to give a crude residue which was further purified by flash column chromatography (20-30% EtOAc/petroleum ether 40-60) to give the desired compound 10 (5.4 g, 23 mmol, 86%) as a pale yellow oil that crystallises upon prolonged standing.

R_f = 0.39 (30% EtOAc/petroleum ether 40-60).

IR (ν_{max} cm^{-1}): 3474 (broad), 2979 (medium), 2937 (weak), 2159 (medium).

HRMS (ESI) *m/z* : $[M+Na]^+$ calculated for $C_{13}H_{20}NaO_4$ 263.1254; found 263.1253.

^1H NMR (600 MHz, $CDCl_3$) δ 5.81 (d, *J* = 3.7 Hz, 1H, H6), 4.75 (t, *J* = 4.3 Hz, 1H, H7), 4.31 (dt, *J* = 10.1, 4.4 Hz, 1H, H9a), 3.93 (td, *J* = 6.4, 4.3 Hz, 1H, H10), 2.45 – 2.33 (m, 2H, H11), 2.20 – 2.13 (m, 2H, H14), 2.09 (dd, *J* = 13.4, 4.5 Hz, 1H, H8), 1.85 (ddd, *J* = 13.0, 10.5, 4.8 Hz, 1H, H8'), 1.52 (s, 3H, H17/H18), 1.33 (s, 3H, H17/H18), 1.12 (td, *J* = 7.5, 1.1 Hz, 3H, H15). **OH10a not observed.*

^{13}C NMR (151 MHz, $CDCl_3$) δ 111.5 (C16), 105.5 (C6), 84.8 (C13), 80.8 (C7), 80.0 (C9), 74.5 (C12), 70.1 (C10), 32.8 (C8), 26.9 (C17/C18), 26.4 (C17/C18), 23.8 (C11), 14.2 (C15), 12.5 (C14).

$[\alpha]_D^{25}$ = -30.0 (c=0.14, $CHCl_3$).

(*R*,*Z*)-1-((3a*R*,5*S*,6a*R*)-2,2-Dimethyltetrahydrofuro[2,3-*d*][1,3]dioxol-5-yl)hex-3-en-1-ol (S1)

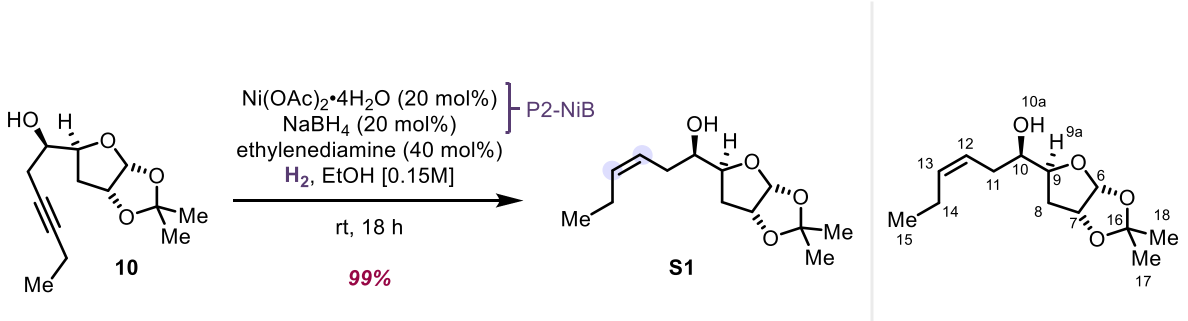

According to the modified procedure of Rychnovsky:[9] Sodium borohydride (0.18 g, 4.6 mmol, 20 mol%) was added to a stirred solution of $Ni(OAc)_2 \bullet 4H_2O$ (0.82 g, 4.6 mmol, 20 mol%) in EtOH (110 mL) and the flask was purged with H_2. Ethylenediamine (0.61 mL, 9.2 mmol, 40 mol%) was added and the catalyst mixture was sparged with H_2 for 20 mins. After this time, compound 10 (5.4 g, 23 mmol, 1.0 eq.) was dissolved in EtOH (20 mL) and added to the reaction mixture. Sparging with H_2 was continued for a further 20 mins before leaving the reaction mixture to stir for 18 h under an atmosphere of H_2. After this time the H_2 atmosphere was discharged and the solvent was removed in vacuo to give a crude residue, that was passed through a silica plug (30% EtOAc/petroleum ether 40-60) to give the desired compound S1 (5.4 g, 23 mmol, 99%) as a colourless oil.

R_f = 0.23 (30% EtOAc/petroleum ether 40-60).

IR (ν_{max} cm^{-1}): 3450 (broad), 2981 (medium), 1374 (medium).

HRMS (ESI) *m/z* : $[M+Na]^+$ calculated for $C_{13}H_{22}NaO_4$ 265.1411; found 265.1412.

^1H NMR (500 MHz, $CDCl_3$) δ 5.82 (d, *J* = 3.7 Hz, 1H, H6), 5.54 (dtt, *J* = 11.0, 7.1, 1.2 Hz, 1H, H13), 5.38 (dtt, *J* = 10.7, 7.1, 1.7 Hz, 1H, H12), 4.75 (t, *J* = 4.1 Hz, 1H, H7), 4.22 (ddd, *J* = 10.5, 4.7, 3.4 Hz, 1H, H9a), 3.92 (ddd, *J* = 7.8, 5.8, 3.4 Hz, 1H, H10), 2.27 – 2.13 (m, 2H, H11), 2.12 – 2.01 (m, 2H, H14), 1.98 (dd, *J* = 13.3, 4.7 Hz, 1H, H8), 1.91 (ddd, *J* = 13.4, 10.5, 4.7 Hz, 1H, H8'), 1.51 (s, 3H, H17/H18), 1.32 (s, 3H, H17/H18), 0.97 (t, *J* = 7.5 Hz, 3H, H15). **OH10a not observed.*

^{13}C NMR (126 MHz, $CDCl_3$) δ 135.0 (C13), 123.9 (C12), 111.4 (C16), 105.4 (C6), 80.8* (C7/C9), 80.7* (C7/C9), 70.6 (C10), 31.9 (C8), 30.9 (C11), 26.9 (C17/C18), 26.3 (C17/C18), 20.8 (C14), 14.3 (C15). **Resonances too close together to unambiguously assign.*

$[\alpha]_D^{25}$ = -14.4 (c=0.09, $CHCl_3$).

(R,Z)-1-((3aR,5S,6aR)-2,2-Dimethyltetrahydrofuro[2,3-d][1,3]dioxol-5-yl)hex-3-en-1-yl acetate (11)

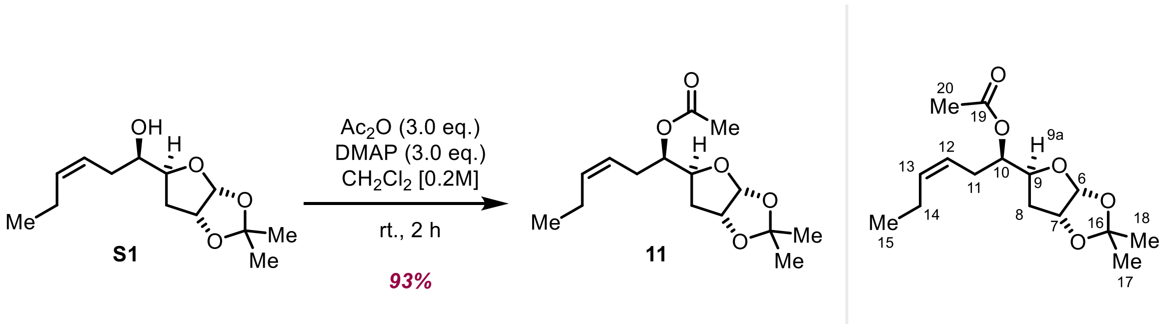

Compound S1 (5.4 g, 22 mmol, 1.0 eq.) and 4-(dimethylamino)pyridine (8.1 g, 70 mmol, 3.0 eq.) were dissolved in CH_2Cl_2 (110 mL). Acetic anhydride (6.3 mL, 70 mmol, 3.0 eq.) was added and the reaction mixture was stirred at rt for 2 h. The reaction mixture was quenched with sat. aq. $NaHCO_3$ (110 mL) and then extracted with CH_2Cl_2 (3 × 50 mL). The combined organic layers were dried ($MgSO_4$), filtered and concentrated in vacuo to give a crude residue which was purified further by flash column chromatography (5-15% EtOAc/ petroleum ether 40-60) to yield the desired compound 11 (5.9 g, 21 mmol, 93%) as a colourless oil.

R_f = 0.17 (10% EtOAc/petroleum ether 40-60).

IR (ν_{max} cm^{-1}): 2963 (medium), 1743 (strong), 1373 (medium).

HRMS (ESI) m/z : $[M+Na]^+$ calculated for $C_{15}H_{24}NaO_5$ 307.1516; found 307.1516.

^{1}H NMR (500 MHz, $CDCl_3$) δ 5.79 (d, J = 3.7 Hz, 1H, H6), 5.49 (dtt, J = 10.8, 7.4, 1.3 Hz, 1H, H13), 5.30 (dtt, J = 10.7, 7.4, 1.6 Hz, 1H, H12), 5.05 (dt, J = 7.1, 5.6 Hz, 1H, H10), 4.72 (t, J = 4.6 Hz, 1H, H7), 4.25 (dt, J = 10.2, 4.8 Hz, 1H, H9a), 2.39 – 2.32 (m, 2H, H11), 2.10 – 2.01 (m, 6H, H8, H14, H20), 1.77 (ddd, J = 13.4, 10.7, 4.8 Hz, 1H, H8'), 1.50 (s, 3H, H17/H18), 1.31 (s, 3H, H17/H18), 0.96 (t, J = 7.5 Hz, 3H, H15).

^{13}C NMR (126 MHz, $CDCl_3$) δ 170.4 (C19), 135.0 (C13), 123.0 (C12), 111.4 (C16), 105.6 (C6), 80.3 (C7), 78.5 (C9), 73.5 (C10), 34.8 (C8), 29.2 (C11), 26.9 (C17/C18), 26.3 (C17/C18), 21.2 (C20), 20.7 (C14), 14.3 (C15).

$[\alpha]_D^{25}$ = -15.7 (c=0.14, $CHCl_3$).

(*R*,*Z*)-1-((2*S*,4*R*,5*S*)-5-Allyl-4-hydroxytetrahydrofuran-2-yl)hex-3-en-1-yl acetate (12) and

(1*R*,*Z*)-1-((2*S*,3a*R*,6a*R*)-5-((trimethylsilyl)methyl)hexahydrofuro[3,2-*b*]furan-2-yl)hex-3-en-1-yl acetate (13)

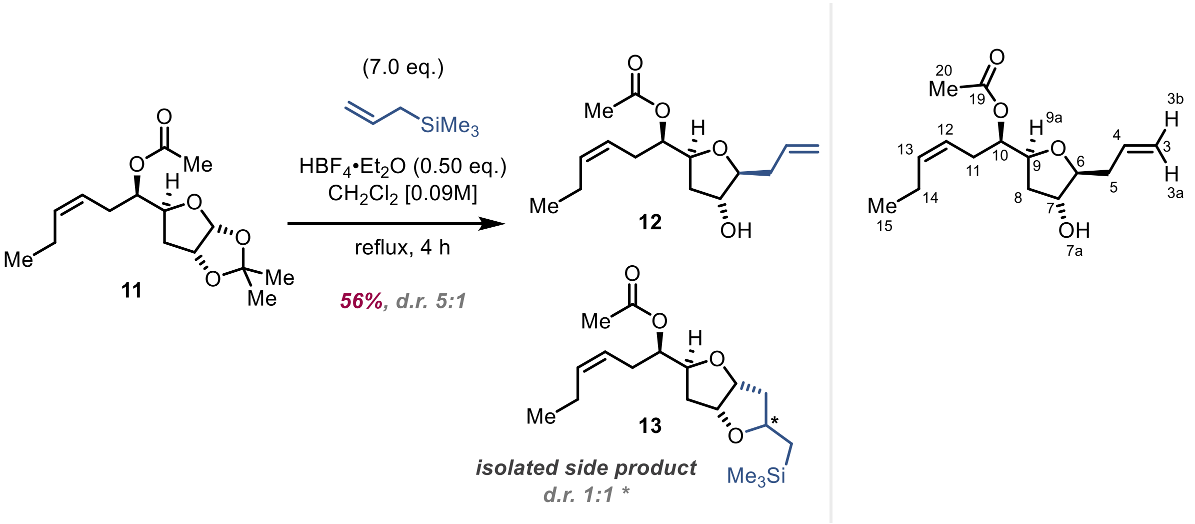

**This reaction likely proceeds via the in situ generation of BF_3•Et_2O.[10]*

Compound 11 (6.0 g, 21 mmol) and allyltrimethylsilane (23 mL, 150 mmol, 7.0 eq.) were dissolved in dry CH_2Cl_2 (210 mL) and stirred at room temperature. A freshly prepared 1 M solution of HBF_4•Et_2O in dry CH_2Cl_2 (10 mL, 10 mmol, 0.50 eq.) [1 M = 1.36 mL HBF_4•Et_2O + 8.64 mL CH_2Cl_2] was then added via syringe, with an exit needle also placed in the septum for 2 minutes to allow for gas evolution. After 30 minutes the reaction was heated to reflux for 4 h. After this time, the reaction mixture was cooled to rt and then quenched via the addition of sat. aq. $NaHCO_3$ solution (200 mL) and stirred vigorously until effervescence ceased. The organic layer was separated and the organics were further extracted with CH_2Cl_2 (3 × 75 mL). The combined organics were then dried ($MgSO_4$), filtered and the solvent removed in vacuo to give a crude residue which was further purified by flash column chromatography (20-30% EtOAc/petroleum ether 40-60) to give the desired compound 12 (3.1 g, 12 mmol, 56%, d.r. (12:S2) > 5:1) as a colourless oil.

Note that side product 13 was not collected during this large-scale experiment but was isolated in a different experiment employing identical conditions to the large-scale reaction. Starting with 1.27 mmol of compound 11 gave desired compound 12 (225 mg, 0.84 mmol, 66%, d.r. (12:S2) > 5:1) and side product 13 (93 mg, 0.27 mmol, 21%, d.r. = 1:1) as a clear oil. Minor diastereomer S2 was unable to be characterised at this stage, however it was isolated and characterised in a later experiment (see page S11). **note: the longer this reaction is left the lower the d.r. becomes, as 13 is slowly converted into S2 under the reaction conditions.*

Characterisation of 12

R_f = 0.20 (30% EtOAc/petroleum ether 40-60).

IR (ν_{max} cm^{-1}): 3439 (broad), 2963 (medium), 1742 (strong), 1642 (weak), 1437 (medium), 1373 (medium).

HRMS (ESI) *m/z* : [M+Na]$^+$ calculated for $C_{15}H_{24}NaO_4$ 291.1567; found 291.1566.

^{1}H NMR (500 MHz, $CDCl_3$) δ 5.81 (ddt, *J* = 17.1, 10.2, 7.4 Hz, 1H, H4), 5.48 (dtt, *J* = 10.8, 7.3, 1.7 Hz, 1H, H13), 5.29 (dtt, *J* = 10.8, 7.5, 1.6 Hz, 1H, H12), 5.13 – 5.05 (m, 2H, H3a, H3b), 5.02 (dt, *J* = 7.6, 5.1 Hz, 1H, H10), 4.19 (ddd, *J* = 8.9, 6.4, 4.8 Hz, 1H, H9a), 4.11 (dt, *J* = 6.2, 3.1 Hz, 1H, H7), 3.80 (td, *J* = 6.5, 3.2 Hz, 1H, H6), 2.41 – 2.30 (m, 2H, H11), 2.30 – 2.18 (m, 2H, H5), 2.08 – 1.96 (m, 6H, H8, H14, H20), 1.87 (ddd, *J* = 13.1, 6.4, 2.9 Hz, 1H, H8'), 0.95 (t, *J* = 7.5 Hz, 3H, H15). **OH7a not observed.* The configuration of 12 at C6 is assigned by comparison with NMR data from previously reported compounds.[5] Ultimately, the configuration at C6 is confirmed from the single crystal X-ray structure of 18.

^{13}C NMR (126 MHz, $CDCl_3$) δ 170.5 (C19), 134.8 (C13), 134.2 (C4), 123.2 (C12), 117.7 (C3), 85.8 (C6), 78.6 (C9), 75.3 (C7), 74.3 (C10), 38.6 (C5), 35.9 (C8), 29.0 (C11), 21.3 (C20), 20.7 (C14), 14.3 (C15).

Characterisation of side product 13

R_f = 0.36 (15% EtOAc/petroleum ether 40-60).

IR (ν_{max} cm^{-1}): 2956 (medium), 1742 (strong), 1642 (weak), 1371 (medium).

HRMS (ESI) *m/z* : [M+Na]$^+$ calculated for $C_{18}H_{32}NaO_4Si$ 363.1963; found 363.1964.

**Compound unable to be unambiguously assigned due to multiple overlapping resonances from 1:1 diastereomeric mixture.*

^{1}H NMR (500 MHz, $CDCl_3$) δ 5.53 – 5.44 (m, 2H), 5.36 – 5.24 (m, 2H), 5.05 (dt, *J* = 7.6, 5.0 Hz, 1H), 4.98 (dt, *J* = 7.6, 5.1 Hz, 1H), 4.72 – 4.67 (m, 2H), 4.64 (t, *J* = 4.4 Hz, 1H), 4.35 (t, *J* = 4.9 Hz, 1H), 4.18 – 4.06 (m, 3H), 3.82 (tt, *J* = 9.5, 5.5 Hz, 1H), 2.41 – 2.24 (m, 5H), 2.17 (dd, *J* = 13.3, 4.6 Hz, 1H), 2.09 – 2.01 (m, 12H), 1.92 (ddd, *J* = 13.4, 9.2, 5.5 Hz, 1H), 1.75 (ddd, *J* = 13.2, 10.4, 5.2 Hz, 1H), 1.50 – 1.41 (m, 2H), 1.18 – 1.01 (m, 2H), 0.96 (t, *J* = 7.5 Hz, 6H), 0.93 – 0.84 (m, 1H), 0.77 (dd, *J* = 14.0, 9.1 Hz, 1H), 0.02 (s, 9H), 0.02 (s, 9H).

^{13}C NMR (126 MHz, $CDCl_3$) δ 170.6, 170.6, 134.9, 134.8, 123.3, 123.2, 85.1, 85.0, 82.6, 82.5, 80.7, 78.3, 78.0, 77.7, 74.4, 73.9, 43.6, 42.6, 36.5, 34.9, 29.1, 29.0, 24.4, 24.0, 21.3, 21.3, 20.7, 14.3, -0.8, -0.8.

Generation of minor diastereomer S2 from side product 13

(R,Z)-1-((2S,4R,5R)-5-Allyl-4-hydroxytetrahydrofuran-2-yl)hex-3-en-1-yl acetate (S2)

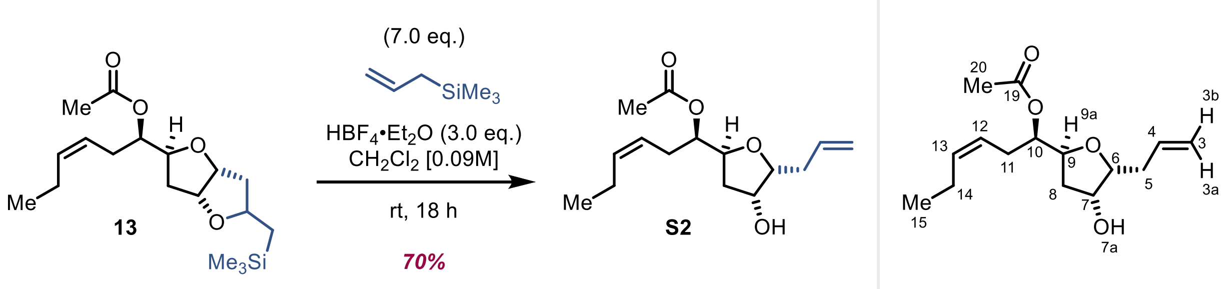

Compound 13 (88 mg, 0.26 mmol) and allyltrimethylsilane (0.29 mL, 1.82 mmol, 7.0 eq.) were dissolved in dry CH_2Cl_2 (1.8 mL) and stirred at room temperature. A freshly prepared 1 M solution of HBF_4•Et_2O in dry CH_2Cl_2 (0.78 mL, 0.78 mmol, 3.0 eq.) [1 M = 1.36 mL HBF_4•Et_2O + 8.64 mL CH_2Cl_2] was then added via syringe, with an exit needle also placed in the septum for 2 minutes to allow for gas evolution. The reaction was then stirred at rt for 18 h. After this time, the reaction mixture was quenched via the addition of sat. aq. $NaHCO_3$ solution (5 mL) and stirred vigorously until effervescence ceased. The organic layer was separated and the organics were further extracted with CH_2Cl_2 (3 × 5 mL). The combined organics were then dried ($MgSO_4$), filtered and the solvent removed in vacuo to give a crude residue which was further purified by flash column chromatography (20-30% EtOAc/petroleum ether 40-60) to give the desired compound S2 (49 mg, 0.18 mmol, 70%) as a colourless oil.

R_f = 0.26 (30% EtOAc/petroleum ether 40-60).

IR (ν_{max} cm^{-1}): 3439 (broad), 2963 (medium), 1742 (strong), 1642 (weak), 1437 (medium), 1373 (medium).

HRMS (ESI) m/z : $[M+Na]^+$ calculated for $C_{15}H_{24}NaO_4$ 291.1567; found 291.1566.

^{1}H NMR (500 MHz, $CDCl_3$) δ 5.84 (dddd, J = 17.6, 10.3, 7.5, 6.3 Hz, 1H, H4), 5.52 – 5.44 (m, 1H, H13), 5.34 – 5.26 (m, 1H, H12), 5.17 (dq, J = 17.2, 1.7 Hz, 1H, H3a), 5.09 (dq, J = 10.2, 1.3 Hz, 1H, H3b), 5.01 (dt, J = 7.4, 5.3 Hz, 1H, H10), 4.33 – 4.25 (m, 2H, H7, H9a), 3.86 (ddd, J = 7.7, 6.7, 2.8 Hz, 1H, H6), 2.47 (dtt, J = 14.5, 6.5, 1.6 Hz, 1H, H5), 2.42 – 2.29 (m, 3H, H5', H11), 2.10 – 1.99 (m, 7H, H8, H14, H20), 0.96 (t, J = 7.5 Hz, 3H, H15). **OH7a not observed.*

^{13}C NMR (126 MHz, $CDCl_3$) δ 170.6 (C19), 134.8 (C13), 134.6 (C4), 123.2 (C12), 117.3 (C3), 82.4 (C6), 77.8 (C9), 75.1 (C10), 73.1 (C7), 37.0 (C8), 33.8 (C5), 29.0 (C11), 21.3 (C20), 20.7 (C14), 14.3 (C15).

$[\alpha]_D^{25}$ = -47.6 (c=0.04, $CHCl_3$).

(2S,3R,5S)-2-Allyl-5-((R,Z)-1-hydroxyhex-3-en-1-yl)tetrahydrofuran-3-yl methanesulfonate (14)

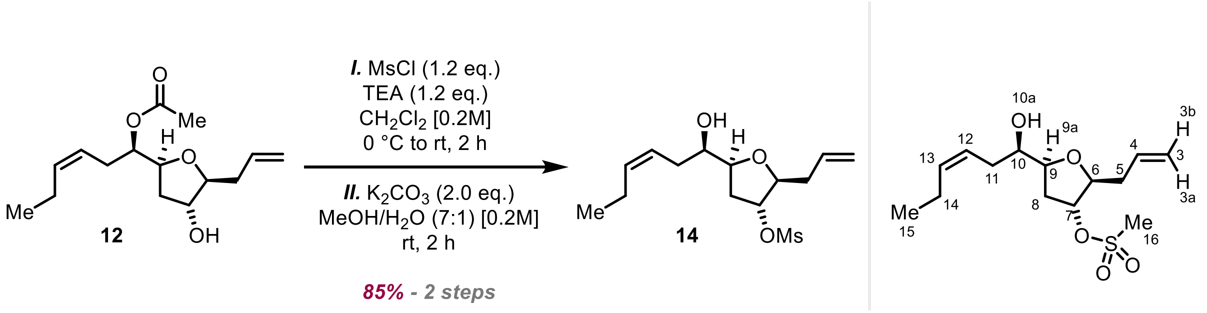

Step 1. Compound 12 (4.3 g, 16 mmol, d.r. > 5:1) was dissolved in dry CH_2Cl_2 (76 mL) and stirred at 0 °C. Triethylamine (2.7 mL, 19 mmol, 1.2 eq.) was added followed by MsCl (1.5 mL, 19 mmol, 1.2 eq.). The reaction mixture was stirred at 0 °C for 20 minutes before being warmed to room temperature and stirred for 2 h. The reaction was then cooled to 0 °C and quenched with sat. aq. $NaHCO_3$ solution (50 mL). H_2O (50 mL) was added, and the organic layer separated. The aqueous layer was extracted with CH_2Cl_2 (3 x 100 mL). The combined organics were washed with brine (300 mL), dried with anhydrous Na_2SO_4, filtered and concentrated under reduced pressure to give a pale-yellow oil that was carried forward to the next step.

Step 2. The crude material from step 1 was dissolved in MeOH/H_2O (7:1, 80 mL) and K_2CO_3 (4.4 g, 32 mmol, 2.0 eq.) was added. The reaction mixture was then stirred at rt for 3 h. After this time the reaction mixture was diluted with H_2O (80 mL) and the organics were extracted with EtOAc (3 × 150 mL). The combined organic layer was dried ($MgSO_4$), filtered and the solvent removed in vacuo to give a crude residue that was further purified by flash column chromatography (15-25% acetone/petroleum ether 40-60) to give desired compound 14 (4.1 g, 14 mmol, 85%) as a pale yellow oil.

R_f = 0.50 (50% EtOAc/petroleum ether 40-60).

IR (ν_{max} cm^{-1}): 3538 (broad), 2964 (medium), 1642 (weak), 1437 (medium), 1355 (strong), 1172 (strong), 1071 (medium), 906 (strong).

HRMS (ESI) m/z : $[M+Na]^+$ calculated for $C_{14}H_{24}NaO_5S$ 327.1237; found 327.1237.

^{1}H NMR (500 MHz, $CDCl_3$) δ 5.80 (ddt, J = 17.2, 10.2, 7.0 Hz, 1H, H4), 5.55 (dtt, J = 10.8, 7.1, 1.4 Hz, 1H, H13), 5.37 (dtt, J = 10.9, 7.4, 1.4 Hz, 1H, H12), 5.21 – 5.12 (m, 2H, H3a, H3b), 4.95 (dt, J = 6.2, 2.1 Hz, 1H, H7), 4.16 (td, J = 6.4, 2.5 Hz, 1H, H6), 4.12 (ddd, J = 10.1, 5.6, 3.5 Hz, 1H, H9a), 3.87 (t, J = 8.2 Hz, 1H, H10), 3.03 (s, 3H, H16), 2.38 – 2.34 (m, 2H, H5), 2.29 – 2.14 (m, 3H, H8, H11), 2.13 – 2.01 (m, 3H, H8', H14), 0.97 (t, J = 7.5 Hz, 3H, H15). *OH10a not observed.*

^{13}C NMR (126 MHz, $CDCl_3$) δ 135.1 (C13), 133.1 (C4), 123.7 (C12), 118.9 (C3), 83.8 (C7), 83.1 (C6), 81.2 (C9), 70.9 (C10), 38.8 (C16), 37.9 (C5), 31.8 (C8), 30.8 (C11), 20.8 (C14), 14.3 (C15).

$[\alpha]_D^{25}$ = -38.0 (c=0.09, $CHCl_3$).

(1*S*,3*S*,4*S*,6*R*)-3-Allyl-6-((*Z*)-pent-2-en-1-yl)-2,5-dioxabicyclo[2.2.1]heptane (15)

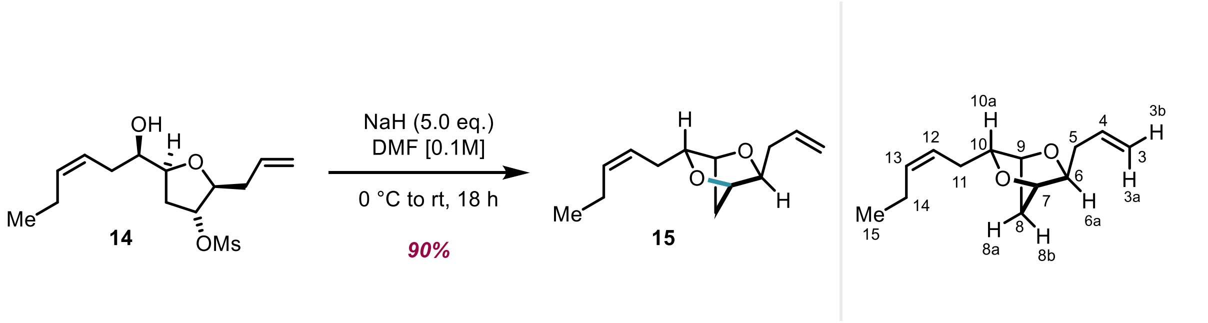

Compound 14 (4.1 g, 14 mmol) was dissolved in dry DMF (140 mL) and stirred at 0 °C. NaH (60% dispersion in mineral oil, 2.8 g, 70 mmol, 5.0 eq.) was then added, with an exit needle in place for 2 minutes to allow for gas evolution. The reaction mixture was then slowly warmed to room temperature and stirred for 18 h. After this time, the reaction mixture was cooled to 0 °C and the excess NaH was quenched by addition of water (300 mL). The organics were extracted with petroleum ether 40-60 (4 x 150 mL). The combined organics were then washed with brine (2 x 200 mL), dried with anhydrous Na_2SO_4, filtered and concentrated under reduced pressure. The crude orange oil was purified by flash column chromatography (5-10% EtOAc/petroleum ether 40-60) to give the desired compound 15 as a pale yellow oil (2.6 g, 13 mmol, 90%). **note: yellow colour likely due to trace DMF related impurity, compound is colourless when isolated from reduction of compounds 16 and 17.*

R_f = 0.36 (10% EtOAc/petroleum ether 40-60).

IR (ν_{max} cm^{-1}): 2963 (medium), 1640 (weak), 1453 (medium), 1367 (strong).

HRMS (ESI) *m/z* : $[M+H]^+$ calculated for $C_{13}H_{21}O_2$ 209.1537; found 209.1537.

^{1}H NMR (500 MHz, $CDCl_3$) δ 5.80 (ddt, *J* = 17.2, 10.2, 7.0 Hz, 1H, H4), 5.49 (dtt, *J* = 10.8, 7.6, 1.6 Hz, 1H, H13), 5.29 (dtt, *J* = 10.2, 7.7, 1.4 Hz, 1H, H12), 5.14 (dq, *J* = 17.2, 1.6 Hz, 1H, H3a), 5.07 (ddt, *J* = 10.2, 2.0, 1.1 Hz, 1H, H3b), 4.35 (d, *J* = 2.6 Hz, 1H, H7), 4.24 (d, *J* = 2.3 Hz, 1H, H9), 3.88 – 3.80 (m, 2H, H6a, H10a), 2.45 – 2.29 (m, 2H, H5), 2.17 – 2.08 (m, 1H, H11), 2.07 – 1.96 (m, 3H, H11', H14), 1.93 (dd, *J* = 10.2, 2.6 Hz, 1H, H8a*), 1.86 (dd, *J* = 10.2, 2.4 Hz, 1H, H8b*), 0.96 (t, *J* = 7.5 Hz, 3H, H15). *H8a/H8b were tentatively assigned from NOESY data. On the basis of these tentative assignments and on comparison with NMR data from previously reported compounds,[5] the structure is consistent with a 6,10-*anti*-dioxabicycloheptane. Ultimately, the 6,10-*anti*-dioxabicycloheptane structure is confirmed from the single crystal X-ray structure of 18.

^{13}C NMR (126 MHz, $CDCl_3$) δ 134.6 (C4), 134.5 (C13), 123.2 (C12), 117.4 (C3), 84.2 (C10), 82.3 (C6), 79.4 (C9), 77.1 (C7), 35.4 (C5), 34.7 (C8), 32.1 (C11), 20.9 (C14), 14.3 (C15).

$[\alpha]_D^{25}$ = +134.2 (c=0.12, $CHCl_3$).

3.2. Bromination, aldehyde degradation and determination of stereochemistry

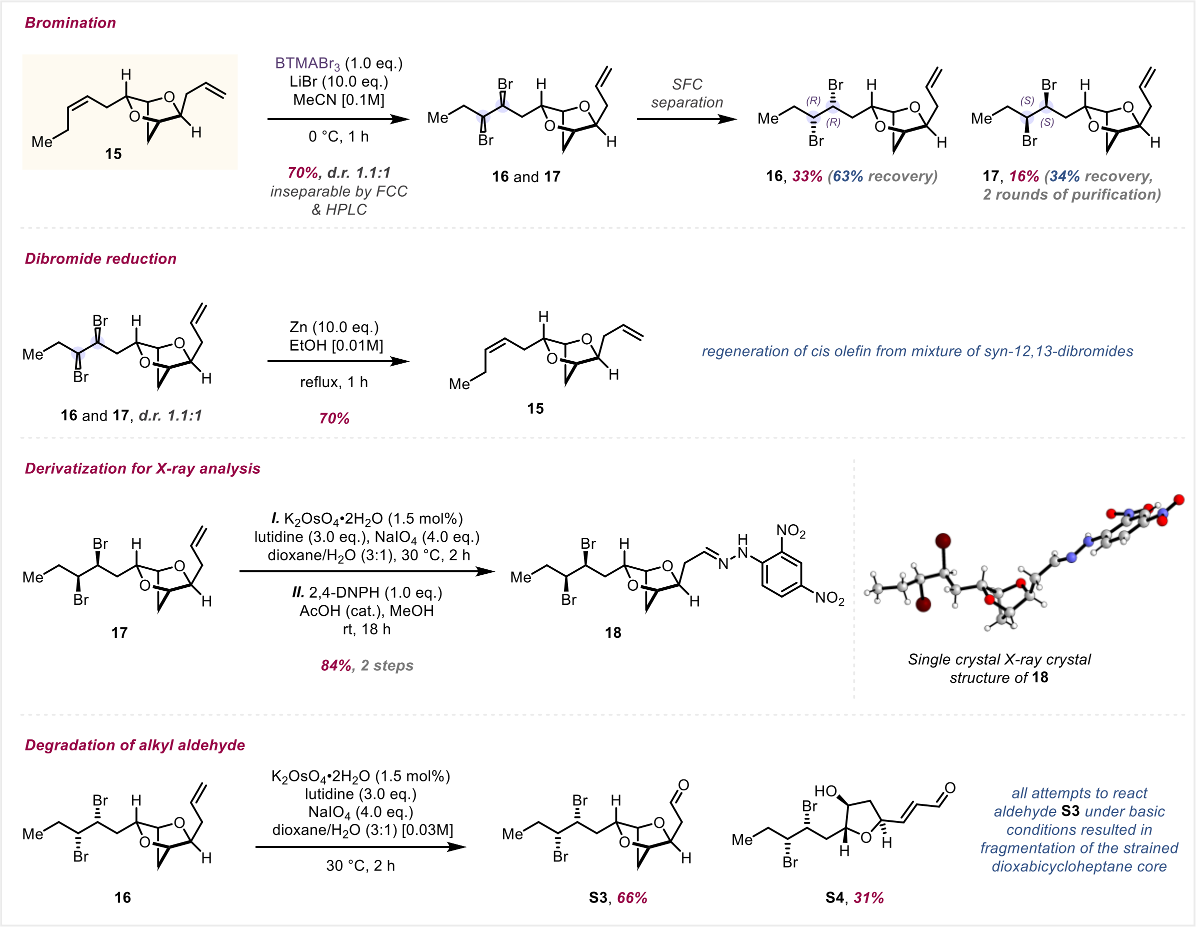

Scheme S2. Diene bromination and experiments to determine the stereochemistry of *syn*-dibromide. Attempts to crystallise derivatives of 16 were unsuccessful.

(1*S*,3*S*,4*S*,6*R*)-3-Allyl-6-((2*R*,3*R*)-2,3-dibromopentyl)-2,5-dioxabicyclo[2.2.1]heptane (16) and

(1*S*,3*S*,4*S*,6*R*)-3-allyl-6-((2*S*,3*S*)-2,3-dibromopentyl)-2,5-dioxabicyclo[2.2.1]heptane (17)

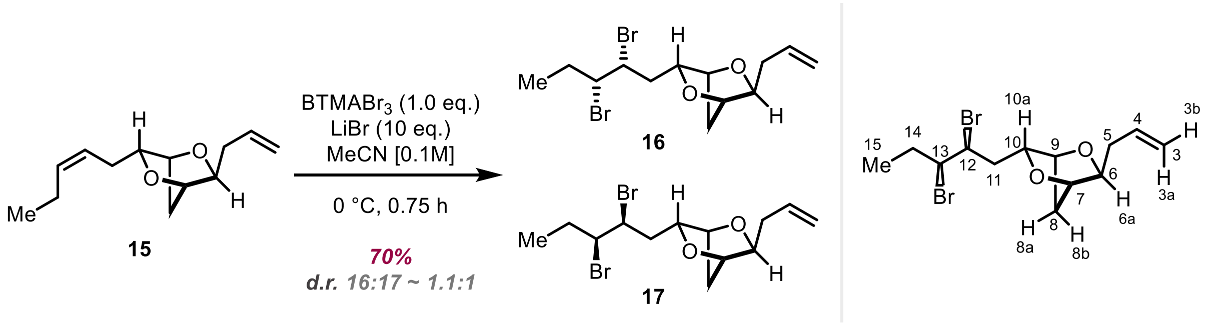

Compound 15 (200 mg, 0.96 mmol) and lithium bromide (834 mg, 9.60 mmol, 10.0 eq.) were dissolved in acetonitrile (9.6 mL) and stirred at room temperature. The solution was cooled to 0 °C and benzyltrimethylammonium tribromide (374 mg, 0.96 mmol, 1.0 eq.) was added and the reaction mixture was stirred for 45 minutes. *At this point the initial bright orange colour of the solution had decolourised indicating full consumption of bromine.* TLC analysis (dichloromethane) at this stage indicated the completion of reaction. The reaction mixture was warmed to room temperature and dry loaded onto Celite® before purification by flash column chromatography (100 g silica, CV = 200 mL, 50-100% dichloromethane/petroleum ether 40-60) to yield inseparable diastereomers 16 and 17 as a clear oil (247 mg, 0.67 mmol, 70%, d.r. (16:17) 1.1:1).

SFC Purification

The isomers were separated by chiral SFC using a Chiralpak IG (250 x 20 mm), 5 µm (Daicel, Chiral Technologies Europe) column at 40 °C using SFC Prep150 Instrument (Waters Corp.). Purification was achieved using a gradient method where the mobile phase 15% MeOH, 85% CO_2 to 15% MeOH, 85% CO_2 over 5.3 min, to 30% MeOH, 70% CO_2 over 1.0 min, 30% MeOH 70% CO_2 for 2.4 min, to 15% MeOH (20 mM NH_3) 85% CO_2 over 0.3 min, at a flow rate of 100 mL/min. Concentration of the sample was ~26.7 mg/mL in CH_2Cl_2 : MeOH 1:1 (no mod.). Injection volume was 1000 µL. Outlet pressure was 120 bar. Detection wavelength was 210.5 nm. RT of Peak 1 (16) was 3.58 min and Peak 2 (17) was 5.53 min. Yield returned of 16: (81 mg. 33% (63% recovery)). Yield returned of 17: (70 mg. 28% (60% recovery)). At this stage 17 still contained a 10-15% isomeric impurity so was subjected to a further round of SFC purification.

Compound 17 was further purified by chiral SFC using a Chiralpal IG-3 (50 x 3 mm), 3 µm (Daicel, Chiral Technologies Europe) column at 40 °C using a UPCC instrument (Waters Corp.). Resolution was achieved using a gradient method where the mobile phase was 5% MeOH (20 mM NH_3), 95% CO_2 to 50% MeOH (20 mM NH_3), 50% CO_2 over 3.5 min at a flow rate of 2 mL/min. Concentration of the sample was ~ 1 mg/mL in MeOH (no mod.). Injection volume was 2 µL. Outlet pressure was 140 bar. Detection wavelength was 210.5 nm. Retention

time of Peak 1 (17) was 1.11 minutes and Peak 2 was 1.46 minutes. Yield returned of 17: (40 mg. 16% (34% recovery)).

Characterisation of 16

R_f = 0.55 (dichloromethane).

IR (ν_{max} cm^{-1}): 2970 (medium), 2920 (medium), 2849 (weak), 1641 (weak), 1454 (weak), 1381 (weak) 1083 (strong), 1056 (strong), 945 (strong), 921 (weak), 669 (medium).

HRMS (ESI) *m/z* : $[M+H]^+$ calculated for $C_{13}H_{21}Br_2O_2$ 366.9903, 368.9883, 370.9862; found 366.9902, 368.9881, 370.9861.

^{1}H NMR (600 MHz, $CDCl_3$) δ 5.81 (ddt, *J* = 17.2, 10.2, 6.9 Hz, 1H, H4), 5.16 (dt, *J* = 17.3, 1.7 Hz, 1H, H3a), 5.09 (ddd, *J* = 10.3, 2.1, 1.1 Hz, 1H, H3b), 4.42 (dt, *J* = 10.7, 2.5 Hz, 1H, H12), 4.36 (d, *J* = 2.6 Hz, 1H, H7), 4.32 (d, *J* = 2.3 Hz, 1H, H9), 4.10 (dt, *J* = 10.1, 3.1 Hz, 1H, H13), 4.07 (dd, *J* = 9.8, 3.3 Hz, 1H, H10a), 3.86 (t, *J* = 7.1 Hz, 1H, H6a), 2.42 (dt, *J* = 14.1, 7.0 Hz, 1H, H5), 2.36 (dt, *J* = 14.0, 7.0 Hz, 1H, H5'), 2.09 (dqd, *J* = 14.5, 7.2, 3.6 Hz, 1H, H14), 1.97 – 1.93 (m, 1H, H11), 1.94 – 1.90 (m, 1H, H8b), 1.86 (dd, *J* = 10.5, 2.7 Hz, 1H, H8a), 1.84 – 1.81 (m, 1H, H14'), 1.81 – 1.77 (m, 1H, H11'), 1.07 (t, *J* = 7.3 Hz, 3H, H15).

^{13}C NMR (151 MHz, $CDCl_3$) δ 134.5 (C4), 117.5 (C3), 82.4 (C6), 81.7 (C10), 80.0 (C9), 77.3 (C7), 62.2 (C13), 54.6 (C12), 40.0 (C11), 35.4 (C8), 35.3 (C5), 29.1 (C14), 12.7 (C15).

$[\alpha]_D^{25}$ = +145.8 (c=0.50, $CHCl_3$).

Characterisation of 17

R_f = 0.55 (dichloromethane).

IR (ν_{max} cm^{-1}): 3003 (medium), 2968 (medium), 2913 (medium), 2877 (weak), 1642 (weak), 1457 (medium), 1436 (medium), 1220 (weak), 1196 (weak), 1081 (strong), 1052 (strong), 950 (strong), 920 (strong), 804 (weak), 774 (medium).

HRMS (APCI) *m/z* : $[M+H]^+$ calculated for $C_{13}H_{21}Br_2O_2$ 366.9903, 368.9883, 370.9862; found 366.9906, 368.9885, 370.9862.

^{1}H NMR (600 MHz, $CDCl_3$) δ 5.80 (ddt, *J* = 17.1, 10.1, 6.9 Hz, 1H, H4), 5.16 (dq, *J* = 17.1, 1.7 Hz, 1H, H3a), 5.09 (dd, *J* = 10.3, 1.4 Hz, 1H, H3b), 4.35 (s, 1H, H7), 4.28 (d, *J* = 2.2 Hz, 1H, H9), 4.22 (ddd, *J* = 8.3, 5.9, 2.6 Hz, 1H, H12), 4.12 (dt, *J* = 9.8, 3.2 Hz, 1H, H13), 4.00 (t, *J* = 7.0 Hz, 1H, H10a), 3.86 (td, *J* = 7.1, 1.1 Hz, 1H, H6a), 2.41 (dt, *J* = 14.0, 7.0 Hz, 1H, H5), 2.34 (dt, *J* = 14.1, 7.1 Hz, 1H, H5'), 2.15 (dt, *J* = 14.5, 6.4 Hz, 1H, H11), 2.07 (dqd, *J* = 14.4, 7.2, 3.7 Hz, 1H, H14), 1.94 – 1.84 (m, 4H, H8a, H8b, H11', H14'), 1.08 (t, *J* = 7.2 Hz, 3H, H15).

^{13}C NMR (151 MHz, $CDCl_3$) δ 134.4 (C4), 117.6 (C3), 82.4 (C6), 81.8 (C10), 79.3 (C9), 77.1* (C7), 60.7 (C13), 53.5 (C12), 40.2 (C11), 35.3 (C5), 35.0 (C8), 29.4 (C14), 12.7 (C15). **Underneath $CDCl_3$ peak. Shift located and assigned using HSQC and HMBC data.*

$[\alpha]_D^{25}$ = +13.1 (c=0.32, $CHCl_3$).

(1*S*,3*S*,4*S*,6*R*)-3-Allyl-6-((*Z*)-pent-2-en-1-yl)-2,5-dioxabicyclo[2.2.1]heptane (15)

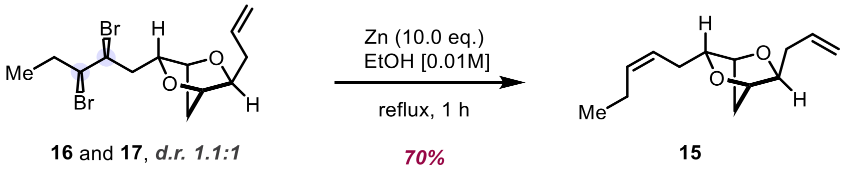

**The Zn powder used in this experiment was activated by washing with 2% HCl (aq.) for 1 minute, before being washed with H_2O, EtOH and Et_2O and dried under high vacuum.*[4]

Compounds 16 and 17 (as an inseparable 1.1:1 mixture) (7 mg, 19 µmol) were dissolved in EtOH (1.5 mL) and added to a rbf containing activated Zn powder (12.4 mg, 190 µmol, 10 eq.). The reaction mixture was stirred and heated to reflux for 1 h. TLC analysis (dichloromethane) at this stage indicated the completion of reaction. The reaction mixture was cooled to rt, filtered through a small plug of Celite® and concentrated. The resultant clear oil was then purified by flash column chromatography (0.5 g silica, CV = 1 mL, 0-5% diethyl ether/dichloromethane) to yield 15 as a clear oil (2.8 mg, 13 µmol, 70%).

Spectroscopic and physical data matched that of 15 isolated via intramolecular S_N2 of 14 (see page S13).

(*E*)-1-(2-((1*S*,3*S*,4*S*,6*R*)-6-((2*S*,3*S*)-2,3-Dibromopentyl)-2,5-dioxabicyclo[2.2.1]heptan-3-yl)ethylidene)-2-(2,4-dinitrophenyl)hydrazine (18)

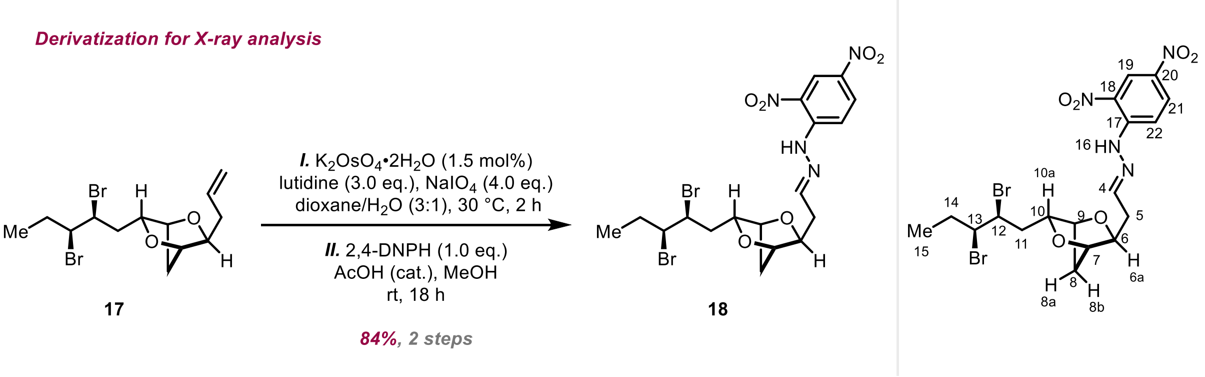

Step 1.[11] Compound 17 (3.9 mg, 11 µmol) was dissolved in 3:1 dioxane/H_2O (0.3 mL) and stirred at rt. Lutidine (3.7 µL, 32 µmol, 3.0 eq.) was added, followed by K_2OsO_4•$2H_2O$ (60 µg, 0.2 µmol, 1.5 mol%) and $NaIO_4$ (9.1 mg, 42 µmol, 4.0 eq.). The reaction mixture was then heated to 30 °C for 2 h. TLC analysis (2.5% diethyl ether/dichloromethane) at this stage indicated the completion of reaction. The reaction mixture was cooled to rt and quenched with sat. aq. $Na_2S_2O_3$ solution (4 mL). H_2O (1 mL) was added and the organics were extracted with EtOAc (3 x 7 mL). The combined organics were washed with brine (8 mL), dried (Na_2SO_4), filtered and concentrated to give a clear oil that was immediately carried forward to the next step.

Step 2. The residue from step 1 was dissolved in MeOH (0.3 mL) and 2,4-dinitrophenylhydrazine (2,4-DNPH) (2.2 mg, 11 µmol, 1.0 eq.) was added to the reaction followed by acetic acid (1 drop). The reaction mixture was then stirred at rt for 18 h. TLC analysis (dichloromethane + 0.5% triethylamine) at this stage indicated the completion of reaction. The reaction mixture was loaded onto Celite® and purified by flash column chromatography (5 g silica, CV = 10 mL, 80% dichloromethane/pentane + 0.5% triethylamine, then 0-2% diethyl ether/dichloromethane + 0.5% triethylamine) to yield 18 as an amorphous yellow solid (4.9 mg, 9 µmol, 84%). Single crystals of 18 were grown by dissolution of 18 in a minimal volume of hot diethyl ether, followed by incubation in a fridge at 3 °C.

R_f = 0.27 (80% dichloromethane/pentane + 0.5% triethylamine).

IR (ν_{max} cm^{-1}): 3300 (weak), 2970 (medium), 1618 (strong), 1593 (medium), 1519 (medium), 1424 (medium), 1334 (strong), 1222 (weak), 1139 (weak), 1074 (medium), 922 (weak), 834 (weak), 762 (medium).

HRMS (ESI) *m/z* : $[M-H]^-$ calculated for $C_{18}H_{21}Br_2N_4O_6$ 546.9833, 548.9812, 550.9792; found 546.9838, 548.9815, 550.9795.

**note that compound 18 exists in solution as a 3:1 mixture of geometric isomers. Only ^1H and ^{13}C NMR data for the major geometric isomer are reported.*

^1H NMR (600 MHz, $CDCl_3$) δ 11.07 (s, 1H, H16), 9.12 (d, *J* = 2.5 Hz, 1H, H19), 8.31 (dd, *J* = 9.6, 2.2 Hz, 1H, H21), 7.91 (d, *J* = 9.5 Hz, 1H, H22), 7.61 (t, *J* = 5.2 Hz, 1H, H4), 4.49 (d, *J* = 2.4 Hz, 1H, H7), 4.35 (d, *J* = 2.1 Hz, 1H, H9), 4.24 – 4.20 (m, 1H, H12), 4.20 – 4.16

(m, 1H, H6a), 4.16 – 4.11 (m, 1H, H13), 4.06 (t, J = 6.9 Hz, 1H, H10a), 2.80 (ddd, J = 15.7, 7.9, 5.1 Hz, 1H, H5), 2.71 (dt, J = 15.6, 5.6 Hz, 1H, H5'), 2.17 (ddd, J = 14.6, 7.0, 5.6 Hz, 1H, H11), 2.09 (ddtd, J = 14.5, 10.9, 7.3, 3.7 Hz, 1H, H14), 2.03 – 1.97 (m, 2H, H8a, H8b), 1.97 – 1.92 (m, 1H, H11), 1.92 – 1.82 (m, 1H, H14), 1.09 (t, J = 7.2 Hz, 3H, H15).

^{13}C NMR (151 MHz, $CDCl_3$) δ 149.3 (C4), 145.1 (C17), 138.2 (C20), 130.1 (C21), 129.2 (C18), 123.6 (C19), 116.7 (C22), 82.1 (C10), 80.2 (C6), 79.4 (C9), 77.5 (C7), 60.6 (C13), 53.3 (C12), 39.9 (C11), 35.0 (C8), 34.3 (C5), 29.1 (C14), 12.7 (C15).

$[\alpha]_D^{25}$ = -2.0 (c=0.49, $CHCl_3$).

Melting point: 116-118 °C (diethyl ether).

Single Crystal Data for 18: $C_{18}H_{22}Br_2N_2O_6$, Mr =550.21. 150 K – monoclinic, P 2_1, a = 13.9502(3) Å, b = 17.7058(3) Å, c = 19.3618(5) Å, β = 94.387(2)°, V = 823.23(3) Å, Data/restraints/parameters – 18008/1/1082, Flack = 0.021(10) for 6851 Friedel pairs, Rint = 0.087, Final R1 = 0.0664, wR2 = 0.1717 (I>2σ(I)).

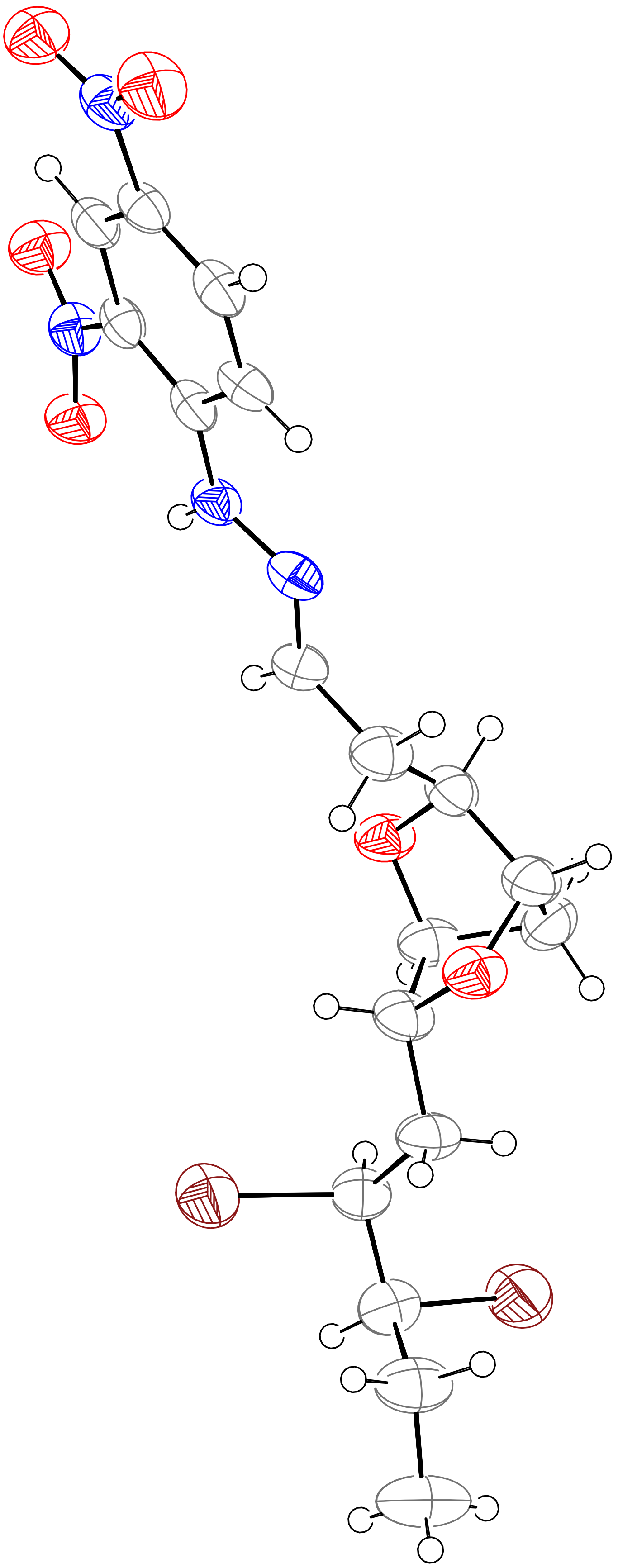

Figure S2: Single crystal X-ray crystal structure of compound 18 with ADPs shown at 50% probability.

2-((1S,3S,4S,6R)-6-((2R,3R)-2,3-Dibromopentyl)-2,5-dioxabicyclo[2.2.1]heptan-3-yl)acetaldehyde (S3) and

(E)-3-((2S,4S,5R)-5-((2R,3R)-2,3-dibromopentyl)-4-hydroxytetrahydrofuran-2-yl)acrylaldehyde (S4)

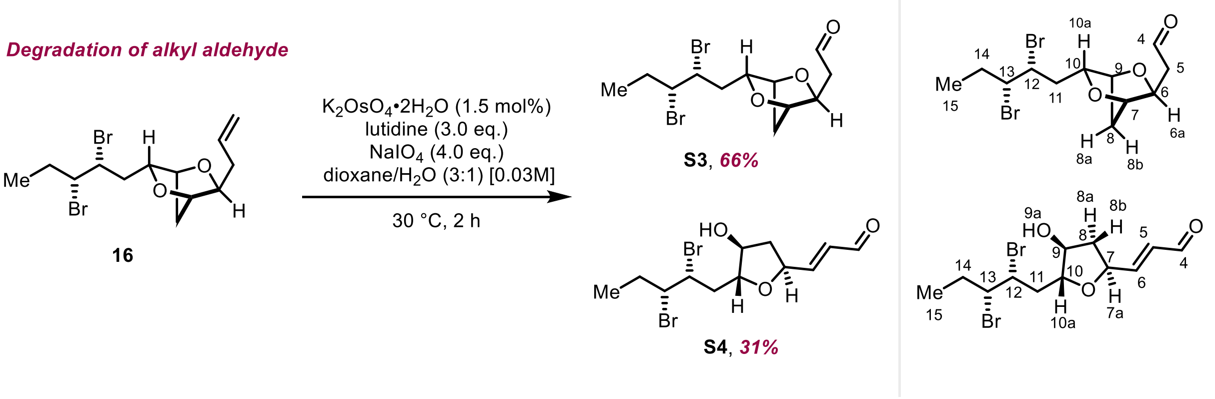

Compound 16 (6.0 mg, 16 µmol) was dissolved in 3:1 dioxane/H_2O (0.5 mL) and stirred at rt. Lutidine (5.7 µL, 49 µmol, 3.0 eq.) was added, followed by K_2OsO_4•$2H_2O$ (90 µg, 0.2 µmol, 1.5 mol%) and $NaIO_4$ (13.9 mg, 65 µmol, 4.0 eq.). The reaction mixture was then heated to 30 °C for 2 h. TLC analysis (2.5% diethyl ether/dichloromethane) at this stage indicated the completion of reaction. The reaction mixture was cooled to rt and quenched with sat. aq. $Na_2S_2O_3$ solution (4 mL). H_2O (1 mL) was added and the organics were extracted with EtOAc (3 x 7 mL). The combined organics were washed with brine (8 mL), dried (Na_2SO_4), filtered and concentrated. *Note that at this stage the degradation product S4 is only present in <10%, most degradation occurs on silica.* The resultant clear oil was then purified by flash column chromatography (2.5 g silica, CV = 5 mL, 0-10% MeOH/dichloromethane) to yield desired compound S3 as a clear oil (4.0 mg, 11 µmol, 66%) and side product S4 as a clear oil (1.9 mg, 5 µmol, 31%, $E/Z > 20:1$). *All attempts to react aldehyde S3 under basic reaction conditions resulted in degradation via the elimination pathway.*

Characterisation of S3

R_f = 0.63 (10% diethyl ether/dichloromethane).

IR (ν_{max} cm^{-1}): 2968 (medium), 2917 (medium), 1721 (strong), 1457 (medium), 1378 (medium) 1070 (strong), 1033 (medium), 945 (strong), 925 (weak), 825 (medium), 766 (strong).

HRMS (ESI) m/z: $[M+Na]^+$ calculated for $C_{12}H_{18}Br_2O_3Na$ 390.9515, 392.9495, 394.9474; found 390.9514, 392.9495, 394.9475.

^1H NMR (600 MHz, $CDCl_3$) δ 9.84 (t, J = 1.1 Hz, 1H, H4), 4.46 (d, J = 2.5 Hz, 1H, H7), 4.40 (dt, J = 10.9, 2.5 Hz, 1H, H12), 4.34 (d, J = 2.4 Hz, 1H, H9), 4.31 (t, J = 6.6 Hz, 1H, H6a), 4.09 (dt, J = 10.0, 3.2 Hz, 1H, H13), 4.06 (dd, J = 10.0, 3.2 Hz, 1H, H10a), 2.89 (ddd, J = 17.8, 6.8, 1.4 Hz, 1H, H5), 2.78 (ddd, J = 17.8, 6.3, 1.1 Hz, 1H, H5'), 2.09 (dqd, J = 14.5, 7.3, 3.7 Hz, 1H, H14), 1.98 (dd, J = 10.5, 2.5 Hz, 1H, H8b), 1.94 (ddd, J = 14.3, 10.9, 3.3 Hz, 1H, H11), 1.90 (dd, J = 10.5, 2.7 Hz, 1H, H8a), 1.86 – 1.82 (m, 1H, H14'), 1.78 (ddd, J = 14.6, 9.9, 2.3 Hz, 1H, H11'), 1.07 (t, J = 7.2 Hz, 3H, H15).

^{13}C NMR (151 MHz, $CDCl_3$) δ 200.6 (C4), 81.8 (C10), 79.9 (C9), 77.6 (C7), 77.4 (C6), 62.0 (C13), 54.5 (C12), 45.3 (C5), 39.8 (C11), 35.2 (C8), 29.1 (C14), 12.7 (C15).

$[\alpha]_D^{25}$ = +129.8 (c=0.40, $CHCl_3$).

Characterisation of side product S4

R_f = 0.20 (10% diethyl ether/dichloromethane).

IR (ν_{max} cm^{-1}): 3437 (broad), 2918 (medium), 2851 (medium), 1686 (strong), 1457 (weak), 1339 (weak), 1219 (weak), 1107 (medium), 1057 (medium), 980 (medium), 768 (strong).

HRMS (ESI) *m/z* : $[M-H]^-$ calculated for ($C_{12}H_{17}Br_2O_3$) 366.9549, 368.9529, 370.9509; found 366.9546, 368.9531, 370.9505.

^1H NMR (600 MHz, $CDCl_3$) δ 9.60 (d, *J* = 7.8 Hz, 1H, H4), 6.89 (dd, *J* = 15.7, 5.2 Hz, 1H, H6), 6.31 (ddd, *J* = 15.7, 7.8, 1.5 Hz, 1H, H5), 4.83 – 4.76 (m, 1H, H7a), 4.50 (dt, *J* = 10.7, 2.6 Hz, 1H, H12), 4.25 – 4.22 (m, 1H, H9), 4.22 – 4.19 (m, 1H, H10a), 4.17 (dt, *J* = 10.1, 3.1 Hz, 1H, H13), 2.57 (ddd, *J* = 14.2, 8.4, 6.1 Hz, 1H, H8a*), 2.18 – 2.10 (m, 2H, H11, H14), 2.04 (ddd, *J* = 14.6, 10.0, 2.5 Hz, 1H, H11'), 1.92 (ddd, *J* = 13.4, 5.8, 4.3 Hz, 1H, H8b*), 1.85 (dtd, *J* = 14.6, 7.3, 2.8 Hz, 1H, H14'), 1.75 (d, *J* = 4.8 Hz, 1H, OH9a), 1.09 (t, *J* = 7.3 Hz, 3H, H15).*H8a/H8b were tentatively assigned from NOESY data.

^{13}C NMR (151 MHz, $CDCl_3$) δ 193.5 (C4), 156.8 (C6), 130.8 (C5), 83.7 (C10), 76.4* (C7), 76.4* (C9), 62.0 (C13), 55.4 (C12), 40.4 (C8), 39.2 (C11), 28.9 (C14), 12.8 (C15). **Resonances too close together to unambiguously assign.*

$[\alpha]_D^{25}$ = +92.1 (c=0.19, $CHCl_3$).

3.3. Enyne syntheses to generate (*E*)- and (*Z*)-ocellenynes (8) and the enantiomers of the Suzuki (*E*)- and (*Z*)-ocellenynes (5)

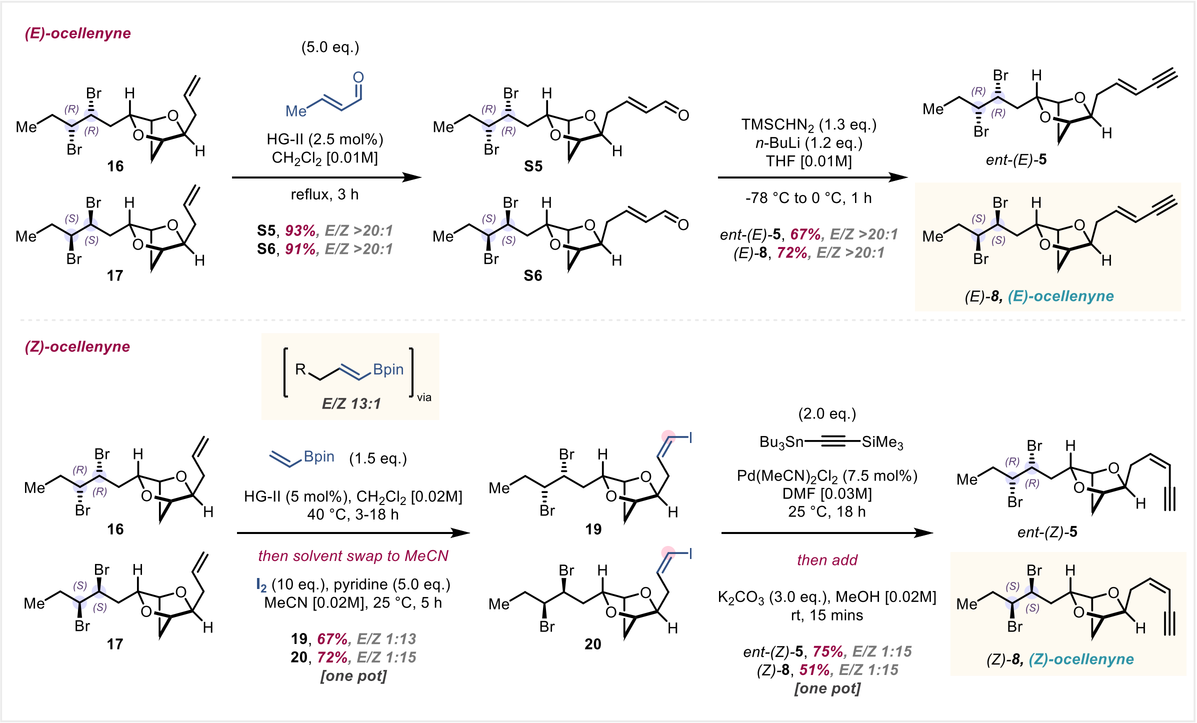

Scheme S3. Enyne syntheses to generate (*E*)- and (*Z*)-ocellenynes (8) and the enantiomers of the Suzuki (*E*)- and (*Z*)-ocellenynes (5).

(*E*)-4-((1*S*,3*S*,4*S*,6*R*)-6-((2*R*,3*R*)-2,3-Dibromopentyl)-2,5-dioxabicyclo[2.2.1]heptan-3-yl)but-2-enal (S5)

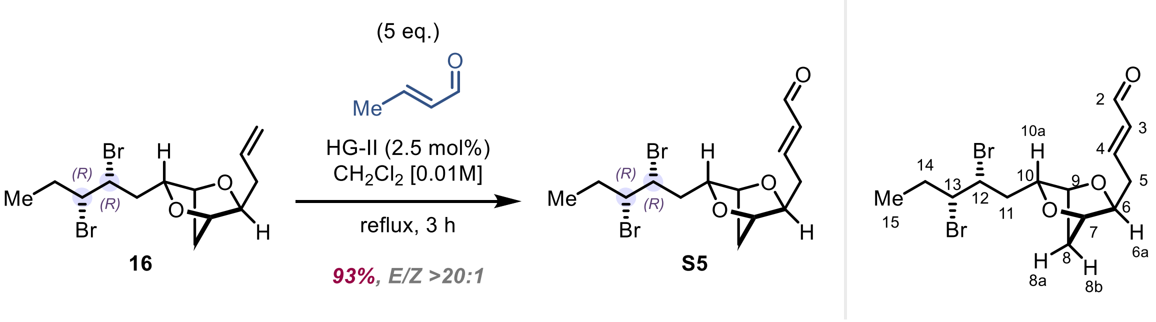

*All solvents used in this reaction were sparged with argon for 15 minutes before use. Compound 16 (5.0 mg, 14 µmol) and crotonaldehyde (5.6 µL, 68 µmol, 5 eq.) were dissolved in dry CH_2Cl_2 (0.75 mL) and stirred at rt. Hoveyda-Grubb's 2nd generation catalyst (0.2 mg, 0.3 µmol, 2.5 mol%) was dissolved in dry CH_2Cl_2 (0.75 mL) and added to the reaction mixture. The reaction mixture was then heated to reflux for 3 h. TLC analysis (5% diethyl ether/dichloromethane) at this stage indicated completion of reaction. The reaction mixture was cooled to rt and DMSO (1 drop) was added before all volatiles were removed under reduced pressure.[12] The resulting deep green residue was purified by flash column chromatography (2.5 g silica, CV = 5 mL, 0-7.5% diethyl ether/dichloromethane) to give the desired compound S5 (5.0 mg, 13 µmol, 93%, $E/Z >$ 20:1) as a clear oil.

R_f = 0.38 (5% diethyl ether/dichloromethane).

IR (ν_{max} cm^{-1}): 2970 (medium), 1687 (strong), 1457 (weak), 1381 (weak), 1136 (weak), 1068 (medium), 944 (medium), 823 (weak).

HRMS (APCI) *m/z* : $[M+H]^+$ calculated for $C_{14}H_{21}Br_2O_3$ 394.9852, 396.9832, 398.9812; found 394.9846, 396.9826, 398.9806.

^{1}H NMR (600 MHz, $CDCl_3$) δ 9.53 (d, *J* = 7.8 Hz, 1H, H2), 6.88 (dt, *J* = 15.7, 7.0 Hz, 1H, H4), 6.23 (ddt, *J* = 15.6, 7.8, 1.5 Hz, 1H, H3), 4.42 (dt, *J* = 10.9, 2.5 Hz, 1H, H12), 4.39 (d, *J* = 2.5 Hz, 1H, H7), 4.36 (d, *J* = 2.3 Hz, 1H, H9), 4.13 – 4.08 (m, 2H, H10a, H13), 3.98 (ddd, *J* = 7.4, 5.9, 1.1 Hz, 1H, H6a), 2.71 (dddd, *J* = 14.7, 8.0, 6.6, 1.6 Hz, 1H, H5), 2.61 (dddd, *J* = 14.9, 7.4, 5.8, 1.4 Hz, 1H, H5'), 2.10 (dqd, *J* = 14.4, 7.2, 3.7 Hz, 1H, H14), 1.98 – 1.89 (m, 3H, H8a, H8b, H11), 1.83 (m, 1H, H14'), 1.80 (ddd, *J* = 14.4, 8.7, 2.7 Hz, 1H, H11'), 1.08 (t, *J* = 7.2 Hz, 3H, H15).

^{13}C NMR (151 MHz, $CDCl_3$) δ 193.9 (C2), 154.2 (C4), 135.0 (C3), 81.8 (C10), 81.1 (C6), 80.1 (C9), 77.4 (C7), 62.0 (C13), 54.4 (C12), 39.8 (C11), 35.4 (C8), 34.5 (C5), 29.0 (C14), 12.7 (C15).

$[\alpha]_D^{25}$ = +111.9 (c=0.50, $CHCl_3$).

(E)-4-((1S,3S,4S,6R)-6-((2S,3S)-2,3-Dibromopentyl)-2,5-dioxabicyclo[2.2.1]heptan-3-yl)but-2-enal (S6)

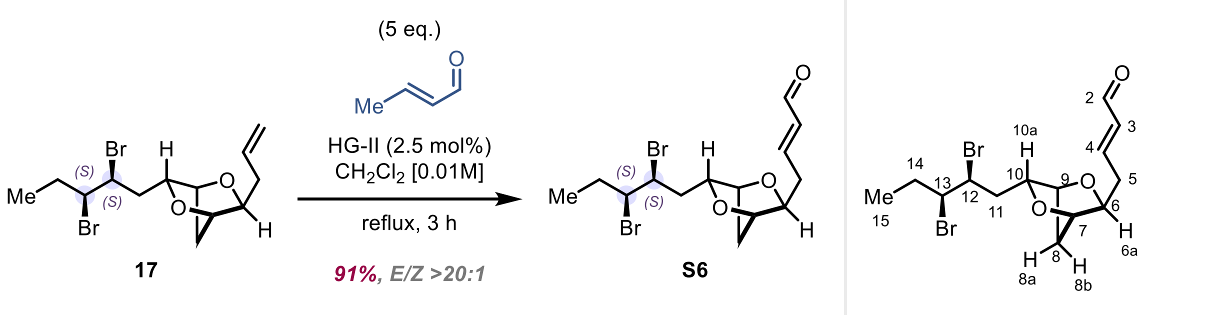

*All solvents used in this reaction were sparged with argon for 15 minutes before use. Compound 17 (5.0 mg, 14 µmol) and crotonaldehyde (5.6 µL, 68 µmol, 5 eq.) were dissolved in dry CH_2Cl_2 (0.75 mL) and stirred at rt. Hoveyda-Grubb's 2nd generation catalyst (0.2 mg, 0.3 µmol, 2.5 mol%) was dissolved in dry CH_2Cl_2 (0.75 mL) and added to the reaction mixture. The reaction mixture was then heated to reflux for 3 h. TLC analysis (5% diethyl ether/dichloromethane) at this stage indicated completion of reaction. The reaction mixture was cooled to rt and DMSO (1 drop) was added before all volatiles were removed under reduced pressure.[12] The resulting deep green residue was purified by flash column chromatography (2.5 g silica, CV = 5 mL, 0-7.5% diethyl ether/dichloromethane) to give the desired compound S6 (4.9 mg, 12 µmol, 91%, $E/Z >$ 20:1) as a clear oil.

R_f = 0.38 (5% diethyl ether/dichloromethane).

IR (ν_{max} cm^{-1}): 2922 (weak), 1686 (strong), 1457 (weak), 1073 (medium), 951 (medium), 763 (weak).

HRMS (APCI) m/z : $[M+H]^+$ calculated for $C_{14}H_{21}Br_2O_3$ 394.9852, 396.9832, 398.9812; found 394.9852, 396.9831, 398.9811.

^{1}H NMR (600 MHz, $CDCl_3$) δ 9.52 (d, J = 7.8 Hz, 1H, H2), 6.87 (dt, J = 15.7, 7.0 Hz, 1H, H4), 6.22 (ddt, J = 15.7, 7.8, 1.5 Hz, 1H, H3), 4.38 (d, J = 2.0 Hz, 1H, H7), 4.33 (d, J = 1.9 Hz, 1H, H9), 4.21 (ddd, J = 8.4, 5.7, 2.6 Hz, 1H, H12), 4.12 (dt, J = 9.8, 3.2 Hz, 1H, H13), 4.02 (t, J = 7.0 Hz, 1H, H10a), 3.98 (ddd, J = 7.5, 5.9, 1.1 Hz, 1H, H6a), 2.70 (dddd, J = 14.7, 7.9, 6.4, 1.6 Hz, 1H, H5), 2.59 (dddd, J = 14.9, 7.4, 5.8, 1.4 Hz, 1H, H5'), 2.16 (ddd, J = 14.7, 7.0, 5.7 Hz, 1H, H11), 2.08 (dqd, J = 14.5, 7.2, 3.7 Hz, 1H, H14), 1.95 (s, 2H, H8a, H8b), 1.94 – 1.89 (m, 1H, H11'), 1.89 – 1.84 (m, 1H, H14'), 1.09 (t, J = 7.2 Hz, 3H, H15).

^{13}C NMR (151 MHz, $CDCl_3$) δ 193.8 (C2), 154.0 (C4), 135.0 (C3), 82.0 (C10), 81.1 (C6), 79.4 (C9), 77.3 (C7), 60.6 (C13), 53.3 (C12), 40.0 (C11), 35.0 (C8), 34.5 (C5), 29.2 (C14), 12.7 (C15).

$[\alpha]_D^{25}$ = -8.3 (c=0.48, $CHCl_3$).

(1*S*,3*R*,4*S*,6*S*)-3-((2*R*,3*R*)-2,3-Dibromopentyl)-6-((*E*)-pent-2-en-4-yn-1-yl)-2,5-dioxabicyclo[2.2.1]heptane (*ent-(E)*-5)

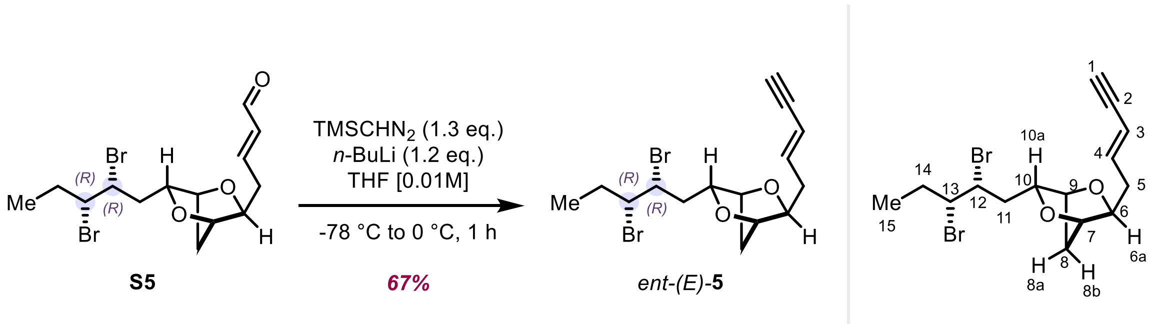

**on small scale it is preferable to prepare a stock solution of lithiated-(trimethylsilyl)diazomethane and transfer a portion of this to the reaction mixture.*

(Trimethylsilyl)diazomethane solution (2.0M in hexanes, 82 µL, 164 µmol, 13 eq.) was added to a flame dried Schlenk tube through a suba seal. Dry THF (1.82 mL) was added and the solution was cooled to -78 °C whilst stirring. *n*-Butyllithium solution (1.6M in hexanes, 95 µL, 151 µmol, 12 eq.) was added dropwise and the resultant orange solution was stirred for 30 minutes at -78 °C. In a separate vessel, compound S5 (5.0 mg, 13 µmol) was dissolved in dry THF (1.0 mL) and cooled to -78 °C whilst stirring. A portion of lithiated-(trimethylsilyl)diazomethane solution (0.2 mL, 15 µmol, 1.2 eq.) was quickly transferred via syringe to the solution of starting material S5. The reaction mixture was stirred for 1 h at -78 °C. The reaction mixture was then warmed to 0 °C for 10 minutes before being quenched with sat. aq. NH_4Cl solution (2 mL) and AcOH (4 drops). H_2O (2 mL) and CH_2Cl_2 (8 mL) were added, and the organic layer was then separated. The aqueous layer was extracted with CH_2Cl_2 (2 x 8 mL). The combined organics were washed with brine (10 mL), dried with anhydrous Na_2SO_4, filtered and concentrated. The resulting residue was purified by flash column chromatography (2.5 g silica, CV = 5 mL, 60-100% dichloromethane/pentane then 0-5% diethyl ether/dichloromethane) to give the desired compound *ent-(E)*-5 (3.3 mg, 8 µmol, 67%, $E/Z > 20{:}1$) as a clear oil.

R_f = 0.59 (dichloromethane).

IR (ν_{max} cm^{-1}): 3291 (medium), 2966 (medium), 2918 (medium), 1559 (weak), 1541 (weak), 1457 (medium), 1242 (weak), 1082 (medium), 823 (weak), 770 (weak).

HRMS (ESI) m/z : $[M+H]^+$ calculated for $C_{15}H_{21}Br_2O_2$ 390.9903, 392.9883, 394.9862; found 390.9903, 392.9881, 394.9862.

^1H NMR (600 MHz, $CDCl_3$) δ 6.23 (dt, J = 15.3, 7.3 Hz, 1H, H4), 5.60 (dq, J = 15.9, 1.8 Hz, 1H, H3), 4.41 (dt, J = 10.8, 2.5 Hz, 1H, H12), 4.36 (d, J = 2.5 Hz, 1H, H7), 4.32 (d, J = 2.3 Hz, 1H, H9), 4.10 (dt, J = 10.0, 3.2 Hz, 1H, H13), 4.06 (dd, J = 9.8, 3.3 Hz, 1H, H10a), 3.85 (t, J = 7.1 Hz, 1H, H6a), 2.81 (d, J = 2.2 Hz, 1H, H1), 2.48 (dtd, J = 14.2, 7.1, 1.6 Hz, 1H, H5), 2.41 (dtd, J = 14.6, 7.4, 1.4 Hz, 1H, H5'), 2.09 (dqd, J = 14.5, 7.3, 3.6 Hz, 1H, H14), 1.96 – 1.93 (m, 1H, H11), 1.92 (dd, J = 10.8, 2.9 Hz, 1H, H8b), 1.87 (dd, J = 10.3, 2.7 Hz, 1H, H8a), 1.85 – 1.82 (m, 1H, H14'), 1.79 (ddd, J = 14.6, 9.8, 2.4 Hz, 1H, H11'), 1.08 (t, J = 7.2 Hz, 3H, H15).

^{13}C NMR (151 MHz, $CDCl_3$) δ 142.0 (C4), 111.5 (C3), 82.3 (C2), 81.8 (C6), 81.7 (C10), 80.0 (C9), 77.2* (C7), 76.6 (C1), 62.2 (C13), 54.6 (C12), 39.9 (C11), 35.3 (C8), 34.6 (C5), 29.1 (C14), 12.7 (C15). **Underneath $CDCl_3$ peak. Shift located and assigned using HSQC and HMBC data.*

$[\alpha]_D^{25}$ = +119.4 (c=0.32, $CHCl_3$).

(1S,3R,4S,6S)-3-((2S,3S)-2,3-Dibromopentyl)-6-((E)-pent-2-en-4-yn-1-yl)-2,5-dioxabicyclo[2.2.1]heptane, (E)-ocellenyne (*(E)*-8)

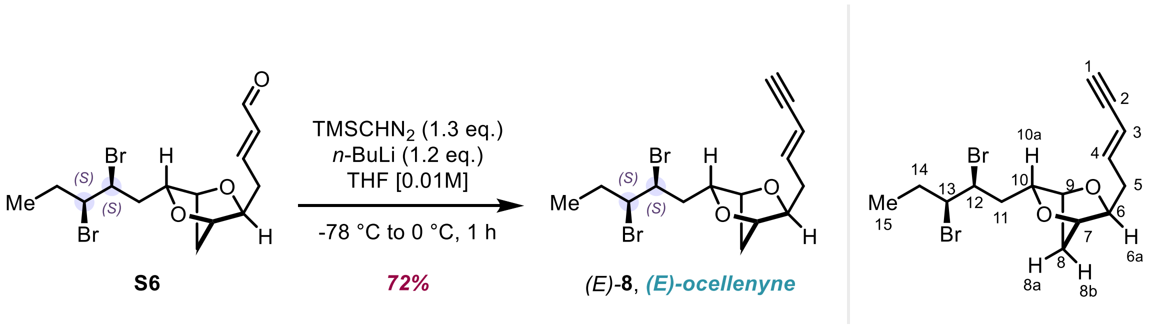

**on small scale it is preferable to prepare a stock solution of lithiated-(trimethylsilyl)diazomethane and transfer a portion of this to the reaction mixture.*

(Trimethylsilyl)diazomethane solution (2.0M in hexanes, 79 µL, 158 µmol, 13 eq.) was added to a flame dried Schlenk tube through a suba seal. Dry THF (1.83 mL) was added and the solution was cooled to -78 °C whilst stirring. n-Butyllithium solution (1.6M in hexanes, 91 µL, 145 µmol, 12 eq.) was added dropwise and the resultant orange solution was stirred for 30 minutes at -78 °C. In a separate vessel, compound S6 (4.8 mg, 12 µmol) was dissolved in dry THF (1.0 mL) and cooled to -78 °C whilst stirring. A portion of lithiated-(trimethylsilyl)diazomethane solution (0.2 mL, 15 µmol, 1.2 eq.) was quickly transferred via syringe to the solution of starting material S6. The reaction mixture was stirred for 1 h at -78 °C. The reaction mixture was then warmed to 0 °C for 10 minutes before being quenched with sat. aq. NH_4Cl solution (2 mL) and AcOH (4 drops). H_2O (2 mL) and CH_2Cl_2 (8 mL) was added, and the organic layer was then separated. The aqueous layer was extracted with CH_2Cl_2 (2 x 8 mL). The combined organics were washed with brine (10 mL), dried with anhydrous Na_2SO_4, filtered and concentrated. The resulting residue was purified by flash column chromatography (2.5 g silica, CV = 5 mL, 60-100% dichloromethane/pentane then 0-5% diethyl ether/dichloromethane) to give (E)-ocellenyne (E)-8 (3.4 mg, 9 µmol, 72%, E/Z > 20:1) as a clear oil.

R_f = 0.59 (dichloromethane).

IR (ν_{max} cm^{-1}): 3291 (medium), 2916 (medium), 1653 (weak), 1559 (weak), 1507 (weak), 1457 (medium), 1221 (weak), 1077 (medium), 952 (medium), 920 (weak), 801 (weak), 767 (weak) 668 (medium), 648 (medium).

HRMS (ESI) m/z : $[M+H]^+$ calculated for $C_{15}H_{21}Br_2O_2$ 390.9903, 392.9883, 394.9862; found 390.9902, 392.9883, 394.9863.

^{1}H NMR (600 MHz, $CDCl_3$) δ 6.22 (dt, J = 15.3, 7.3 Hz, 1H, H4), 5.59 (dq, J = 16.1, 1.8 Hz, 1H, H3), 4.35 (s, 1H, H7), 4.29 (d, J = 1.9 Hz, 1H, H9), 4.21 (ddd, J = 8.4, 5.9, 2.6 Hz, 1H, H12), 4.12 (dt, J = 9.6, 3.2 Hz, 1H, H13), 3.98 (t, J = 7.0 Hz, 1H, H10a), 3.85 (t, J = 7.1 Hz, 1H, H6a), 2.81 (d, J = 2.2 Hz, 1H, H1), 2.51 – 2.44 (m, 1H, H5), 2.42 – 2.36 (m, 1H, H5'), 2.15 (dt, J = 14.6, 6.5 Hz, 1H, H11), 2.07 (dqd, J = 14.5, 7.3, 3.8 Hz, 1H, H14), 1.94 – 1.83 (m, 4H, H8a, H8b, H11', H14'), 1.08 (t, J = 7.2 Hz, 3H, H15).

^{1}H NMR (600 MHz, C_6D_6) δ 6.15 (dt, J = 16.1, 7.3 Hz, 1H, H4), 5.47 (dq, J = 15.9, 1.8 Hz, 1H, H3), 3.99 (td, J = 7.2, 2.5 Hz, 1H, H12), 3.92 (dd, J = 7.8, 6.3 Hz, 1H, H10a), 3.84 (ddd, J = 9.0, 4.7, 2.5 Hz, 1H, H13), 3.80 (d, J = 2.2 Hz, 1H, H9), 3.70 (d, J = 2.4 Hz, 1H, H7), 3.34 (td, J = 7.0, 1.1 Hz, 1H, H6a), 2.55 (d, J = 2.2 Hz, 1H, H1), 2.33 – 2.25 (m, 1H, H5), 2.17 – 2.08 (m, 1H, H5'), 1.98 (dt, J = 14.5, 6.5 Hz, 1H, H11), 1.76 – 1.66 (m, 3H, H11', H14), 1.30 – 1.23 (m, 2H, H8a, H8b), 0.75 (t, J = 7.2 Hz, 3H, H15).

^{13}C NMR (151 MHz, $CDCl_3$) δ 141.8 (C4), 111.6 (C3), 82.2 (C2), 81.8 (C6, C10), 79.3 (C9), 77.1* (C7), 76.6 (C1), 60.7 (C13), 53.4 (C12), 40.1 (C11), 34.9 (C8), 34.6 (C5), 29.3 (C14), 12.7 (C15).

^{13}C NMR (151 MHz, C_6D_6) δ 142.2 (C4), 111.7 (C3), 82.5 (C2), 81.7 (C10), 81.6 (C6), 79.1 (C9), 77.1 (C1), 77.0 (C7), 60.8 (C13), 53.8 (C12), 40.9 (C11), 34.8 (C5), 34.7 (C8), 30.0 (C14), 12.4 (C15).

$[\alpha]_D^{25}$ = +28.5 (c=0.34, $CHCl_3$). c.f. literature[4]: $[\alpha]_D^{25}$ = +3.21 (c=0.53, $CHCl_3$). *Note that both of our optical rotation measurements for synthetic (*E*)- and (*Z*)- ocellenyne are different by roughly a factor of 10 to that reported by Scheuer for natural (*E*)- and (*Z*)- ocellenyne. Since it is unusual to report optical rotation data to 2 decimal places, we suspect that there may have been an error in the placement of the decimal place in the reported data. Thus, we suspect the true value for (*E*)-ocellenyne to be $[\alpha]_D^{25}$ = +32.1 (c=0.53, $CHCl_3$), in better keeping with our recorded value.

(1*S*,3*R*,4*S*,6*S*)-3-((2*R*,3*R*)-2,3-Dibromopentyl)-6-((*Z*)-3-iodoallyl)-2,5-dioxabicyclo[2.2.1]heptane (19)

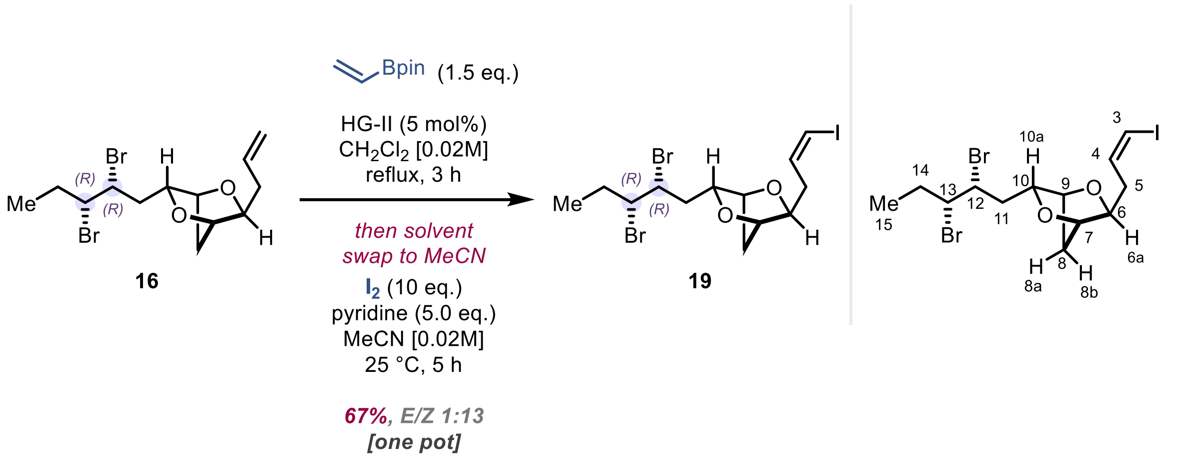

Step 1. **All solvents used in this reaction were sparged with argon for 15 minutes before use.* Compound 16 (11 mg, 30 µmol) and vinylboronic acid pinacol ester (8.0 µL, 45 µmol, 1.5 eq.) were dissolved in dry CH_2Cl_2 (0.75 mL) and stirred. Hoveyda-Grubb's 2nd generation catalyst (0.9 mg, 1 µmol, 5 mol%) was dissolved in dry CH_2Cl_2 (0.75 mL) and added to the reaction mixture. The reaction mixture was then heated to reflux for 3 h (*alternatively the reaction can be heated at 30 °C for 18 h*). TLC analysis (dichloromethane) at this stage indicated completion of reaction. The reaction mixture was cooled to rt and DMSO (1 drop) was added before all volatiles were removed under a constant stream of nitrogen.

Step 2. *Note that this reaction works well using either THF or CH_2Cl_2 as the solvent, however higher yields were observed with MeCN. The use of fewer equivalents of iodine resulted in longer reaction times and incomplete conversion of starting material. The reaction is also best conducted with exclusion of light throughout.* The residue from the previous step was immediately dissolved in MeCN (0.3 mL) and stirred at rt. Pyridine (12 µL, 149 µmol, 5.0 eq.) was then added. Iodine (76 mg, 299 µmol, 10.0 eq.) was dissolved in MeCN (0.4 mL) and sonicated to aid dissolution (note poor solubility). The solution of iodine was then added to the reaction mixture and the residual undissolved iodine was redissolved in MeCN (3 x 0.4 mL) and added to the reaction mixture. The reaction was then heated at 25 °C for 5 h. TLC analysis (1% diethyl ether/dichloromethane) at this stage indicated completion of reaction. The reaction was quenched with sat. aq. $Na_2S_2O_3$ solution (6 mL) and stirred vigorously for 5 minutes (reaction decolourises at this stage). The solution was diluted with H_2O (2 mL) and the aqueous layer was extracted with diethyl ether (3 x 8 mL). The combined organics were washed with sat. aq. $Na_2S_2O_3$ solution (6 mL) and brine (10 mL), dried with anhydrous Na_2SO_4, filtered and concentrated. The resulting residue was purified by flash column chromatography (7.5 g silica, CV = 15 mL, 40-100% dichloromethane) to give the desired compound 19 (9.9 mg, 20 µmol, 67%, E/Z = 1:13) as a clear oil.

R_f = 0.25 (50% dichloromethane/pentane).

IR (ν_{max} cm^{-1}): 2967 (medium), 2917 (medium), 1609 (weak), 1456 (weak), 1380 (weak), 1300 (medium), 1254 (medium), 1081 (strong), 1062 (strong), 944 (strong), 823 (medium), 687 (weak), 625 (weak).

HRMS (APCI) m/z : $[M+H]^+$ calculated for $C_{13}H_{20}Br_2IO_2$ 492.8870, 494.8849, 496.8829; found 492.8876, 494.8855, 496.8834.

^{1}H NMR (600 MHz, $CDCl_3$) δ 6.36 (dt, J = 7.5, 1.3 Hz, 1H, H3), 6.27 (q, J = 7.0 Hz, 1H, H4), 4.43 (dt, J = 10.7, 2.5 Hz, 1H, H12), 4.36 (d, J = 2.5 Hz, 1H, H7), 4.33 (d, J = 2.3 Hz, 1H, H9), 4.15 (dd, J = 10.0, 3.3 Hz, 1H, H10a), 4.11 (ddd, J = 9.9, 3.7, 2.6 Hz, 1H, H13), 3.92 (ddd, J = 7.7, 6.2, 1.1 Hz, 1H, H6a), 2.58 (m, 1H, H5), 2.44 – 2.37 (m, 1H, H5'), 2.09 (dqd, J = 14.5, 7.2, 3.6 Hz, 1H, H14), 1.98 – 1.95 (m, 1H, H11), 1.94 (dd, J = 10.2, 2.3 Hz, 1H, H8b), 1.88 (dd, J = 10.6, 2.6 Hz, 1H, H8a), 1.86 – 1.83 (m, 1H, H14'), 1.80 (ddd, J = 14.6, 9.8, 2.3 Hz, 1H, H11'), 1.08 (t, J = 7.3 Hz, 3H, H15).

^{13}C NMR (151 MHz, $CDCl_3$) δ 137.3 (C4), 84.9 (C3), 81.7 (C10), 81.1 (C6), 80.0 (C9), 77.5 (C7), 62.2 (C13), 54.6 (C12), 39.9 (C11), 36.6 (C5), 35.4 (C8), 29.2 (C14), 12.7 (C15).

$[\alpha]_D^{25}$ = +107.0 (c=0.99, $CHCl_3$).

(1S,3R,4S,6S)-3-((2S,3S)-2,3-Dibromopentyl)-6-((Z)-3-iodoallyl)-2,5-dioxabicyclo[2.2.1]heptane (20)

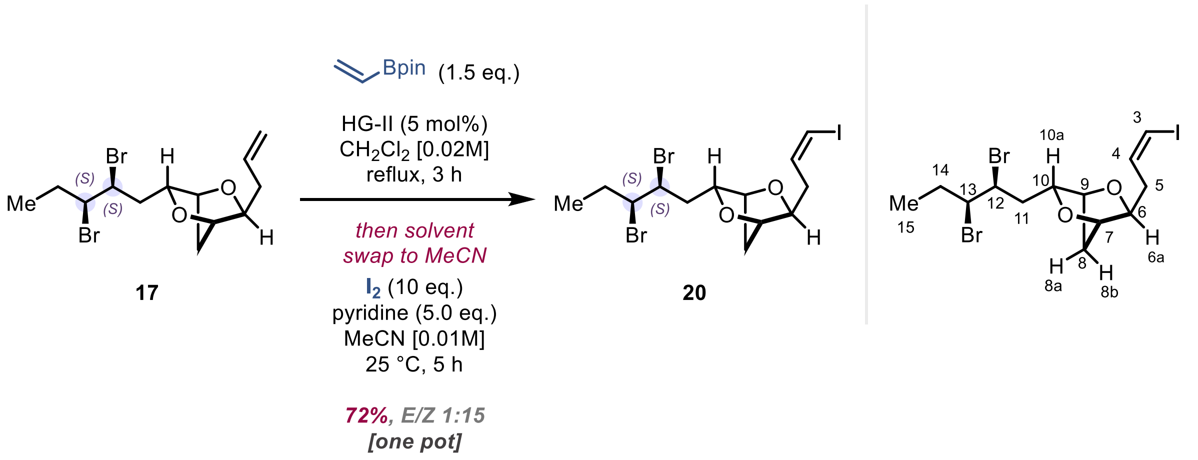

Step 1. **All solvents used in this reaction were sparged with argon for 15 minutes before use.* Compound 17 (6.0 mg, 16 µmol) and vinylboronic acid pinacol ester (4.3 µL, 24 µmol, 1.5 eq.) were dissolved in dry CH_2Cl_2 (0.5 mL) and stirred. Hoveyda-Grubb's 2nd generation catalyst (0.5 mg, 1 µmol, 5 mol%) was dissolved in dry CH_2Cl_2 (0.5 mL) and added to the reaction mixture. The reaction mixture was then heated to reflux for 3 h (*alternatively the reaction can be heated at 30 °C for 18 h*). TLC analysis (dichloromethane) at this stage indicated completion of reaction. The reaction mixture was cooled to rt and DMSO (1 drop) was added before all volatiles were removed under a constant stream of nitrogen.

Step 2. *Note that this reaction works well using either THF or CH_2Cl_2 as the solvent, however higher yields were observed with MeCN. The use of fewer equivalents of iodine resulted in longer reaction times and incomplete conversion of starting material. The reaction is also best conducted with exclusion of light throughout.* The residue from the previous step was immediately dissolved in MeCN (0.2 mL) and stirred at rt. Pyridine (6.6 µL, 82 µmol, 5.0 eq.) was then added. Iodine (41 mg, 163 µmol, 10.0 eq.) was dissolved in MeCN (0.3 mL) and sonicated to aid dissolution (note poor solubility). The solution of iodine was then added to the reaction mixture and the residual undissolved iodine was redissolved in MeCN (3 x 0.3 mL) and added to the reaction mixture. The reaction was then heated at 25 °C for 5 h. TLC analysis (1% diethyl ether/dichloromethane) at this stage indicated completion of reaction. The reaction was quenched with sat. aq. $Na_2S_2O_3$ solution (6 mL) and stirred vigorously for 5 minutes (reaction decolourises at this stage). The solution was diluted with H_2O (2 mL) and the aqueous layer was extracted with diethyl ether (3 x 8 mL). The combined organics were washed with sat. aq. $Na_2S_2O_3$ solution (6 mL) and brine (10 mL), dried with anhydrous Na_2SO_4, filtered and concentrated. The resulting residue was purified by flash column chromatography (7.5 g silica, CV = 15 mL, 40-100% dichloromethane) to give the desired compound 20 (5.8 mg, 12 µmol, 72%, E/Z = 1:15) as a clear oil.

R_f = 0.25 (50% dichloromethane/pentane).

IR (ν_{max} cm^{-1}): 2917 (medium), 1457 (weak), 1302 (weak), 1254 (weak), 1072 (medium), 948 (medium), 768 (medium), 668 (medium).

HRMS (APCI) *m/z* : $[M+H]^+$ calculated for $C_{13}H_{20}Br_2IO_2$ 492.8870, 494.8849, 496.8829; found 492.8869, 494.8848, 496.8827.

^{1}H NMR (600 MHz, $CDCl_3$) δ 6.36 (dt, *J* = 7.4, 1.4 Hz, 1H, H3), 6.25 (q, *J* = 7.1 Hz, 1H, H4), 4.34 (s, 1H, H7), 4.29 (d, *J* = 2.1 Hz, 1H, H9), 4.24 (ddd, *J* = 8.4, 6.5, 2.5 Hz, 1H, H12), 4.16 (ddd, *J* = 9.7, 3.9, 2.5 Hz, 1H, H13), 4.07 (t, *J* = 7.0 Hz, 1H, H10a), 3.92 (ddd, *J* = 7.6, 6.3, 1.1 Hz, 1H, H6a), 2.56 (dddd, *J* = 14.7, 8.1, 6.8, 1.4 Hz, 1H, H5), 2.39 (dt, *J* = 13.5, 6.7 Hz, 1H, H5'), 2.18 (dt, *J* = 14.3, 6.5 Hz, 1H, H11), 2.06 (dqd, *J* = 14.5, 7.2, 3.9 Hz, 1H, H14), 1.96 – 1.86 (m, 4H, H8a, H8b, H11', H14'), 1.08 (t, *J* = 7.3 Hz, 3H, H15).

^{13}C NMR (151 MHz, $CDCl_3$) δ 137.1 (C4), 85.0 (C3), 81.7 (C10), 81.0 (C6), 79.4 (C9), 77.4* (C7), 60.6 (C13), 53.6 (C12), 40.3 (C11), 36.6 (C5), 35.0 (C8), 29.7 (C14), 12.7 (C15). **Underneath $CDCl_3$ peak. Shift located and assigned using HSQC and HMBC data.*

$[\alpha]_D^{25}$ = +14.6 (c=0.57, $CHCl_3$).

(1S,3R,4S,6S)-3-((2R,3R)-2,3-Dibromopentyl)-6-((Z)-pent-2-en-4-yn-1-yl)-2,5-dioxabicyclo[2.2.1]heptane (*ent-(Z)*-5)

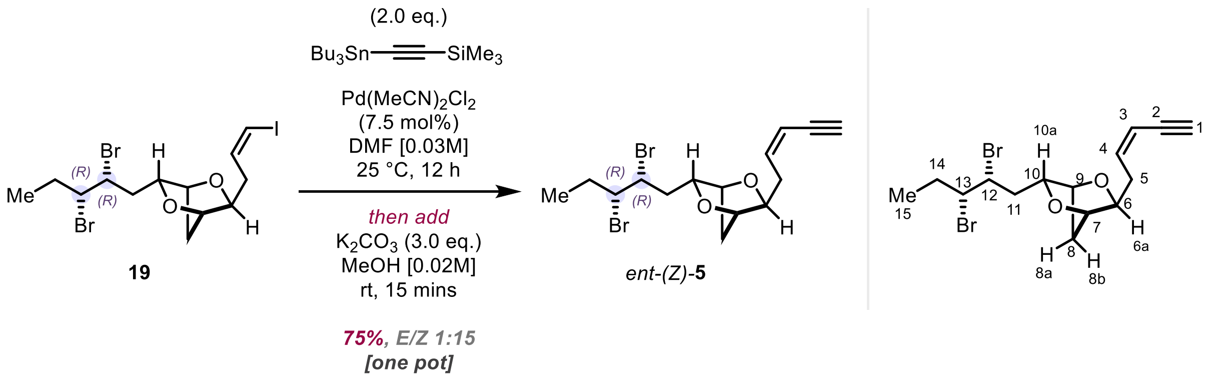

**All solvents used in this reaction were sparged with argon for 1 hour before use.* Compound 19 (9.8 mg, 20 µmol) and trimethyl[(tributylstannyl)ethynyl]silane (15 mg, 40 µmol, 2.0 eq.) were dissolved in DMF (0.6 mL) and stirred at rt. Bis(acetonitrile)palladium dichloride (0.4 mg, 1 µmol, 7.5 mol%) was dissolved in DMF (50 µL) and added to the reaction, with a colour change from clear to opaque black being observed. The reaction was then heated at 25 °C for 12 h. TLC analysis (0.5% diethyl ether/dichloromethane) at this stage indicated completion of reaction. The solution was diluted with MeOH (0.6 mL) and K_2CO_3 (8.2 mg, 60 µmol, 3.0 eq.) was added. The reaction was then stirred at rt for 15 minutes. TLC analysis (0.5% diethyl ether/dichloromethane) at this stage indicated completion of reaction. The reaction was quenched with sat. aq. NH_4Cl solution (0.6 mL), filtered through a Celite® plug (10 mL) and washed on the filter with diethyl ether (3 x 15 mL). The filtrate was washed with H_2O (15 mL) and brine (2 x 25 mL) before being dried with anhydrous Na_2SO_4, filtered and concentrated. The resulting residue was purified by flash column chromatography (10 g silica, CV = 20 mL, 10-50% diethyl ether/pentane) to give the desired compound *ent-(Z)*-5 (5.8 mg, 15 µmol, 75%, E/Z = 1:15) as a clear oil.

R_f = 0.27 (2.5% diethyl ether/toluene).

IR (ν_{max} cm^{-1}): 3291 (medium), 2917 (medium), 1457 (medium), 1244 (weak), 1191 (weak), 1081 (strong), 1068 (strong), 944 (strong), 819 (medium), 768 (medium).

HRMS (ESI) m/z : $[M+H]^+$ calculated for $C_{15}H_{21}Br_2O_2$ 390.9903, 392.9883, 394.9862; found 390.9902, 392.9883, 394.9861.

^{1}H NMR (600 MHz, $CDCl_3$) δ 6.07 (dt, J = 10.9, 7.5 Hz, 1H, H4), 5.58 (ddt, J = 10.9, 2.4, 1.3 Hz, 1H, H3), 4.43 (dt, J = 10.8, 2.5 Hz, 1H, H12), 4.36 (d, J = 2.5 Hz, 1H, H7), 4.33 (d, J = 2.4 Hz, 1H, H9), 4.14 (dd, J = 10.0, 3.2 Hz, 1H, H10a), 4.11 (dt, J = 10.0, 3.2 Hz, 1H, H13), 3.90 (ddd, J = 7.9, 6.3, 1.1 Hz, 1H, H6a), 3.12 (d, J = 2.3 Hz, 1H, H1), 2.83 – 2.74 (m, 1H, H5), 2.61 – 2.53 (m, 1H, H5'), 2.09 (dqd, J = 14.5, 7.2, 3.6 Hz, 1H, H14), 1.98 – 1.94 (m, 1H, H11), 1.94 – 1.90 (m, 1H, H8b), 1.87 (dd, J = 10.3, 2.4 Hz, 1H, H8a), 1.86 – 1.82 (m, 1H, H14'), 1.79 (ddd, J = 14.6, 10.0, 2.4 Hz, 1H, H11'), 1.08 (t, J = 7.2 Hz, 3H, H15).

^{13}C NMR (151 MHz, $CDCl_3$) δ 141.3 (C4), 110.7 (C3), 82.3 (C1), 81.7 (C6), 81.6 (C10), 80.2 (C2), 80.0 (C9), 77.6 (C7), 62.2 (C13), 54.6 (C12), 39.9 (C11), 35.4 (C8), 32.1 (C5), 29.2 (C14), 12.7 (C15).

$[\alpha]_D^{25}$ = +116.9 (c=0.58, $CHCl_3$).

(1*S*,3*R*,4*S*,6*S*)-3-((2*S*,3*S*)-2,3-Dibromopentyl)-6-((*Z*)-pent-2-en-4-yn-1-yl)-2,5-dioxabicyclo[2.2.1]heptane, (*Z*)-ocellenyne ((*Z*)-8)

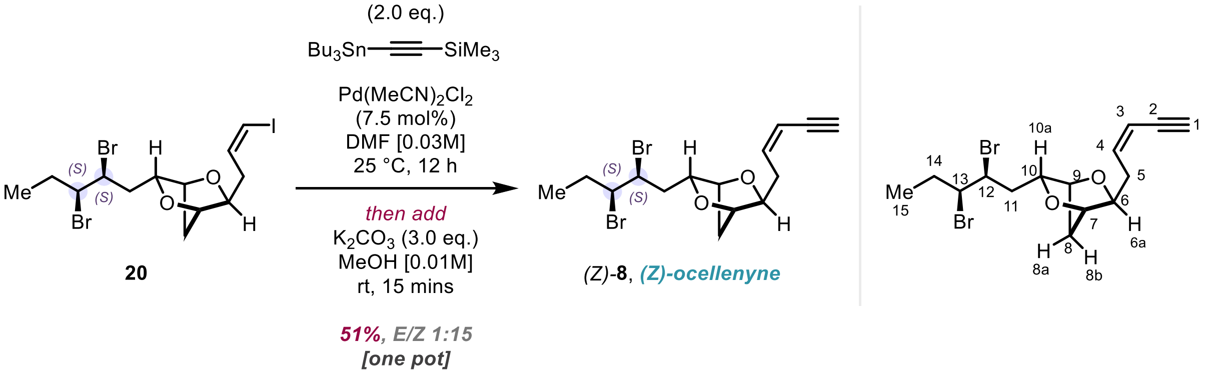

**All solvents used in this reaction were sparged with argon for 1 hour before use.* Compound 20 (5.7 mg, 12 µmol) and trimethyl[(tributylstannyl)ethynyl]silane (8.9 mg, 23 µmol, 2.0 eq.) were dissolved in DMF (0.3 mL) and stirred at rt. Bis(acetonitrile)palladium dichloride (0.2 mg, 1 µmol, 7.5 mol%) was dissolved in DMF (100 µL) and added to the reaction, with a colour change from clear to opaque black being observed. The reaction was then heated at 25 °C for 12 h. TLC analysis (0.5% diethyl ether/dichloromethane) at this stage indicated completion of reaction. The solution was diluted with MeOH (0.4 mL) and K_2CO_3 (4.8 mg, 35 µmol, 3.0 eq.) was added. The reaction was then stirred at rt for 15 minutes. TLC analysis (0.5% diethyl ether/dichloromethane) at this stage indicated completion of reaction. The reaction was quenched with sat. aq. NH_4Cl solution (0.4 mL), filtered through a Celite® plug (10 mL) and washed on the filter with diethyl ether (3 x 15 mL). The filtrate was washed with H_2O (15 mL) and brine (2 x 25 mL) before being dried with anhydrous Na_2SO_4, filtered and concentrated. The resulting residue was purified by flash column chromatography (10 g silica, CV = 20 mL, 10-50% diethyl ether/pentane) to give (*Z*)-ocellenyne (*Z*)-8 with a 10% impurity resulting from degradation of the vicinal dibromide. The compound was further purified by preparative TLC (2.5% diethyl ether/toluene) to give (*Z*)-ocellenyne (*Z*)-8 (2.3 mg, 6 µmol, 51%, *E*/*Z* = 1:15) as a clear oil. *Note that on this scale it is hard to assess if the compound is an oil or solid.*

R_f = 0.27 (2.5% diethyl ether/toluene).

IR (ν_{max} cm^{-1}): 3294 (weak), 2961 (strong), 2920 (strong), 1457 (weak), 1377 (weak), 1076 (strong), 948 (medium), 914 (medium), 816 (medium), 743 (medium), 669 (medium).

HRMS (APCI) *m/z* : [M+H]$^+$ calculated for $C_{15}H_{21}Br_2O_2$ 390.9903, 392.9883, 394.9862; found 390.9898, 392.9877, 394.9857.

^{1}H NMR (600 MHz, $CDCl_3$) δ 6.06 (dt, *J* = 10.8, 7.6 Hz, 1H, H4), 5.58 (dq, *J* = 10.9, 1.5 Hz, 1H, H3), 4.35 (t, *J* = 1.5 Hz, 1H, H7), 4.29 (d, *J* = 2.1 Hz, 1H, H9), 4.23 (ddd, *J* = 8.6, 6.4, 2.5 Hz, 1H, H12), 4.14 (ddd, *J* = 9.7, 3.9, 2.5 Hz, 1H, H13), 4.05 (t, *J* = 7.0 Hz, 1H, H10a), 3.90 (ddd, *J* = 7.7, 6.1, 1.1 Hz, 1H, H6a), 3.12 (d, *J* = 2.3 Hz, 1H, H1), 2.76 (dtd, *J* = 14.0, 7.8, 1.4 Hz, 1H, H5), 2.55 (dt, *J* = 14.0, 6.9 Hz, 1H, H5'), 2.17 (dt, *J* = 14.4, 6.5 Hz, 1H, H11), 2.06 (dqd, *J* = 14.5, 7.3, 3.9 Hz, 1H, H14), 1.95 – 1.86 (m, 4H, H8a, H8b, H11', H14'), 1.08 (t, *J* = 7.2 Hz, 3H, H15).

^{13}C NMR (151 MHz, $CDCl_3$) δ 141.1 (C4), 110.7 (C3), 82.2 (C1), 81.7 (C6), 81.6 (C10), 80.3 (C2), 79.3 (C9), 77.5 (C7), 60.6 (C13), 53.6 (C12), 40.3 (C11), 35.0 (C8), 32.1 (C5), 29.6 (C14), 12.7 (C15).

$[\alpha]_D^{25}$ = +22.0 (c=0.22, $CHCl_3$). c.f. literature[4]: $[\alpha]_D^{25}$ = +2.22 (c=0.27, $CHCl_3$). *Note that both of our optical rotation measurements for synthetic (*E*)- and (*Z*)- ocellenyne are different by roughly a factor of 10 to that reported by Scheuer for natural (*E*)- and (*Z*)- ocellenyne. Since it is unusual to report optical rotation data to 2 decimal places we suspect that there may have been an error in the placement of the decimal place in the reported data. Thus, we suspect the true value for (*Z*)-ocellenyne to be $[\alpha]_D^{25}$ = +22.2 (c=0.27, $CHCl_3$), in better keeping with our recorded value.

4. Comparison of Synthetic and Natural Ocellenyne Data

Optical rotation:

Natural (*E*)-ocellenyne: $[\alpha]_D^{25}$ = +3.21 (c=0.53, $CHCl_3$).[4]
Synthetic (*E*)-8: $[\alpha]_D^{25}$ = +28.5 (c=0.34, $CHCl_3$).
Natural (*Z*)-ocellenyne: $[\alpha]_D^{25}$ = +2.22 (c=0.27, $CHCl_3$).[4]
Synthetic (*Z*)-8: $[\alpha]_D^{25}$ = +22.0 (c=0.22, $CHCl_3$).
Our optical rotation measurements for synthetic (*E*)- and (*Z*)- ocellenyne (*E*/*Z*)-8 are different by roughly a factor of 10 to that reported by Scheuer for natural (*E*)- and (*Z*)- ocellenyne. Since it is unusual to report optical rotation data to 2 decimal places, we suspect that there may have been an error in the placement of the decimal place in the reported data.

Comparison of ^{1}H NMR data for synthetic (*E*)-8 vs natural (*E*)-ocellenyne

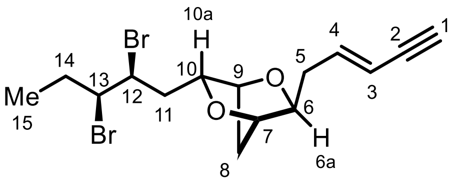

	Synthetic sample (S)	Natural sample (N)	
Proton	^{1}H NMR (400 MHz, $CDCl_3$) / ppm	^{1}H NMR (360 MHz, $CDCl_3$) / ppm	Δδ (N-S) / ppm
H15	1.08 (t, *J* = 7.2 Hz, 3H)	1.02 (t, *J* = 7.0 Hz, 3H)	-0.06
H14	2.06 (dtd, *J* = 14.5, 7.2, 3.8, 1H)	2.07 (dqd, *J* = 14.5, 7.0, 3.0 Hz, 1H)	0.01
H14'	1.90 (m, 1H)	1.87 (ddq, *J* = 14.5, 9.5, 7.0 Hz, 1H)	-0.03
H13	4.12 (ddd, *J* = 9.8, 3.9, 2.6 Hz, 1H)	4.12 (ddd, *J* = 9.5, 3.0, 2.5 Hz, 1H)	0.00
H12	4.21 (ddd, *J* = 8.4, 5.9, 2.6 Hz, 1H)	4.21 (ddd, *J* = 8.0, 6.5, 2.5 Hz, 1H)	0.00
H11	2.15 (ddd, *J* = 14.6, 6.9, 5.9 Hz, 1H)	2.15 (ddd, *J* = 14.5, 6.5, 6.5 Hz, 1H)	0.00
H11'	1.90 (m, 1H)	1.91 (ddd, *J* = 14.5, 8.0, 7.0 Hz, 1H)	0.01
H10a	3.98 (t, *J* = 7.0 Hz, 1H)	3.98 (dd, *J* = 7.0, 6.5 Hz, 1H)	0.00
H9	4.29 (br s, 1H)	4.29 (br s, 1H)	0.00
H8	1.90 (m, 2H)	1.90 (br s, 2H)	0.00
H7	4.35 (br s, 1H)	4.35 (br s, 1H)	0.00
H6a	3.85 (td, *J* = 7.0, 1.1 Hz, 1H)	3.85 (br dd, *J* = 7.5, 7.0 Hz, 1H)	0.00
H5	2.47 (dtd, *J* = 14.0, 6.9, 1.7 Hz, 1H)	2.46 (ddd, *J* = 13.5, 7.5, 7.5 Hz, 1H)	-0.01
H5'	2.39 (dtd, *J* = 14.4, 7.4, 1.4 Hz, 1H)	2.41 (ddd, *J* = 13.5, 7.5, 7.0 Hz, 1H)	0.02
H4	6.22 (dt, *J* = 16.1, 7.4 Hz, 1H)	6.22 (ddd, *J* = 15.5, 7.5, 7.5 Hz, 1H)	0.00
H3	5.59 (dq, *J* = 16.0, 1.6 Hz, 1H)	5.59 (dd, *J* = 15.5, 1.5 Hz, 1H)	0.00
H1	2.81 (d, *J* = 2.2 Hz, 1H)	2.80 (d, *J* = 1.5 Hz, 1H)	-0.01

	Synthetic sample (S)	Natural Sample (N)	
Proton	^{1}H NMR (400 MHz, C_6D_6) / ppm	^{1}H NMR (360 MHz, C_6D_6) / ppm	Δδ (N-S) / ppm
H15	0.75 (t, J = 7.2 Hz, 3H)	0.79 (t, J = 7.0 Hz, 3H)	0.04
H14	1.71 (m, 2H)	1.76 (dq, J = 7.0, 7.0 Hz, 2H)	0.05
H13	3.84 (ddd, J = 8.0, 5.2, 2.5 Hz, 1H)	3.87[a] ([c])	0.03
H12	3.99 (td, J = 7.1, 2.5 Hz, 1H)	4.03[a] (td, J = 7.0, 2.5 Hz, 1H)	0.04
H11	1.98 (dt, J = 14.5, 6.5 Hz, 1H)	2.02 ([c])	0.04
H11'	1.71 (m, 1H)	1.71 ([c])	0
H10a	3.92 (dd, J = 7.8, 6.3 Hz, 1H)	3.96 ([c])	0.04
H9	3.80 (d, J = 2.0 Hz, 1H)	3.84 ([c])	0.04
H8	1.26 (m, 2H)	1.74[b] ([c])	0.48
H7	3.70 (br s, 1H)	3.74 ([c])	0.04
H6a	3.34 (td, J = 7.1, 1.1 Hz, 1H)	3.38 ([c])	0.04
H5	2.29 (m, 1H)	2.31 ([c])	0.02
H5'	2.12 (m, 1H)	2.16 ([c])	0.04
H4	6.15 (dt, J = 16.1, 7.4 Hz, 1H)	6.19 ([c])	0.04
H3	5.47 (dq, J = 16.0, 1.8 Hz, 1H)	5.51 ([c])	0.04
H1	2.55 (d, J = 2.2 Hz, 1H)	2.59 ([c])	0.04

[a] Our assignments of protons 12 and 13 are reversed from their original assignment in reference[4]. [b] Suspected mistake in chemical shift data reported. [c] No multiplicity data reported.

Comparison of ^{13}C NMR data for synthetic (E)-8 vs natural (E)-ocellenyne – *there is a 1.1 ppm difference between the chemical shifts of the C-7 carbons which may be a typographical error in the original manuscript.*

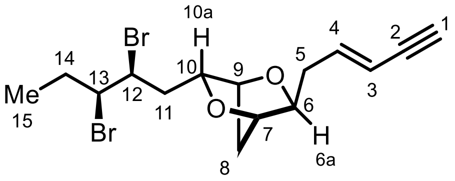

	Synthetic sample (S)	Natural sample[a] (N)	
Carbon	^{13}C NMR (151 MHz, CDCl$_3$) / ppm	^{13}C NMR (25 MHz, CDCl$_3$) / ppm	Δδ (N-S) / ppm
C15	12.7	12.9	0.2
C14	29.4	29.4	0.0
C13	60.7	60.7	0.0
C12	53.4	53.5	0.1
C11	40.1	40.2	0.1
C10	81.8	81.8[b]	0.0
C9	79.3	79.3	0.0
C8	35.0	35.0	0.0
C7	77.1	78.2	1.1
C6	81.8	81.8[b]	0.0
C5	34.6	34.7	0.1
C4	141.8	141.7	-0.1
C3	111.6	111.6	0.0
C2	82.2	81.8[b]	-0.4
C1	76.6	76.6	0.0

[a] carbon resonances are not assigned in reference[4]. [b] three ^{13}C NMR resonances are all stated as being at 81.8 ppm in the isolation paper; since the C2 quaternary carbon resonance is of lower signal intensity than the non-quaternary resonances it is likely that this resonance was not observed when recording data with a less powerful 25 MHz ^{13}C NMR probe.

Comparison of ^{1}H NMR data for synthetic (*Z*)-8 vs natural (*Z*)-ocellenyne*

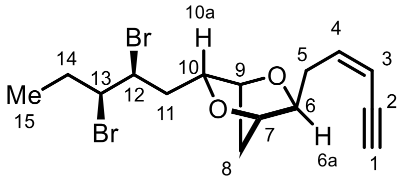

	Synthetic sample (S)	Natural sample (N)	
Proton	^{1}H NMR (400 MHz, CDCl$_3$) / ppm	^{1}H NMR (100 MHz, CDCl$_3$) / ppm	Δδ (N-S) / ppm
H15	1.08 (t, *J* = 7.2 Hz, 3H)	1.07 (t, *J* = 7.0 Hz, 3H)	-0.01
H14	2.05 (dtd, *J* = 14.5, 7.3, 3.9 Hz, 1H)	2.05 (dqd, *J* = 13.0, 7.0, 3.0 Hz, 1H)	0.00
H14'	1.90 (m, 1H)	1.85 (ddq, *J* = 13.0, 9.0, 7.0 Hz, 1H)	-0.05
H13	4.14 (ddd, *J* = 9.7, 3,9, 2.5 Hz, 1H)	4.10 (ddd, *J* = 9.0, 3.0, 3.0 Hz, 1H)	-0.04
H12	4.23 (ddd, *J* = 7.9, 6.3, 2.5 Hz, 1H)	4.21 (ddd, *J* = 8.0, 7.0, 3.0 Hz, 1H)	-0.02
H11	2.17 (dt, *J* = 14.4, 6.5 Hz, 1H)	2.13 (dt, *J* = 14.0, 7.0 Hz, 1H)	-0.04
H11'	1.90 (m, 1H)	1.92 (ddd, *J* = 14.0, 8.0, 7.0 Hz, 1H)	0.02
H10a	4.05 (t, *J* = 7.0 Hz, 1H)	4.05 (t, *J* = 7.0 Hz, 1H)	0.00
H9	4.29 (br s, 1H)	4.28 (br s, 1H)	-0.01
H8	1.90 (m, 2H)	1.90 (br s, 2H)	0.00
H7	4.35 (br s, 1H)	4.34 (br s, 1H)	-0.01
H6a	3.90 (ddd, *J* = 7.6, 6.2, 1.1 Hz, 1H)	3.90 (br dd, *J* = 7.5, 7.0 Hz, 1H)	0.00
H5	2.76 (dtd, *J* = 14.0, 7.8, 1.4 Hz, 1H)	2.74 (dt, *J* = 14.0, 7.0 Hz, 1H)	-0.02
H5'	2.55 (dt, *J* = 14.0, 6.9 Hz, 1H)	2.56 (ddd, *J* = 14.0, 7.5, 7.0 Hz, 1H)	0.01
H4	6.06 (dtd, *J* = 10.8, 7.5, 1.0 Hz, 1H)	6.05 (dt, *J* = 10.5, 7.0 Hz, 1H)	-0.01
H3	5.58 (ddt, *J* = 10.8, 2.5, 1.4 Hz, 1H)	5.57 (dd, *J* = 10.5, 2.0 Hz, 1H)	-0.01
H1	3.13 (d, *J* = 2.3 Hz, 1H)	3.12 (d, *J* = 2.0 Hz, 1H)	-0.01

*No ^{13}C NMR data is available for (*Z*)-ocellenyne.

Comparison of ^{1}H NMR data for synthetic enantiomer of Suzuki (*E*)-ocellenyne *ent*-(*E*)-5 vs natural (*E*)-ocellenyne

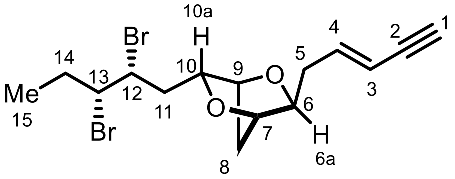

	Synthetic sample (S)	Natural sample (N)	
Proton	1H NMR (400 MHz, $CDCl_3$) / ppm	1H NMR (360 MHz, $CDCl_3$) / ppm	Δδ (N-S) / ppm
H15	1.07 (t, J = 7.2 Hz, 3H)	1.02 (t, J = 7.0 Hz, 3H)	-0.05
H14	2.09 (dqd, J = 14.5, 7.2, 3.7 Hz, 1H)	2.07 (dqd, J = 14.5, 7.0, 3.0 Hz, 1H)	-0.02
H14'	1.82 (m, 1H)	1.87 (ddq, J = 14.5, 9.5, 7.0 Hz, 1H)	0.05
H13	4.10 (ddd, J = 10.2, 3.7, 2.7 Hz, 1H)	4.12 (ddd, J = 9.5, 3.0, 2.5 Hz, 1H)	0.02
H12	4.41 (dt, J = 10.7, 2.5 Hz, 1H)	4.21 (ddd, J = 8.0, 6.5, 2.5 Hz, 1H)	-0.20
H11	1.95 (m, 1H)	2.15 (ddd, J = 14.5, 6.5, 6.5 Hz, 1H)	0.20
H11'	1.78 (m, 1H)	1.91 (ddd, J = 14.5, 8.0, 7.0 Hz, 1H)	0.13
H10a	4.06 (dd, J = 9.9, 3.4 Hz, 1H)	3.98 (dd, J = 7.0, 6.5 Hz, 1H)	-0.08
H9	4.32 (d, J = 2.0 Hz, 1H)	4.29 (br s, 1H)	-0.03
H8	1.92 (m, 1H)	1.90 (br s, 2H)	-0.02
H8'	1.87 (m, 1H)	1.90 (br s, 2H)	0.03
H7	4.36 (d, J = 2.5 Hz, 1H)	4.35 (br s, 1H)	-0.01
H6a	3.85 (td, J = 7.1, 1.1 Hz, 1H)	3.85 (br dd, J = 7.5, 7.0 Hz, 1H)	0.00
H5	2.48 (dtd, J = 14.2, 7.1, 1.7 Hz, 1H)	2.46 (ddd, J = 13.5, 7.5, 7.5 Hz, 1H)	-0.02
H5'	2.40 (dtd, J = 14.4, 7.3, 1.5 Hz, 1H)	2.41 (ddd, J = 13.5, 7.5, 7.0 Hz, 1H)	0.01
H4	6.23 (dt, J = 16.0, 7.3 Hz, 1H)	6.22 (ddd, J = 15.5, 7.5, 7.5 Hz, 1H)	-0.01
H3	5.60 (dq, J = 16.0, 1.6 Hz, 1H)	5.59 (dd, J = 15.5, 1.5 Hz, 1H)	-0.01
H1	2.81 (d, J = 2.2 Hz, 1H)	2.80 (d, J = 1.5 Hz, 1H)	-0.01

Comparison of ^{13}C NMR data for synthetic enantiomer of Suzuki (*E*)-ocellenyne *ent*-(*E*)-5 vs natural (*E*)-ocellenyne

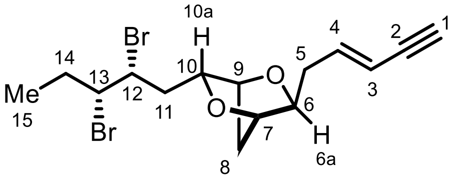

	Synthetic sample (S)	Natural sample[a] (N)	
Carbon	^{13}C NMR (151 MHz, $CDCl_3$) / ppm	^{13}C NMR (25 MHz, $CDCl_3$) / ppm	Δδ (N-S) / ppm
C15	12.7	12.9	0.2
C14	29.1	29.4	0.3
C13	62.2	60.7	-1.5
C12	54.6	53.5	-1.1
C11	39.9	40.2	0.3
C10	81.7	81.8[b]	0.1
C9	80.0	79.3	-0.7
C8	35.4	35.0	-0.4
C7	77.2	78.2	1.0
C6	81.8	81.8[b]	0.0
C5	34.6	34.7	0.1
C4	142.0	141.7	-0.3
C3	111.5	111.6	0.1
C2	82.3	81.8[b]	-0.5
C1	76.6	76.6	0.0

[a] carbon resonances are not assigned in reference[4]. [b] three ^{13}C NMR resonances are all stated as being at 81.8 ppm in the isolation paper; since the C2 quaternary carbon resonance is of lower signal intensity than the non-quaternary resonances it is likely that this resonance was not observed when recording data on a less powerful 25MHz ^{13}C NMR probe.

Comparison of ^{1}H NMR data for synthetic enantiomer of Suzuki (*Z*)-ocellenyne *ent*-(*Z*)-5 vs natural (*Z*)-ocellenyne*

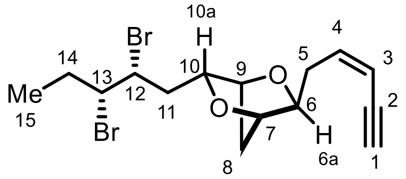

	Synthetic sample (S)	Natural sample (N)	
Proton	^{1}H NMR (400 MHz, $CDCl_3$) / ppm	^{1}H NMR (100 MHz, $CDCl_3$) / ppm	Δδ (N-S) / ppm
H15	1.07 (t, J = 7.3 Hz, 3H)	1.07 (t, J = 7.0 Hz, 3H)	0.00
H14	2.08 (dtd, J = 14.5, 7.2, 3.7 Hz, 1H)	2.05 (dqd, J = 13.0, 7.0, 3.0 Hz, 1H)	-0.03
H14'	1.83 (m, 1H)	1.85 (ddq, J = 13.0, 9.0, 7.0 Hz, 1H)	0.02
H13	4.10 (dt, J = 10.0, 3.2 Hz, 1H)	4.10 (ddd, J = 9.0, 3.0, 3.0 Hz, 1H)	0.00
H12	4.43 (dt, J = 10.7, 2.6 Hz, 1H)	4.21 (ddd, J = 8.0, 7.0, 3.0 Hz, 1H)	-0.22
H11	1.96 (m, 1H)	2.13 (dt, J = 14.0, 7.0 Hz, 1H)	0.17
H11'	1.78 (m, 1H)	1.92 (ddd, J = 14.0, 8.0, 7.0 Hz, 1H)	0.14
H10a	4.14 (dd, J = 10.0, 3.2 Hz, 1H)	4.05 (t, J = 7.0 Hz, 1H)	-0.09
H9	4.33 (d, J = 2.3 Hz, 1H)	4.28 (br s, 1H)	-0.05
H8	1.93 (m, 1H)	1.90 (br s, 2H)	-0.03
H8'	1.87 (dd, J = 9.4, 2.6 Hz, 1H)	1.90 (br s, 2H)	0.03
H7	4.36 (d, J = 2.1 Hz, 1H)	4.34 (br s, 1H)	-0.02
H6a	3.90 (ddd, J = 7.8, 6.2, 1.1 Hz, 1H)	3.90 (br dd, J = 7.5, 7.0 Hz, 1H)	0.00
H5	2.78 (dtd, J = 14.0, 7.9, 1.5 Hz, 1H)	2.74 (dt, J = 14.0, 7.0 Hz, 1H)	-0.04
H5'	2.57 (dddd, J = 14.0, 7.5, 6.1, 1.3 Hz, 1H)	2.56 (ddd, J = 14.0, 7.5, 7.0 Hz, 1H)	-0.01
H4	6.07 (dtd, J = 11.0, 7.5, 0.9 Hz, 1H)	6.05 (dt, J = 10.5, 7.0 Hz, 1H)	-0.02
H3	5.58 (ddt, J = 10.8, 2.6, 1.4 Hz, 1H)	5.57 (dd, J = 10.5, 2.0 Hz, 1H)	-0.01
H1	3.13 (d, J = 2.1 Hz, 1H)	3.12 (d, J = 2.0 Hz, 1H)	-0.01

*No ^{13}C NMR data is available for (*Z*)-ocellenyne.

5. Comparison of ^{1}H NMR Spectra for Synthetic vs Simulated Natural Ocellenyne

We have been unable to obtain the original ^{1}H and ^{13}C NMR spectra of the natural ocellenynes for comparison with the NMR spectra of our synthetic ocellenynes. *In lieu* of this, we have simulated the ^{1}H NMR spectra of natural (*E*)-and (*Z*)-ocellenyne, using the MestreNova Spin Simulation function, from the available chemical shift and *J*-coupling data reported in the isolation paper.[4] The simulated spectra (lower spectra in red in the figures below) are compared with the ^{1}H NMR data for our synthetic (*E*)-8 and (*Z*)-8 as well as with our synthetic ^{1}H NMR data for the enantiomers of the Suzuki ocellenynes *ent*-(*E*)-5 and *ent*-(*Z*)-5. It is clear from these spectra that our synthetic enantiomers of the Suzuki ocellenynes *ent*-(*E*)-5 and *ent*-(*Z*)-5 do not match those of the natural ocellenynes. The ^{1}H NMR spectrum for our synthetic (*E*)-8 is an excellent match for simulated spectrum natural (*E*)-8 in terms of chemical shift and line shape apart from the chemical shift of the C-15 methyl group which is different by 0.06 ppm (in $CDCl_3$). There is also a good match between the simulated ^{1}H NMR spectrum of (*Z*)-8 with that of natural (*Z*)-ocellenyne.

Comparison of synthetic (*E*)-ocellenyne (*E*)-8 ^{1}H NMR in $CDCl_3$ at 400 MHz (top spectrum) with natural (*E*)-ocellenyne ^{1}H NMR simulated at 400 MHz, line width = 1.5 Hz (bottom spectrum)

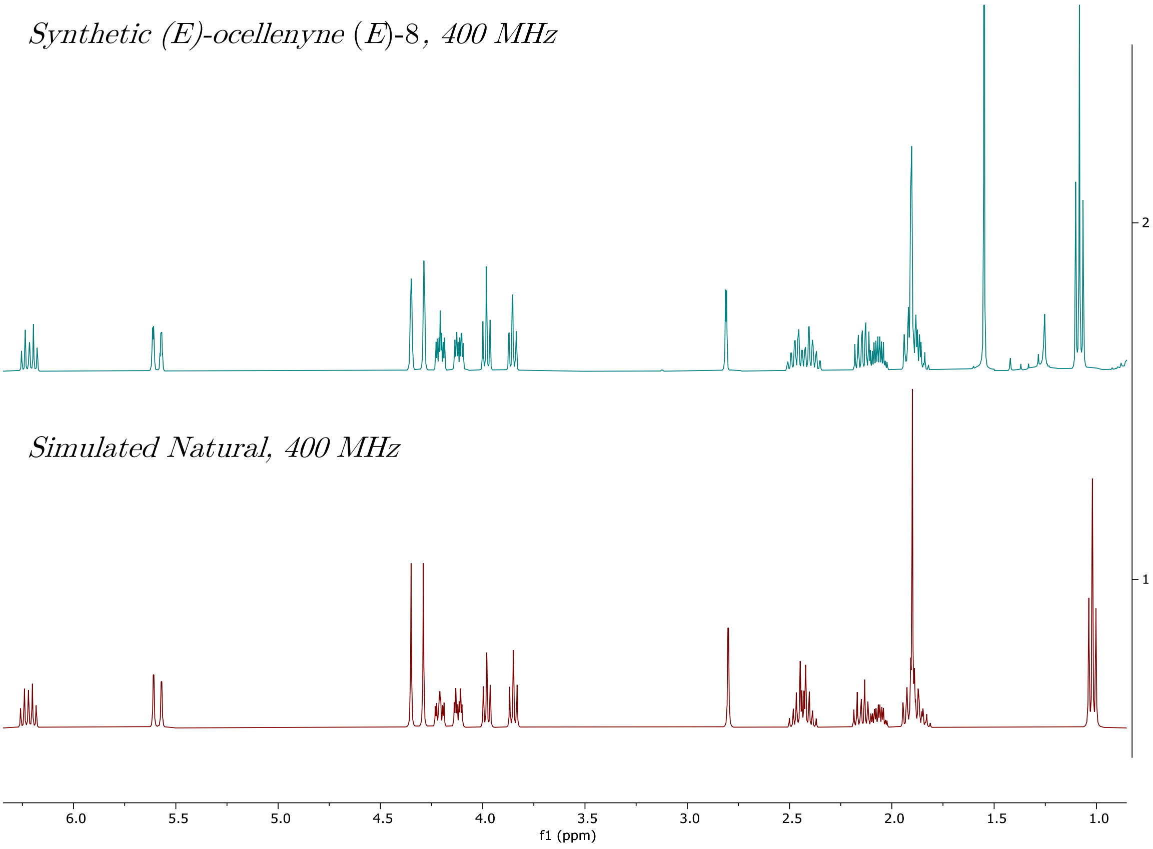

Comparison of synthetic (*E*)-ocellenyne (*E*)-8 ^{1}H NMR in $CDCl_3$ at 600 MHz (top spectrum) with natural (*E*)-ocellenyne ^{1}H NMR simulated at 600 MHz, line width = 1.5 Hz (bottom spectrum)

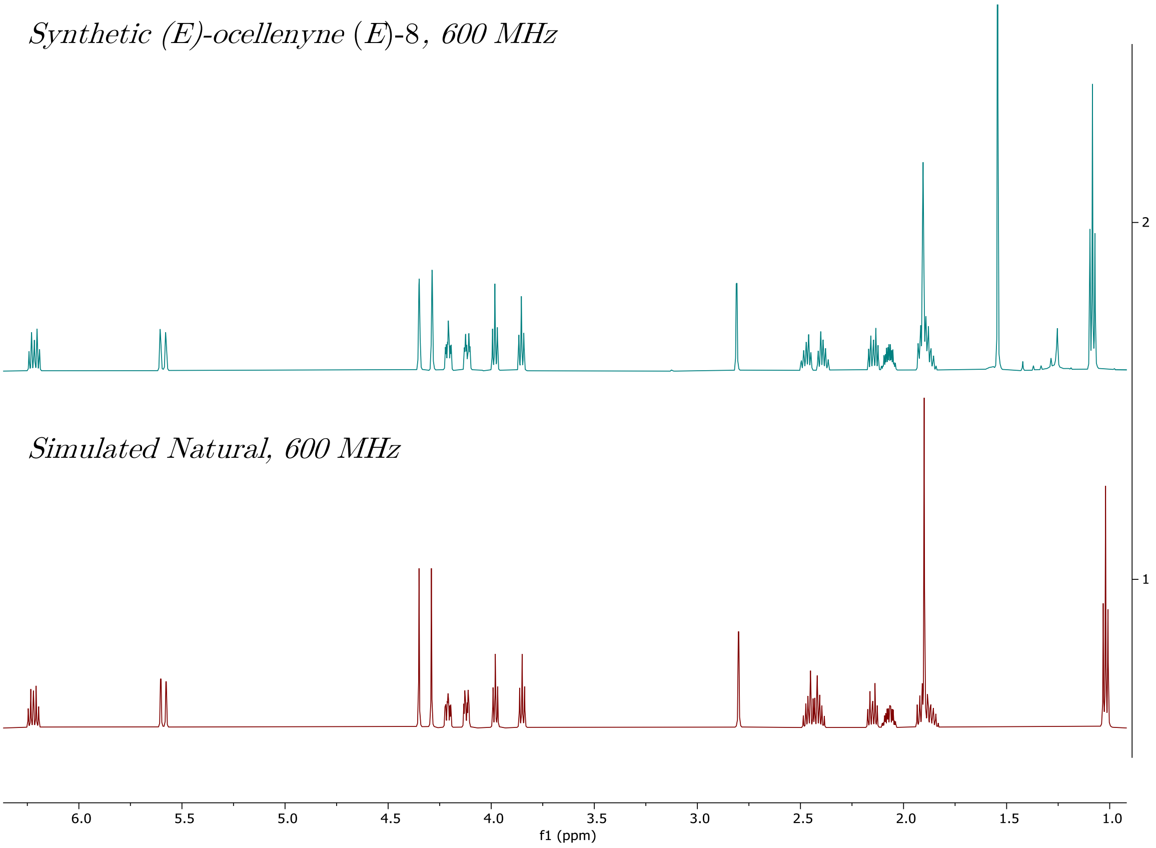

Comparison of synthetic enantiomer of Suzuki (*E*)-ocellenyne *ent*-(*E*)-5 ^{1}H NMR in $CDCl_3$ at 400 MHz (top spectrum) with natural (*E*)-ocellenyne ^{1}H NMR simulated at 400 MHz, line width = 1.5 Hz (bottom spectrum)

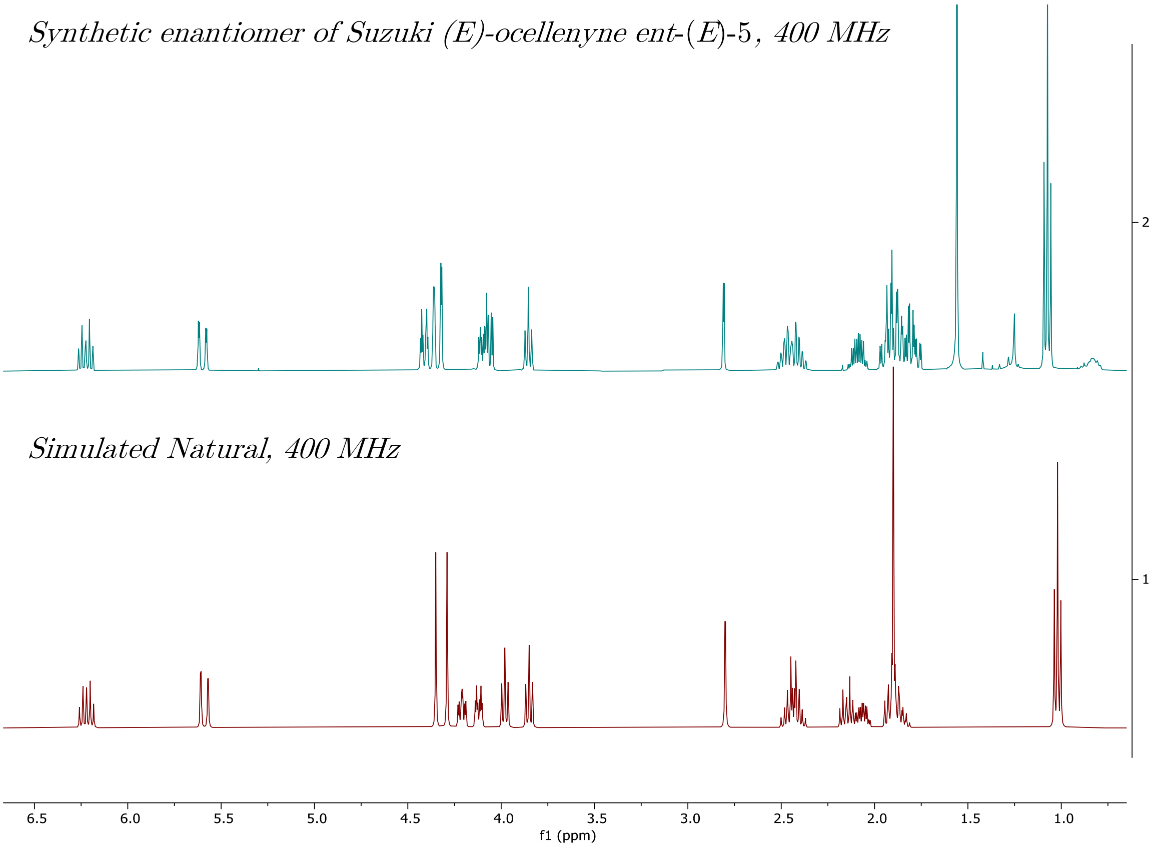

Comparison of synthetic (*Z*)-ocellenyne (*Z*)-8 ^{1}H NMR in $CDCl_3$ at 400 MHz (top spectrum) with natural (*Z*)-ocellenyne ^{1}H NMR simulated at 400 MHz, line width = 1.5 Hz (bottom spectrum)

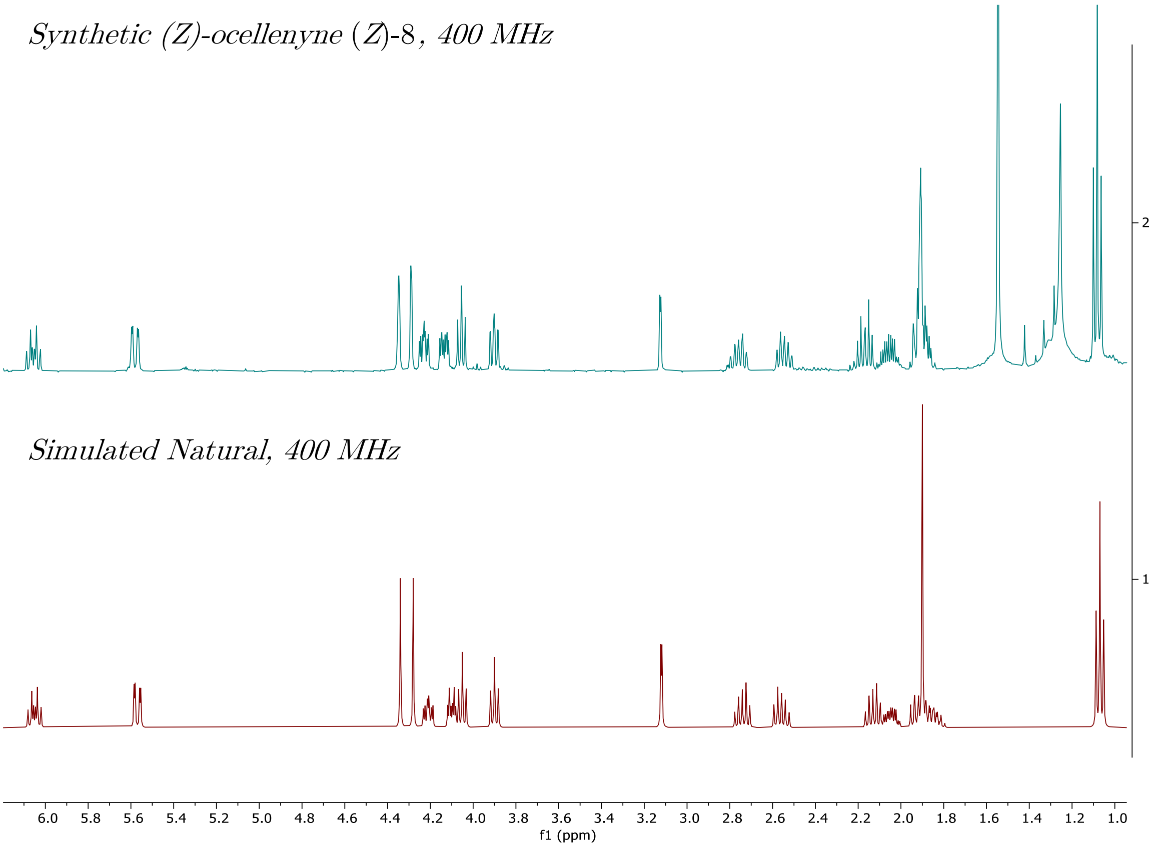

Comparison of synthetic (*Z*)-ocellenyne (*Z*)-8 ^{1}H NMR in $CDCl_3$ at 600 MHz (top spectrum) with natural (*Z*)-ocellenyne ^{1}H NMR simulated at 600 MHz, line width = 1.5 Hz (bottom spectrum)

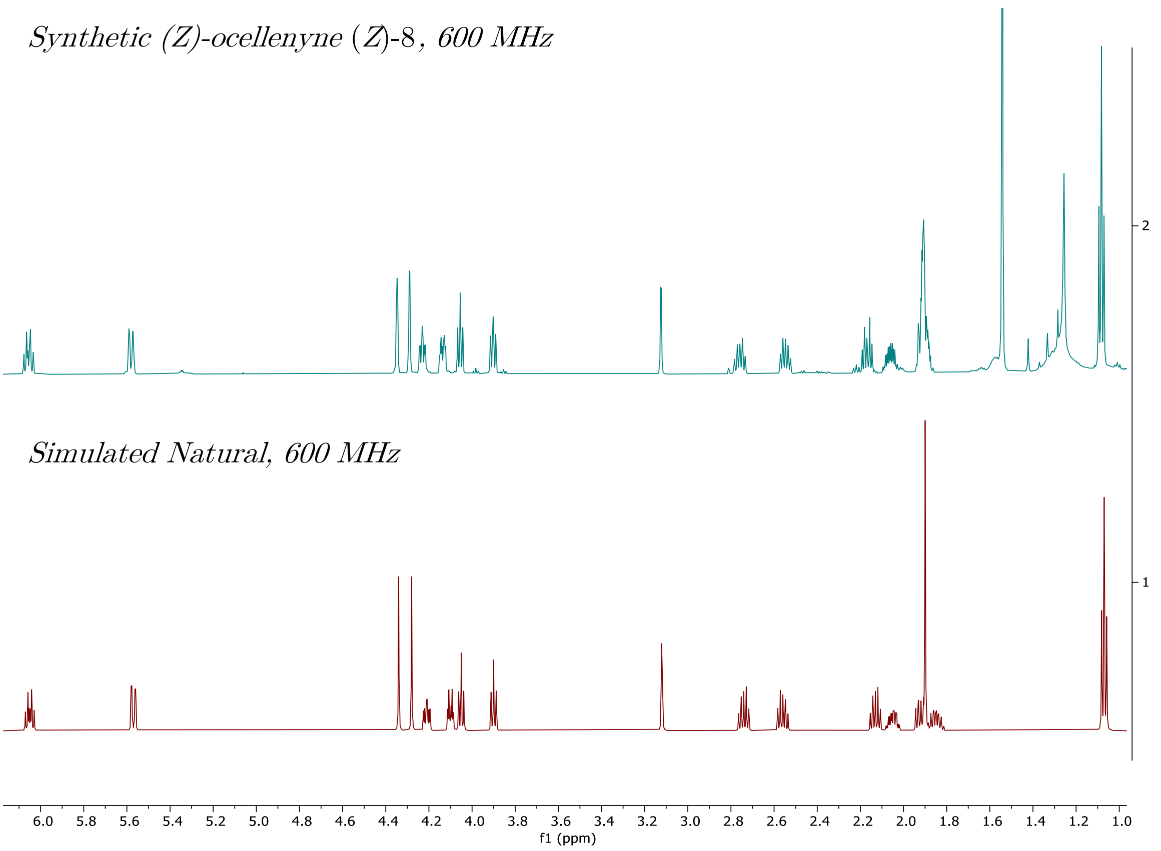

Comparison of synthetic enantiomer of Suzuki (*Z*)-ocellenyne *ent*-(*Z*)-5 ^{1}H NMR in $CDCl_3$ at 400 MHz (top spectrum) with natural (*Z*)-ocellenyne ^{1}H NMR simulated at 400 MHz, line width = 1.5 Hz (bottom spectrum)

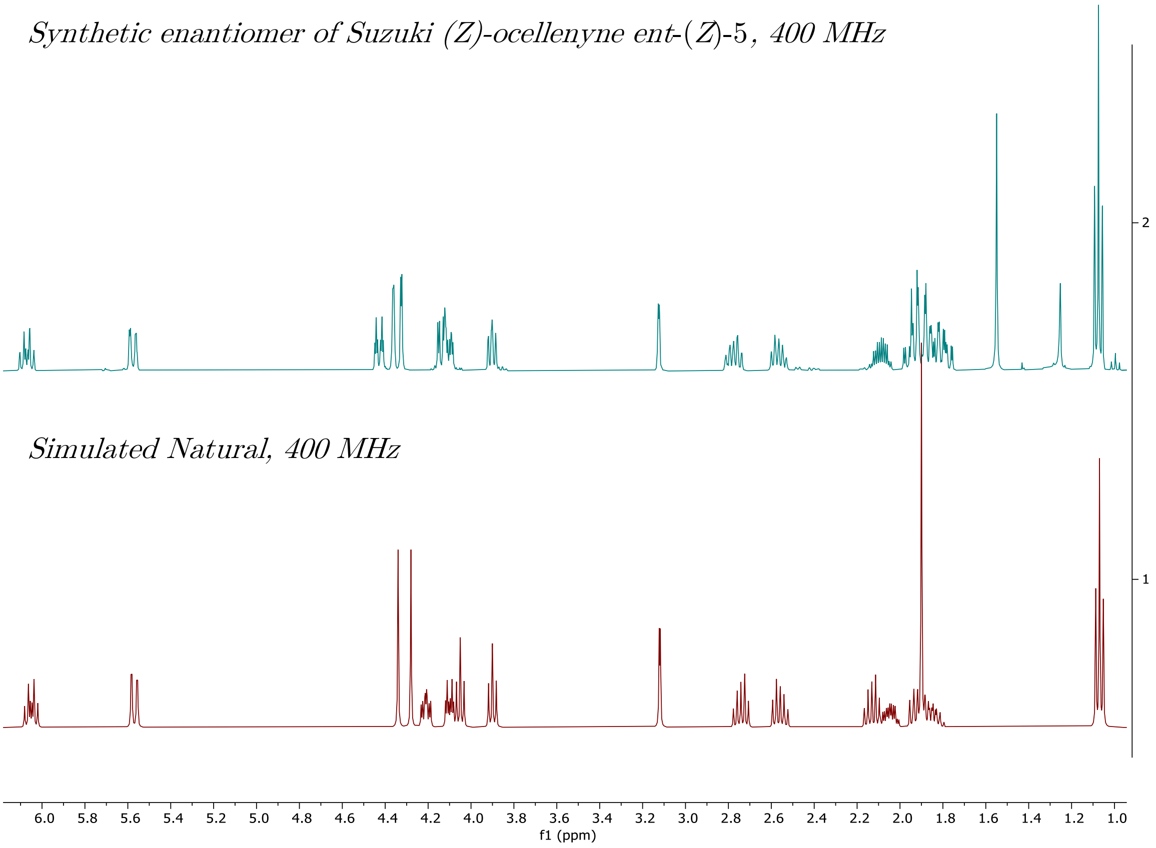

6. References

[1] J. Cosier, A. M. Glazer, *J. Appl. Crystallogr.* 1986, *19*, 105-107.

[2] L. Palatinus, G. Chapuis, *J. Appl. Crystallogr.* 2007, *40*, 786-790.

[3] a. P. W. Betteridge, J. R. Carruthers, R. I. Cooper, K. Prout, D. J. Watkin, *J. Appl. Crystallogr.* 2003, *36*, 1487; b. P. Parois, R. I. Cooper, A. L. Thompson, *Chemistry Central Journal* 2015, *9*, 30; c. R. I. Cooper, A. L. Thompson, D. J. Watkin, *J. Appl. Crystallogr.* 2010, *43*, 1100-1107.

[4] G. R. Schulte, M. C. H. Chung, P. J. Scheuer, *J. Org. Chem.* 1981, *46*, 3870-3873.

[5] H. S. S. Chan, Q. N. N. Nguyen, R. S. Paton, J. W. Burton, *J. Am. Chem. Soc.* 2019, *141*, 15951-15962.

[6] a. T. Hanaya, K.-i. Sugiyama, H. Kawamoto, H. Yamamoto, *Carbohydr. Res.* 2003, *338*, 1641-1650; b. Hau Sun (Sam) Chan, DPhil-Thesis, Wolfson College, University of Oxford 2019.

[7] B. H. Lipshutz, D. W. Chung, B. Rich, R. Corral, *Org. Lett.* 2006, *8*, 5069-5072.

[8] S. Das, C. V. Ramana, *Tetrahedron* 2015, *71*, 8577-8584.

[9] T. I. Richardson, S. D. Rychnovsky, *J. Org. Chem.* 1996, *61*, 4219-4231.

[10] a. A. R. Bassindale, T. Stout, *Tet. Lett.* 1984, *25*, 1631-1632; b. G. A. Olah, A. Husain, B. G. B. Gupta, G. F. Salem, S. C. Narang, *J. Org. Chem.* 1981, *46*, 5212-5214.

[11] W. Yu, Y. Mei, Y. Kang, Z. Hua, Z. Jin, *Org. Lett.* 2004, *6*, 3217-3219.

[12] Y. M. Ahn, K. Yang, G. I. Georg, *Org. Lett.* 2001, *3*, 1411-1413.

7. NMR Spectra for Novel Compounds

(*R*)-1-((3a*R*,5*S*,6a*R*)-2,2-Dimethyltetrahydrofuro[2,3-*d*][1,3]dioxol-5-yl)hex-3-yn-1-ol (10)

^{1}H NMR (600 MHz, CDCl$_3$)

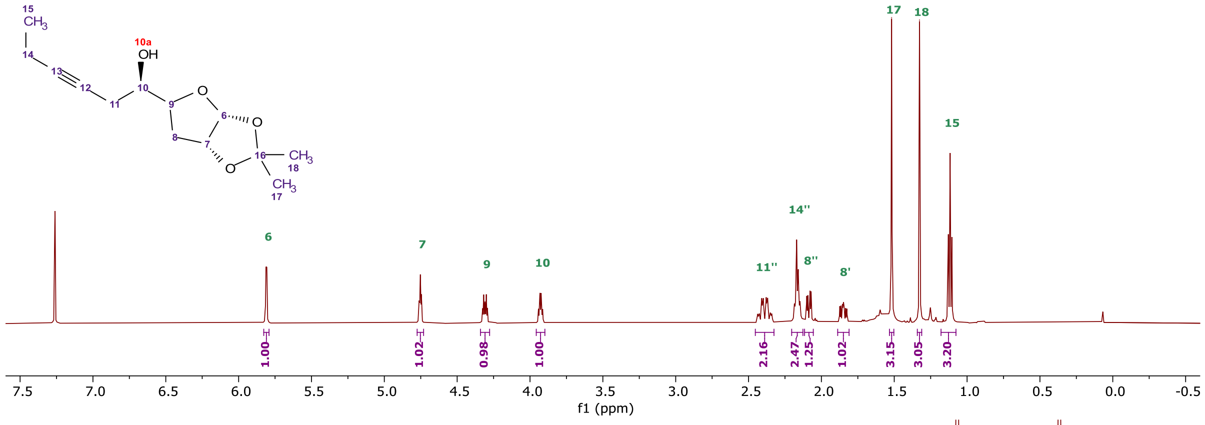

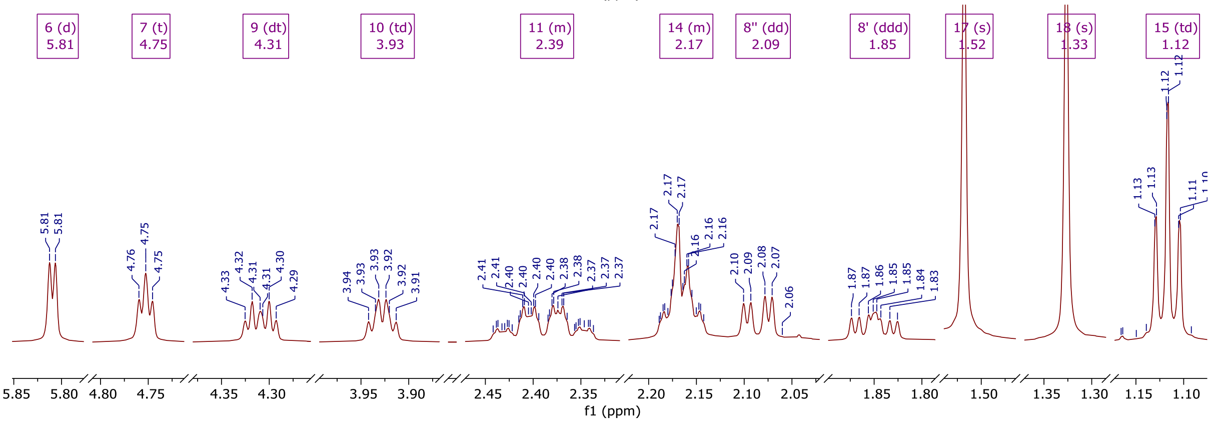

^{13}C NMR (151 MHz, CDCl$_3$)

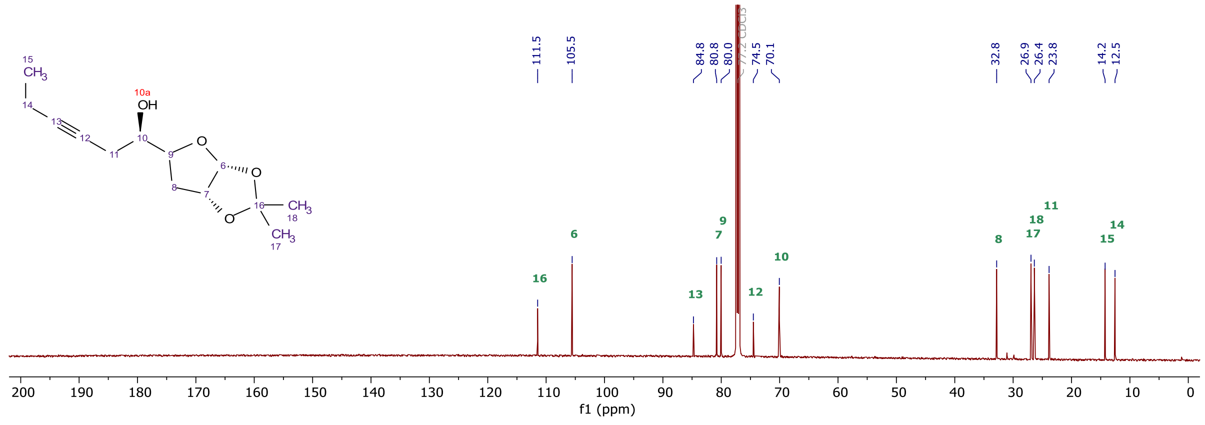

^{1}H-^{1}H COSY of 10

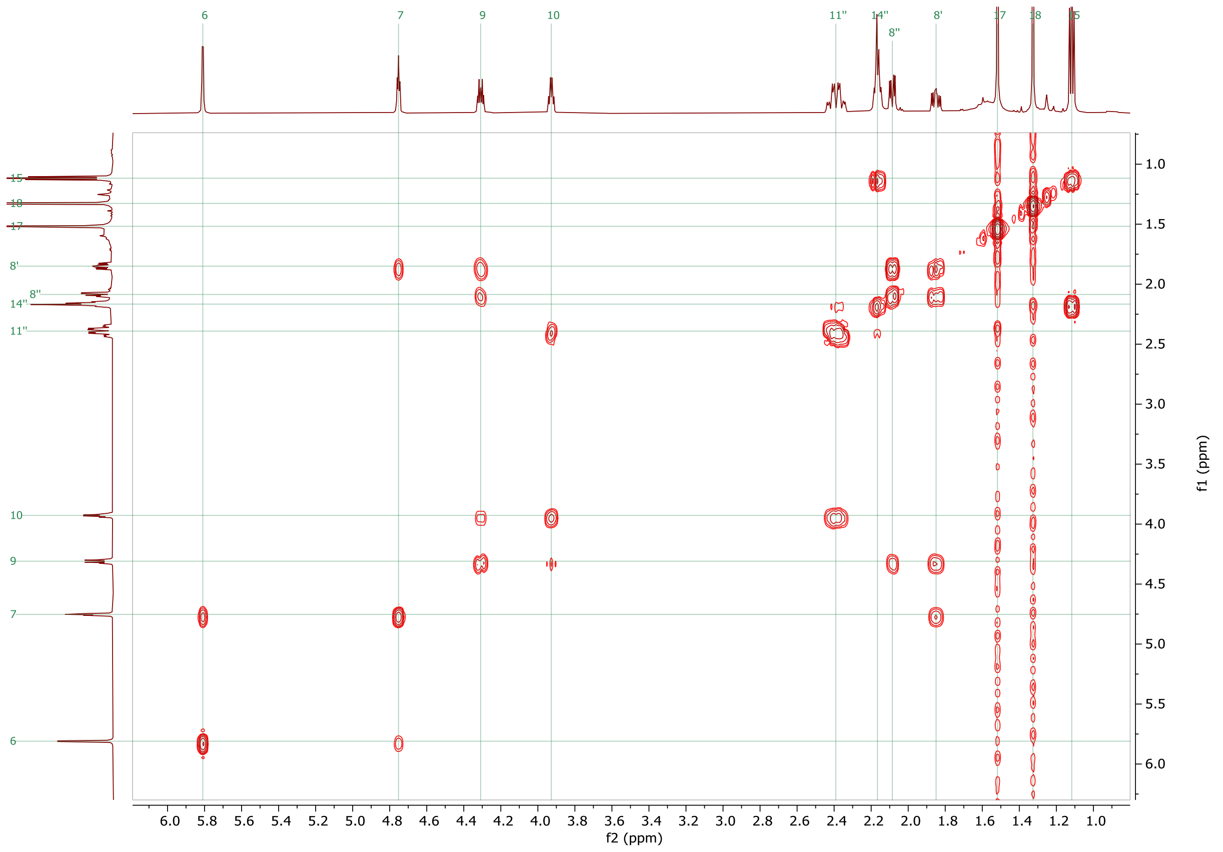

HSQC of 10

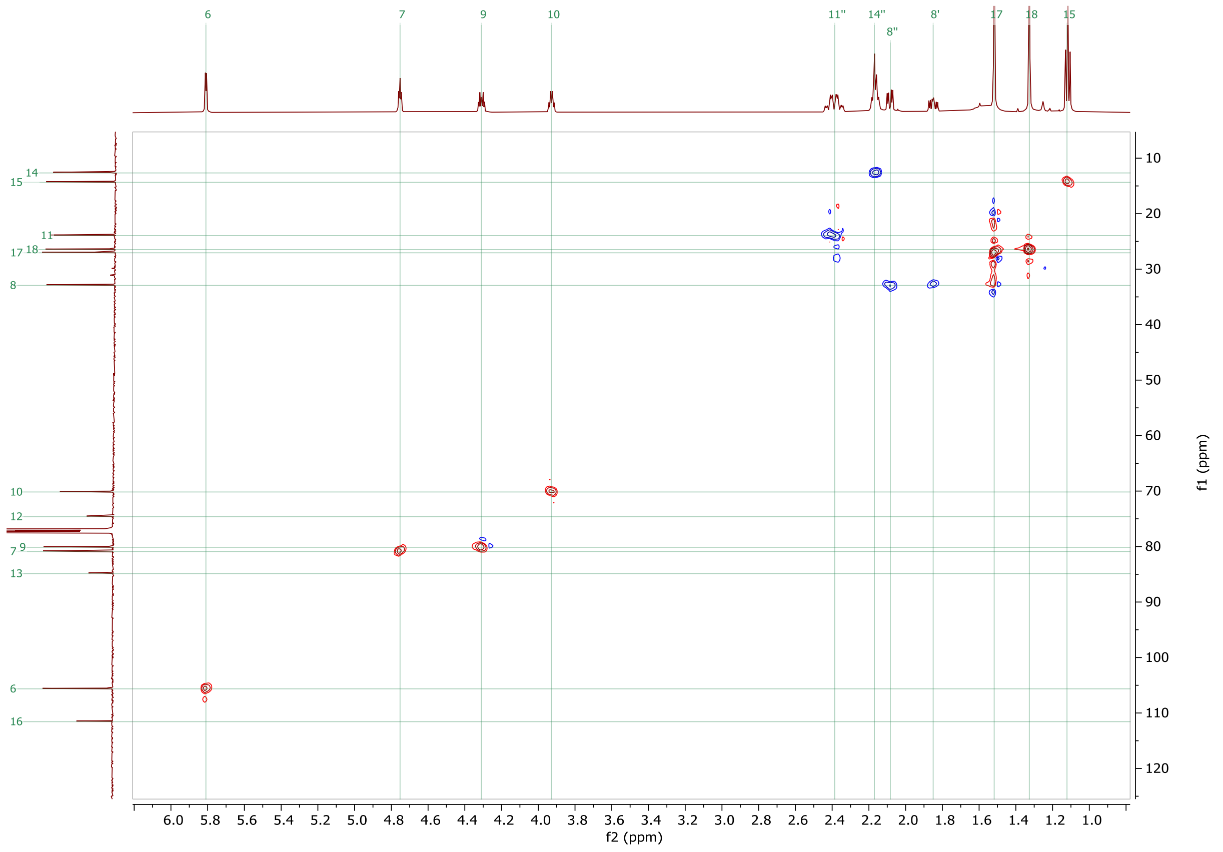

HMBC of 10

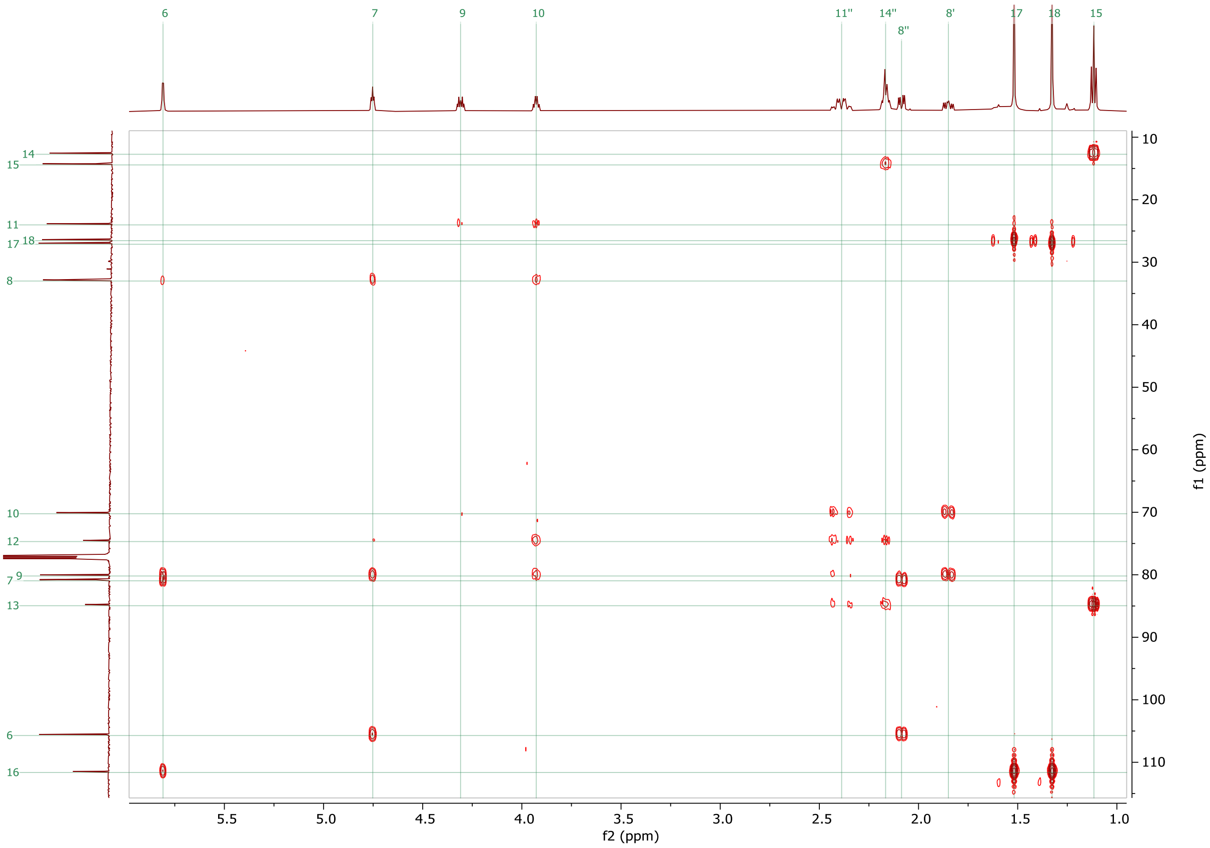

6
7
9
10
11''
14''
8''
8'
17
18
15
14
15
11
18
17
8
10
12
9
7
13
6
16
5.5
5.0
4.5
4.0
3.5
3.0
2.5
2.0
1.5
1.0
f2 (ppm)
10
20
30
40
50
60
70
80
90
100
110
f1 (ppm)

(*R*,*Z*)-1-((3a*R*,5*S*,6a*R*)-2,2-Dimethyltetrahydrofuro[2,3-*d*][1,3]dioxol-5-yl)hex-3-en-1-ol (S1)

^{1}H NMR (500 MHz, $CDCl_3$)

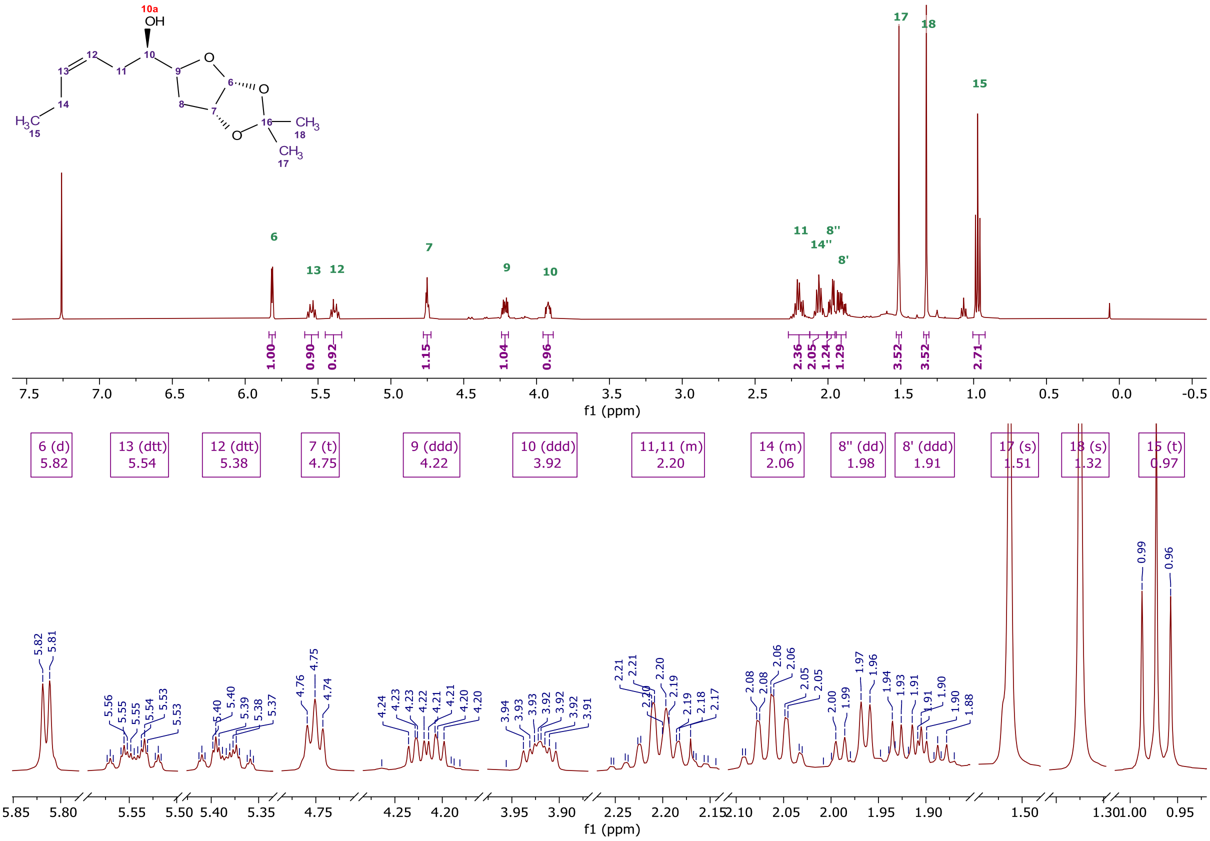

^{13}C NMR (126 MHz, $CDCl_3$)

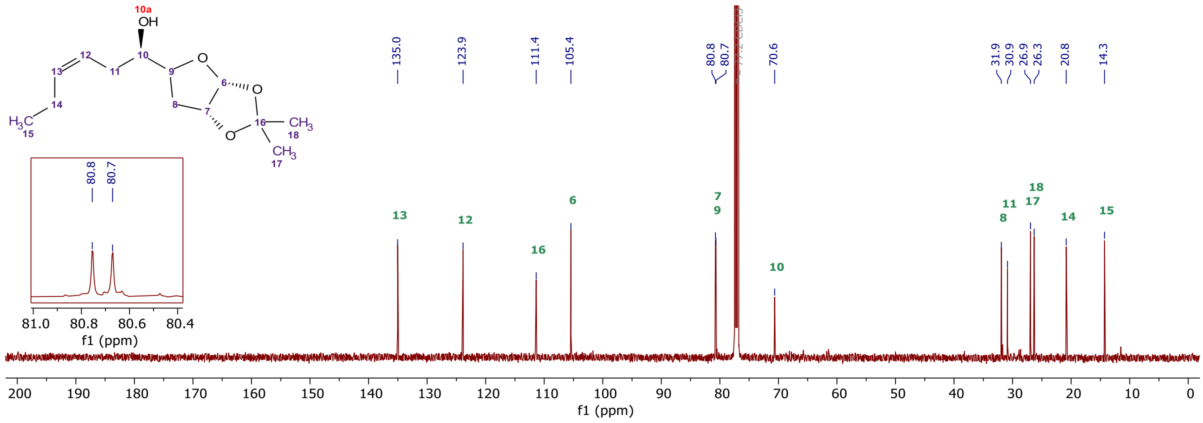

^1H-^1H COSY of S1

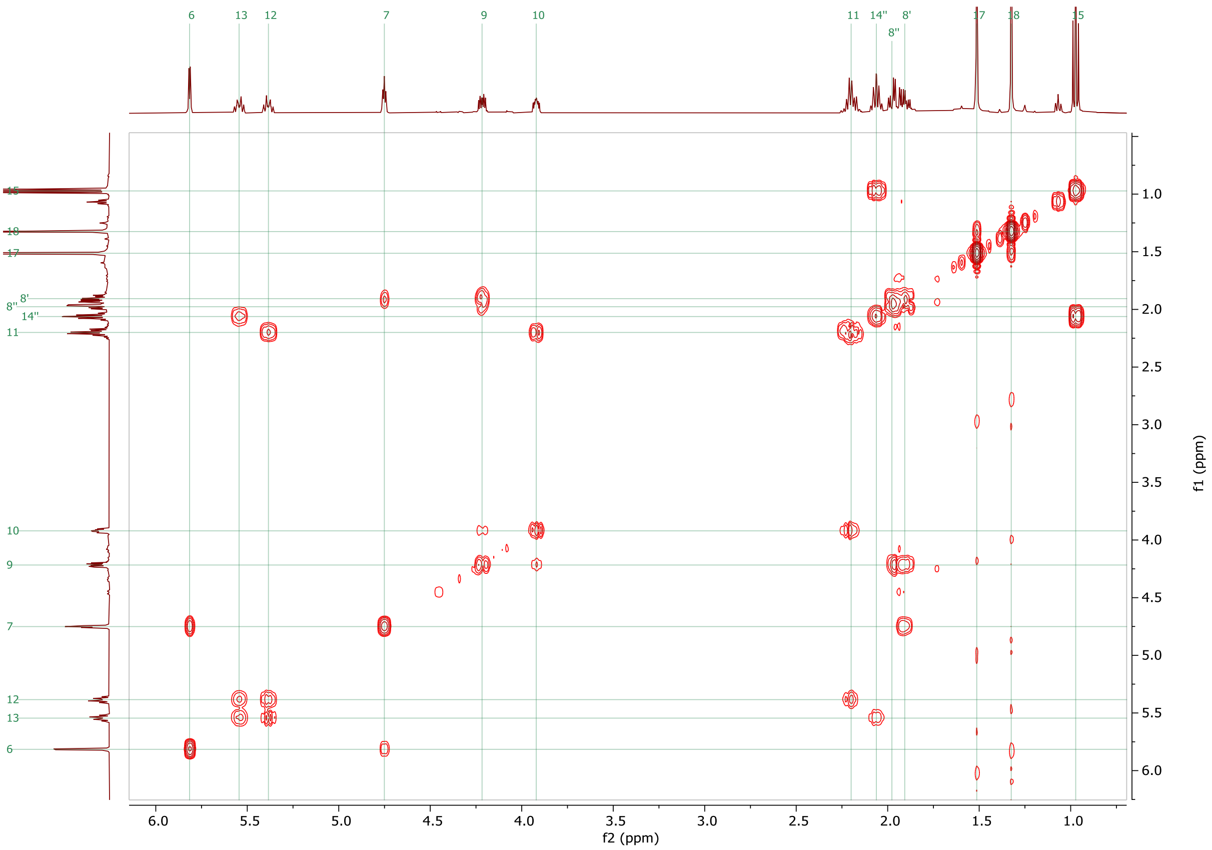

HSQC of S1

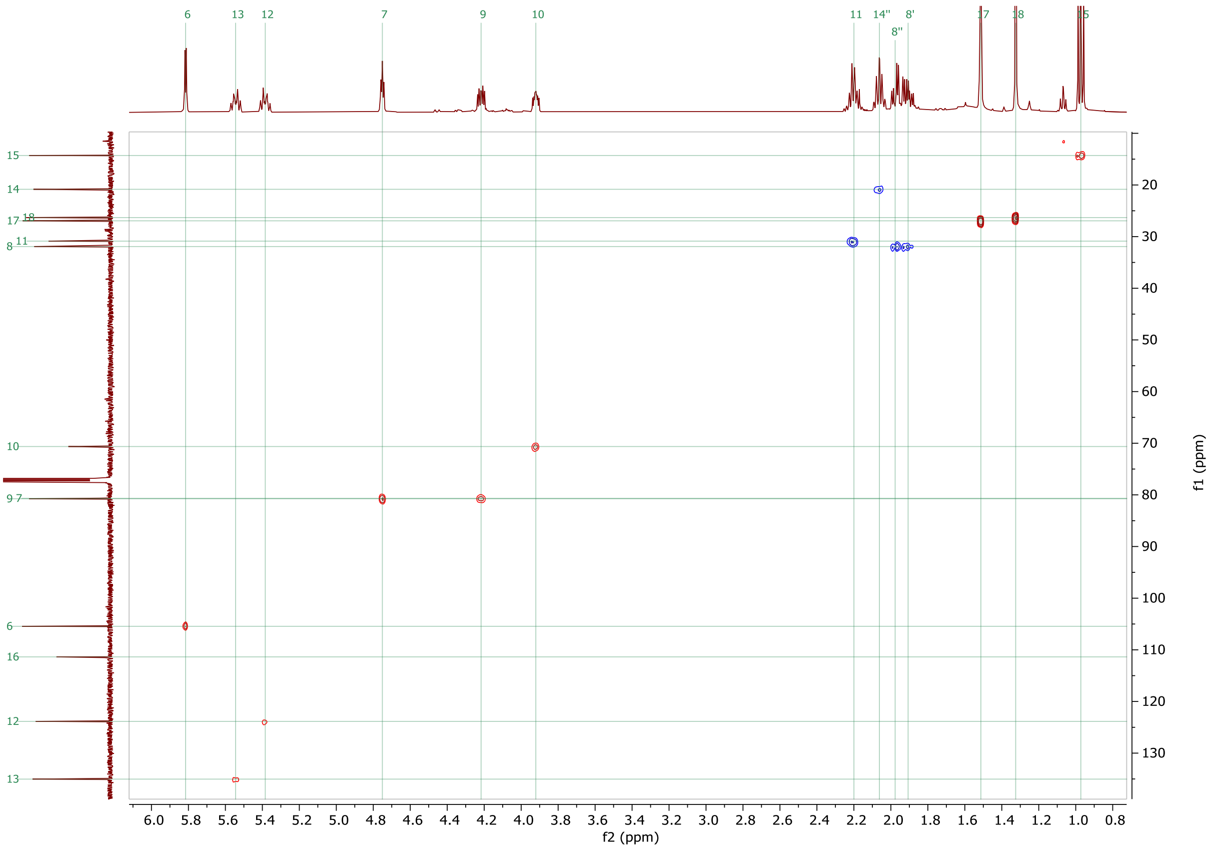

HMBC of S1

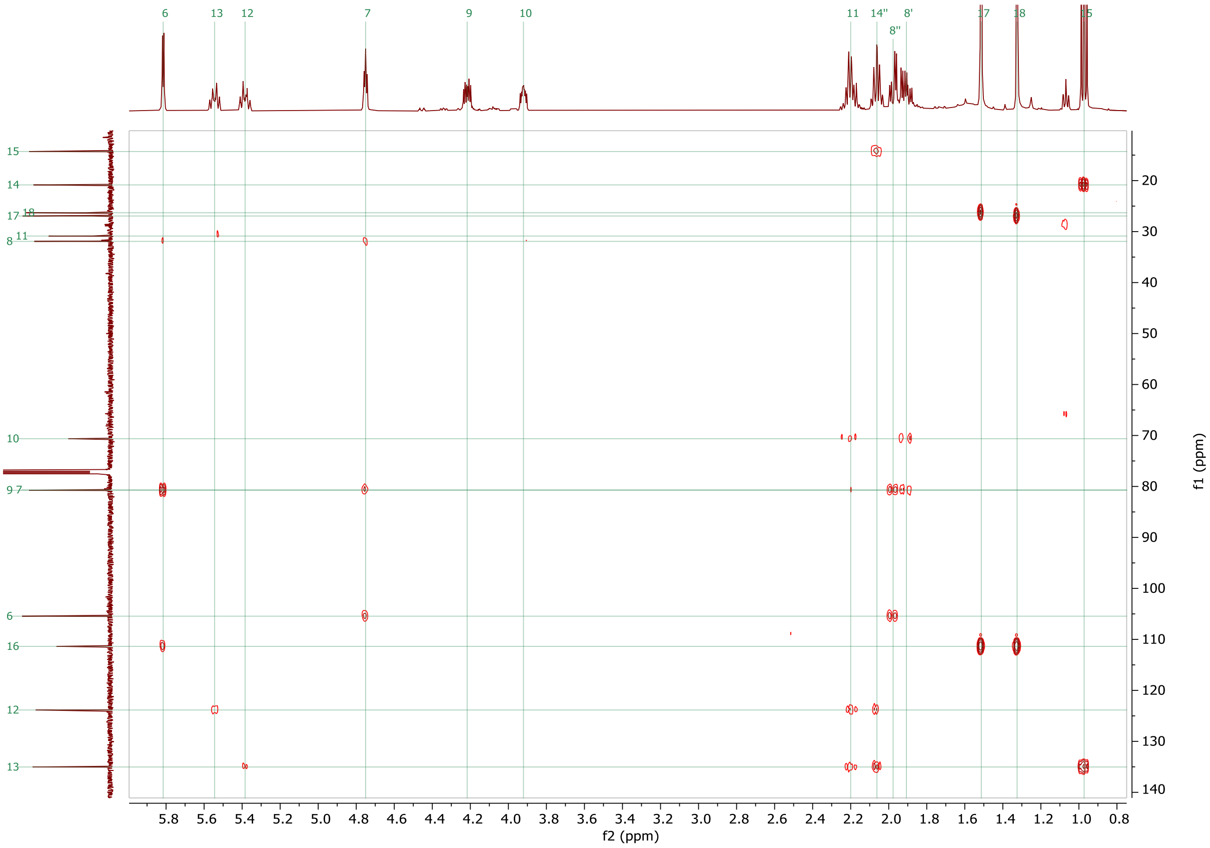

6
13
12
7
9
10
11
14''
8''
8'
17
18
15
15
14
17
18
11
8
10
9 7
6
16
12
13
f2 (ppm)
f1 (ppm)

(*R*,*Z*)-1-((3a*R*,5*S*,6a*R*)-2,2-Dimethyltetrahydrofuro[2,3-*d*][1,3]dioxol-5-yl)hex-3-en-1-yl acetate (11)

^{1}H NMR (500 MHz, $CDCl_3$)

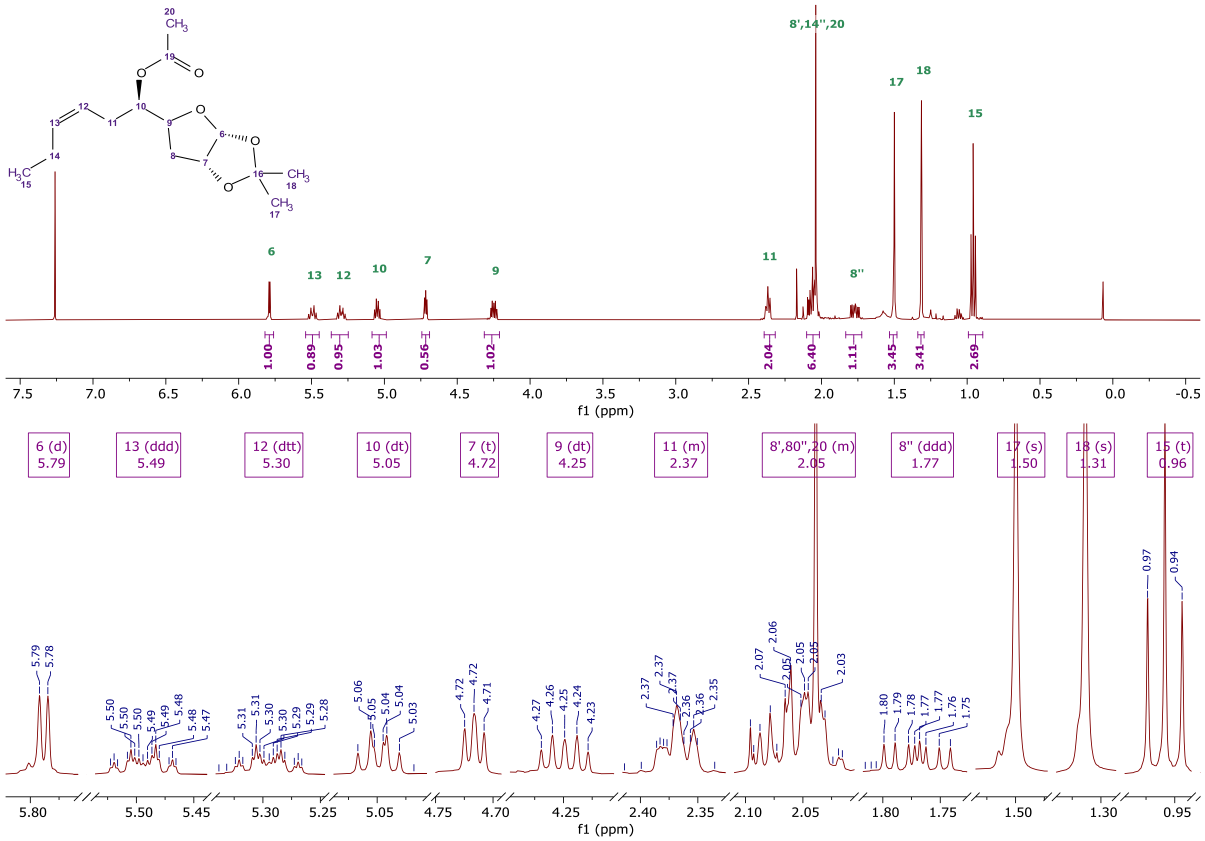

^{13}C NMR (126 MHz, $CDCl_3$)

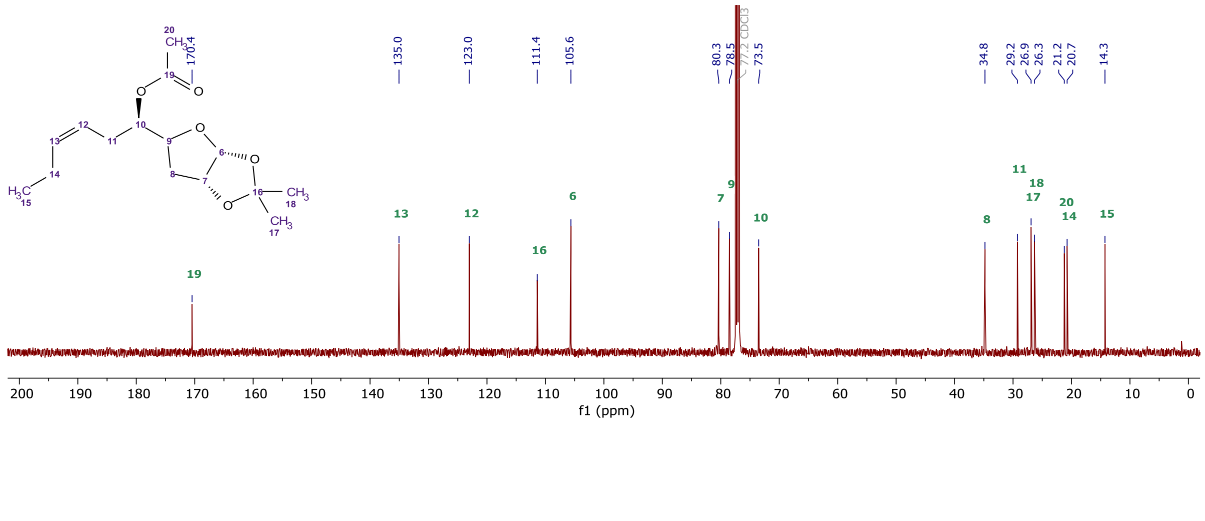

^{1}H-^{1}H COSY of 11

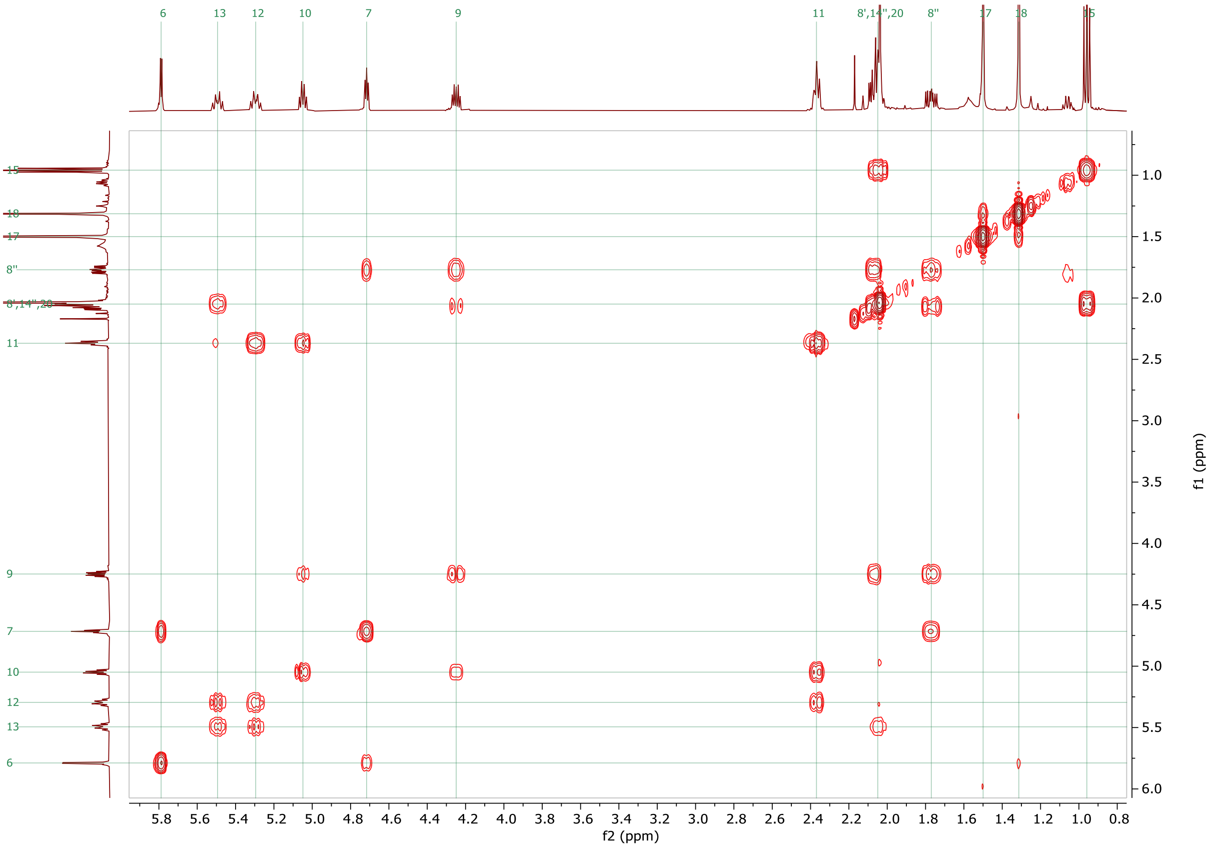

HSQC of 11

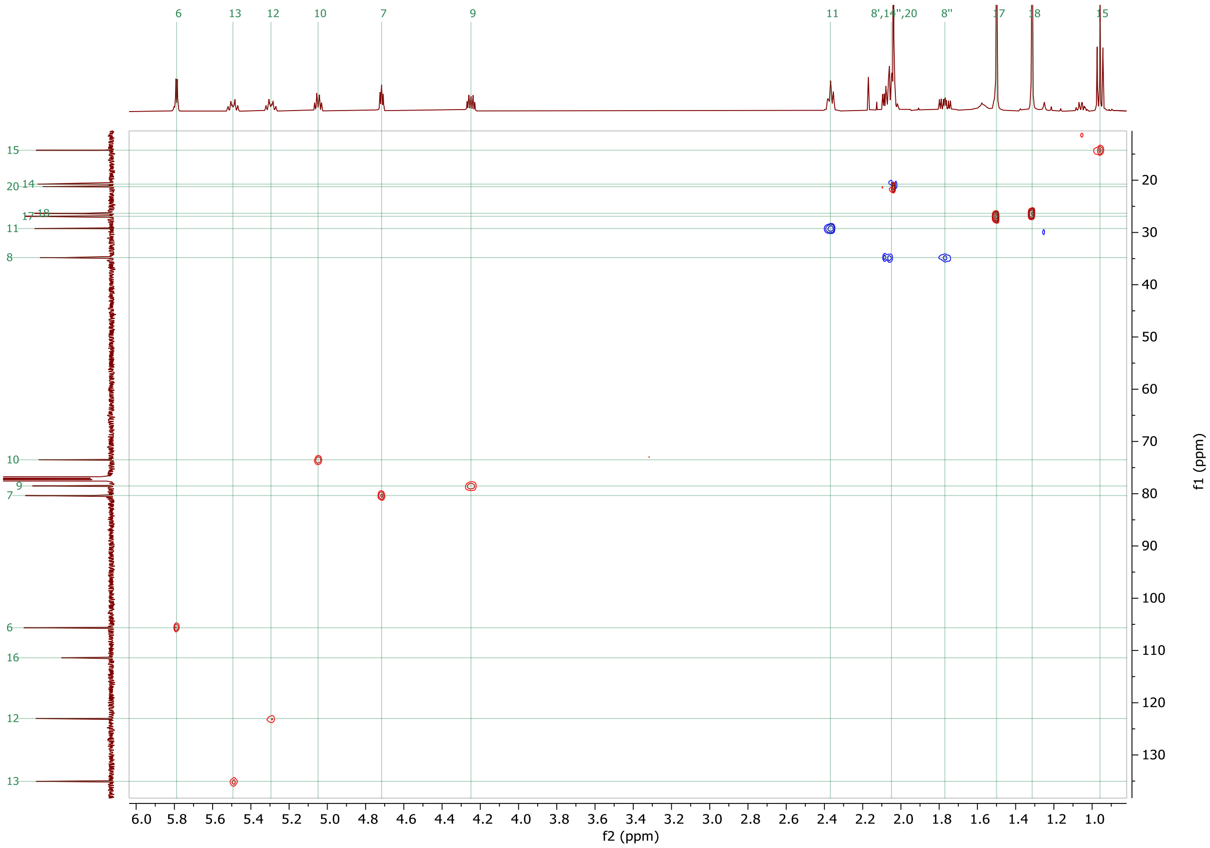

HMBC of 11

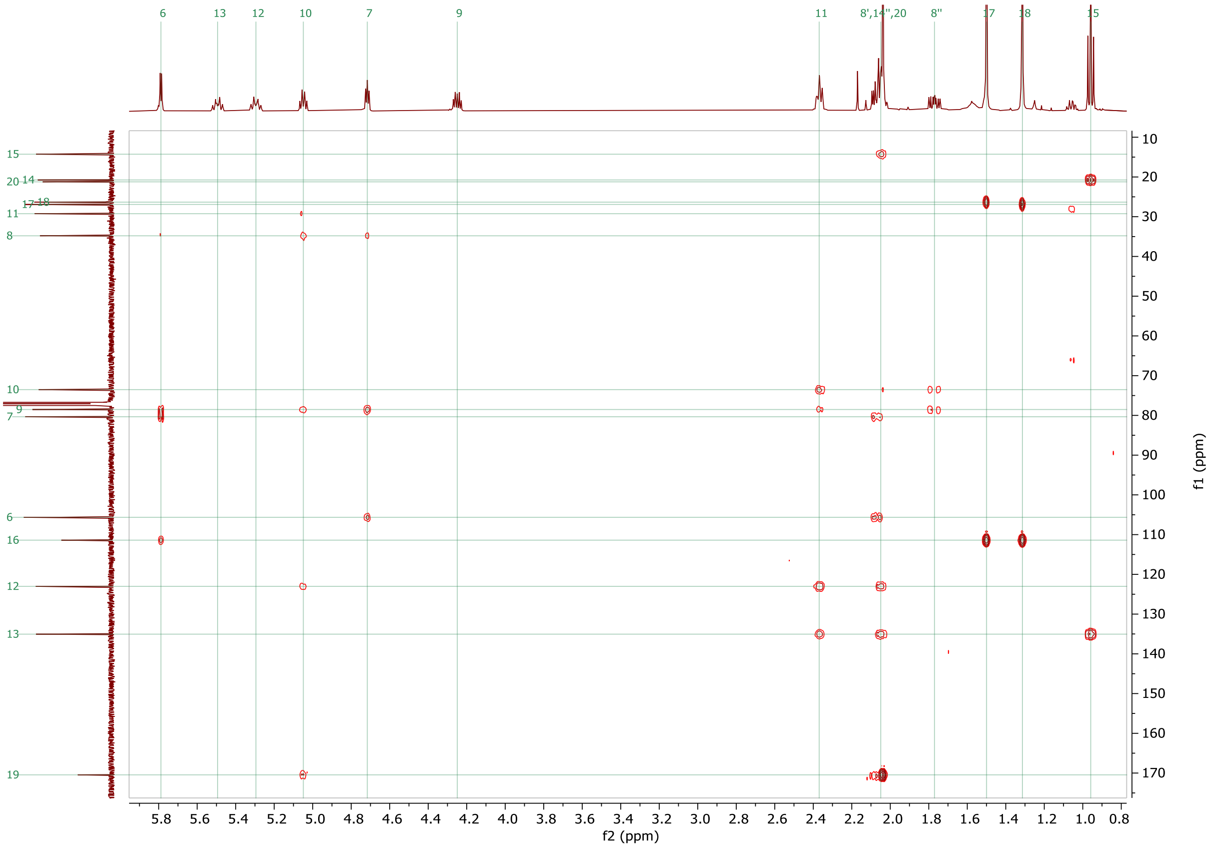

6
13
12
10
7
9
11
8',14'',20
8''
17
18
15
15
20
14
17
18
11
8
10
9
7
6
16
12
13
19
f2 (ppm)
f1 (ppm)

(R,Z)-1-((2S,4R,5S)-5-Allyl-4-hydroxytetrahydrofuran-2-yl)hex-3-en-1-yl acetate (12)

^{1}H NMR (500 MHz, CDCl$_3$)

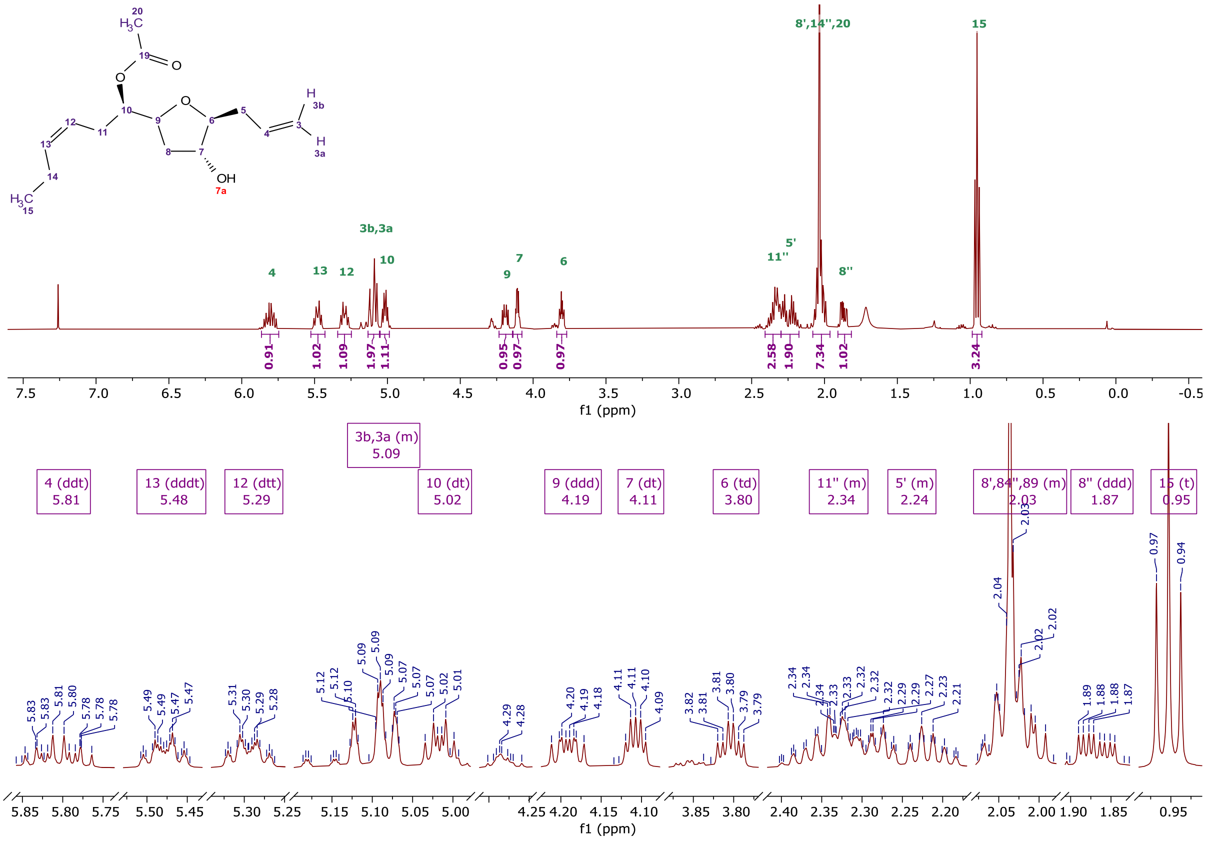

^{13}C NMR (126 MHz, CDCl$_3$)

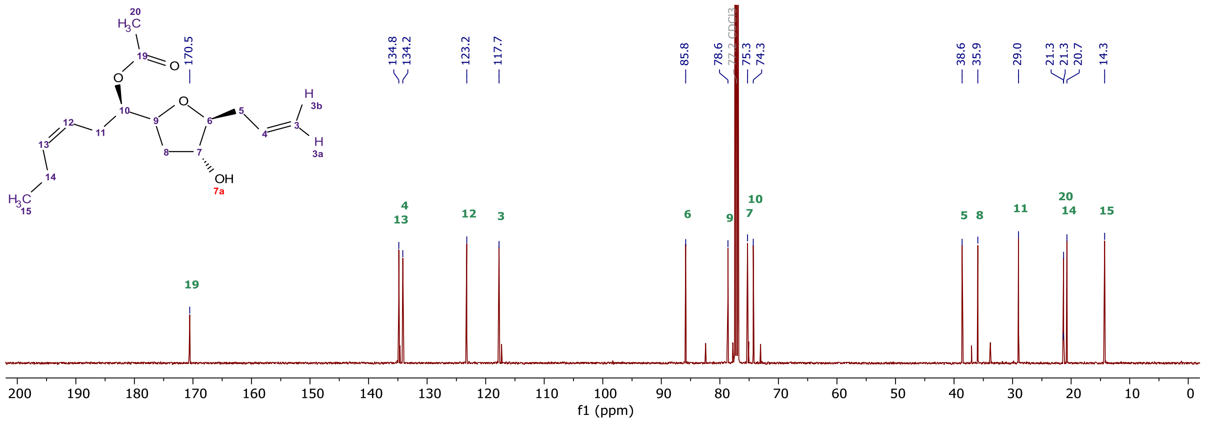

^{1}H-^{1}H COSY of 12

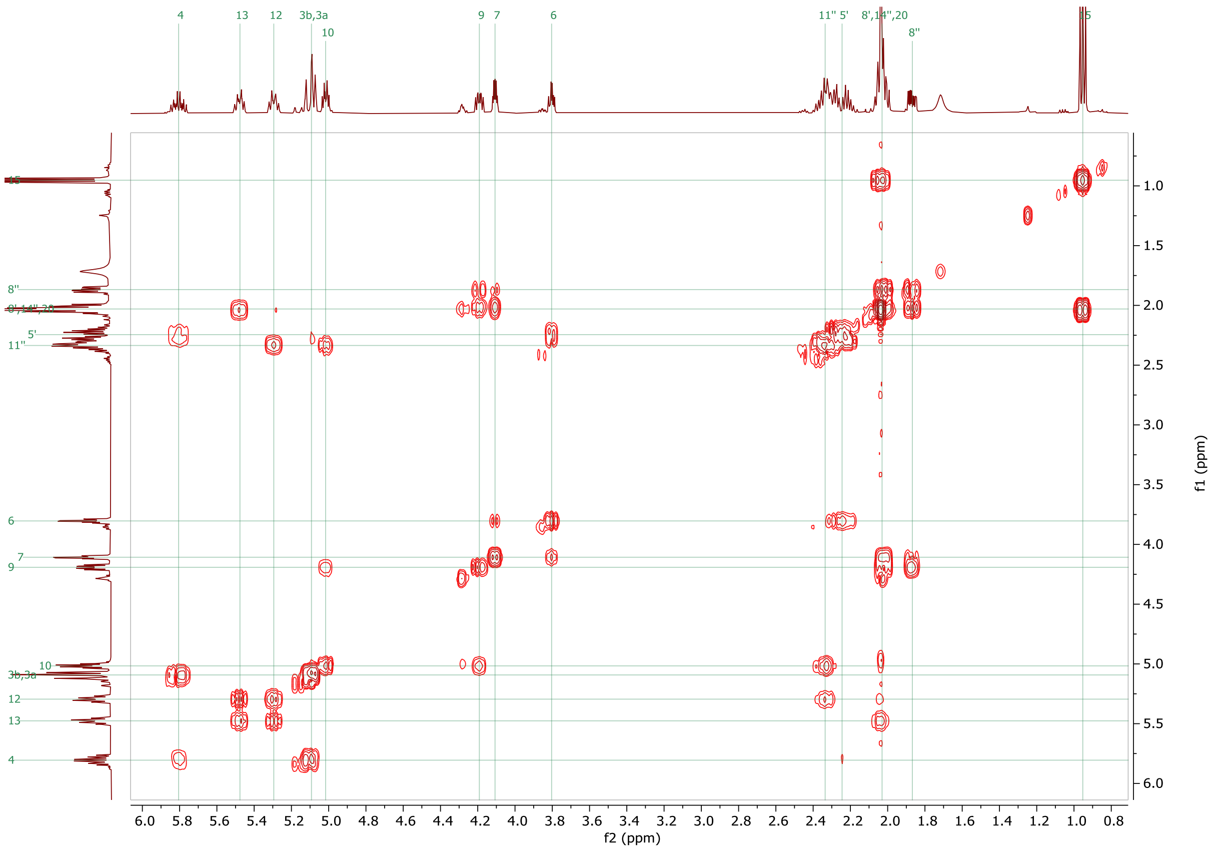

HSQC of 12

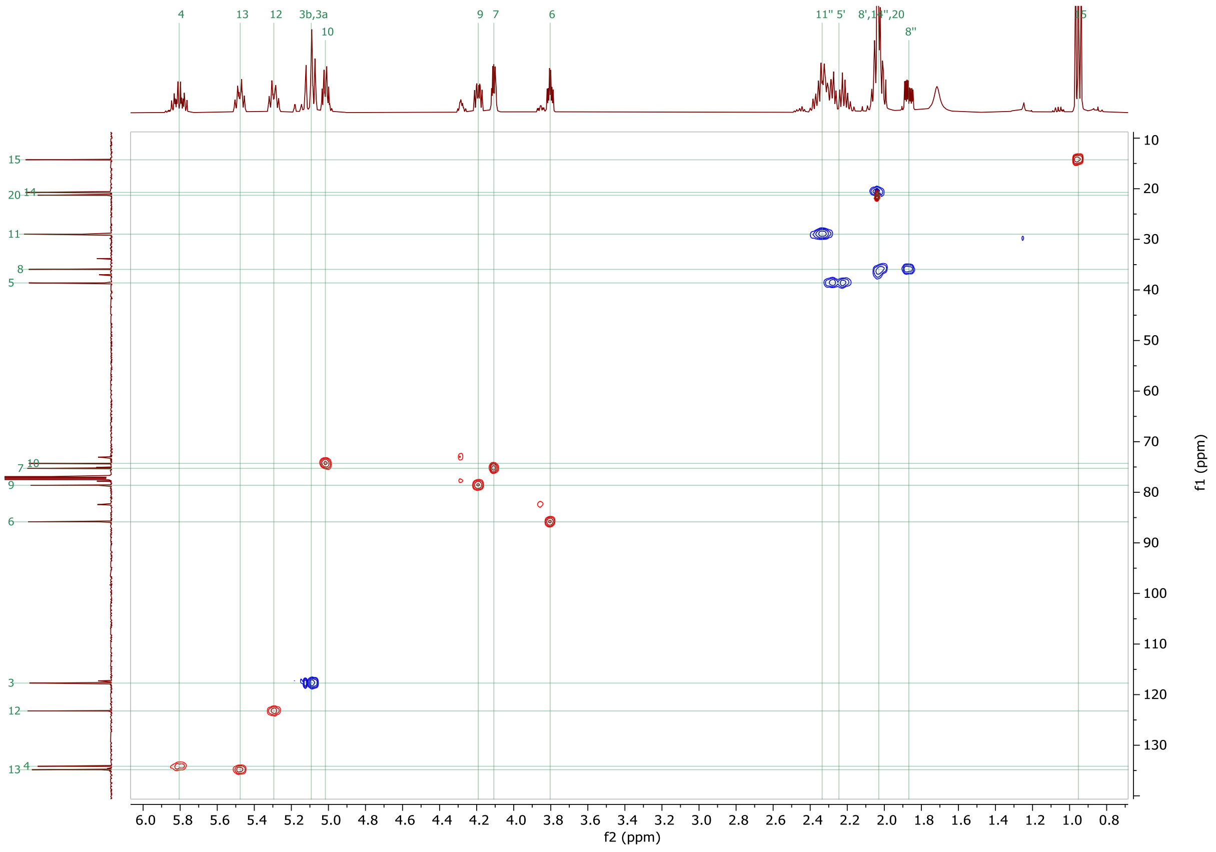

HMBC of 12

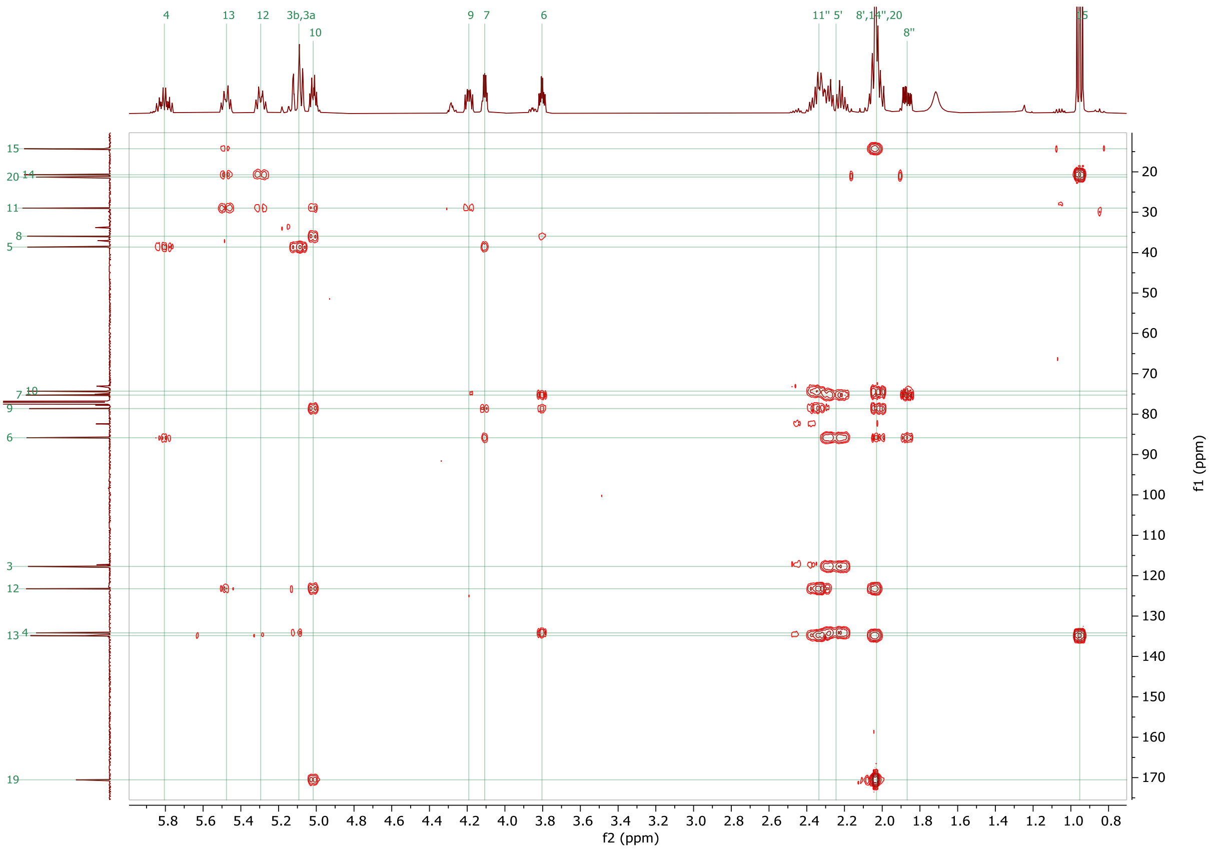
4
13
12
3b,3a
10
9
7
6
11''
5'
8',14'',20
8''
15
15
20
14
11
8
5
7
10
9
6
3
12
13
4
19
f2 (ppm)
f1 (ppm)

(1R,Z)-1-((2S,3aR,6aR)-5-((Trimethylsilyl)methyl)hexahydrofuro[3,2-b]furan-2-yl)hex-3-en-1-yl acetate (13)

^{1}H NMR (500 MHz, $CDCl_3$)

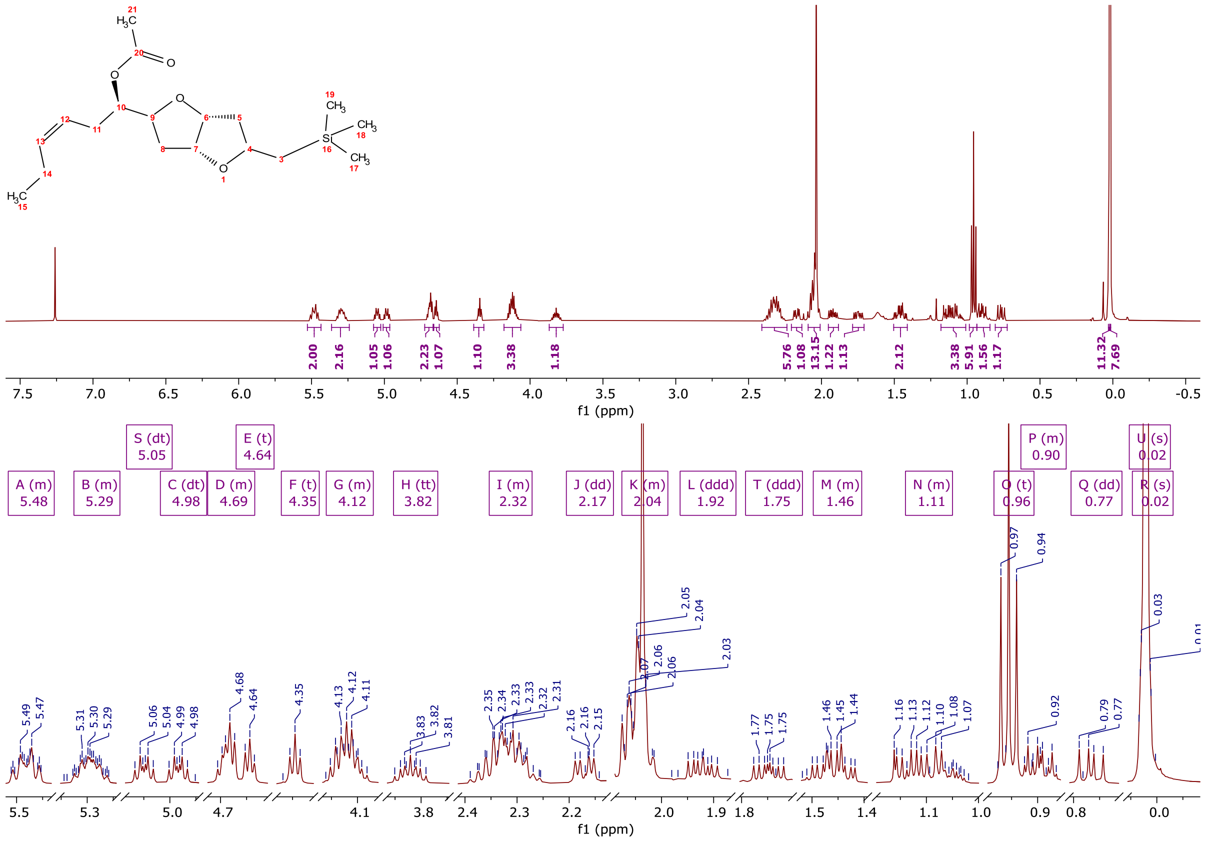

^{13}C NMR (126 MHz, $CDCl_3$)

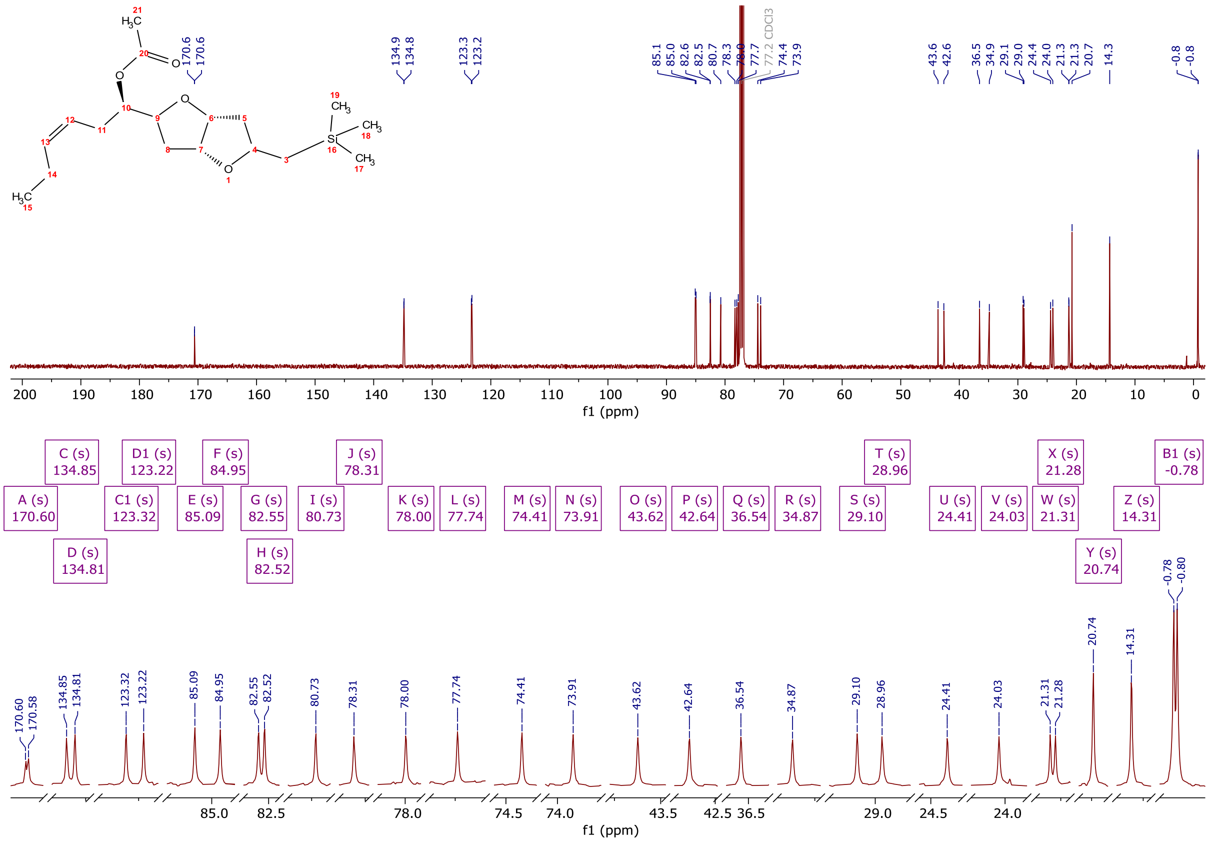

^1H-^1H COSY 13

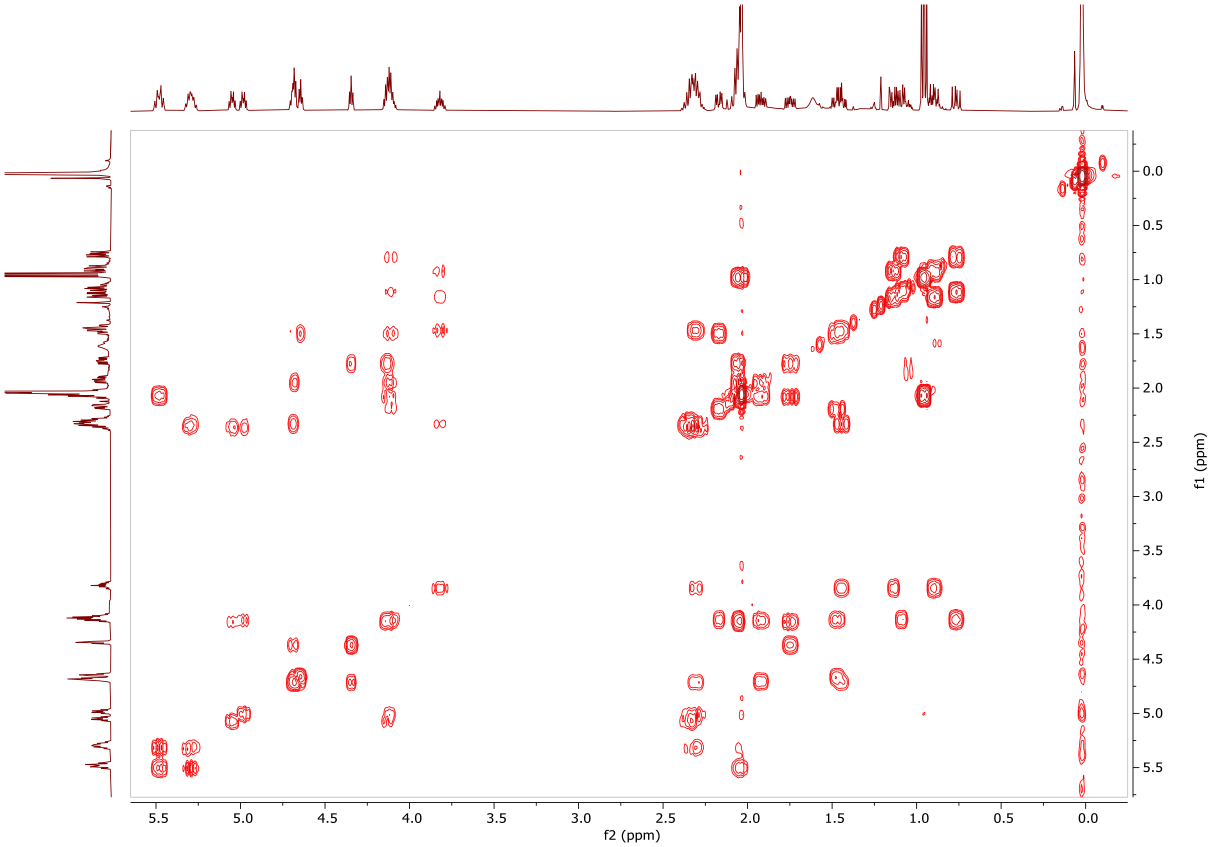

HSQC of 13

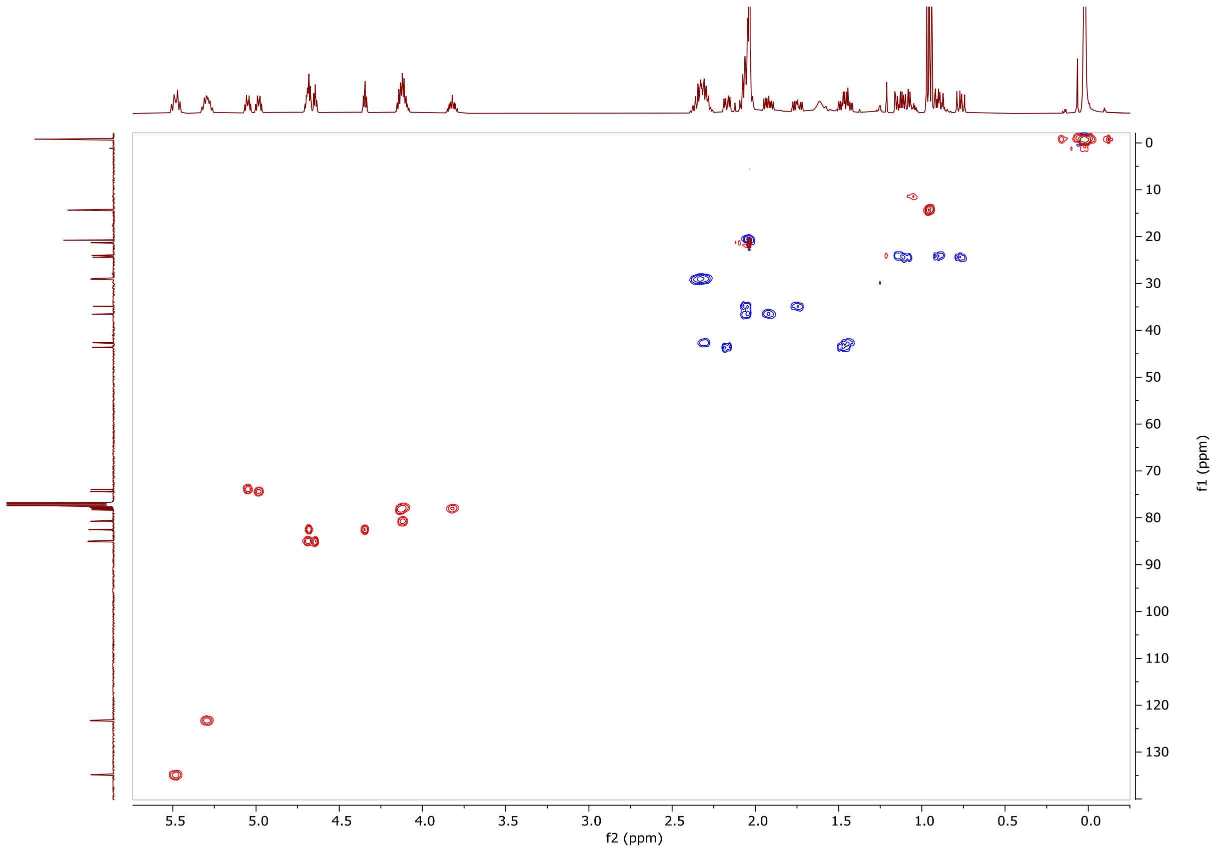

HMBC of 13

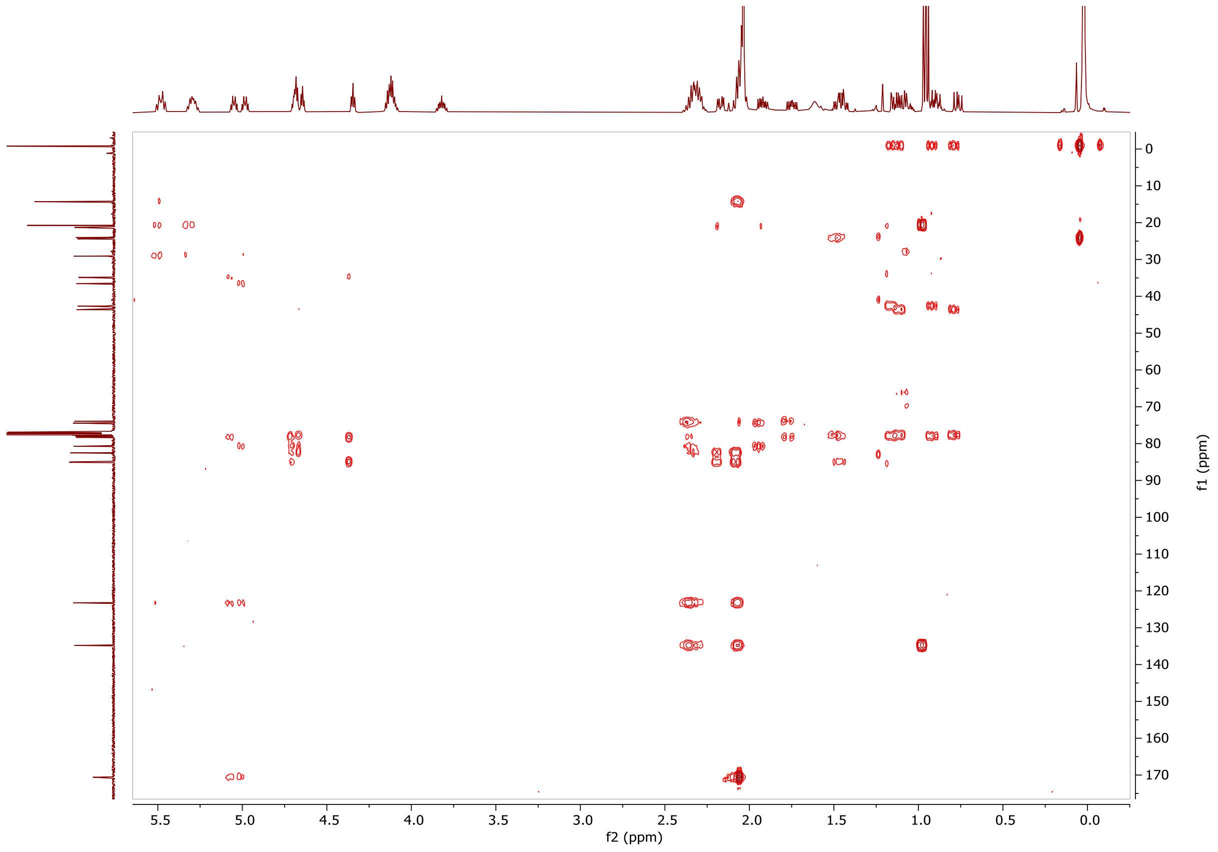

(*R*,*Z*)-1-((2*S*,4*R*,5*R*)-5-Allyl-4-hydroxytetrahydrofuran-2-yl)hex-3-en-1-yl acetate (S2)

^{1}H NMR (500 MHz, $CDCl_3$)

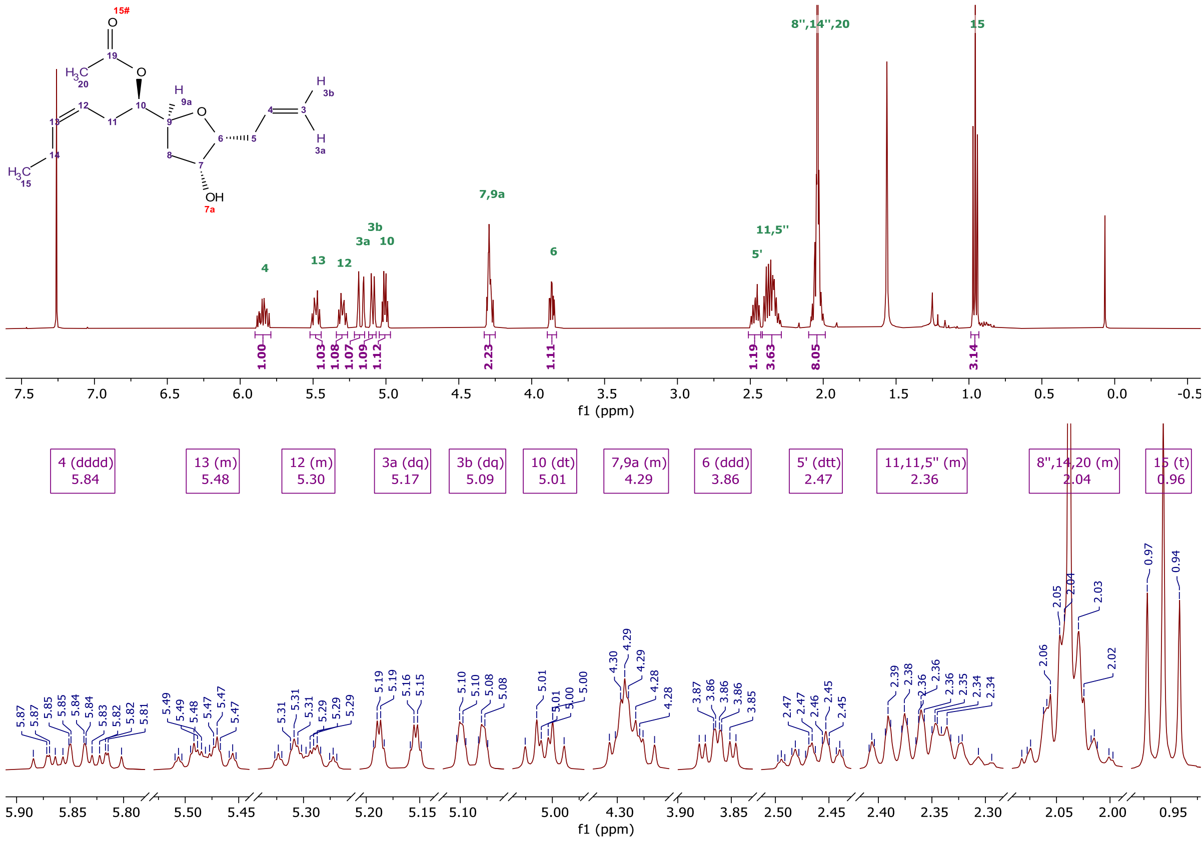

^{13}C NMR (126 MHz, $CDCl_3$)

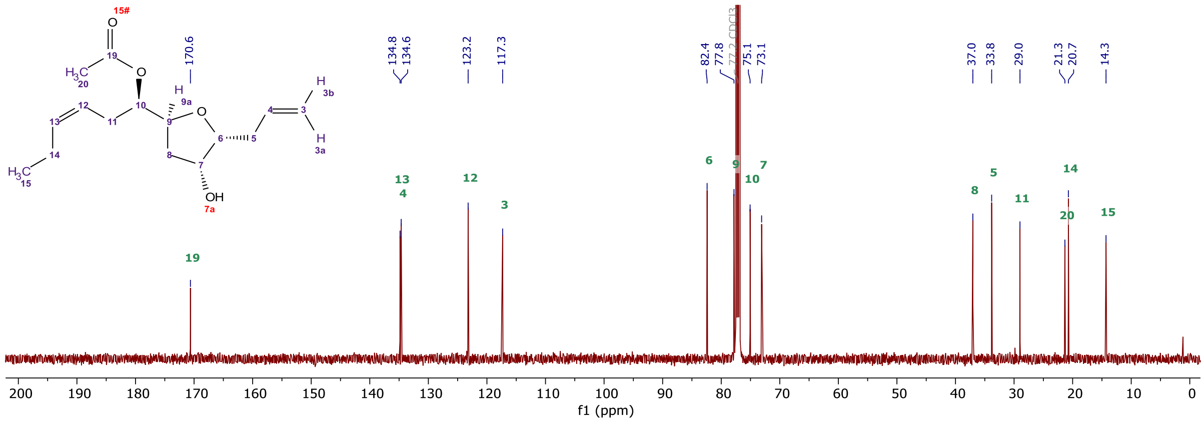

^{1}H-^{1}H COSY of S2

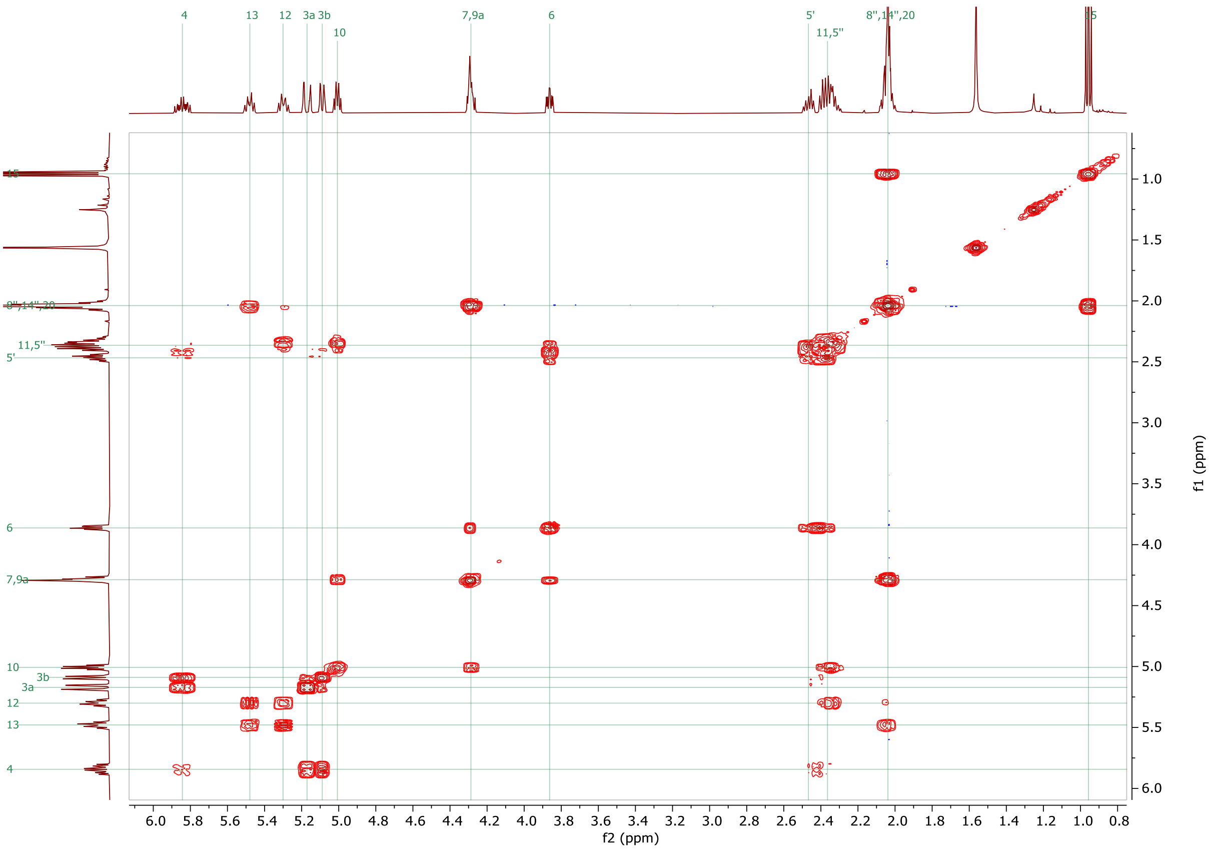

HSQC of S2

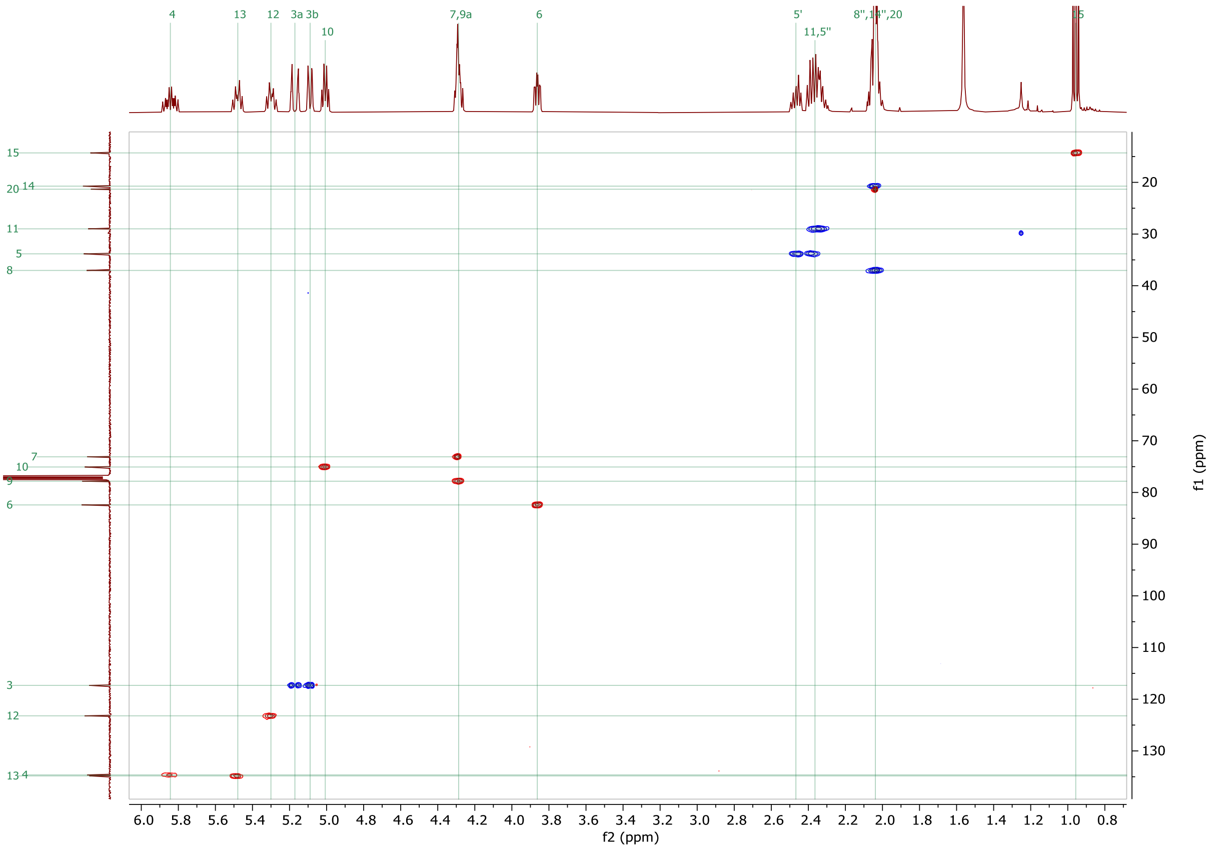

HMBC of S2

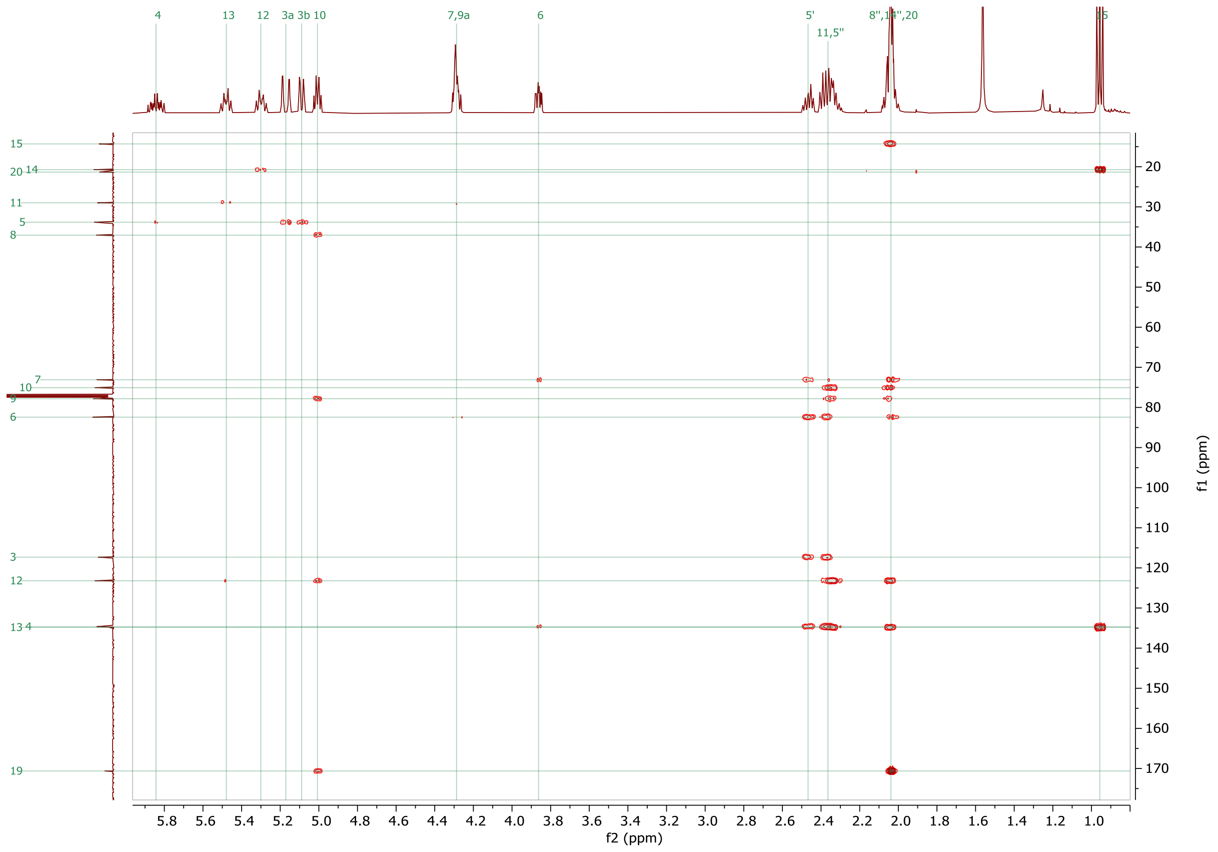

4
13
12
3a
3b
10
7,9a
6
5'
11,5''
8'',14'',20
15
20
14
11
5
8
7
10
9
6
3
12
13
4
19
f2 (ppm)
f1 (ppm)

(2*S*,3*R*,5*S*)-2-Allyl-5-((*R*,*Z*)-1-hydroxyhex-3-en-1-yl)tetrahydrofuran-3-yl methanesulfonate (14)

^{1}H NMR (500 MHz, $CDCl_3$)

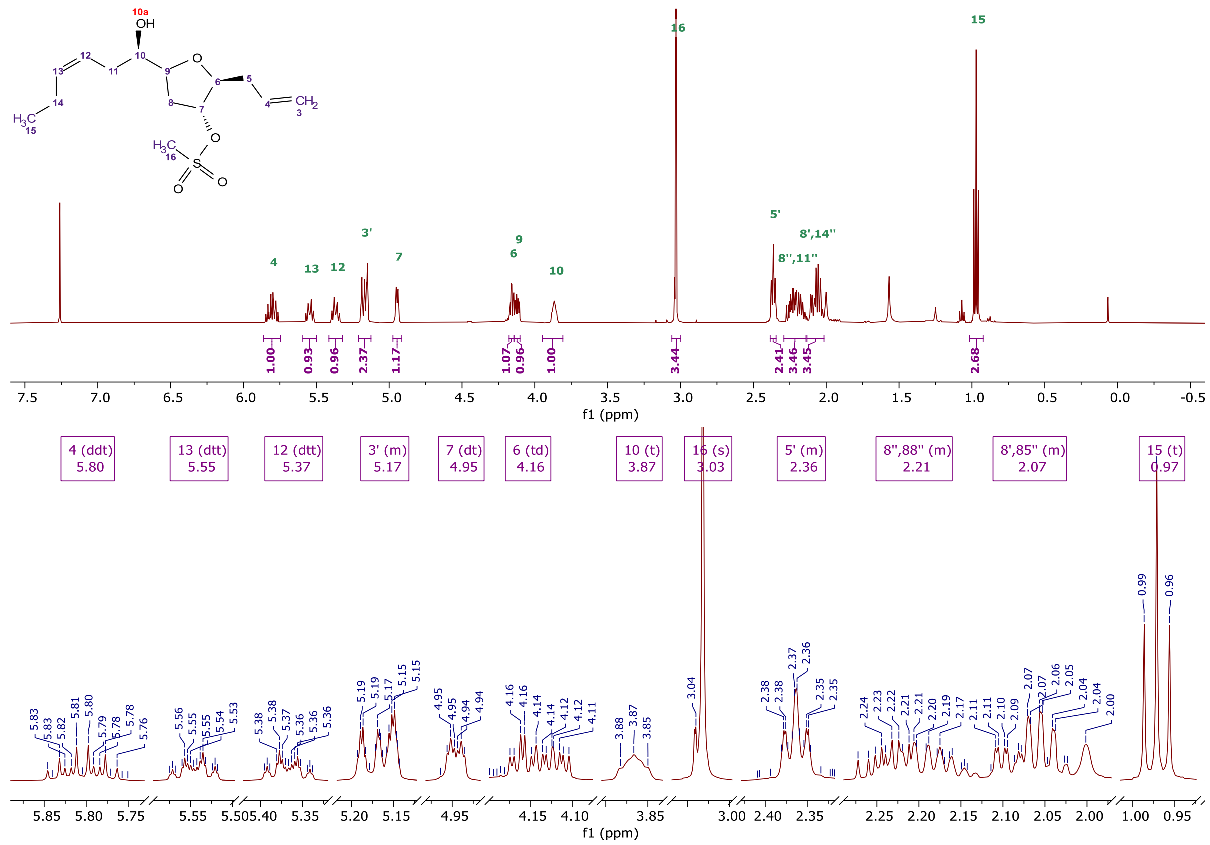

^{13}C NMR (126 MHz, $CDCl_3$)

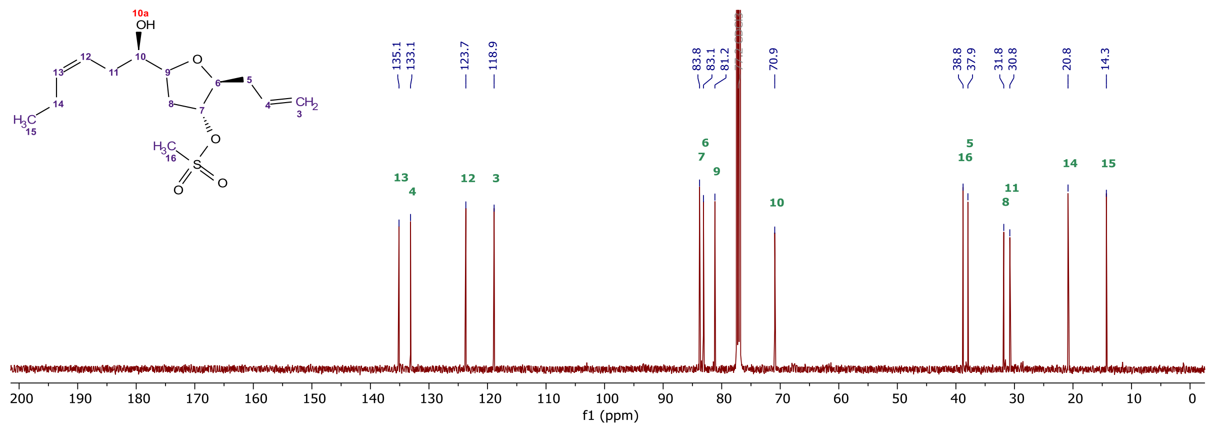

^1H-^1H COSY of 14

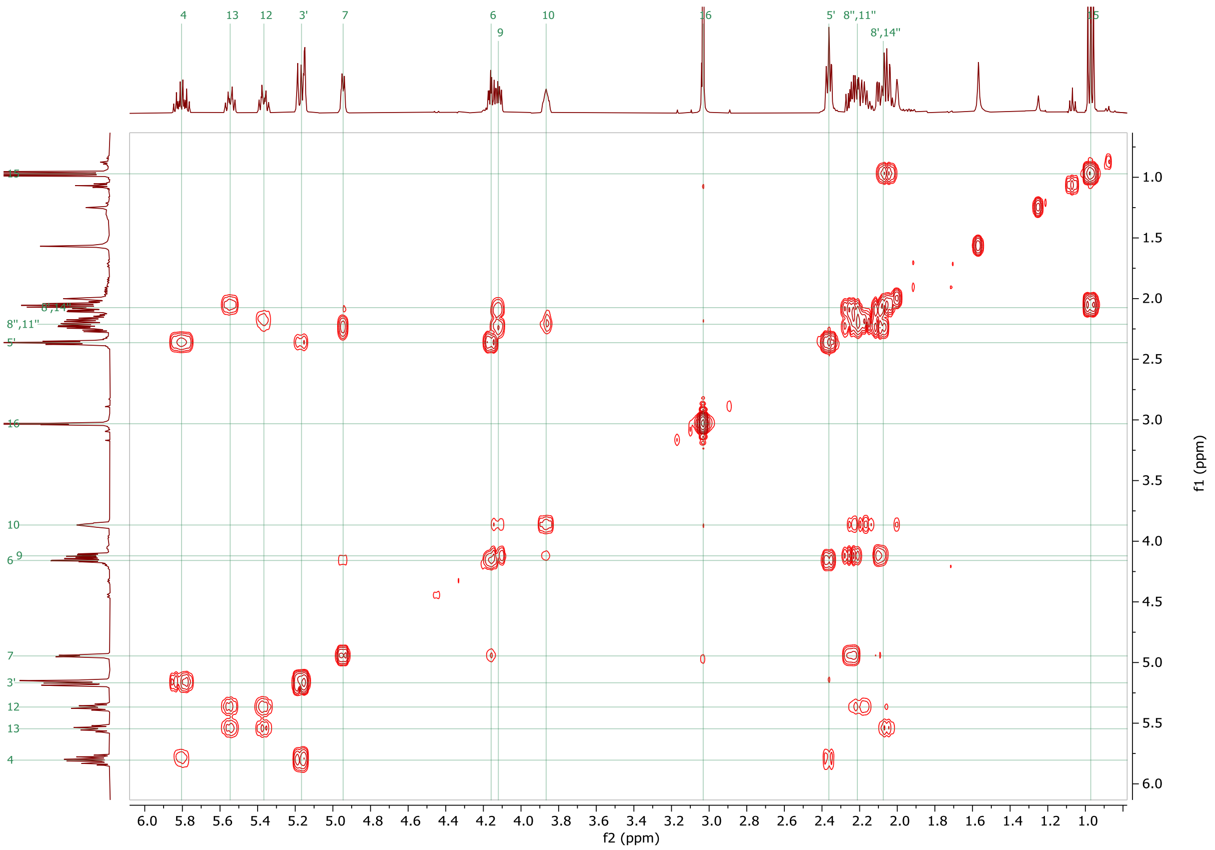

HSQC of 14

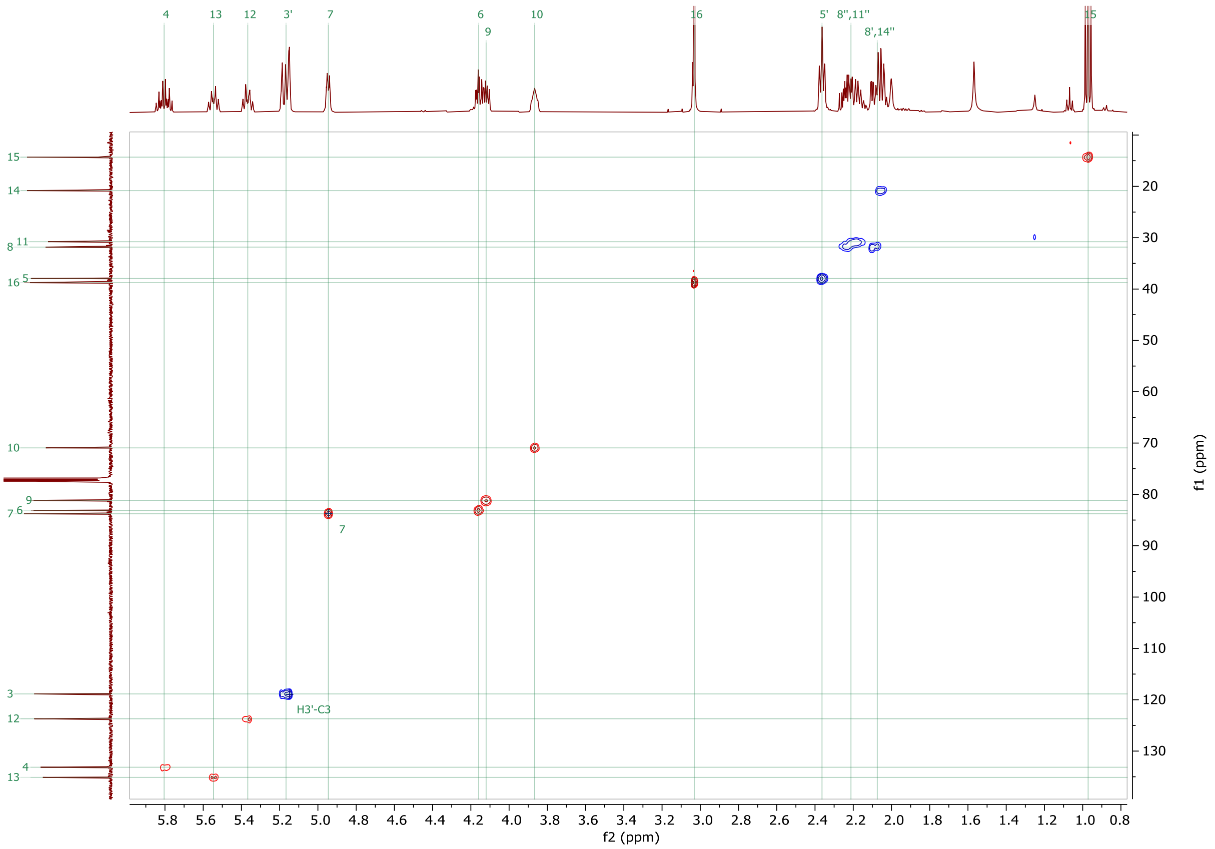

HMBC of 14

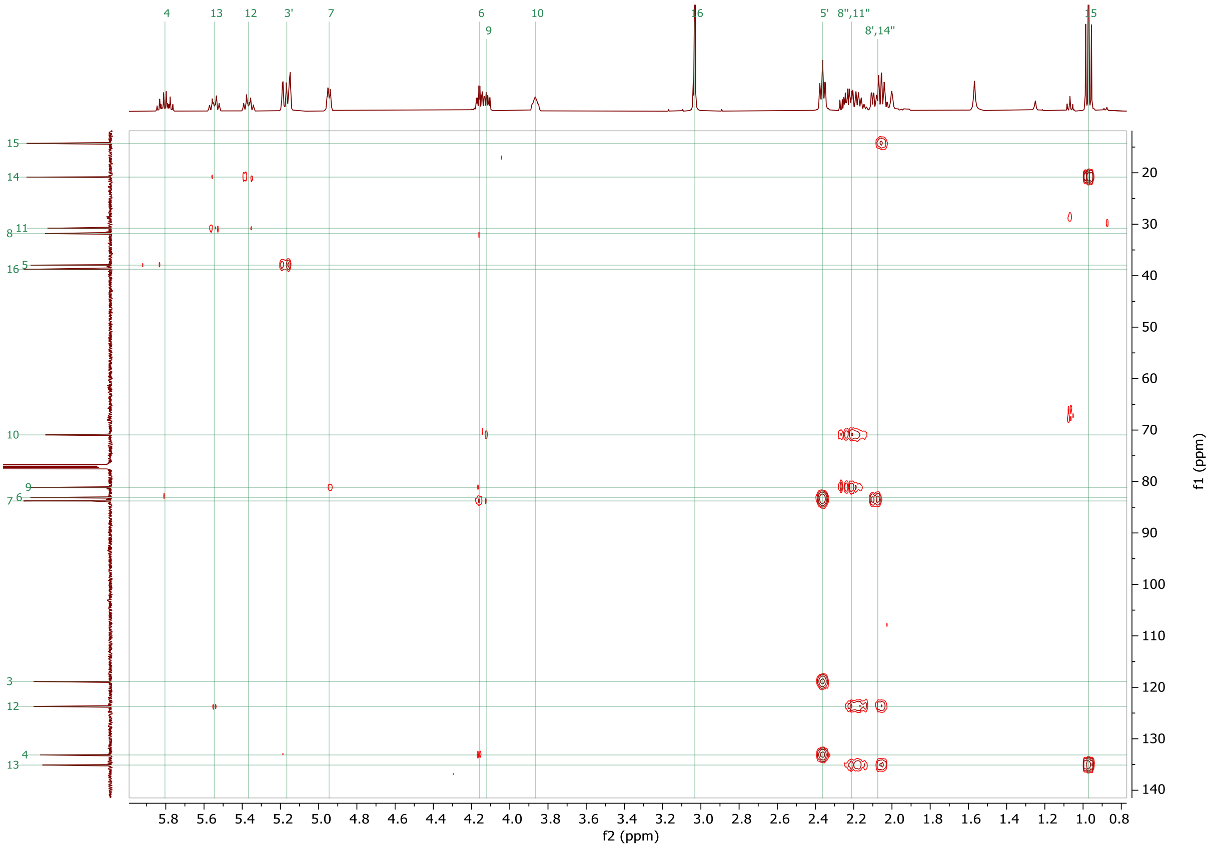

4
13
12
3'
7
6
9
10
16
5'
8'',11''
8',14''
15
14
11
8
5
16
10
9
6
7
3
12
4
13
f1 (ppm)
f2 (ppm)

(1*S*,3*S*,4*S*,6*R*)-3-Allyl-6-((*Z*)-pent-2-en-1-yl)-2,5-dioxabicyclo[2.2.1]heptane (15)

^{1}H NMR (500 MHz, $CDCl_3$)

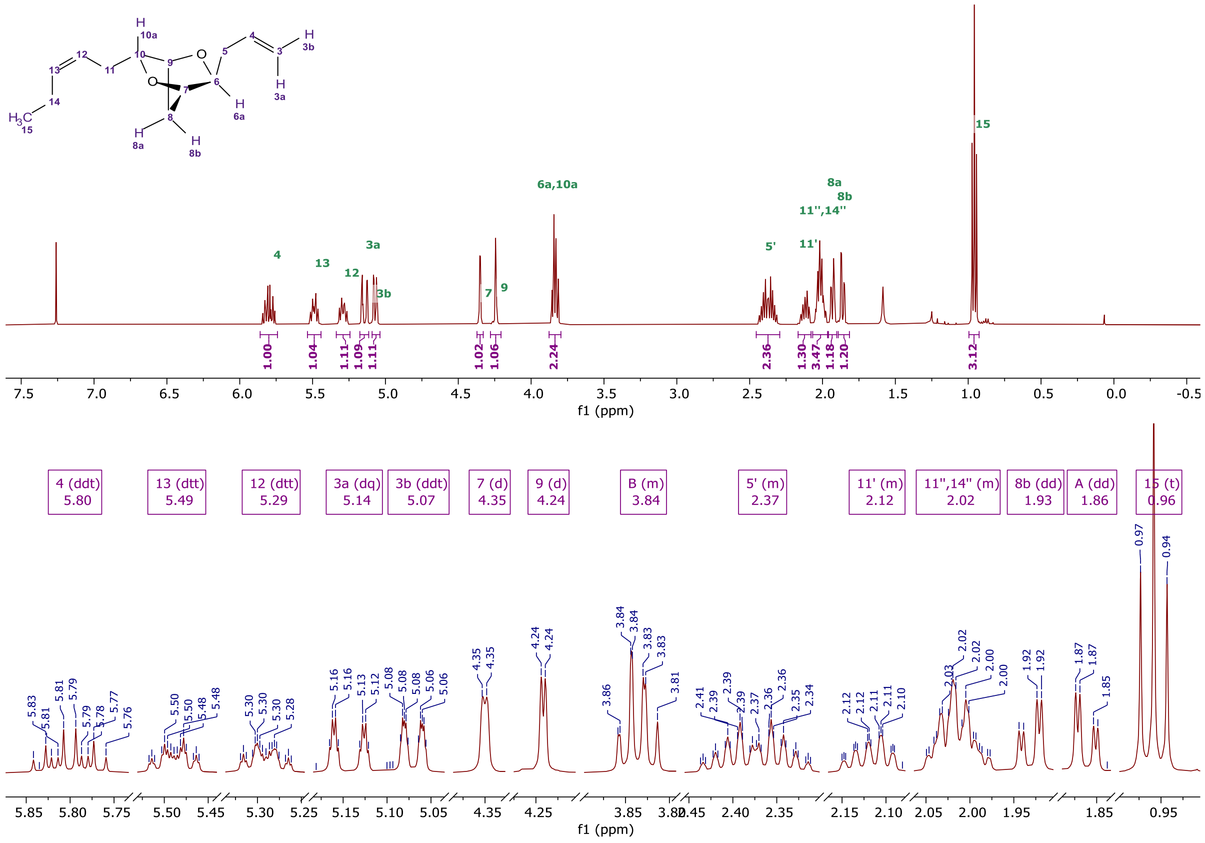

^{13}C NMR (126 MHz, $CDCl_3$)

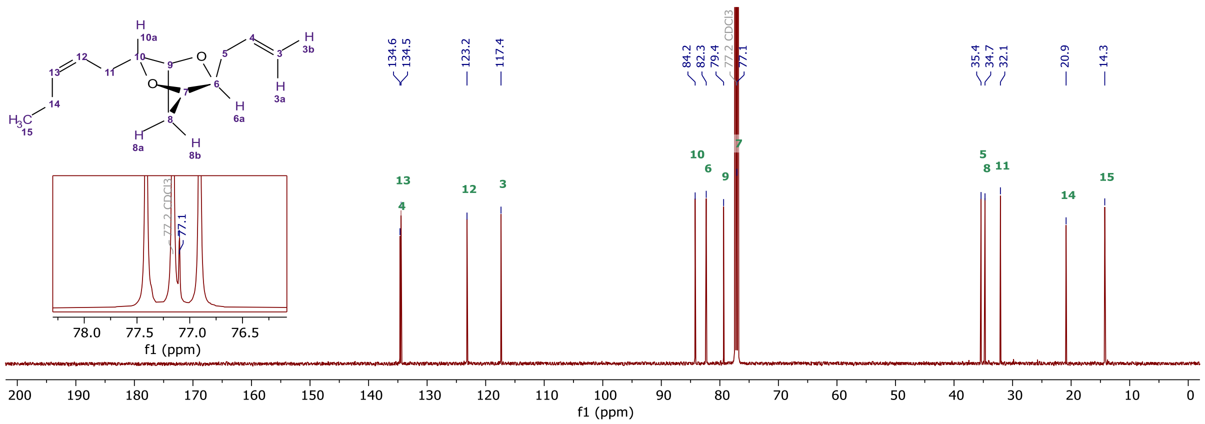

^{1}H-^{1}H COSY 15

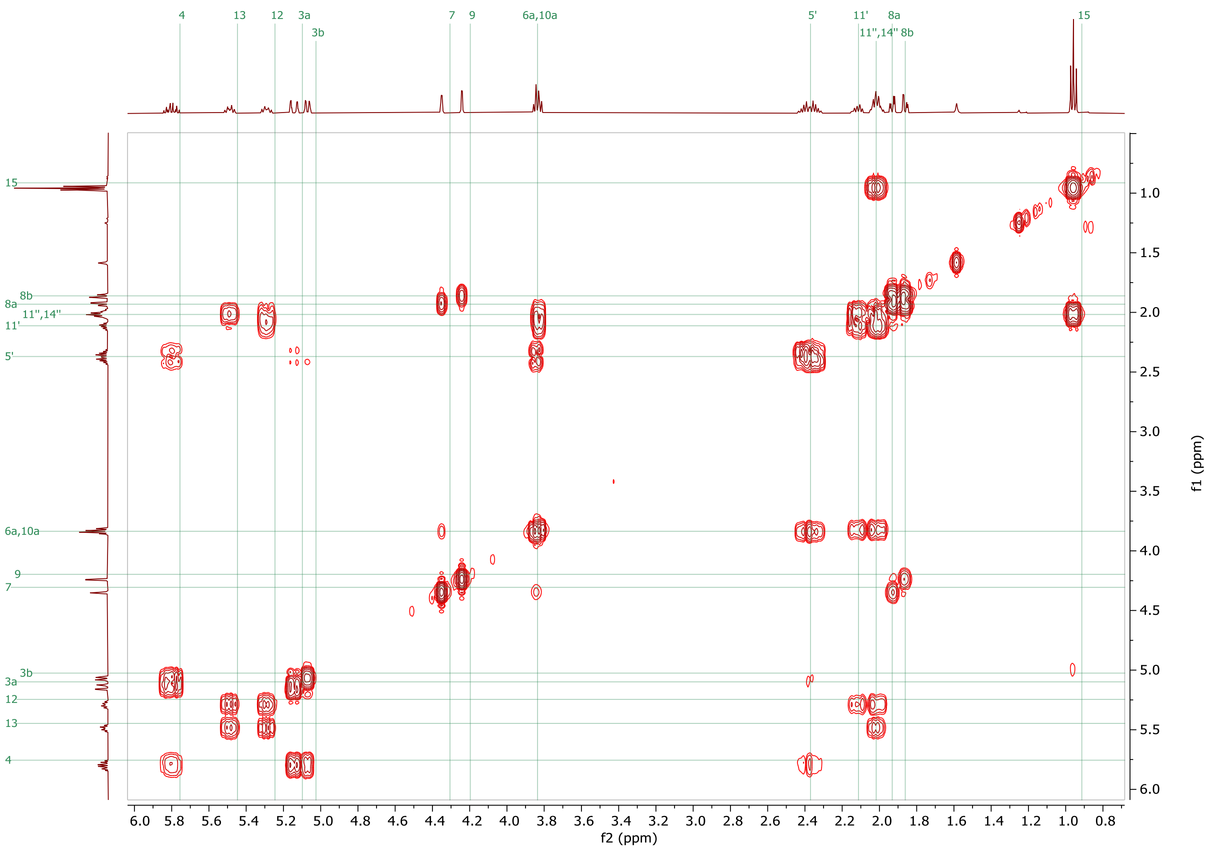

HSQC of 15

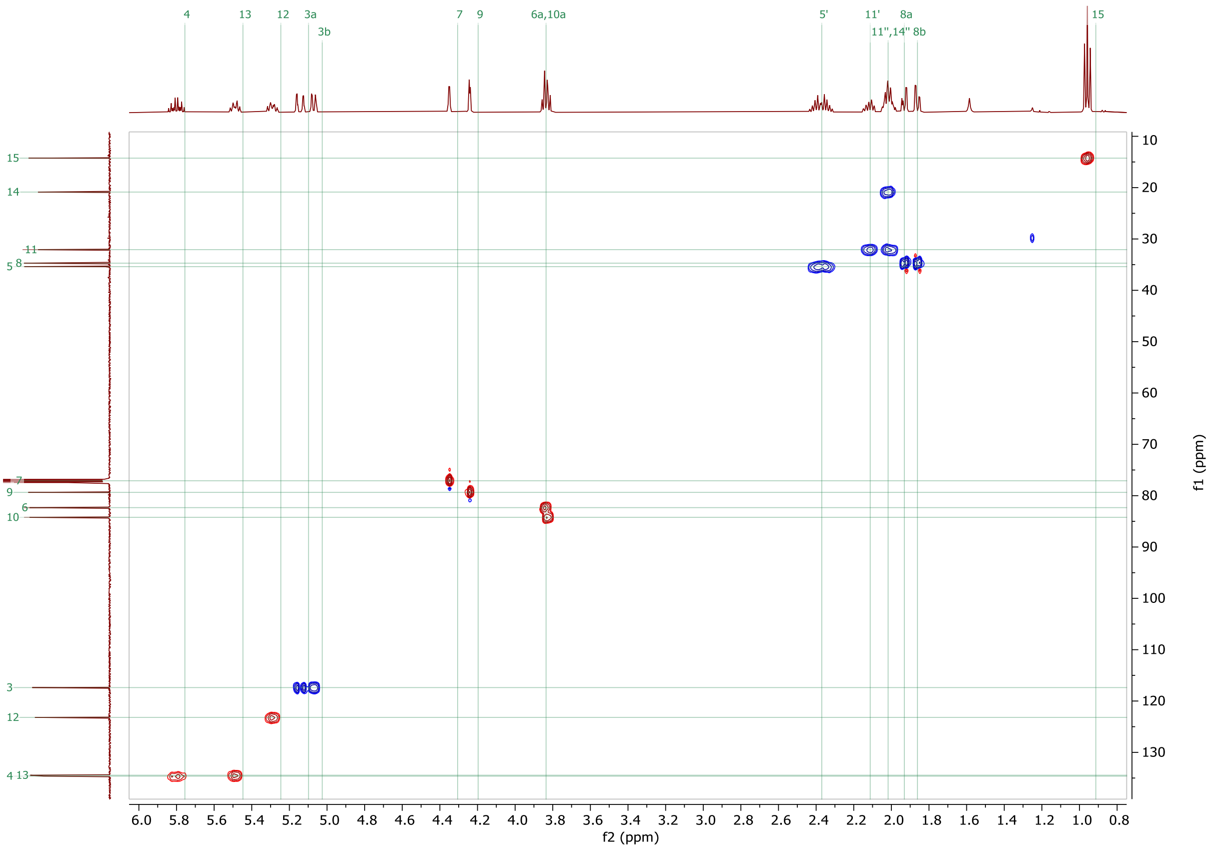

HMBC of 15

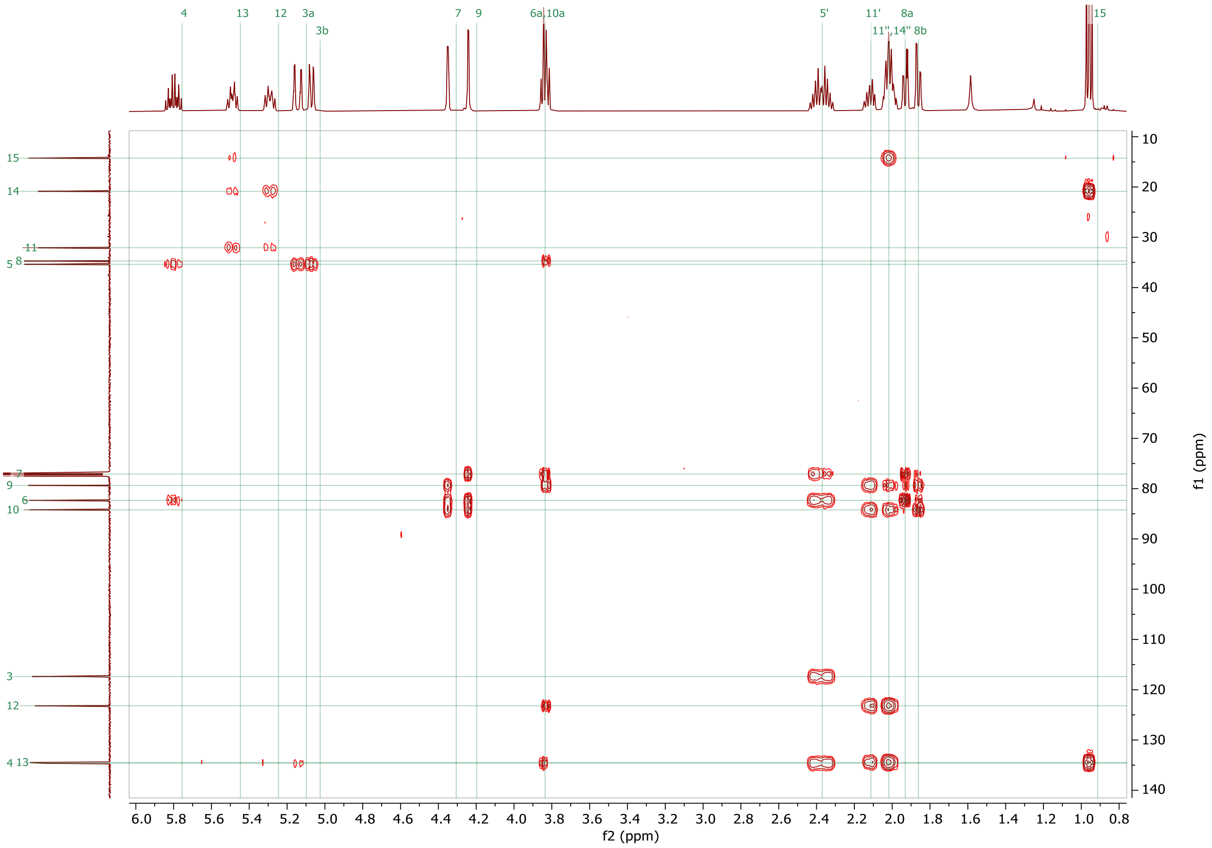
4
13
12
3a
3b
7
9
6a
10a
5'
11'
11''
14''
8a
8b
15
14
11
5
8
9
6
10
3
12
4
13
f1 (ppm)
f2 (ppm)

(1S,3S,4S,6R)-3-Allyl-6-((2R,3R)-2,3-dibromopentyl)-2,5-dioxabicyclo[2.2.1]heptane (16)

^{1}H NMR (600 MHz, $CDCl_3$)

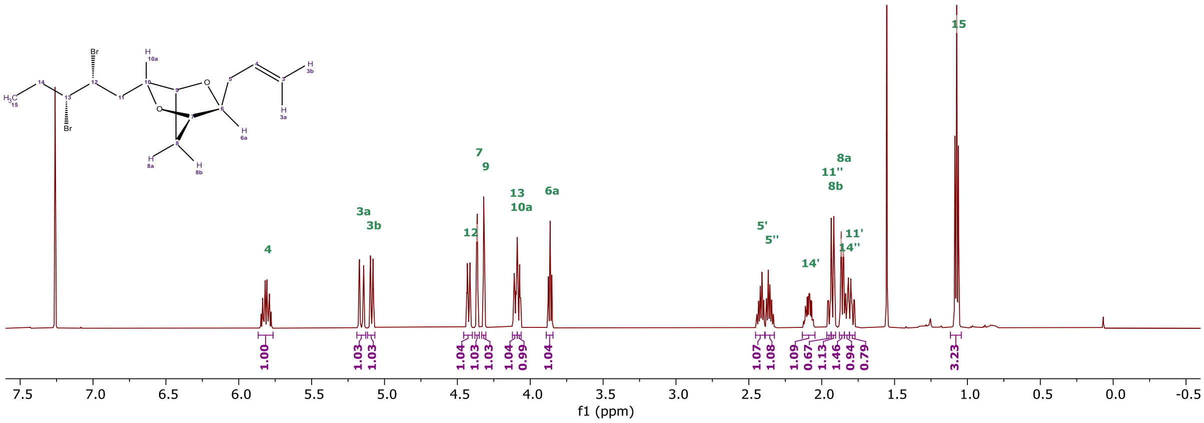

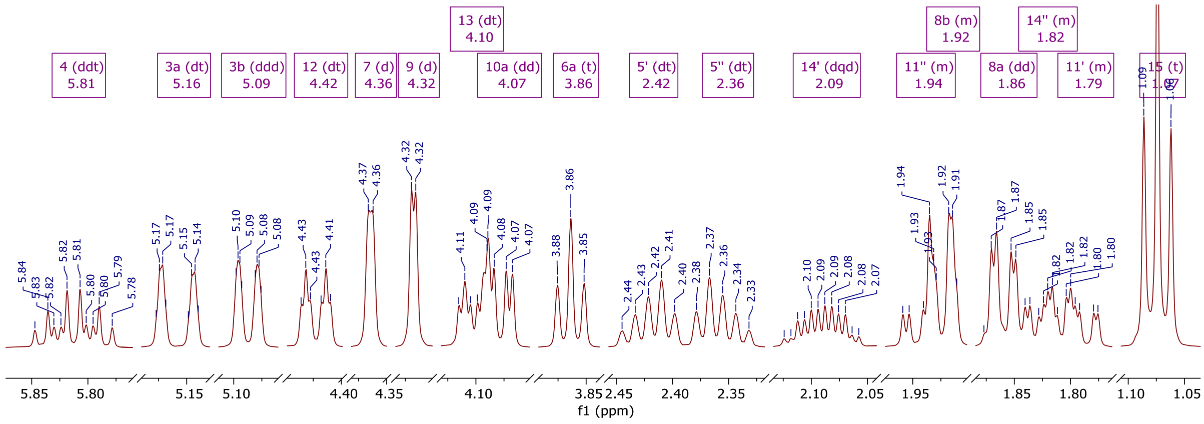

^{13}C NMR (151 MHz, $CDCl_3$)

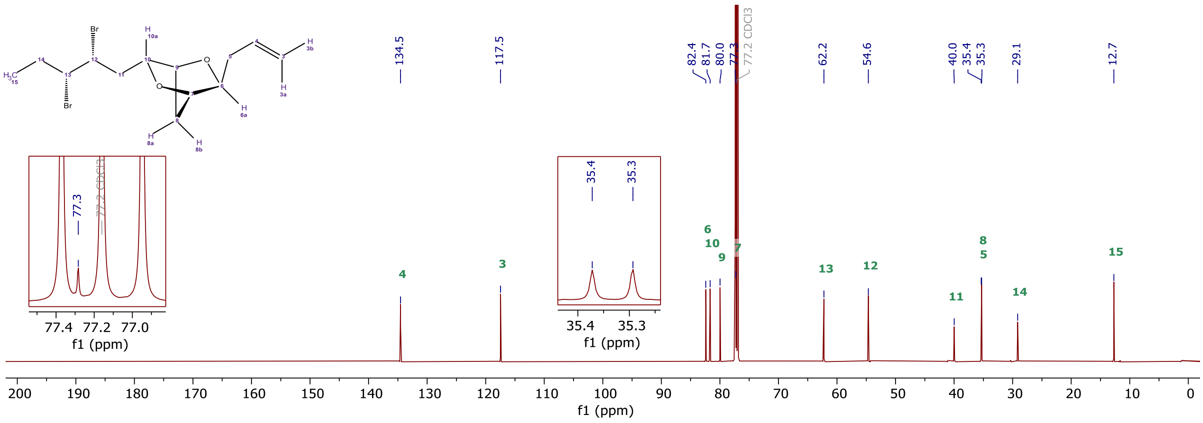

^{1}H-1H COSY 16

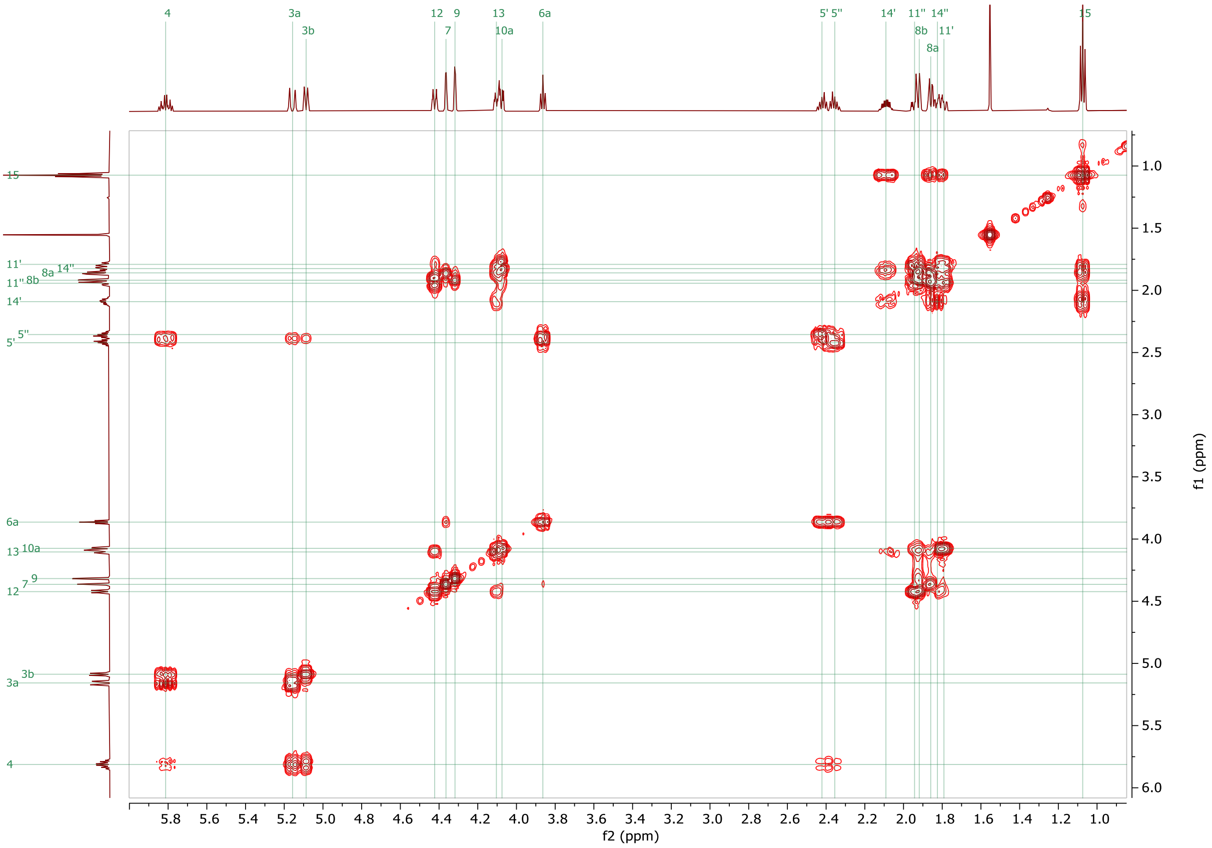

HSQC of 16

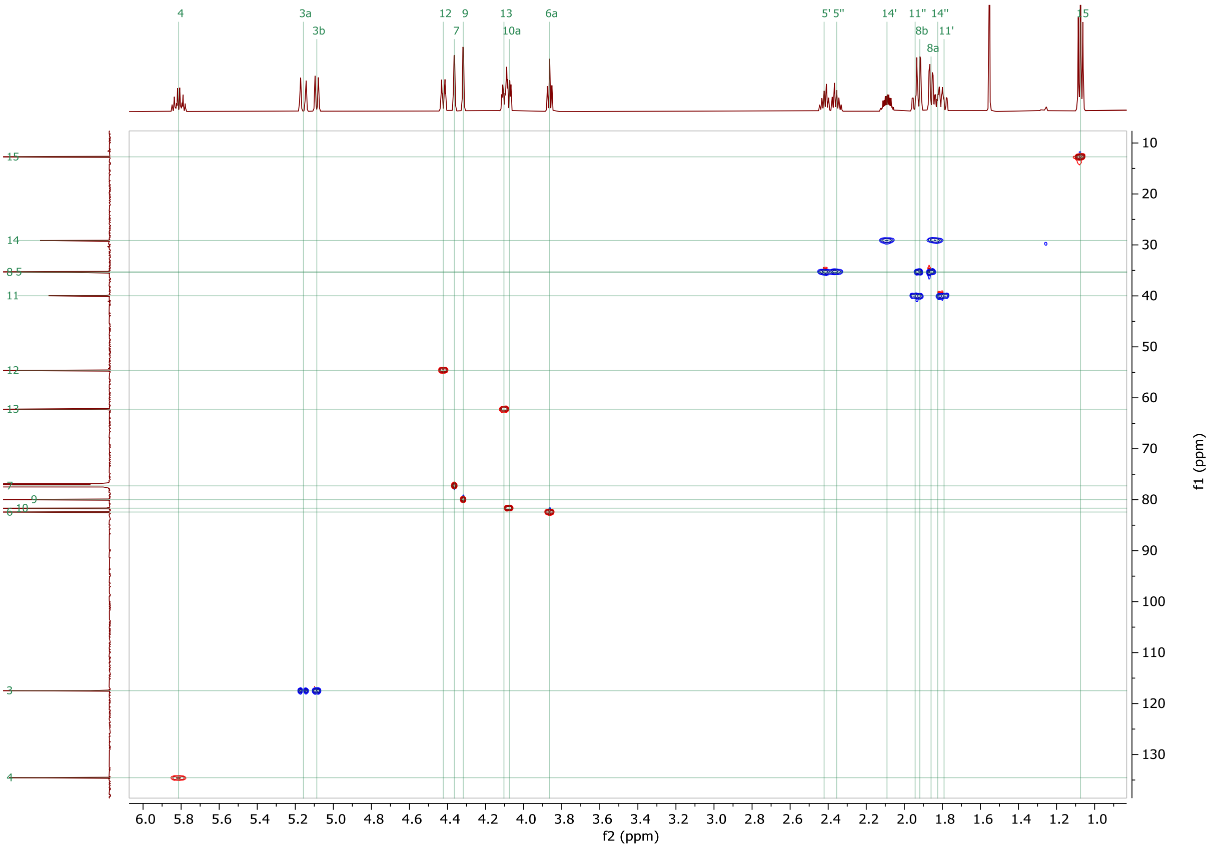

HMBC of 16

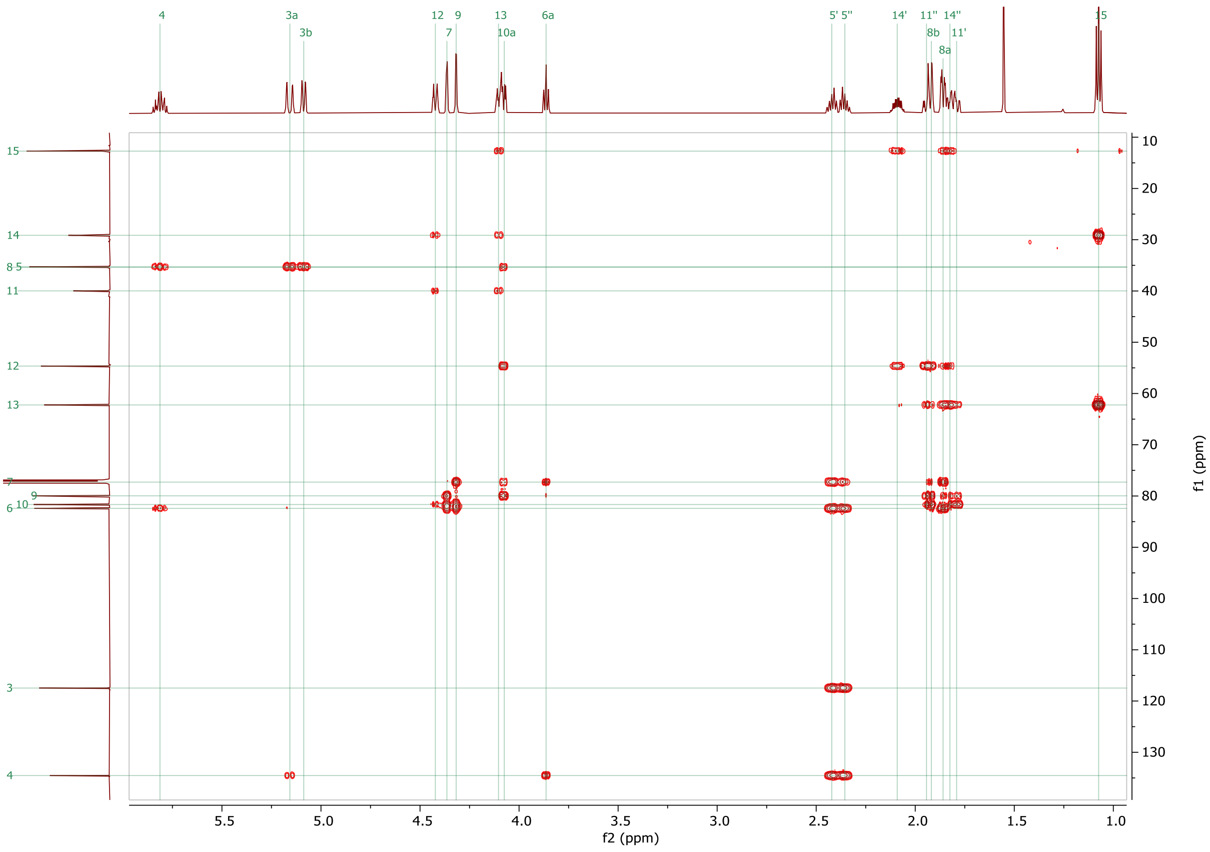

^{1}H-^{1}H NOESY of 16

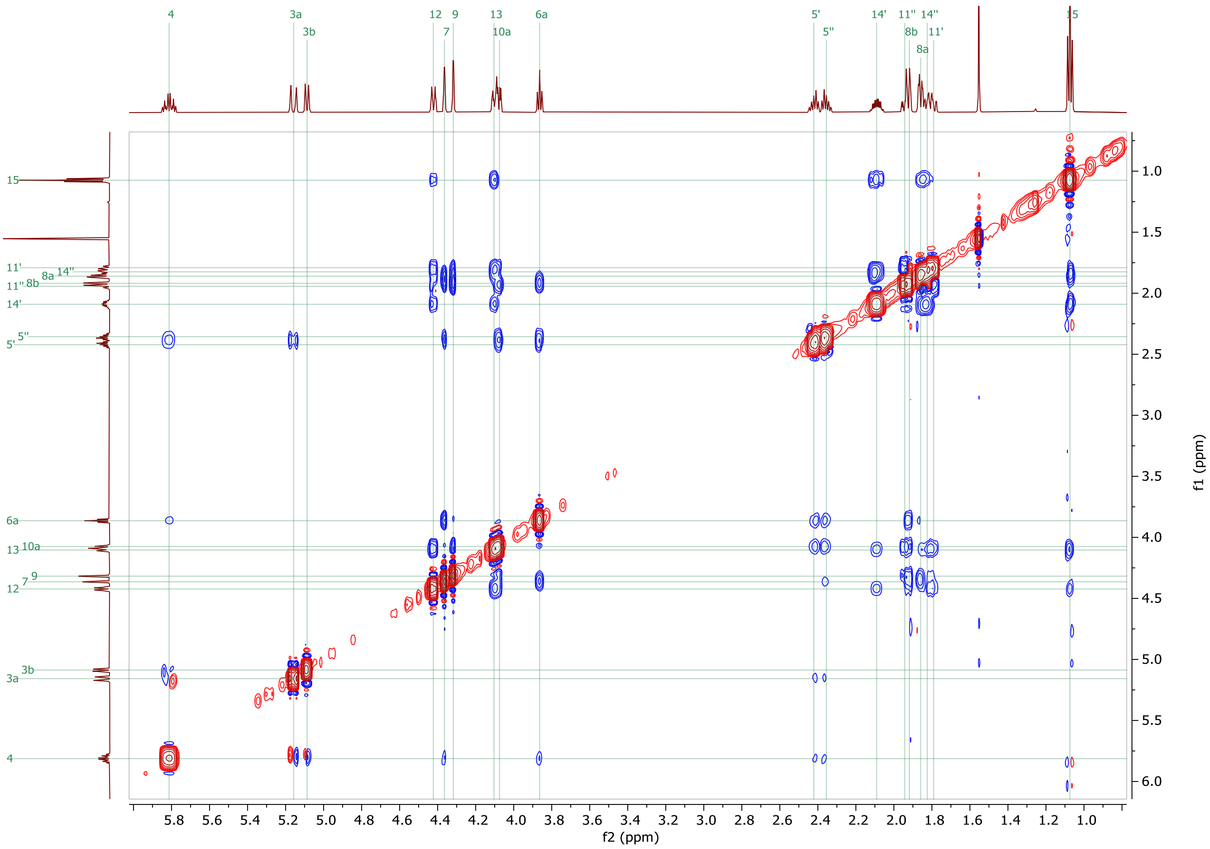

(1*S*,3*S*,4*S*,6*R*)-3-Allyl-6-((2*S*,3*S*)-2,3-dibromopentyl)-2,5-dioxabicyclo[2.2.1]heptane (17)

^{1}H NMR (600 MHz, $CDCl_3$)

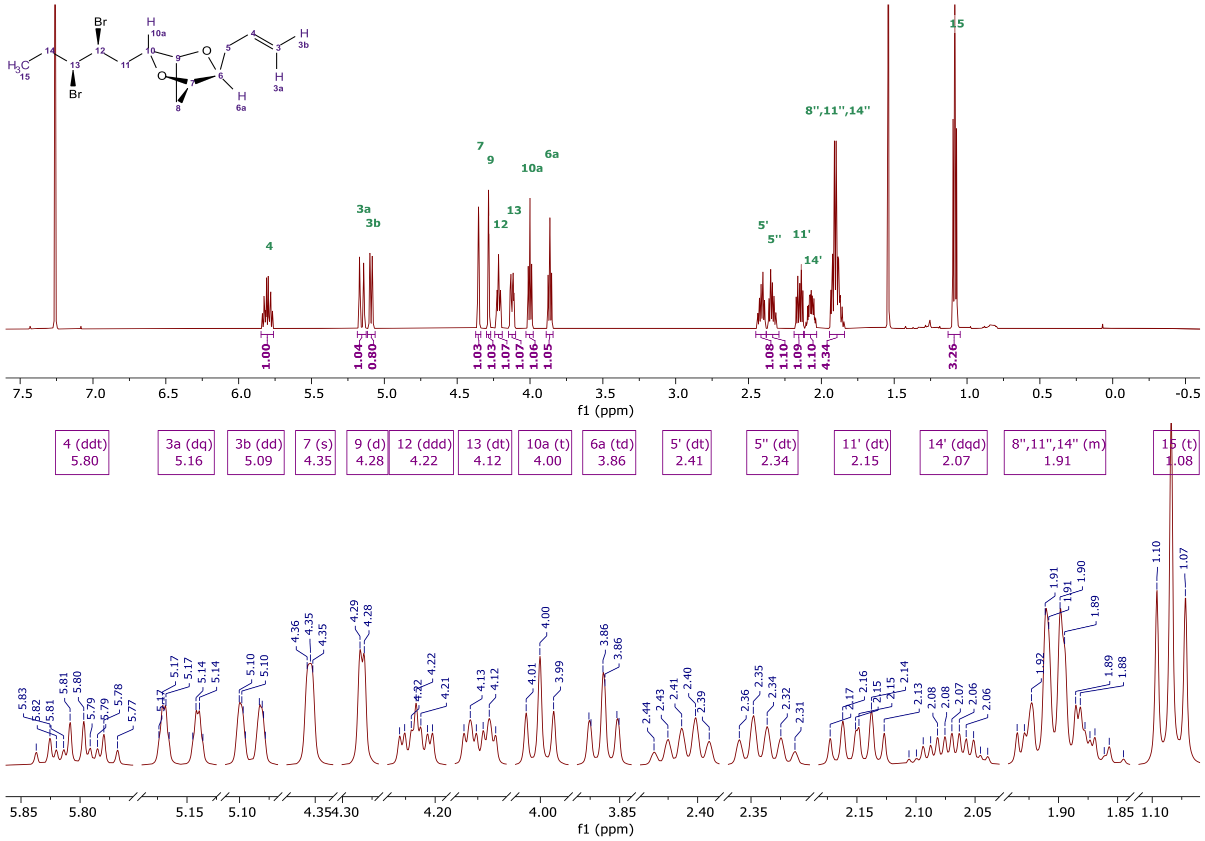

^{13}C NMR (151 MHz, $CDCl_3$)

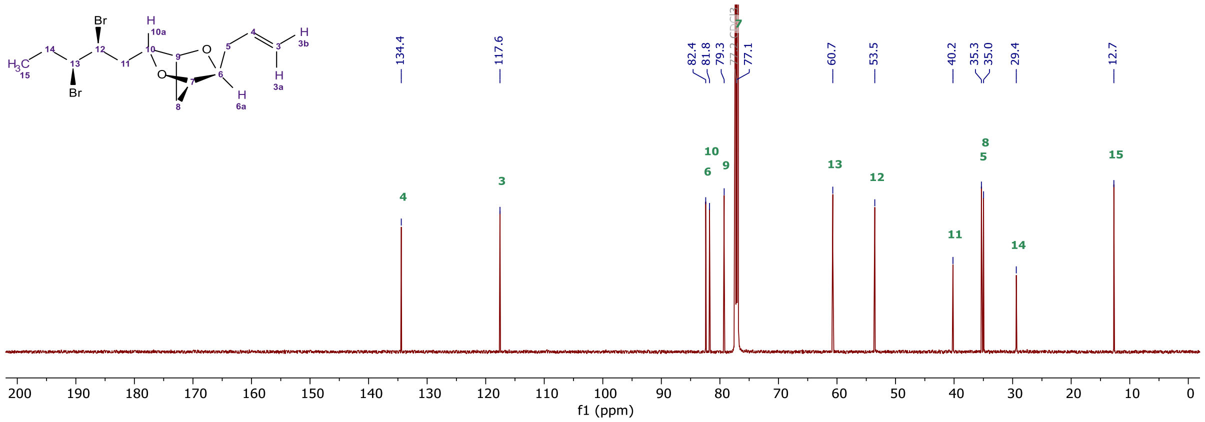

1H-1H COSY of 17

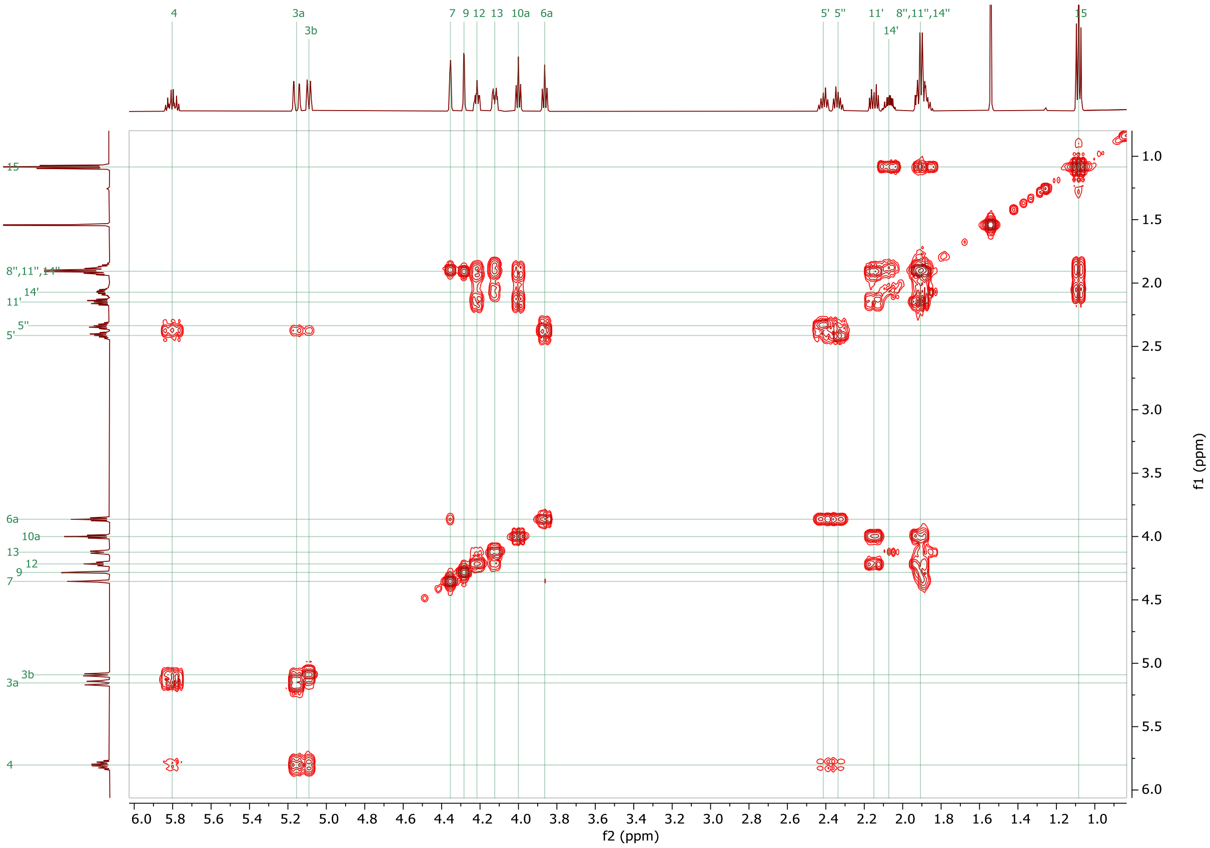

HSQC of 17

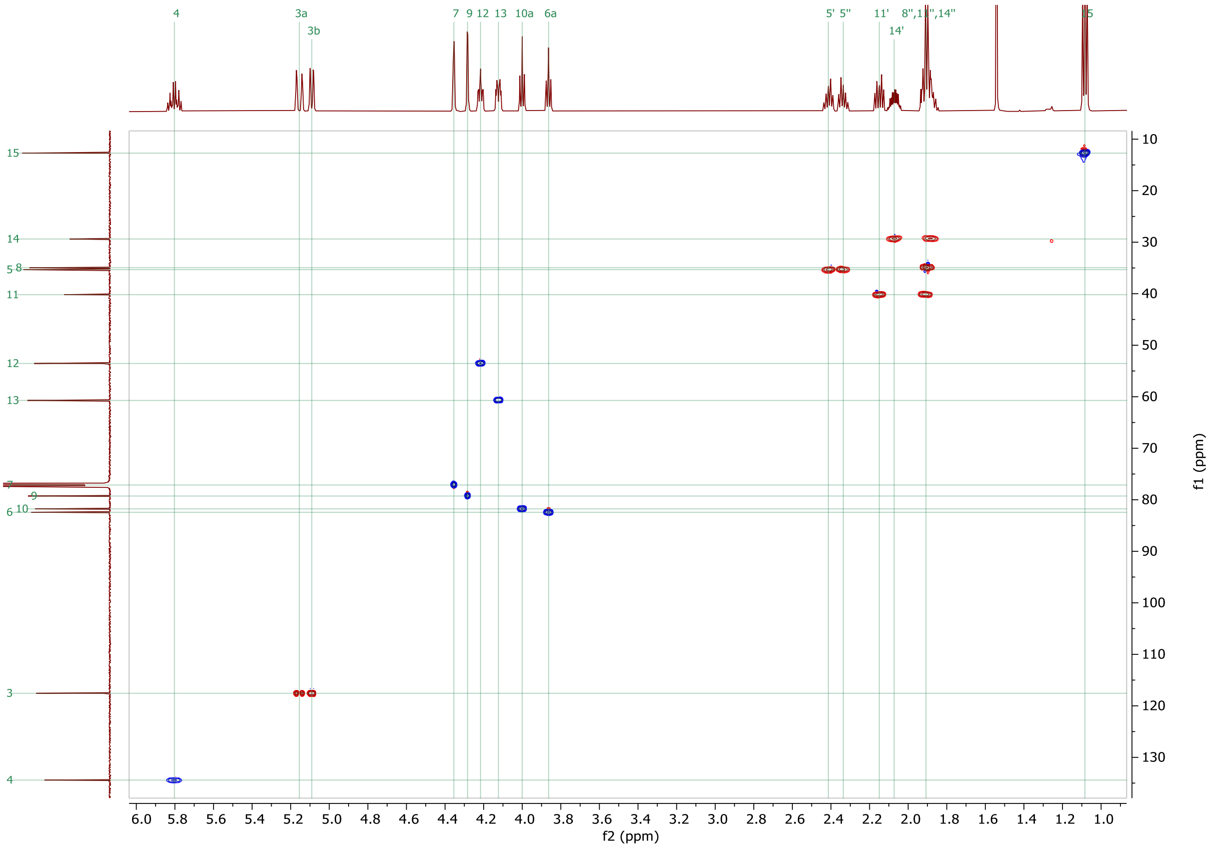

HMBC of 17

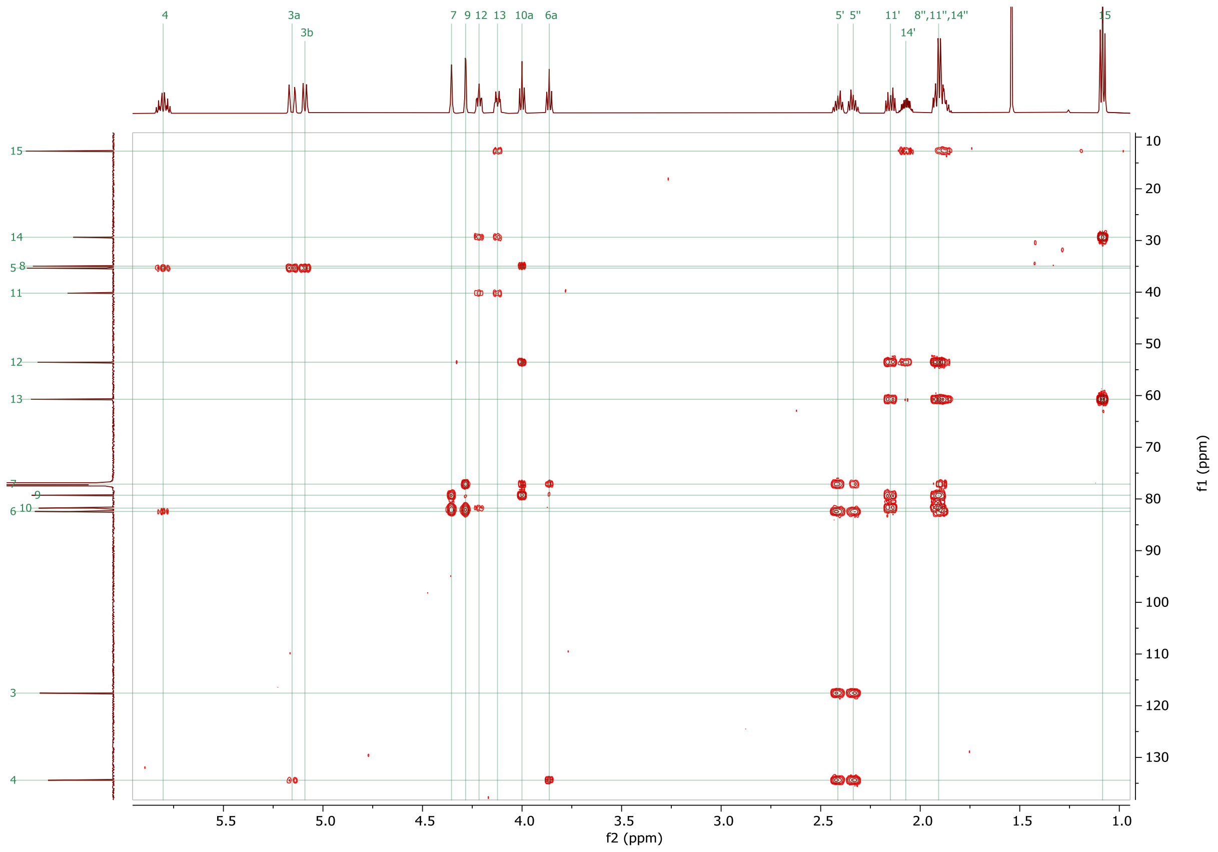

^{1}H-^{1}H NOESY of 17

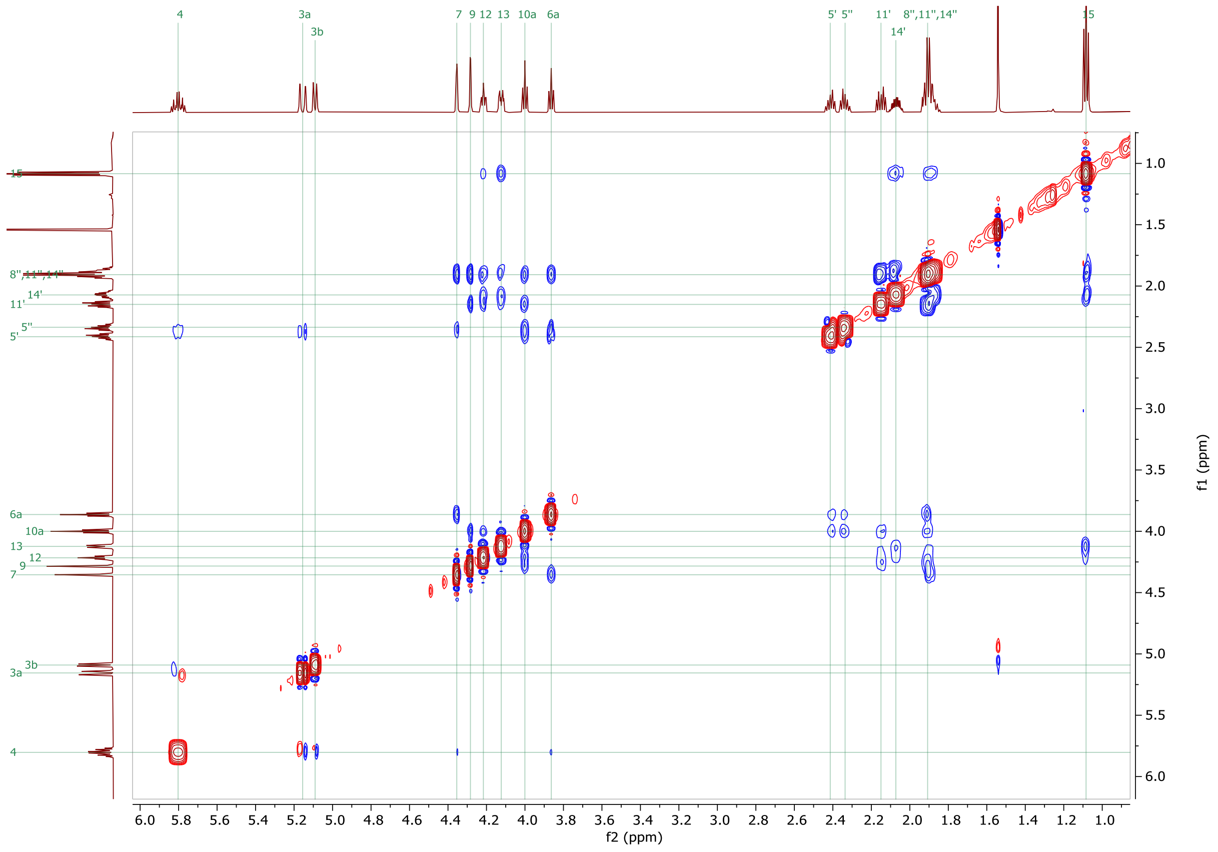

(*E*)-1-(2-((1*S*,3*S*,4*S*,6*R*)-6-((2*S*,3*S*)-2,3-Dibromopentyl)-2,5-dioxabicyclo[2.2.1]heptan-3-yl)ethylidene)-2-(2,4-dinitrophenyl)hydrazine (18)

^{1}H NMR (600 MHz, $CDCl_3$)

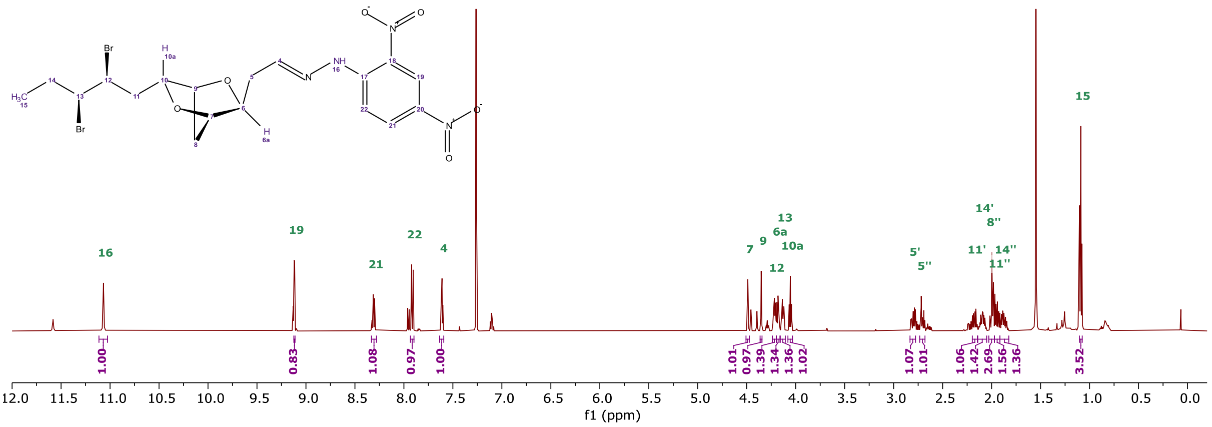

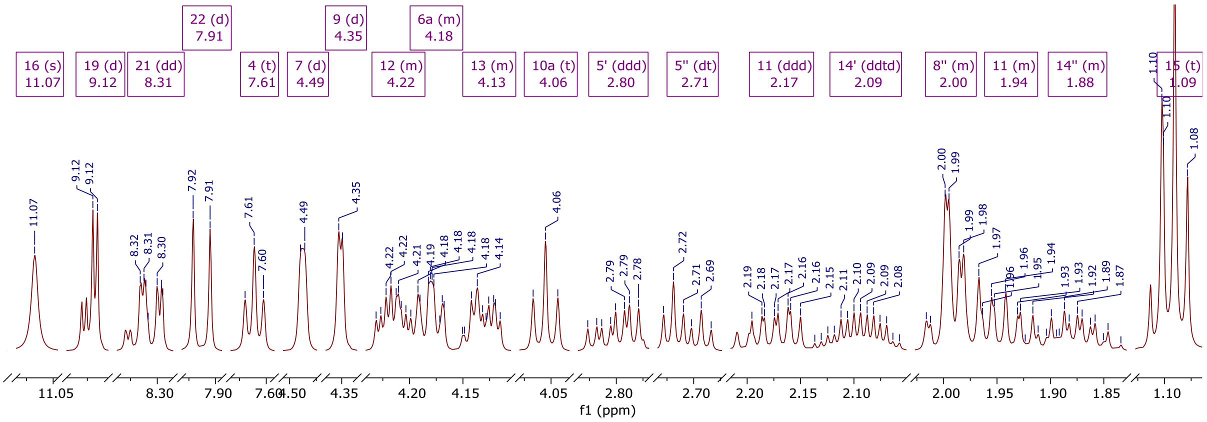

^{13}C NMR (151 MHz, $CDCl_3$)

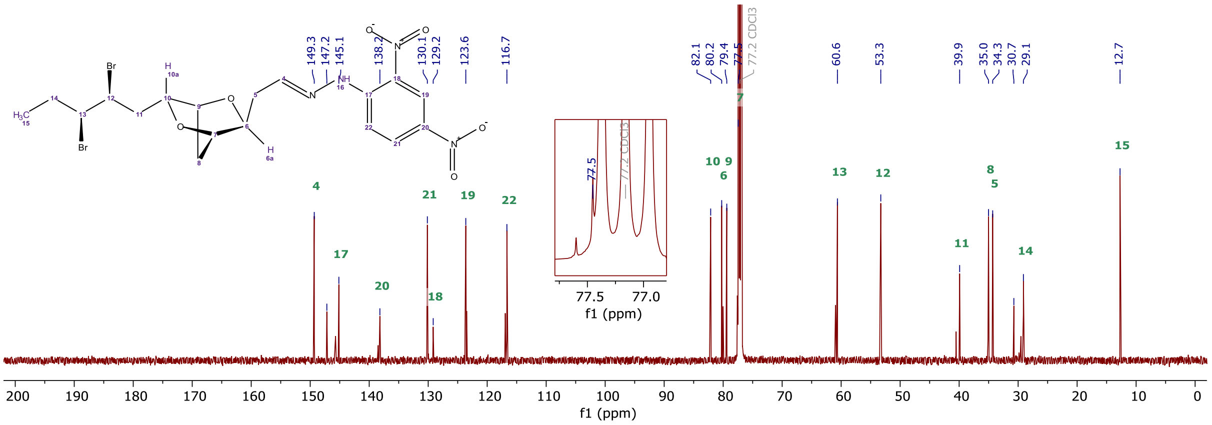

^{1}H-^{1}H COSY of 18

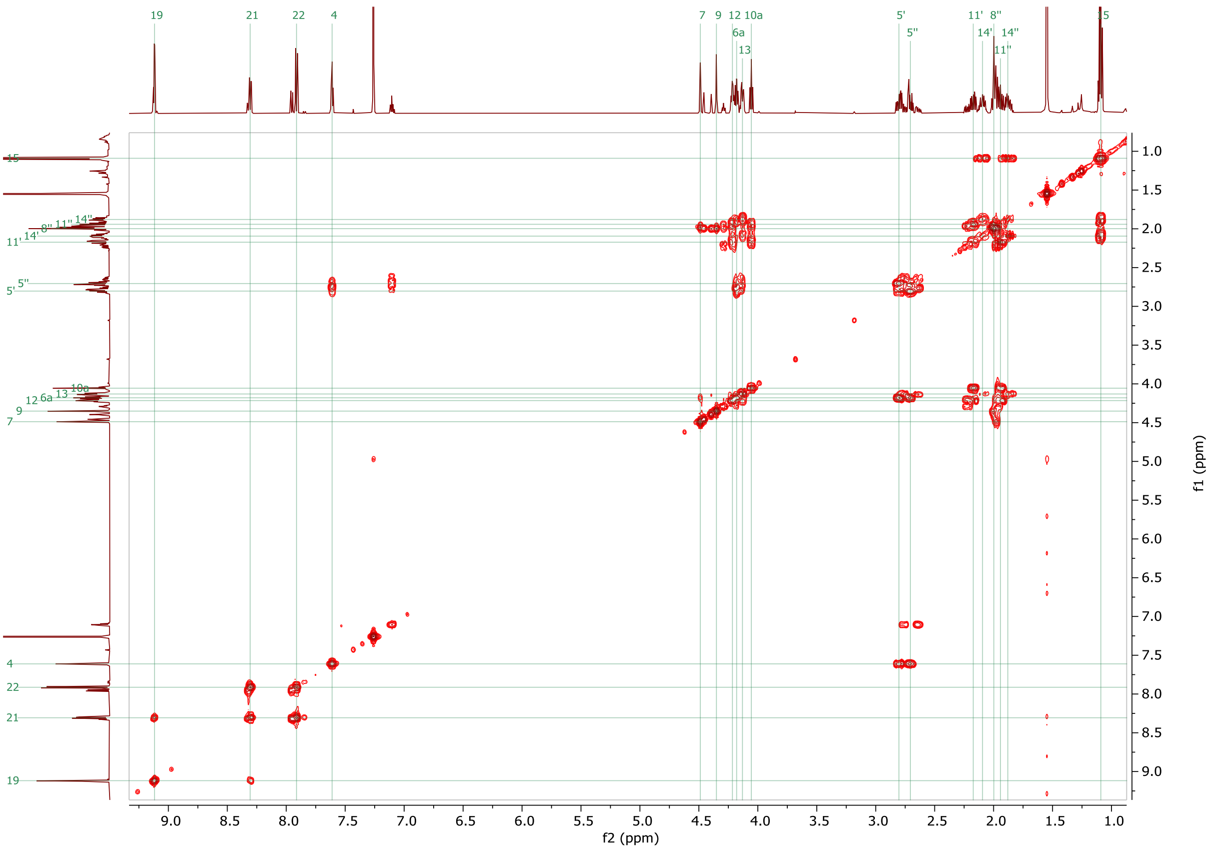

HSQC of 18

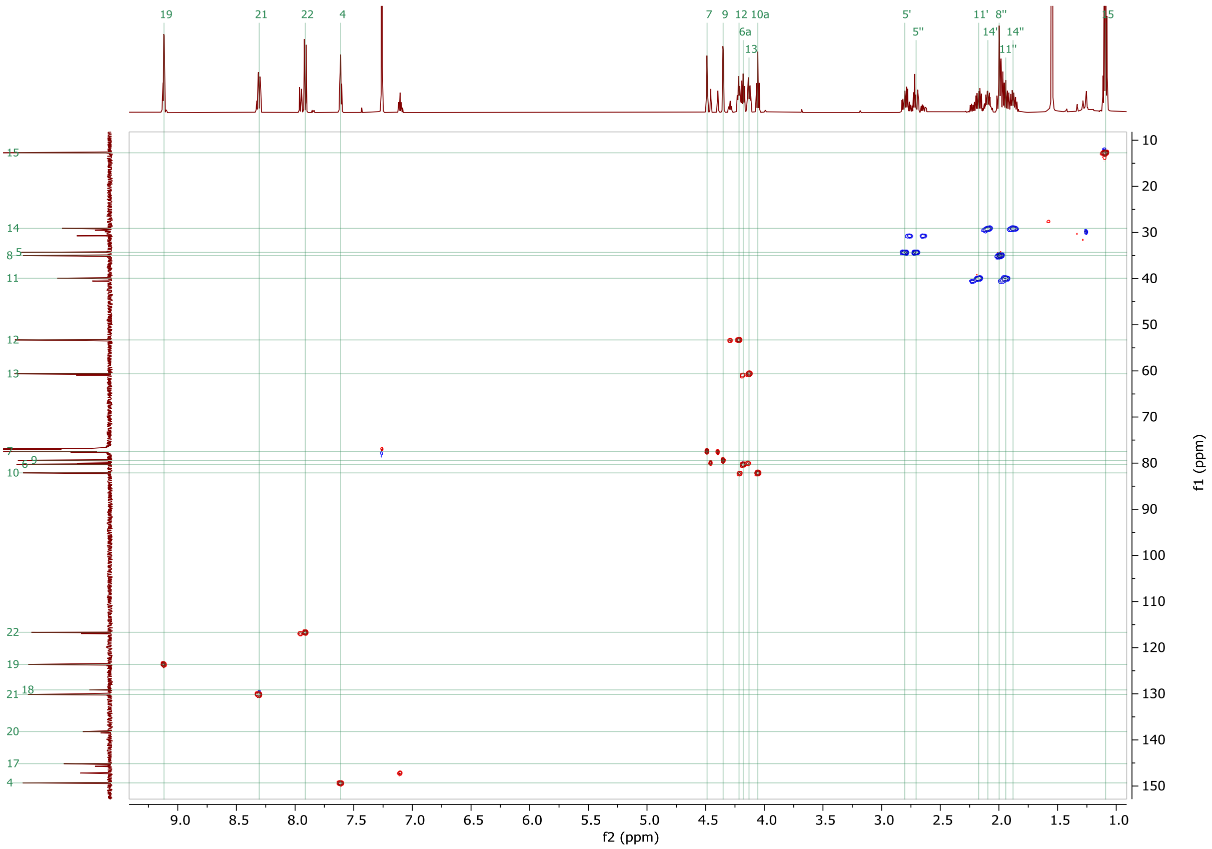

HMBC of 18

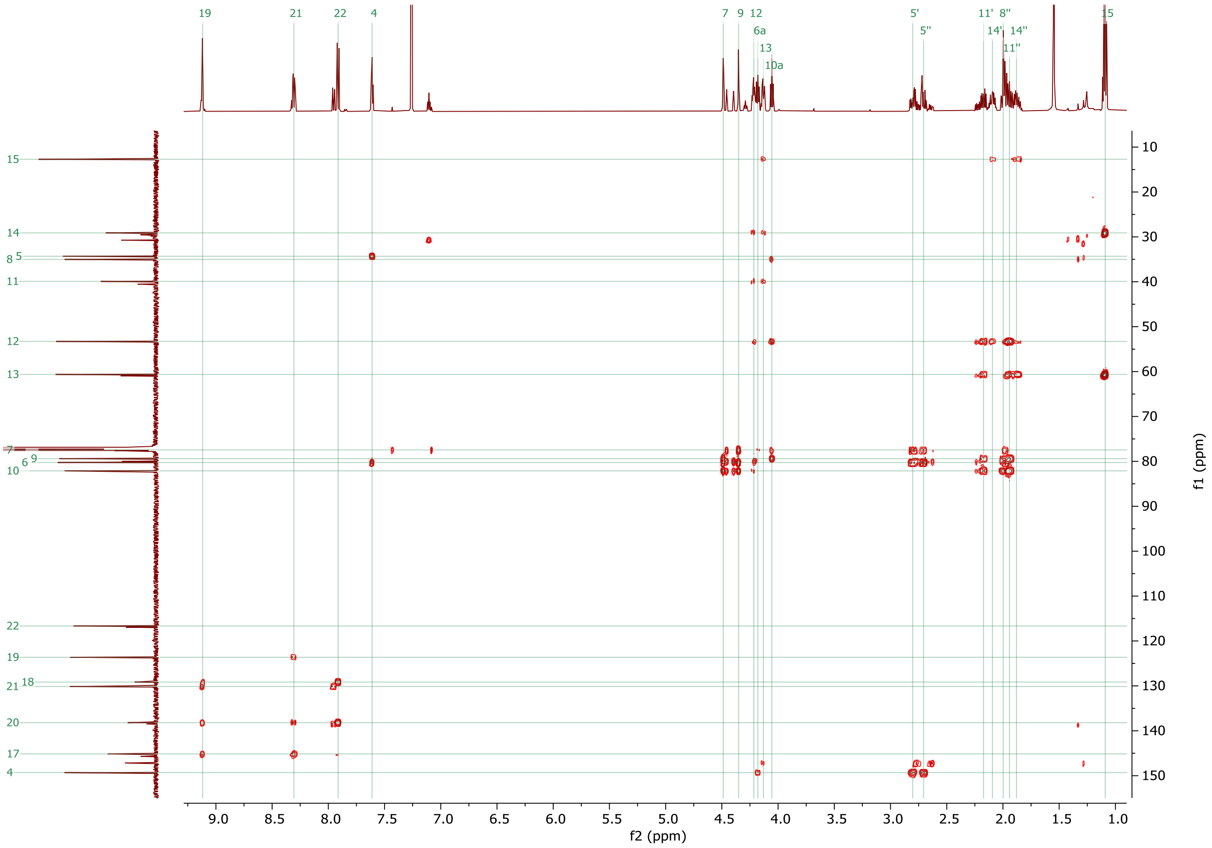

^1H-^1H NOESY of 18

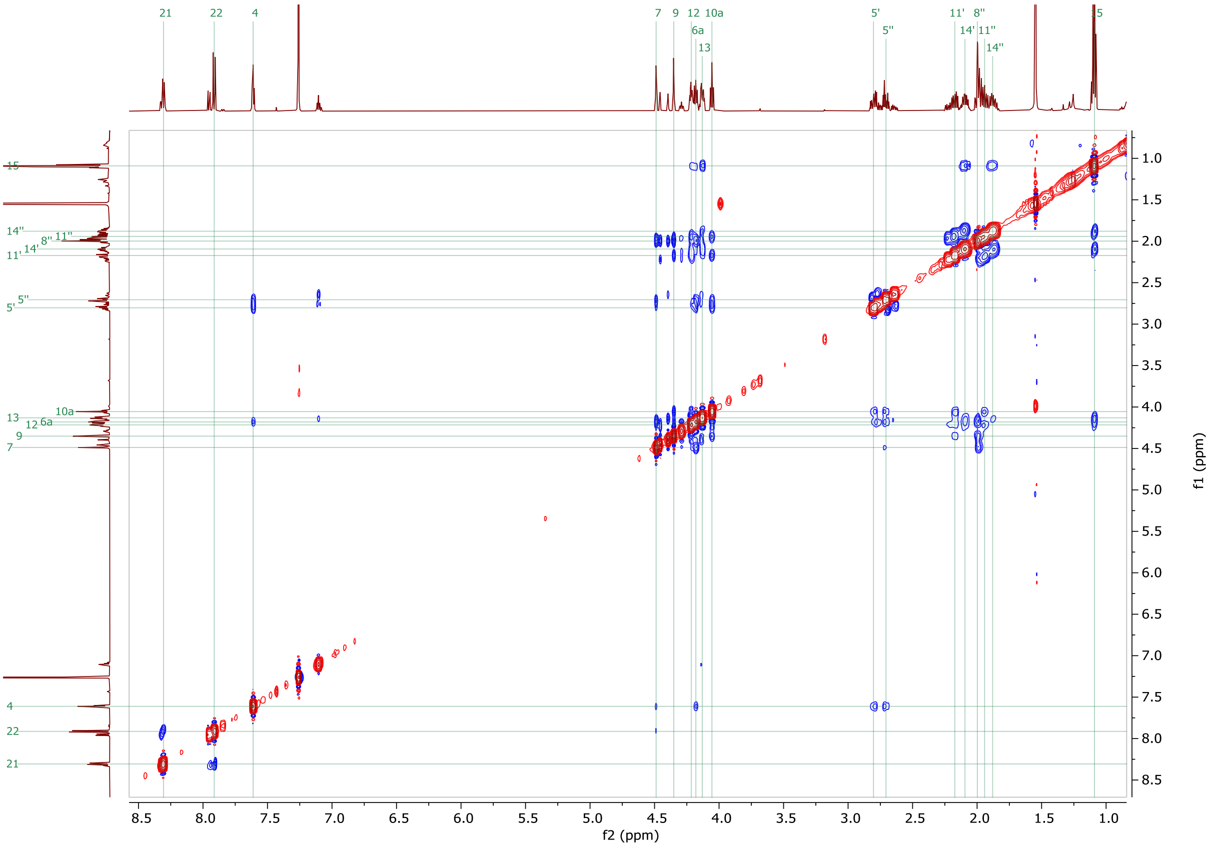

2-((1S,3S,4S,6R)-6-((2R,3R)-2,3-Dibromopentyl)-2,5-dioxabicyclo[2.2.1]heptan-3-yl)acetaldehyde (S3)

^{1}H NMR (600 MHz, $CDCl_3$)

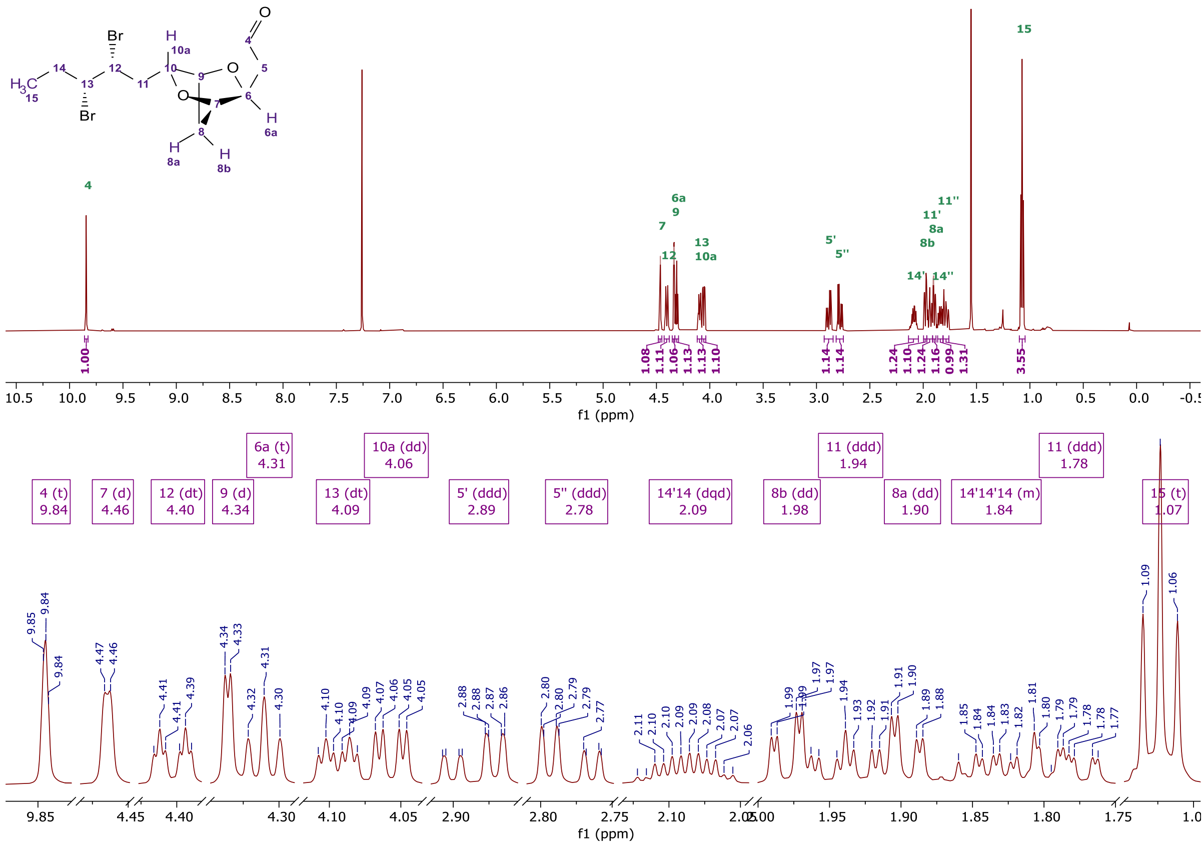

^{13}C NMR (151 MHz, $CDCl_3$)

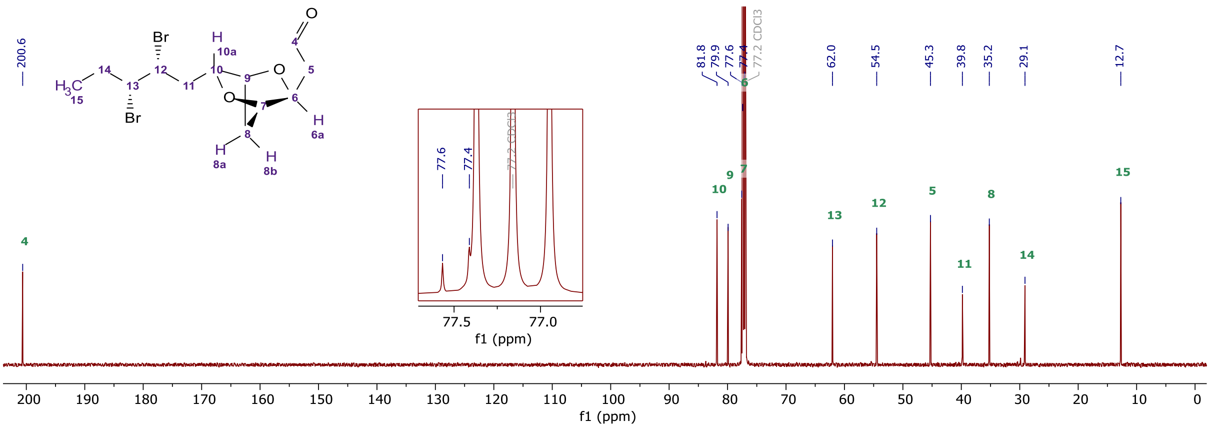

^{1}H-^{1}H COSY of S3

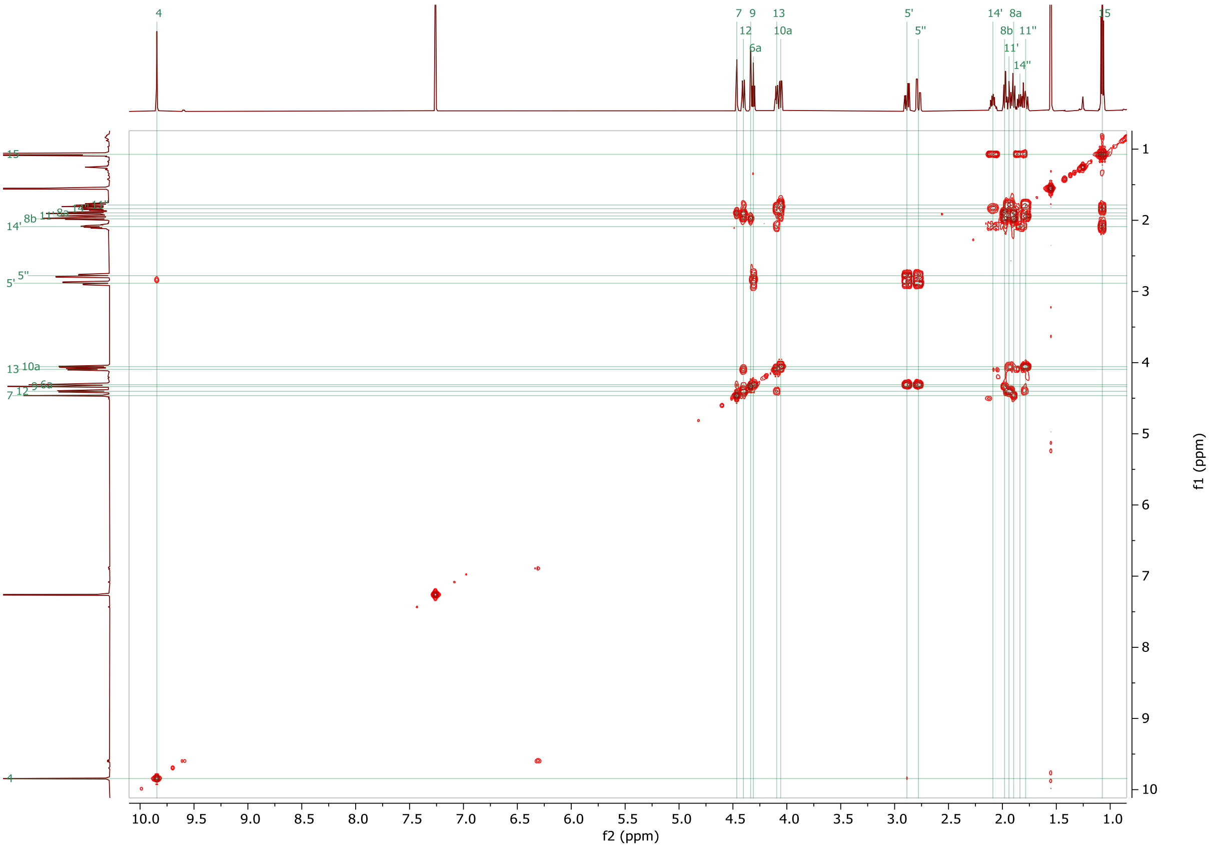

HSQC of S3

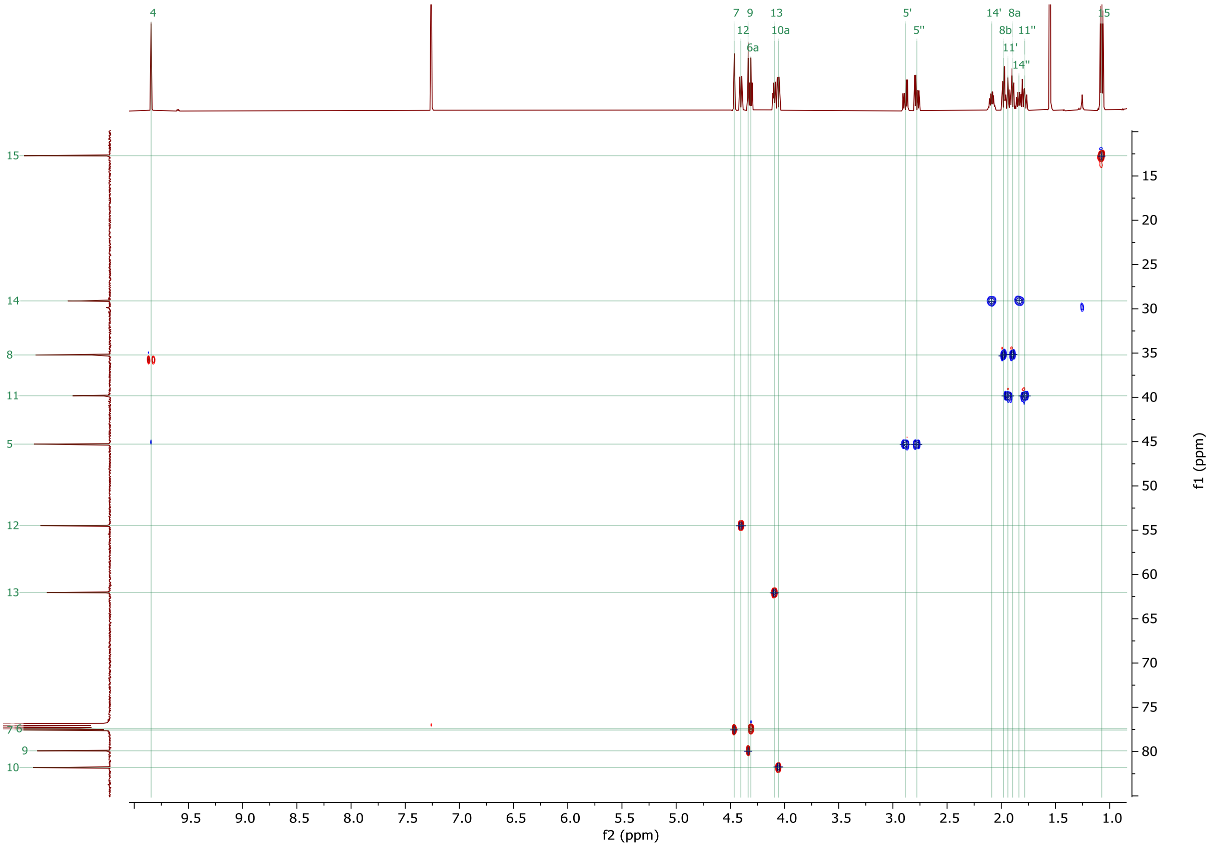

HMBC of S3

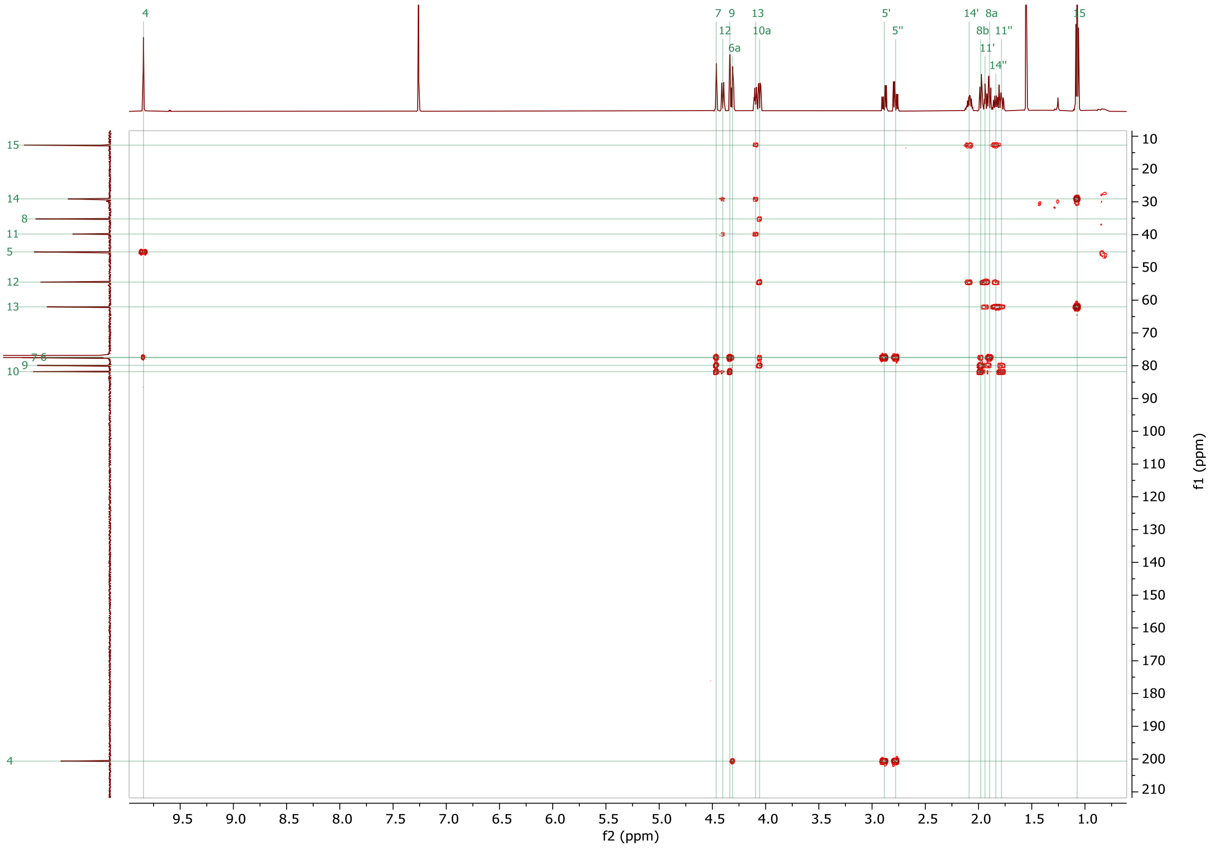

^{1}H-^{1}H NOESY of S3

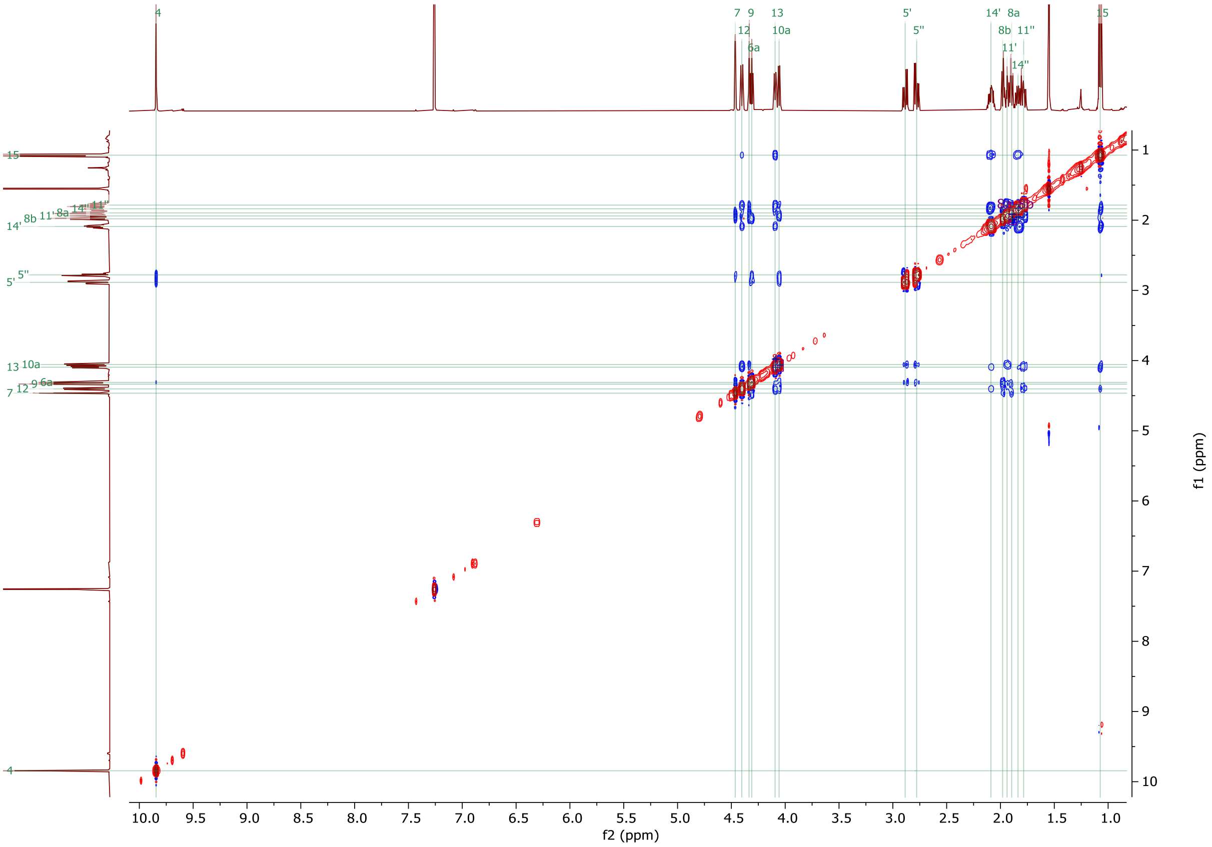

(*E*)-3-((2*S*,4*S*,5*R*)-5-((2*R*,3*R*)-2,3-Dibromopentyl)-4-hydroxytetrahydrofuran-2-yl)acrylaldehyde (S4)

^{1}H NMR (600 MHz, $CDCl_3$)

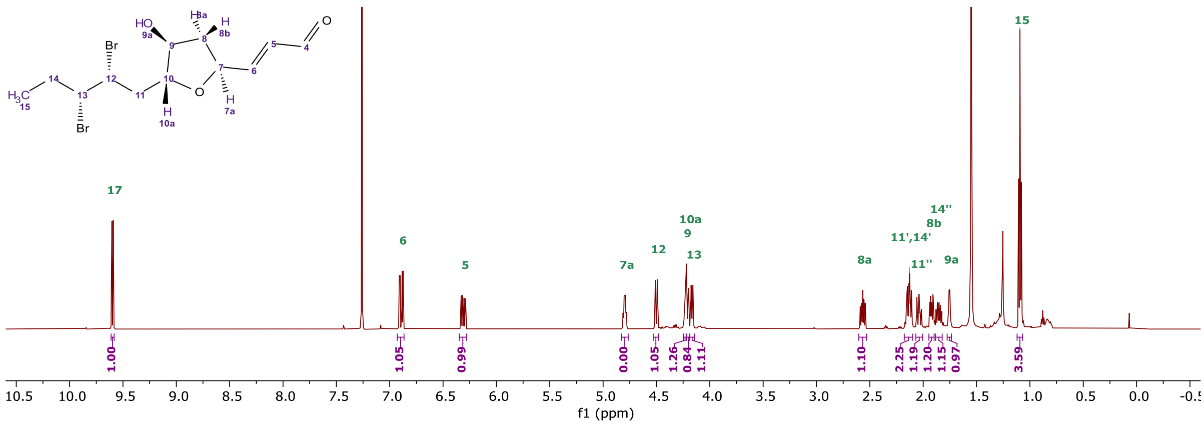

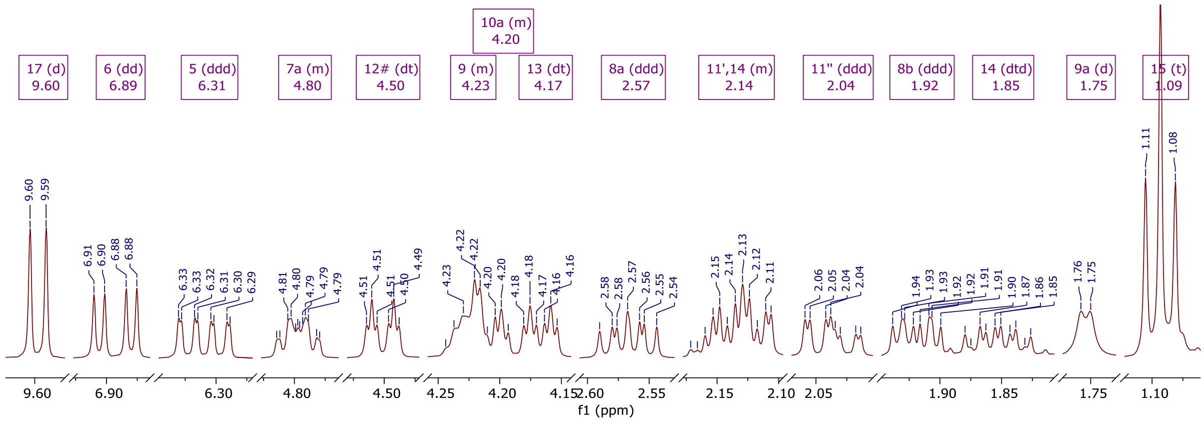

^{13}C NMR (151 MHz, $CDCl_3$)

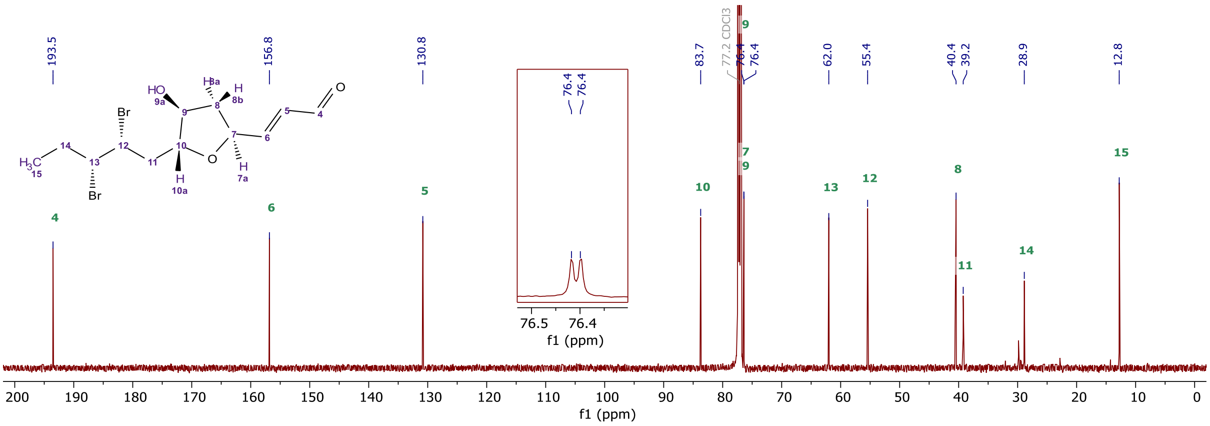

^{1}H-^{1}H COSY of S4

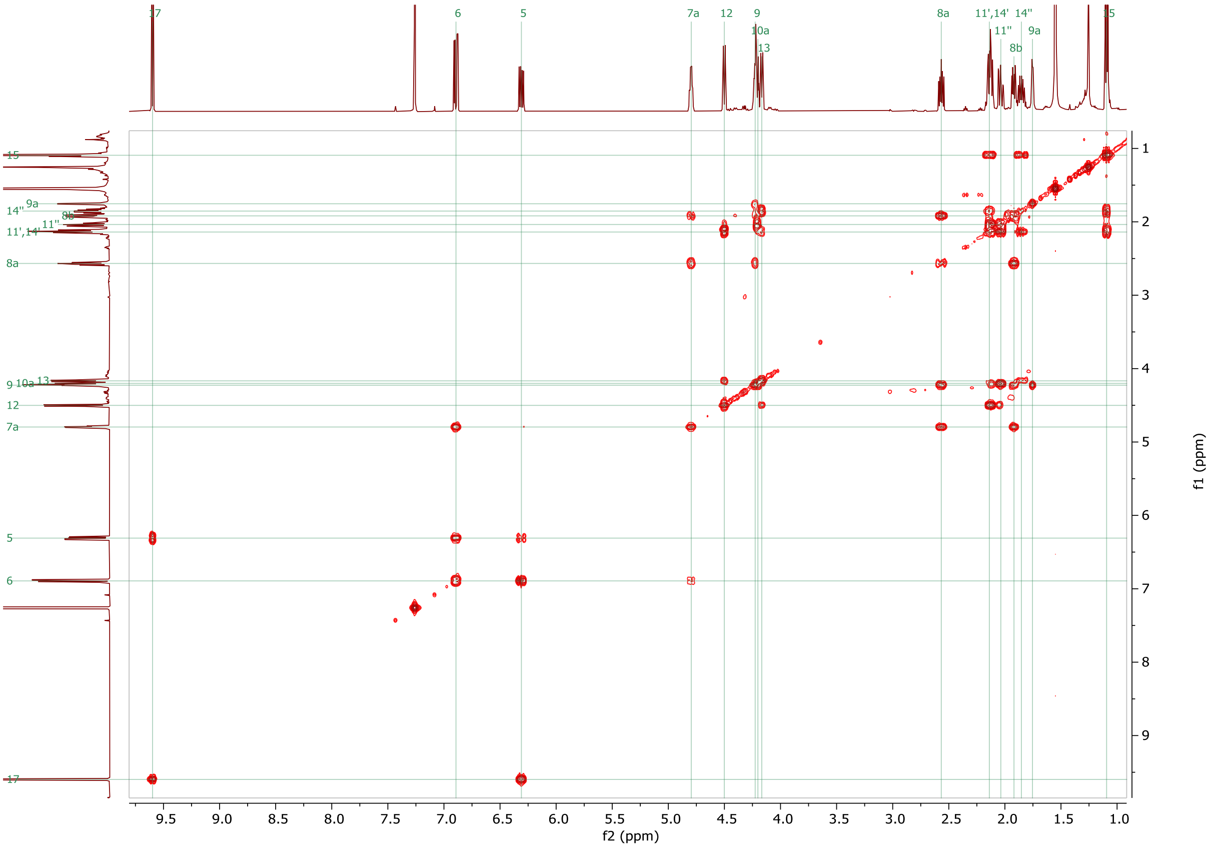

HSQC of S4

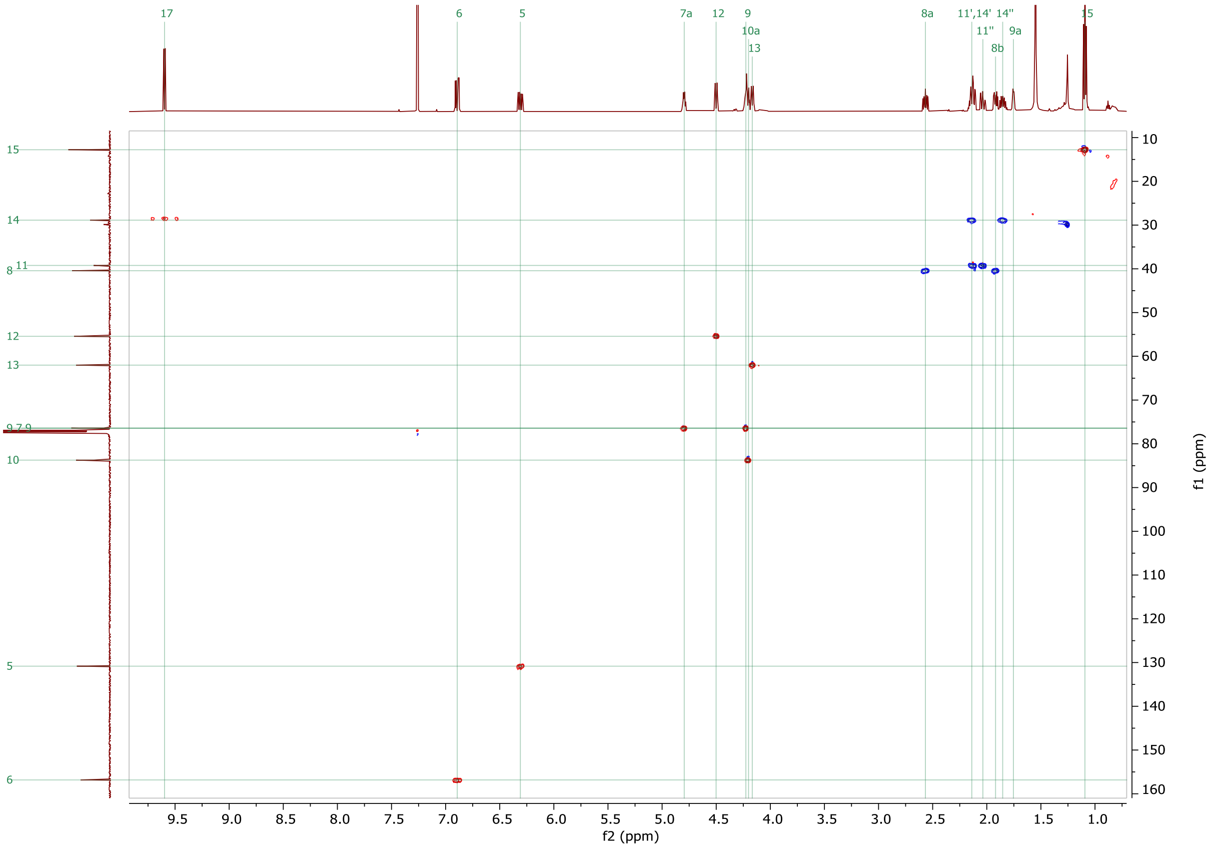

HMBC of S4

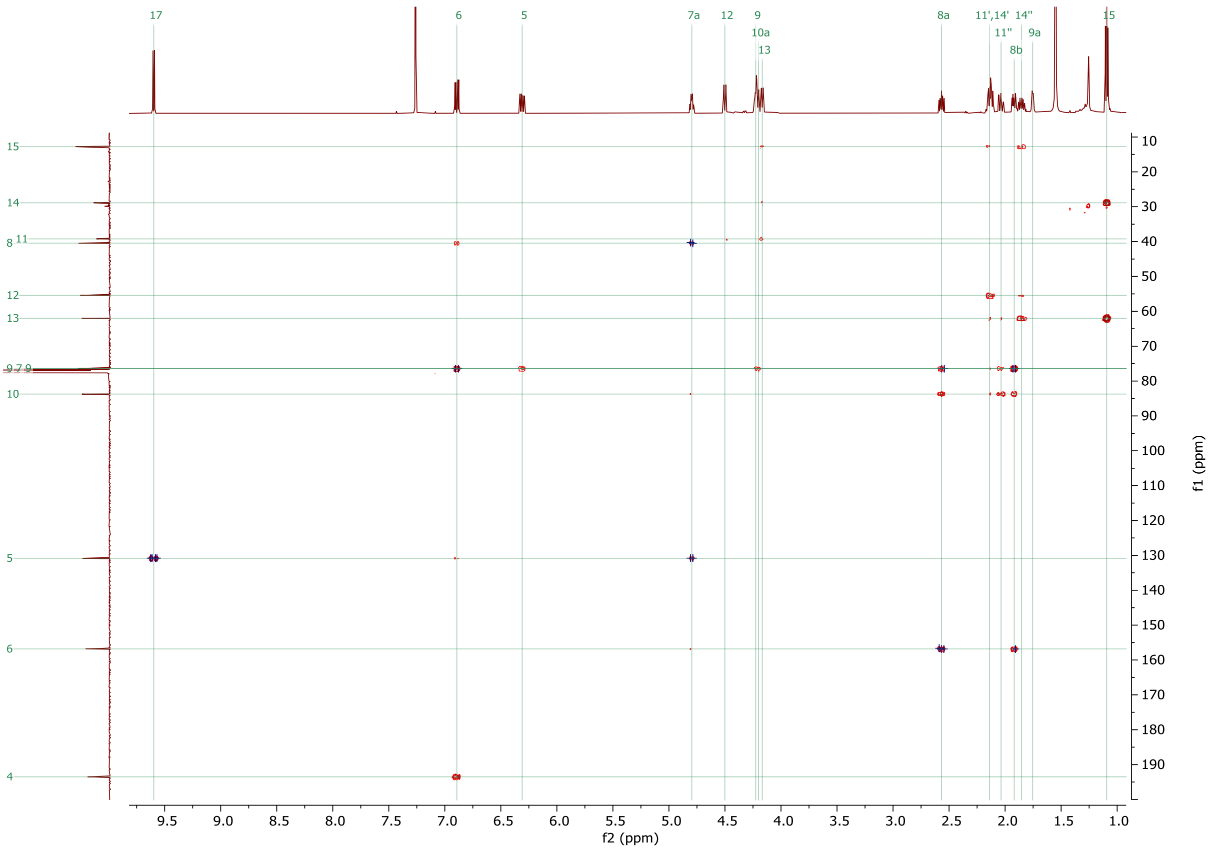

^{1}H-^{1}H NOESY of S4

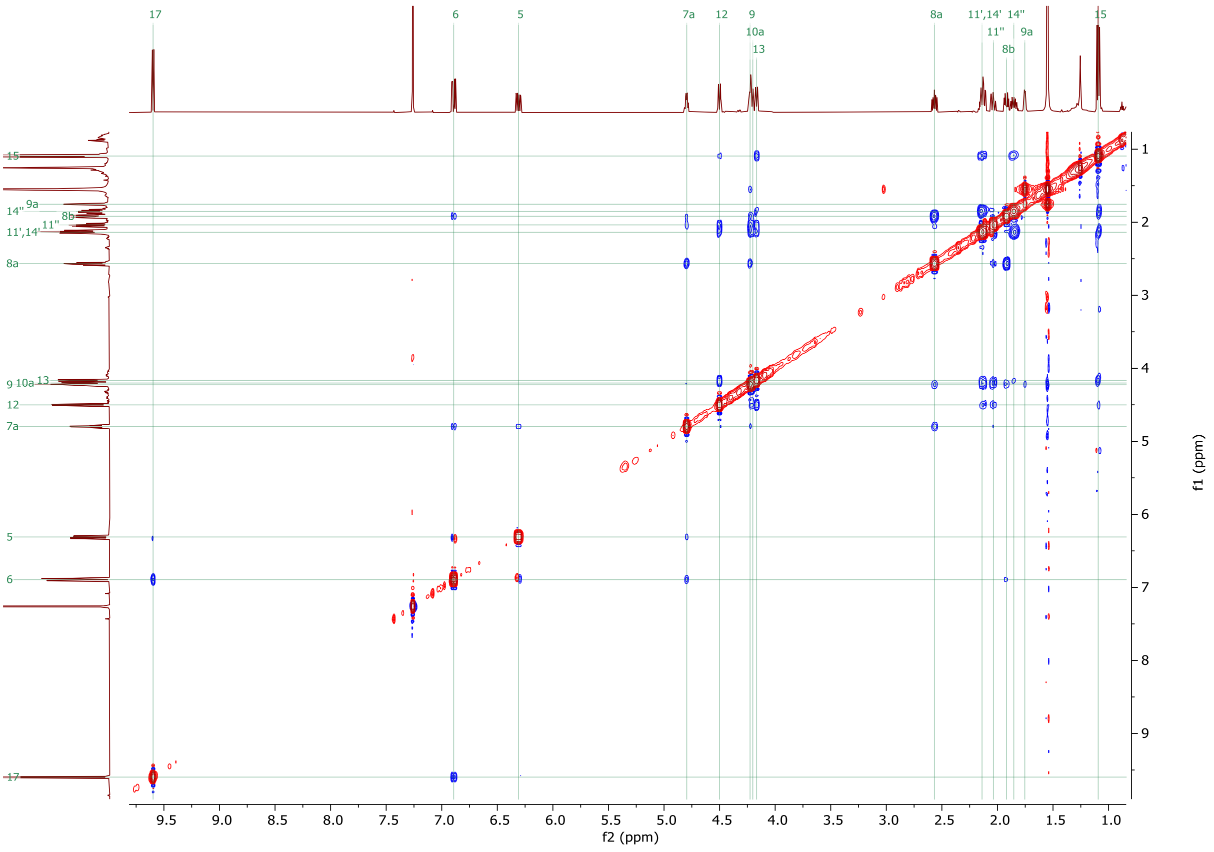

(*E*)-4-((1*S*,3*S*,4*S*,6*R*)-6-((2*R*,3*R*)-2,3-Dibromopentyl)-2,5-dioxabicyclo[2.2.1]heptan-3-yl)but-2-enal (S5)

^{1}H NMR (600 MHz, $CDCl_3$)

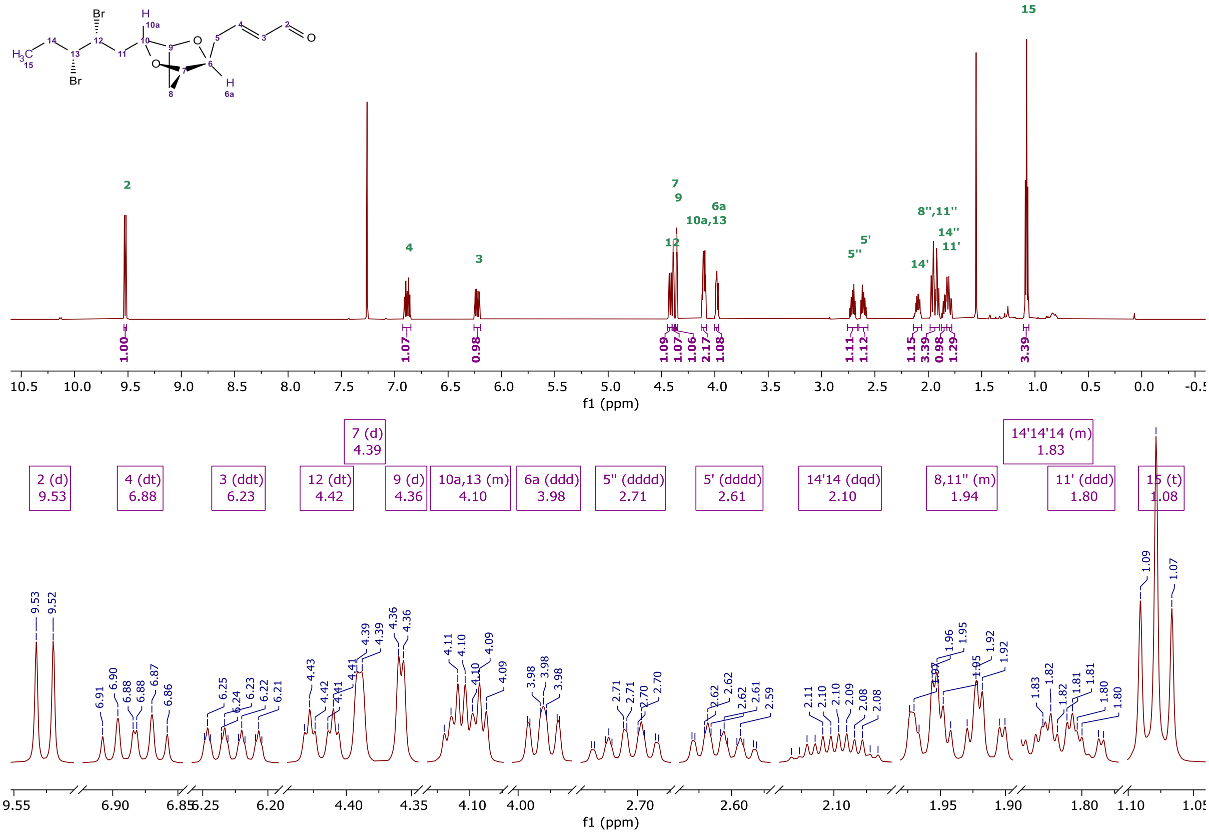

^{13}C NMR (151 MHz, $CDCl_3$)

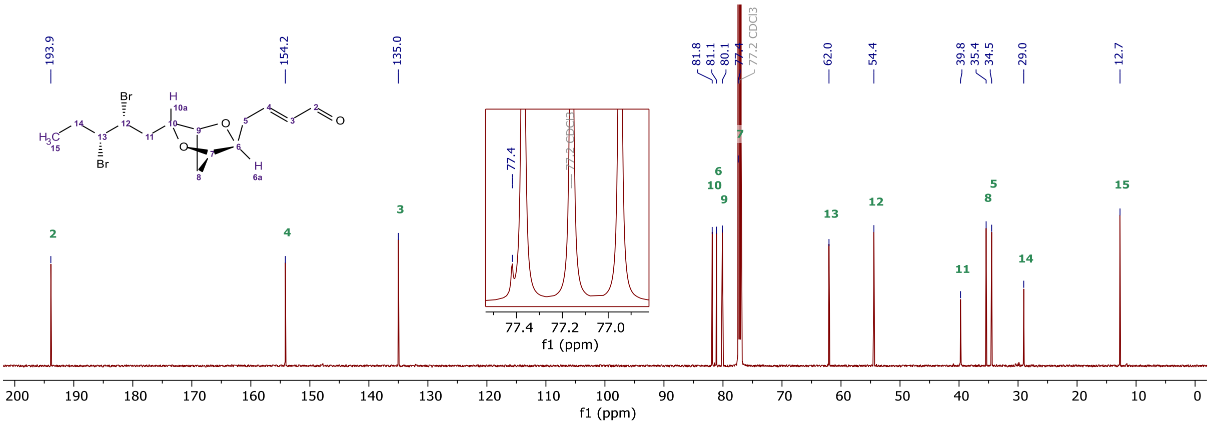

^{1}H-^{1}H COSY of S5

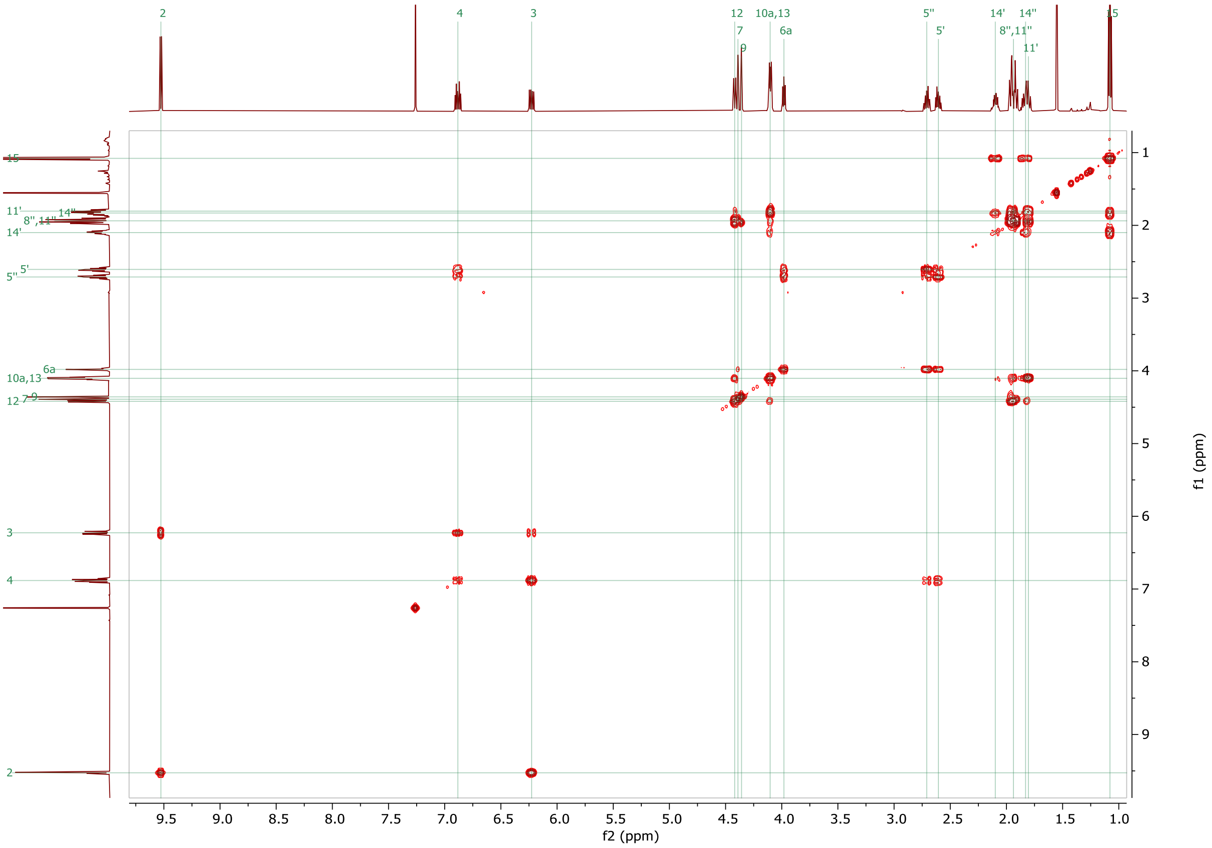

HSQC of S5

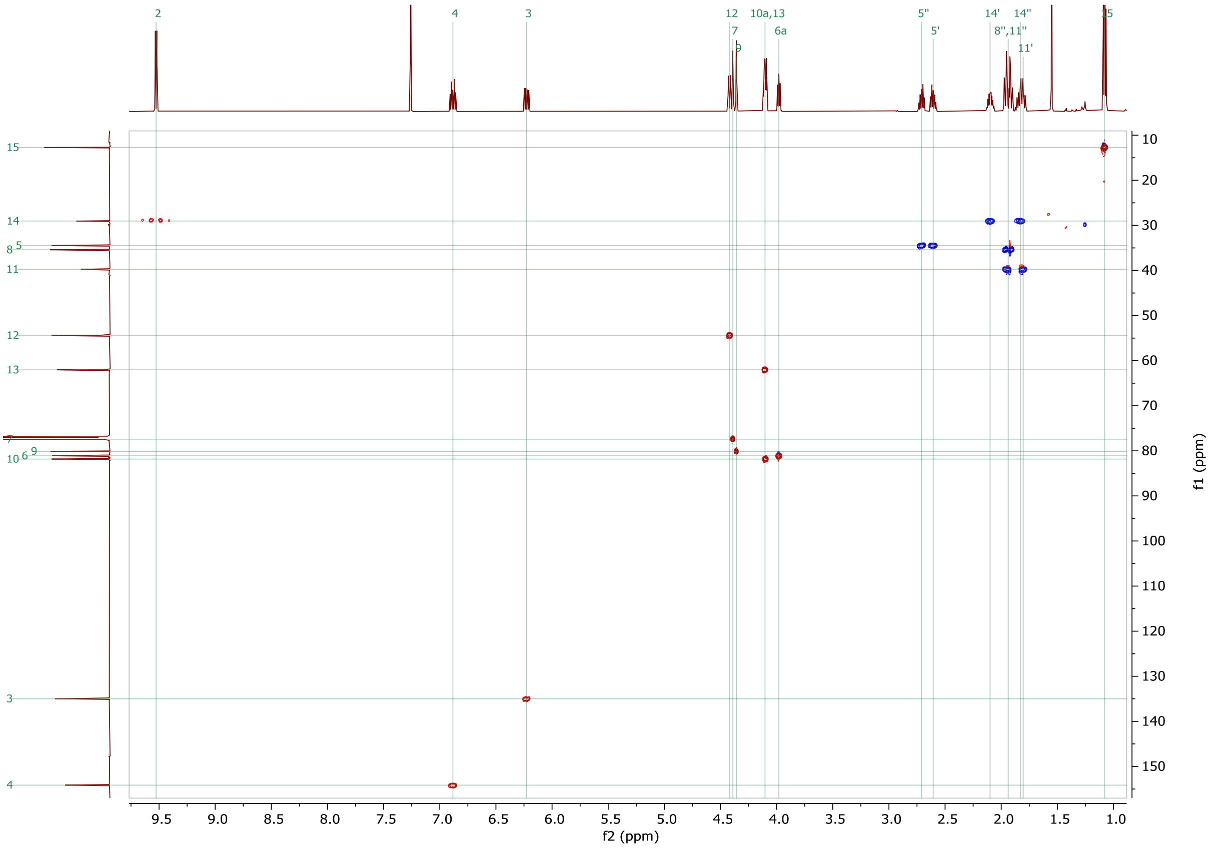

HMBC of S5

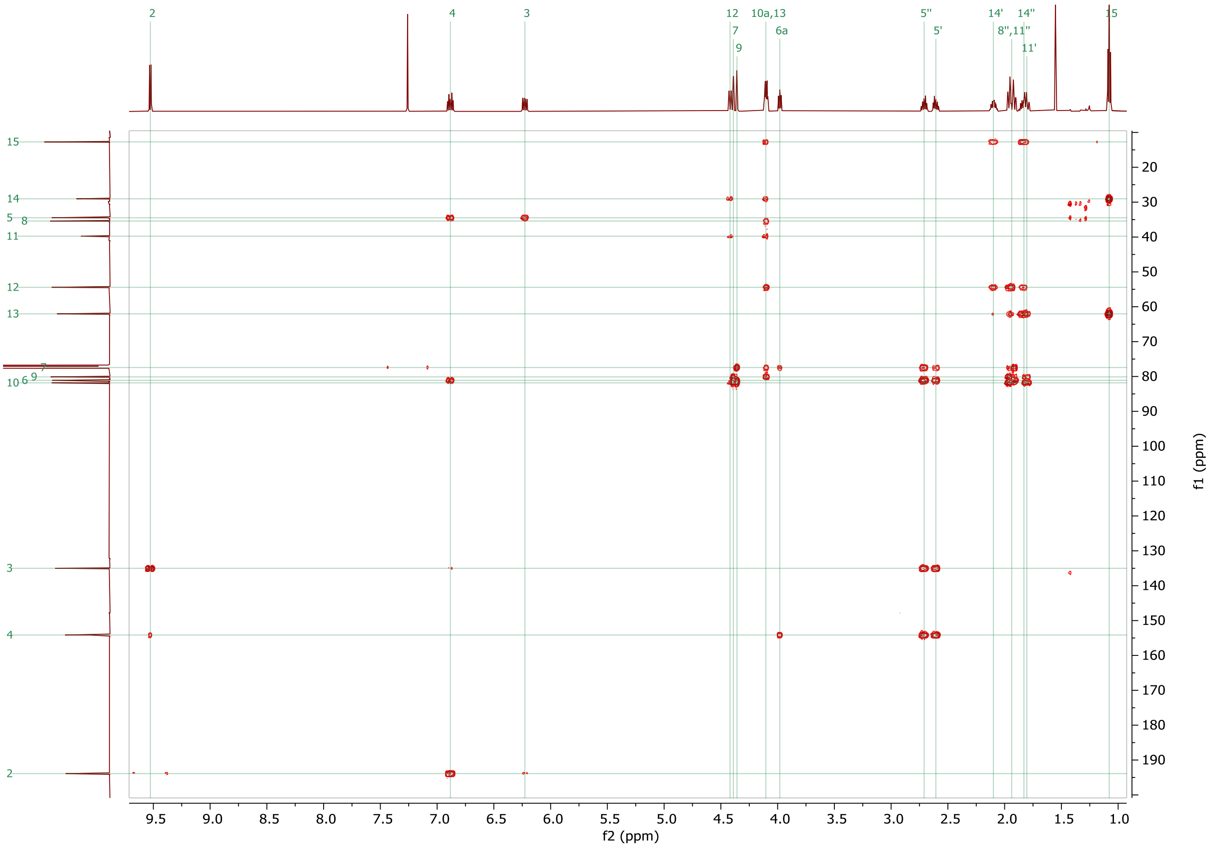

^{1}H-^{1}H NOESY of S5

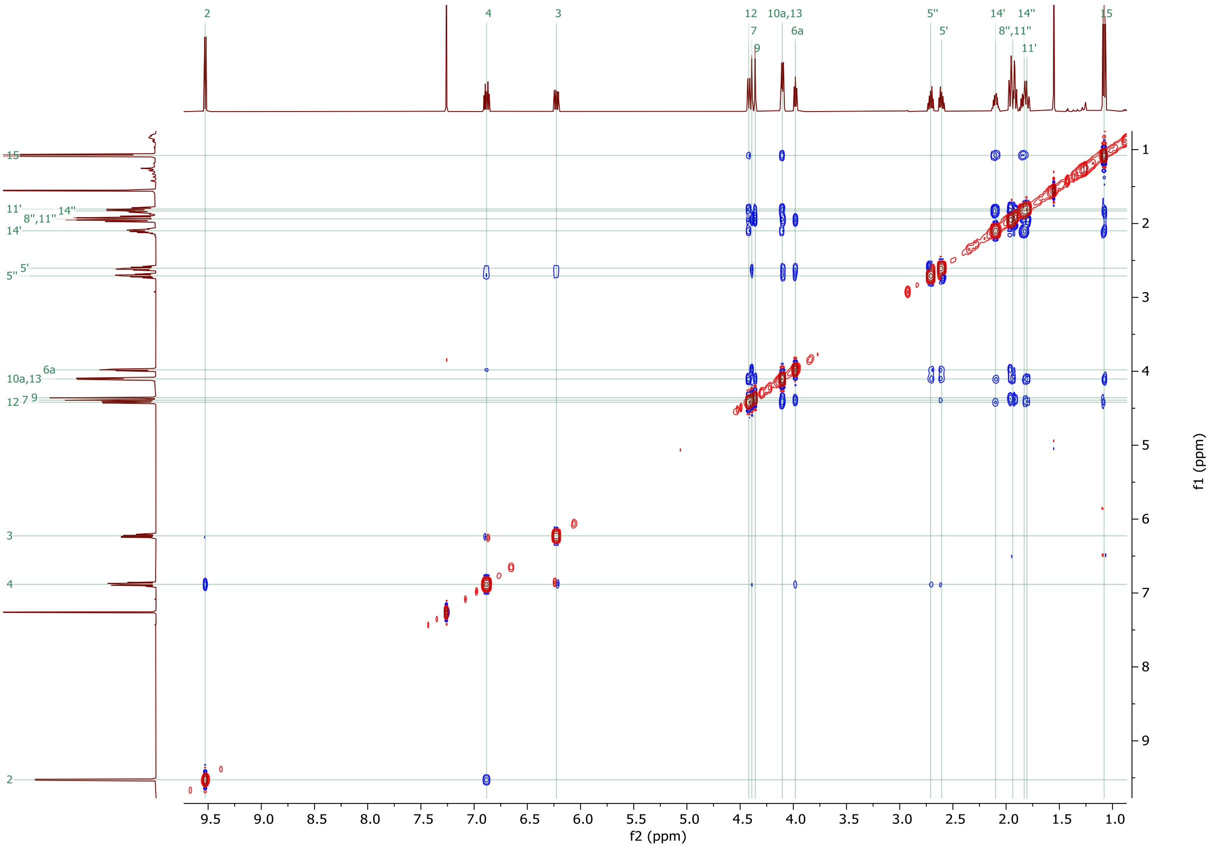

(*E*)-4-((1*S*,3*S*,4*S*,6*R*)-6-((2*S*,3*S*)-2,3-Dibromopentyl)-2,5-dioxabicyclo[2.2.1]heptan-3-yl)but-2-enal (S6)

^{1}H NMR (600 MHz, $CDCl_3$)

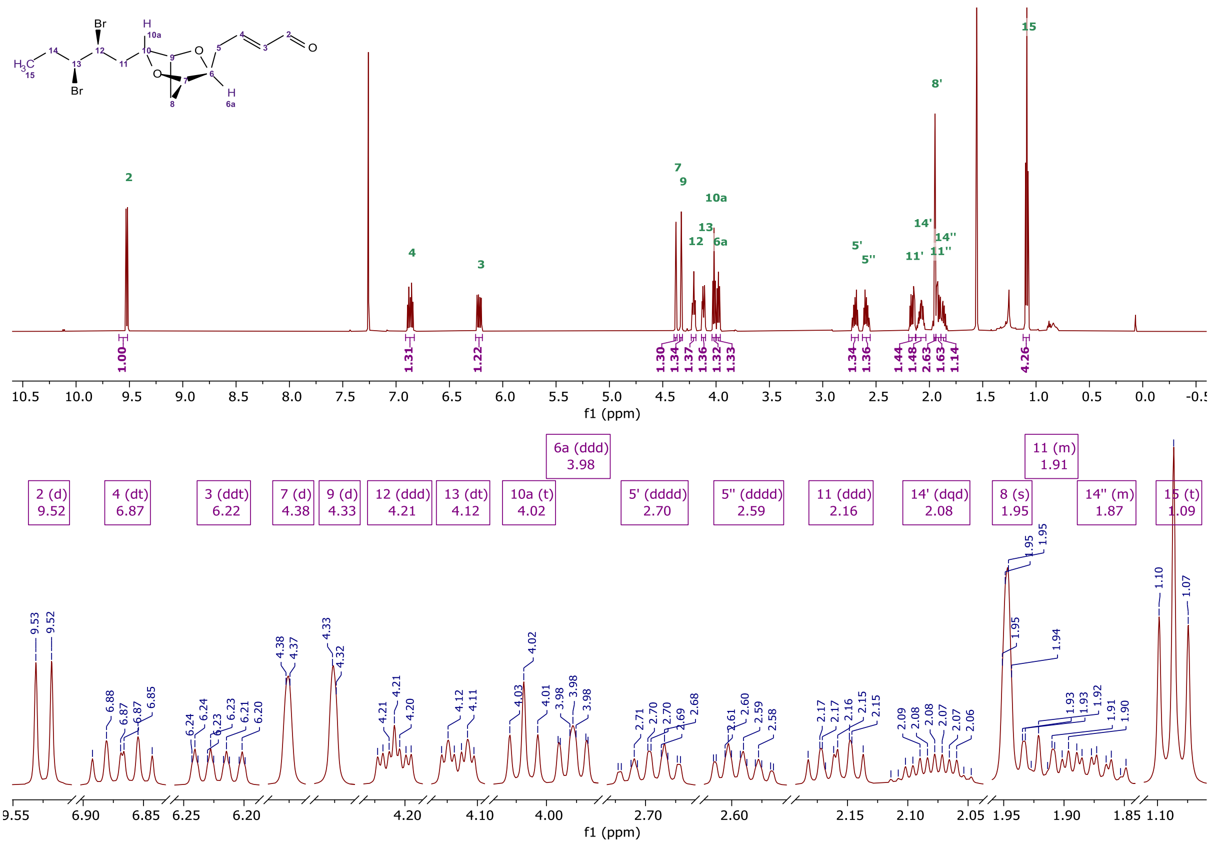

^{13}C NMR (151 MHz, $CDCl_3$)

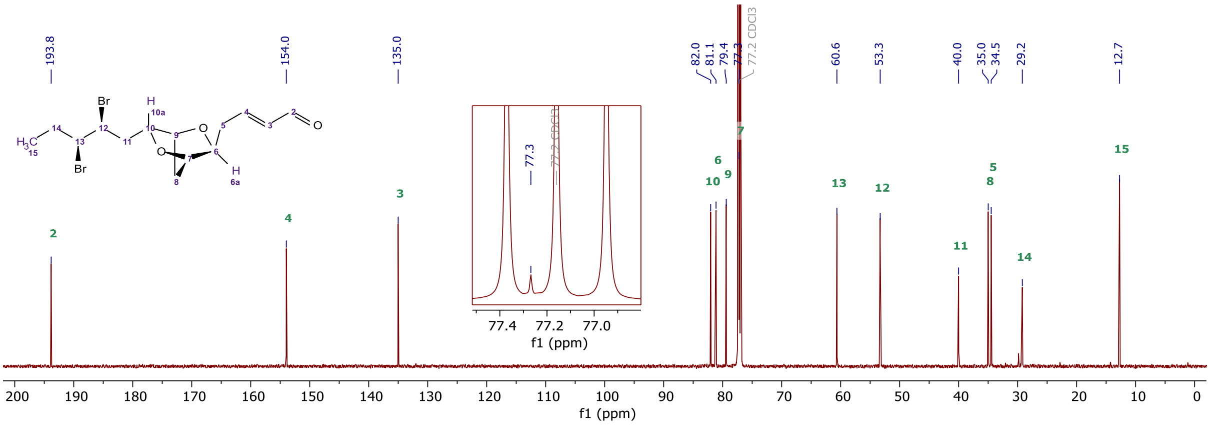

^1H-^1H COSY of S6

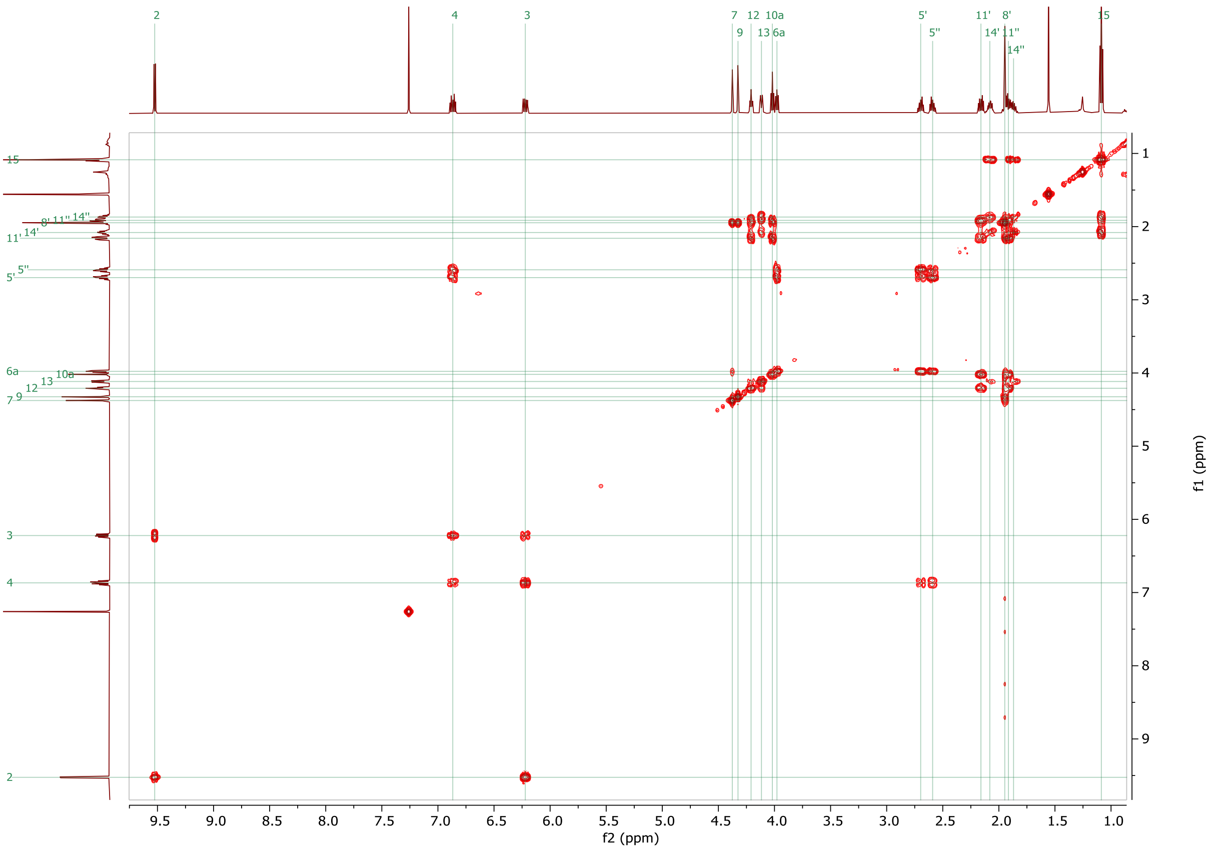

HSQC of S6

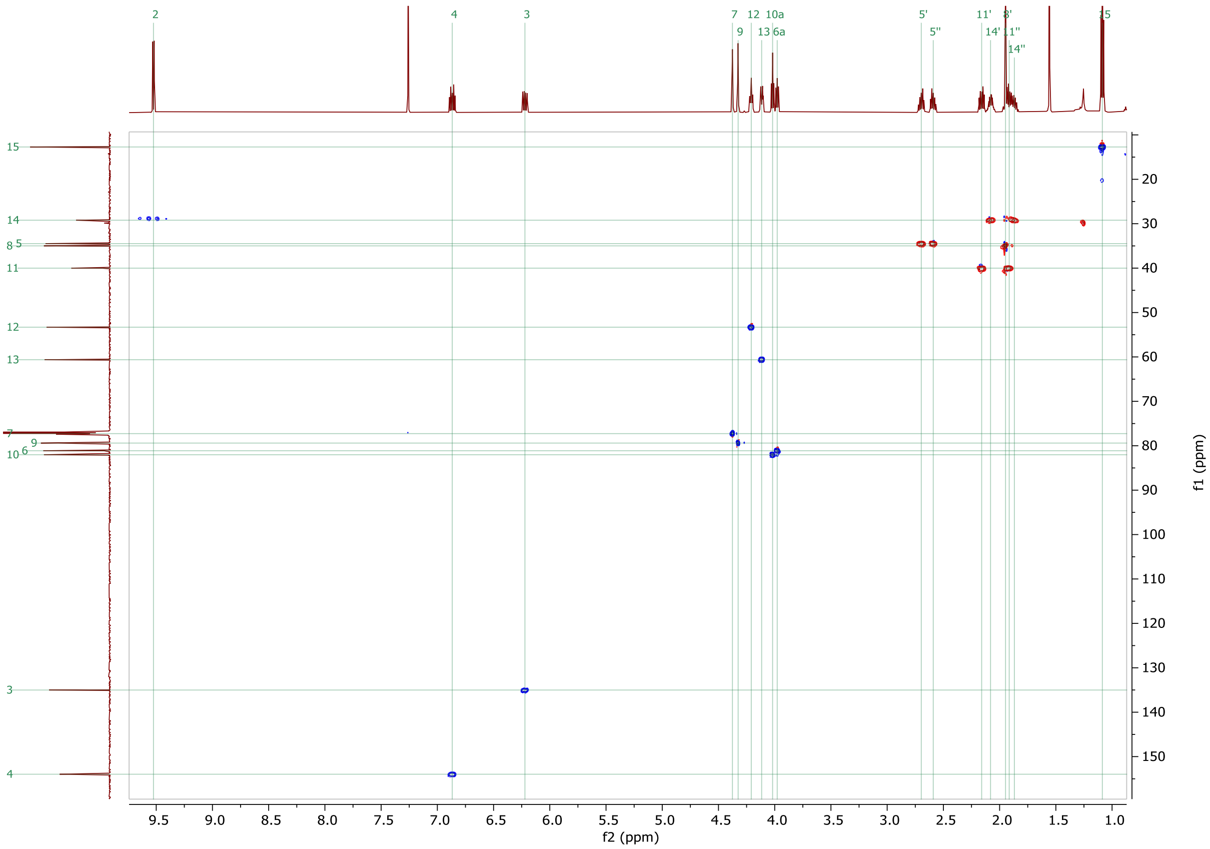

HMBC of S6

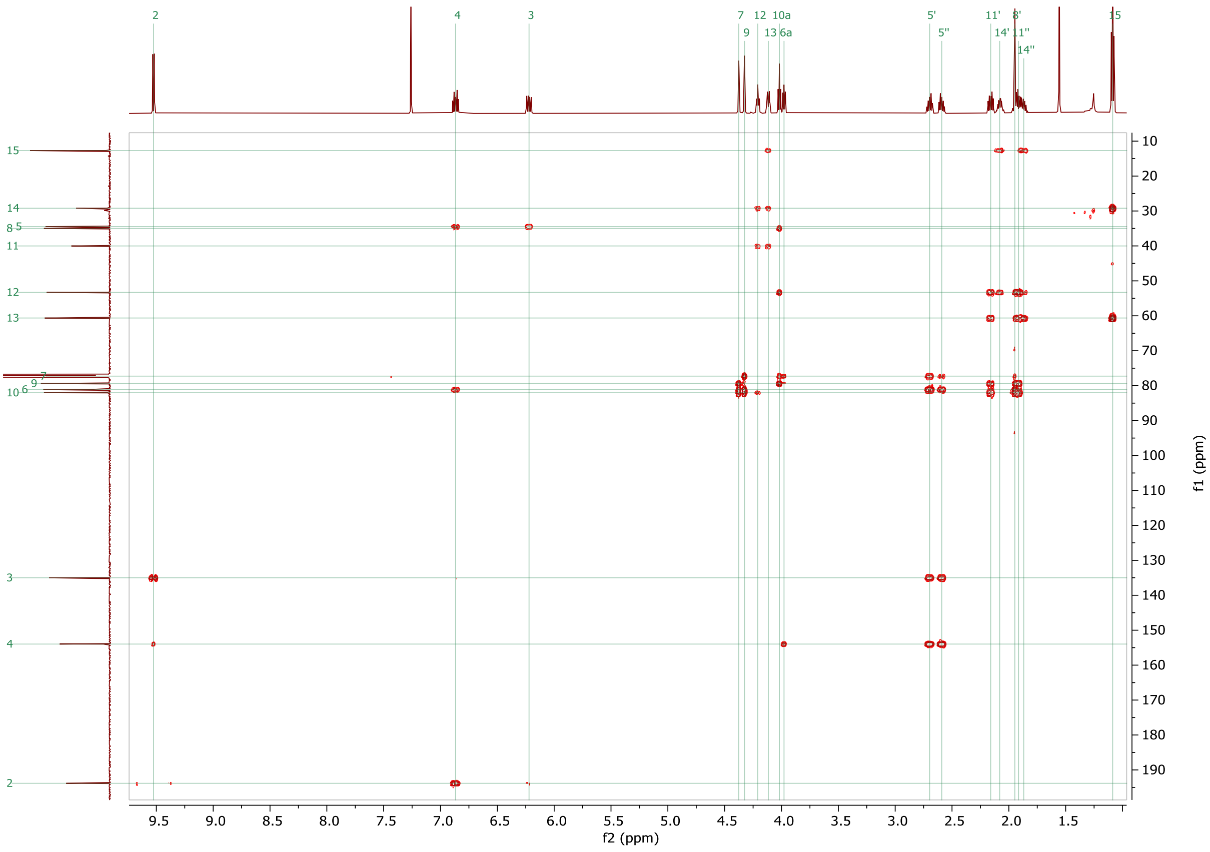

^{1}H-^{1}H NOESY of S6

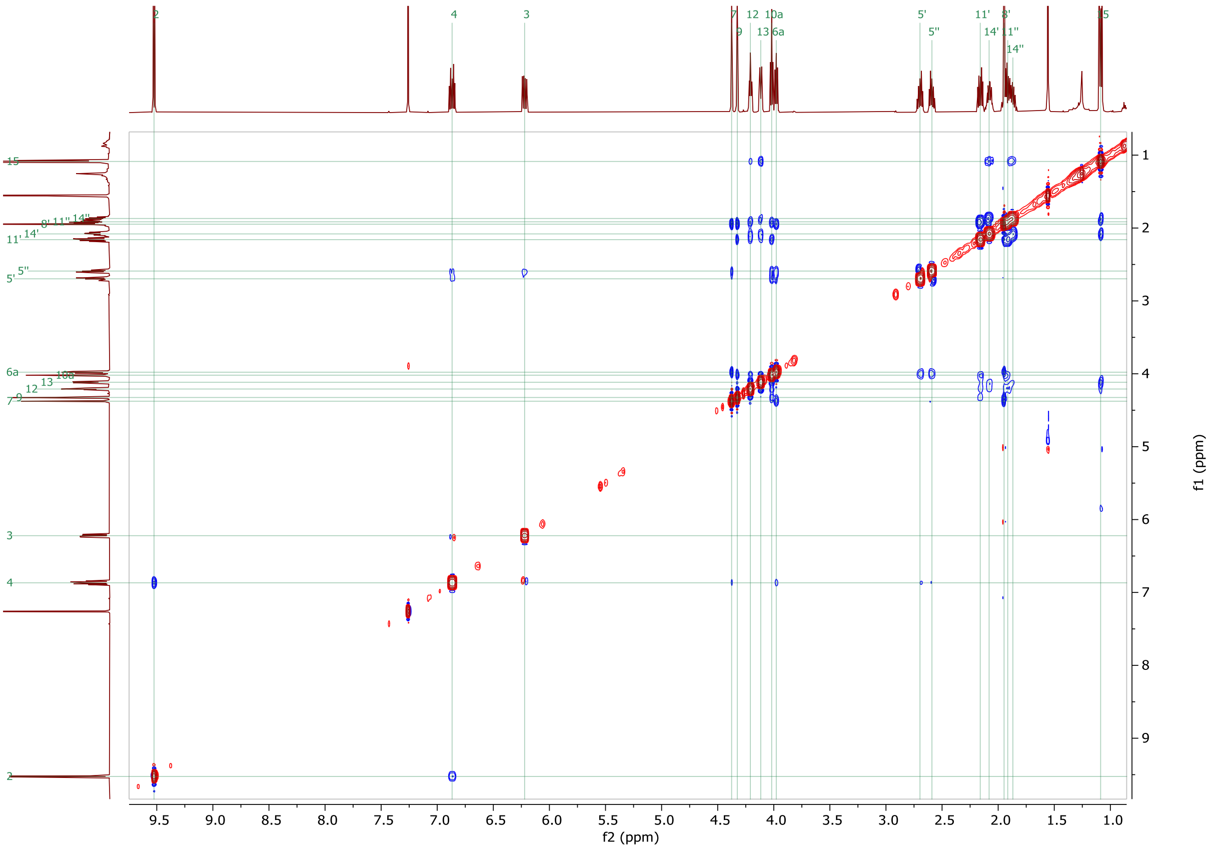

(1S,3R,4S,6S)-3-((2R,3R)-2,3-Dibromopentyl)-6-((E)-pent-2-en-4-yn-1-yl)-2,5-dioxabicyclo[2.2.1]heptane (ent-(E)-5)

^{1}H NMR (600 MHz, $CDCl_3$)

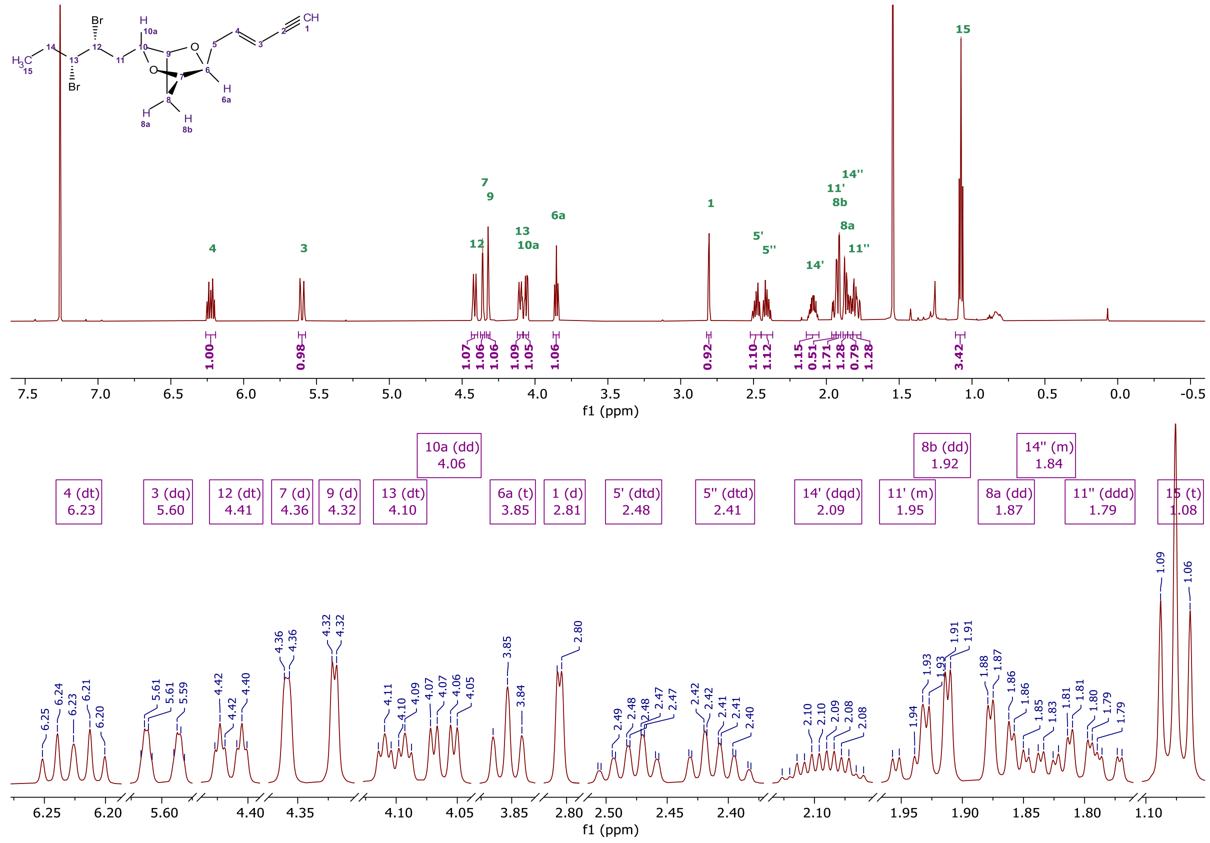

^{1}H NMR (400 MHz, $CDCl_3$) of *ent*-(*E*)-5

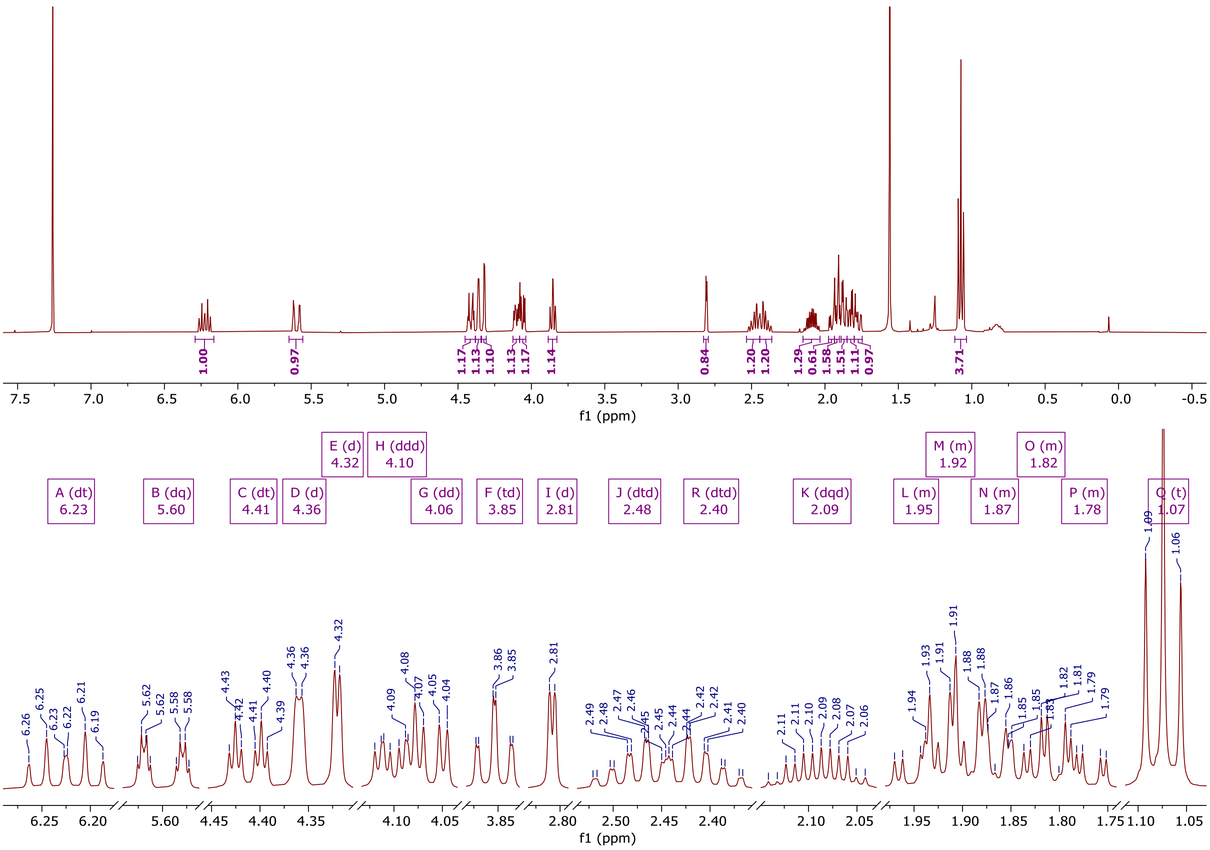

^{13}C NMR (151 MHz, $CDCl_3$)

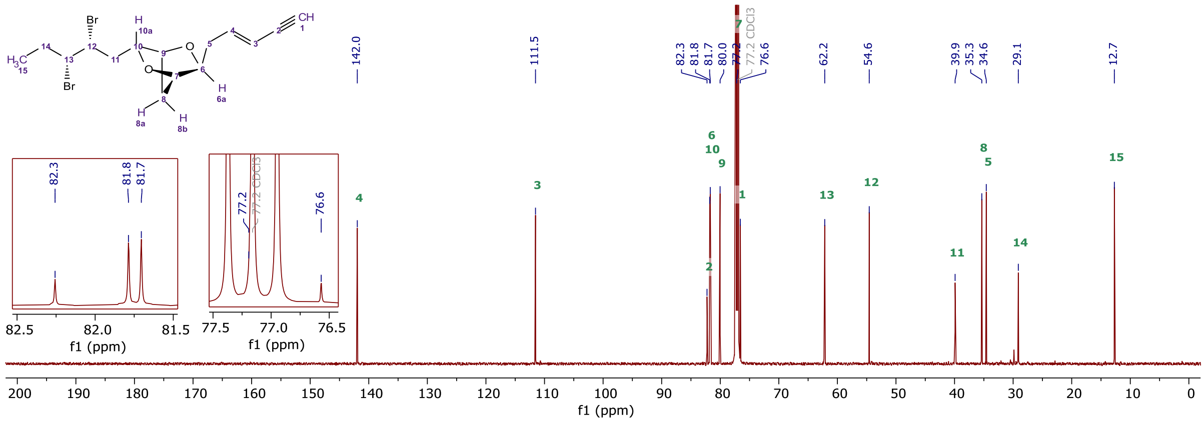

^{1}H-^{1}H COSY of *ent*-(*E*)-5

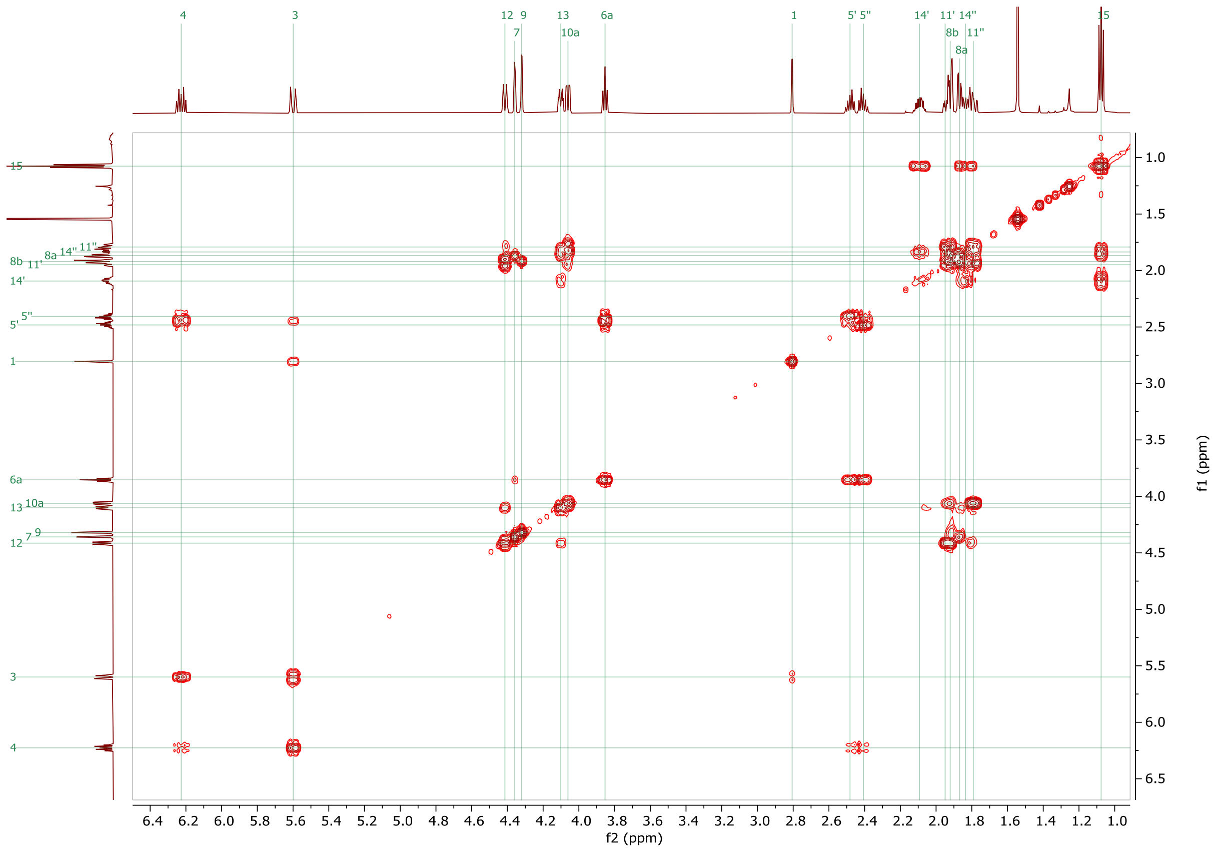

HSQC of *ent*-(*E*)-5

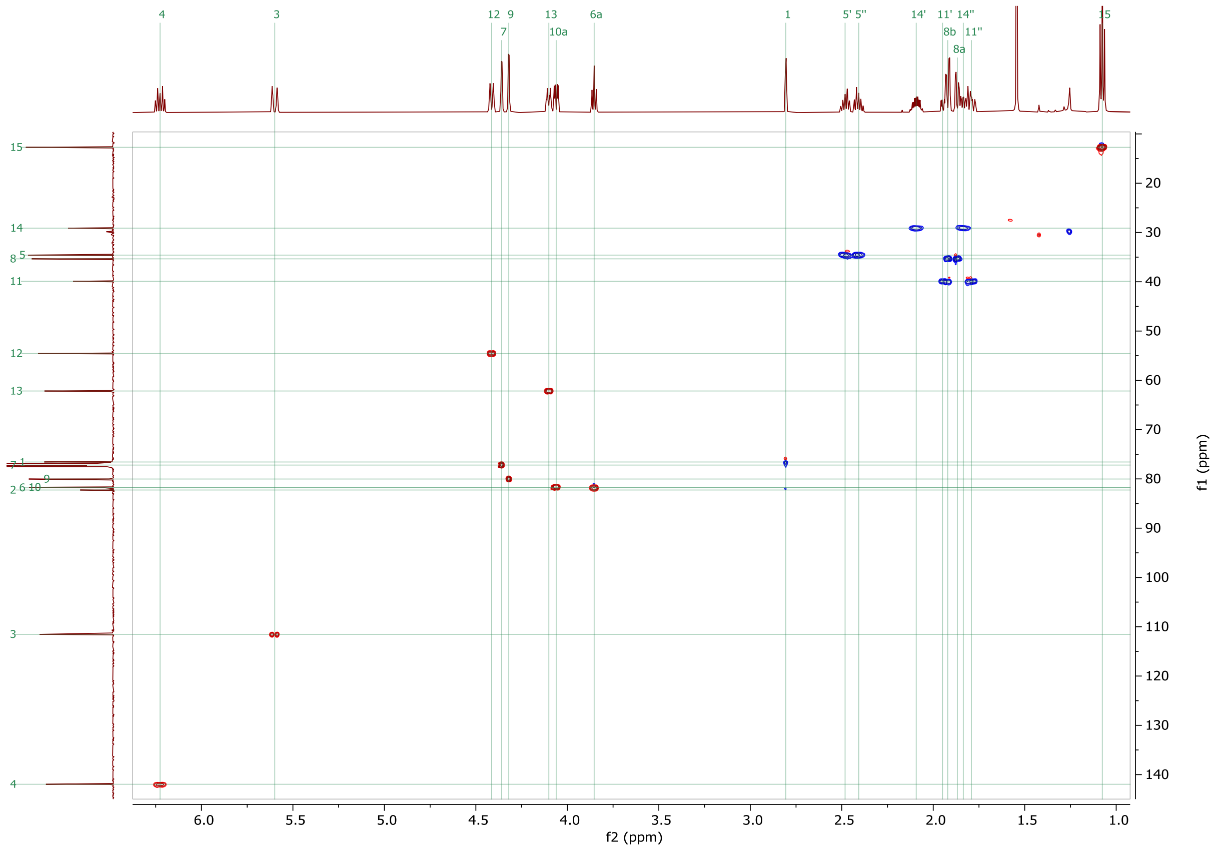

HMBC of *ent*-(*E*)-5

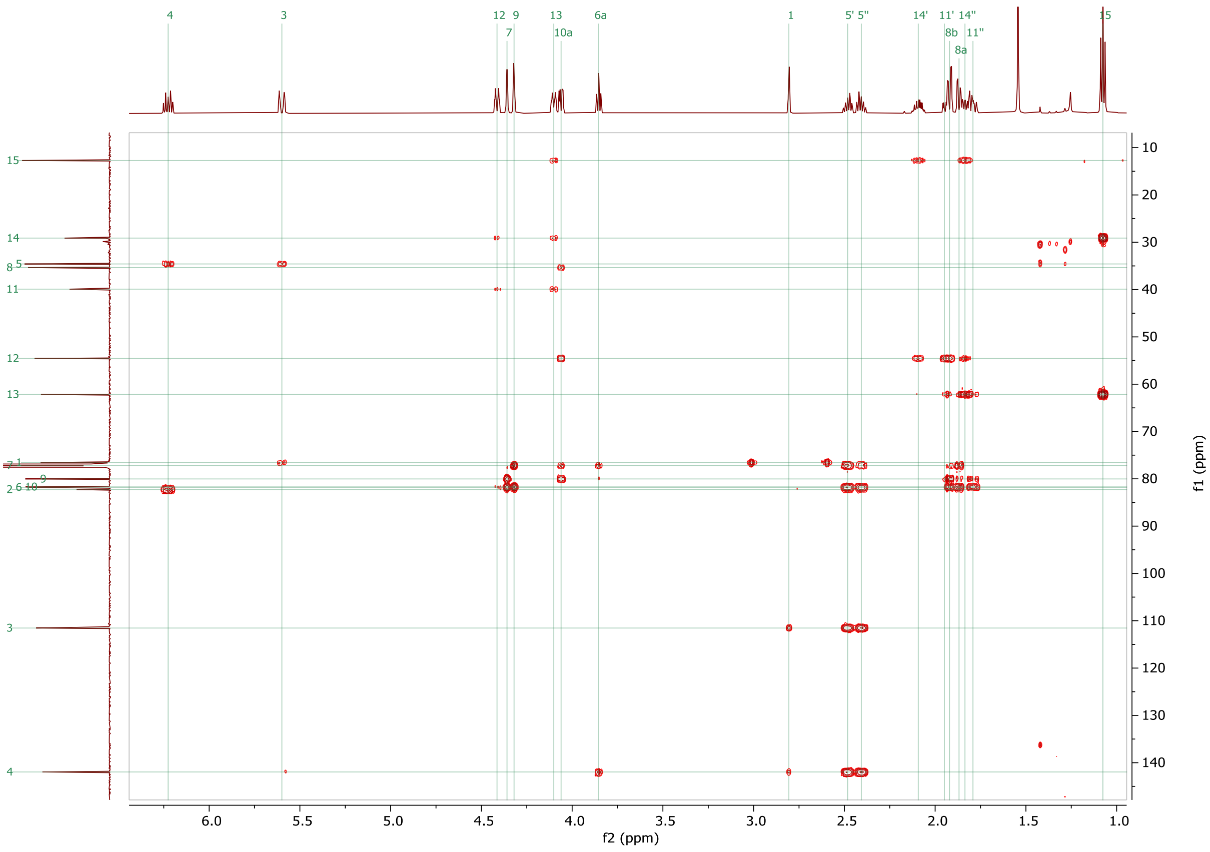

^{1}H-^{1}H NOESY of *ent*-(*E*)-5

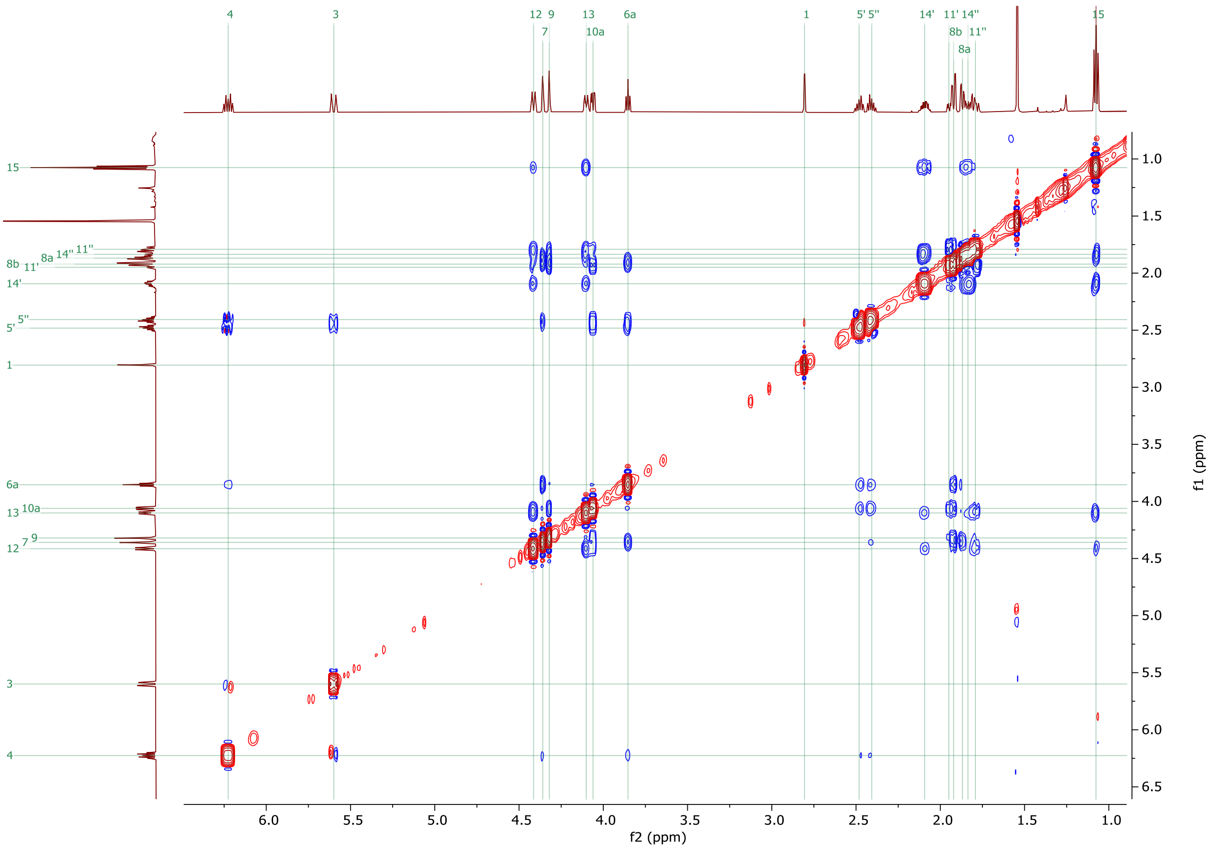

(1*S*,3*R*,4*S*,6*S*)-3-((2*S*,3*S*)-2,3-Dibromopentyl)-6-((*E*)-pent-2-en-4-yn-1-yl)-2,5-dioxabicyclo[2.2.1]heptane, (*E*)-ocellenyne ((*E*)-8)

^{1}H NMR (600 MHz, $CDCl_3$)

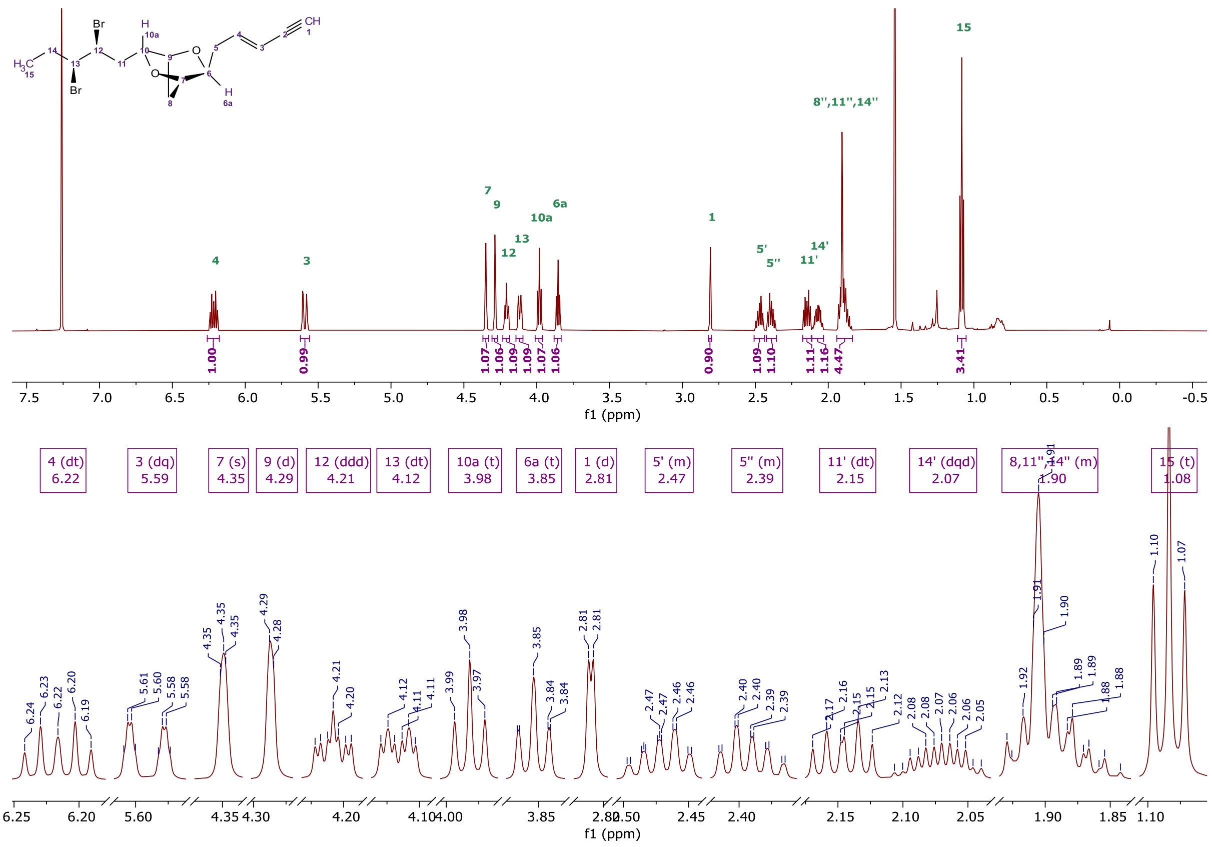

^{1}H NMR (400 MHz, $CDCl_3$) of (*E*)-ocellenyne (*E*)-8

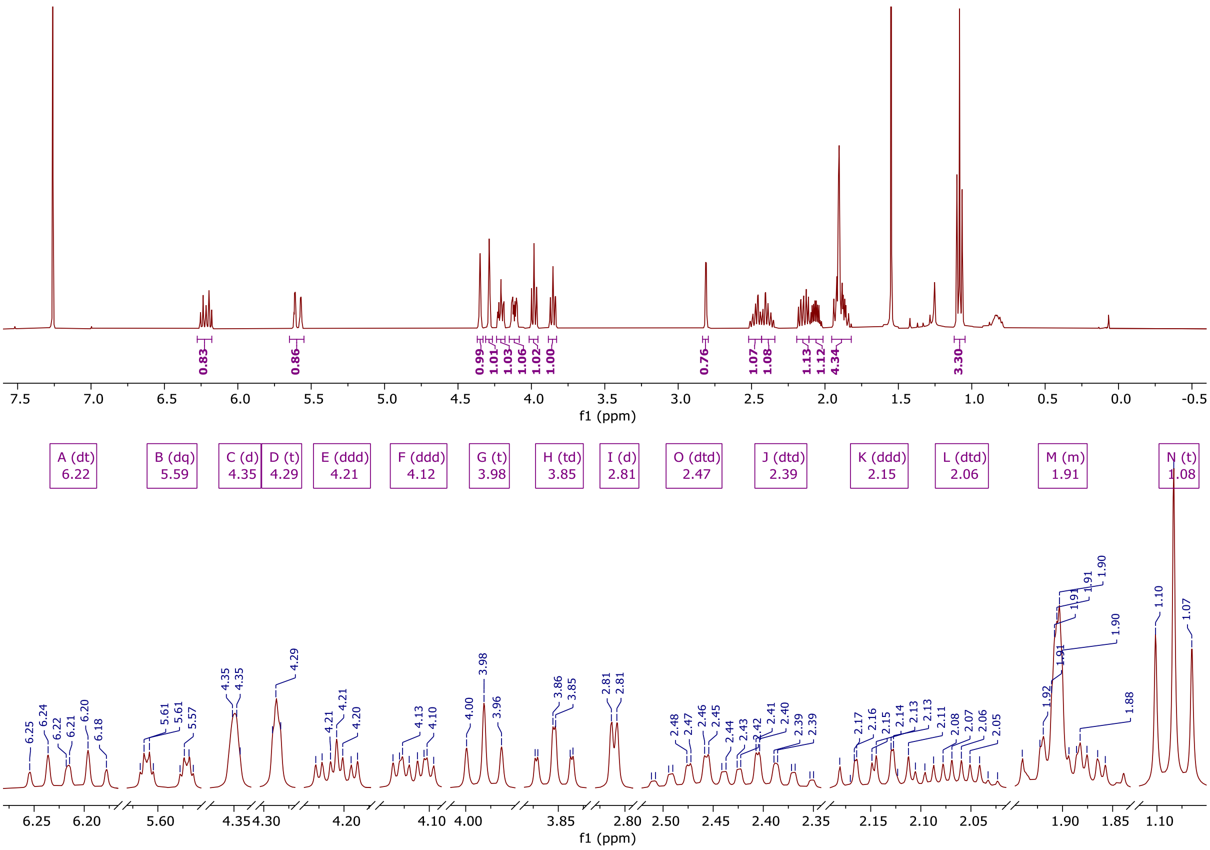

^{13}C NMR (151 MHz, $CDCl_3$)

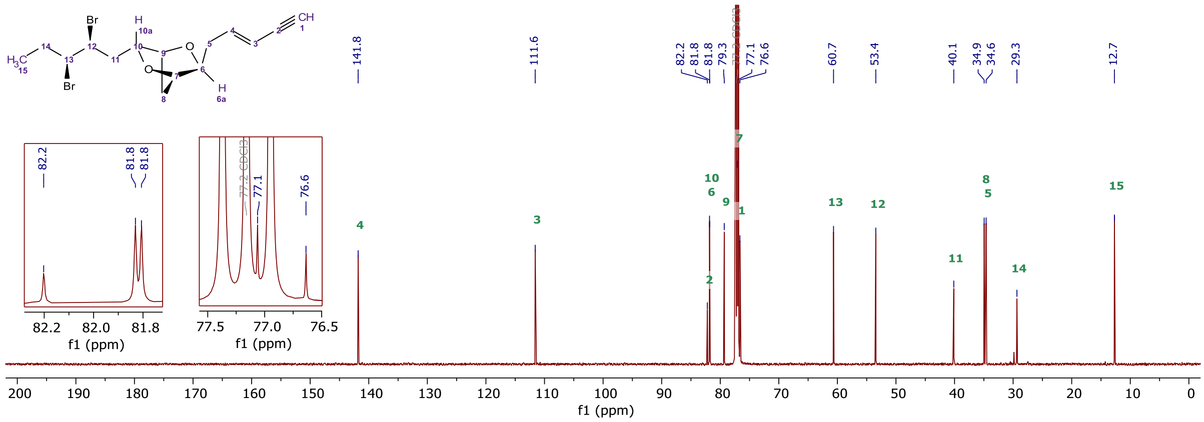

^1H-^1H COSY (*E*)-ocellenyne (*E*)-8

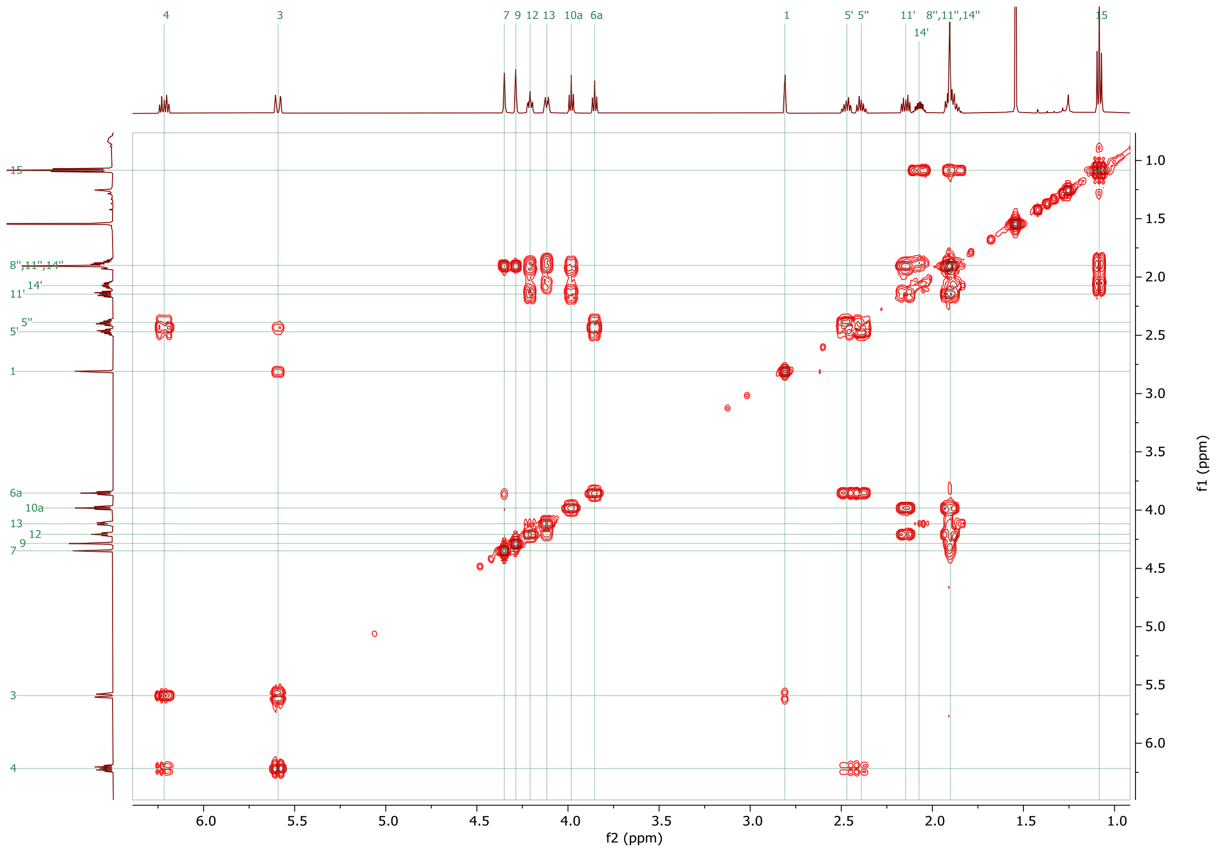

HSQC (*E*)-ocellenyne (*E*)-8

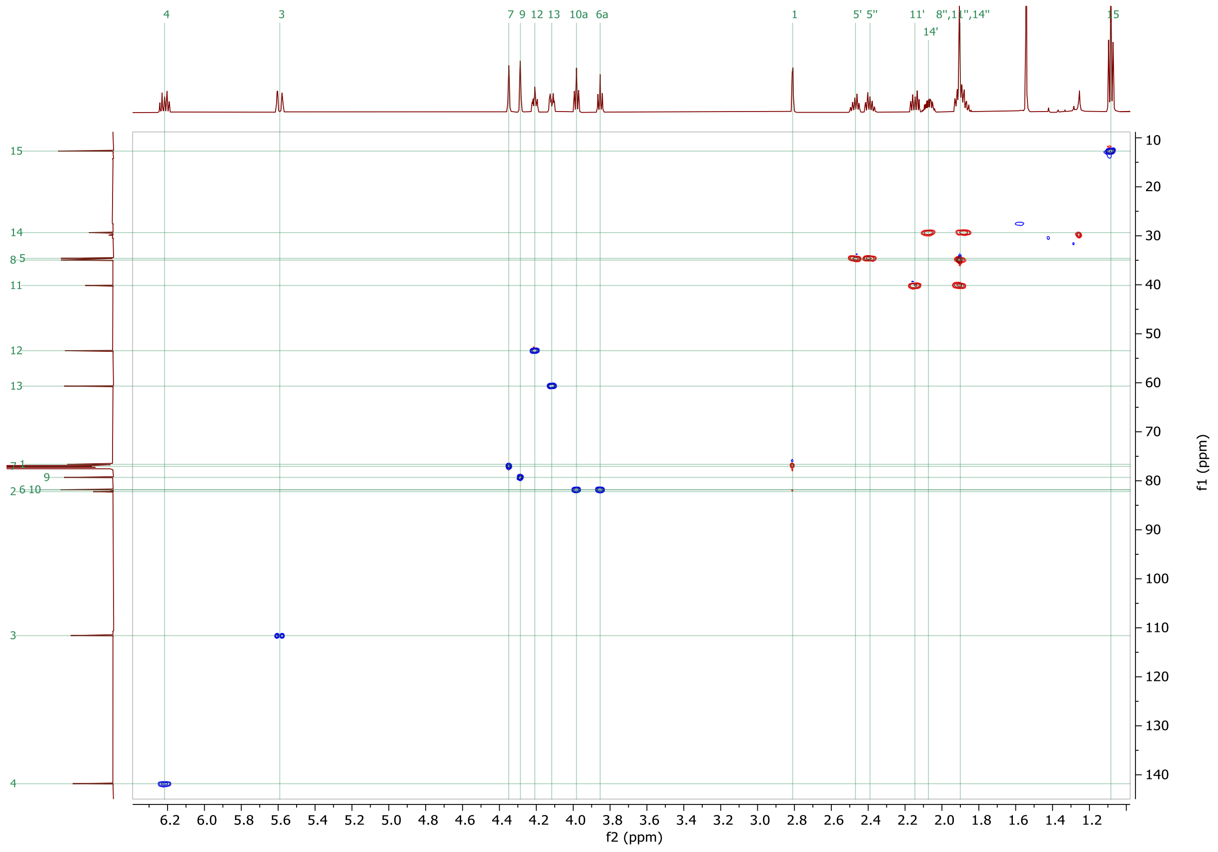

HMBC of (*E*)-ocellenyne (*E*)-8

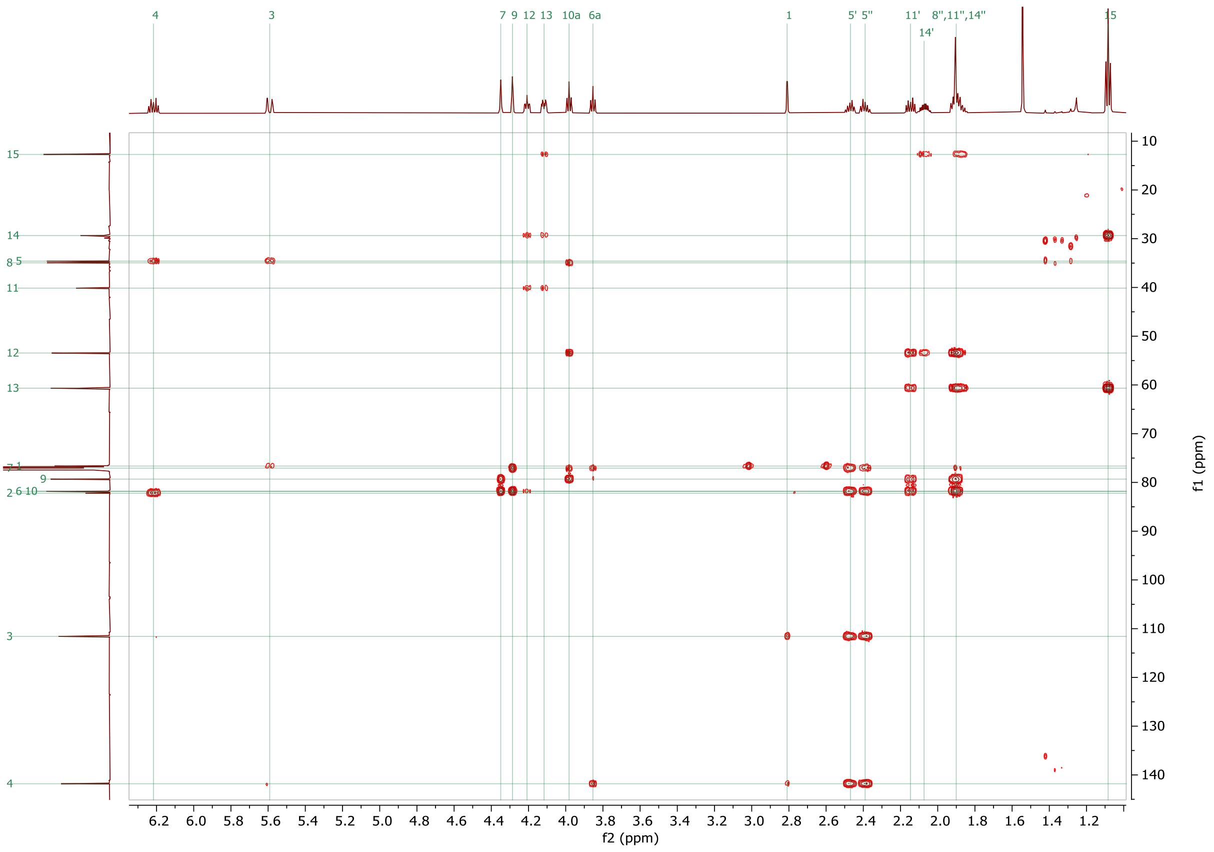

^{1}H-^{1}H NOESY of (*E*)-ocellenyne (*E*)-8

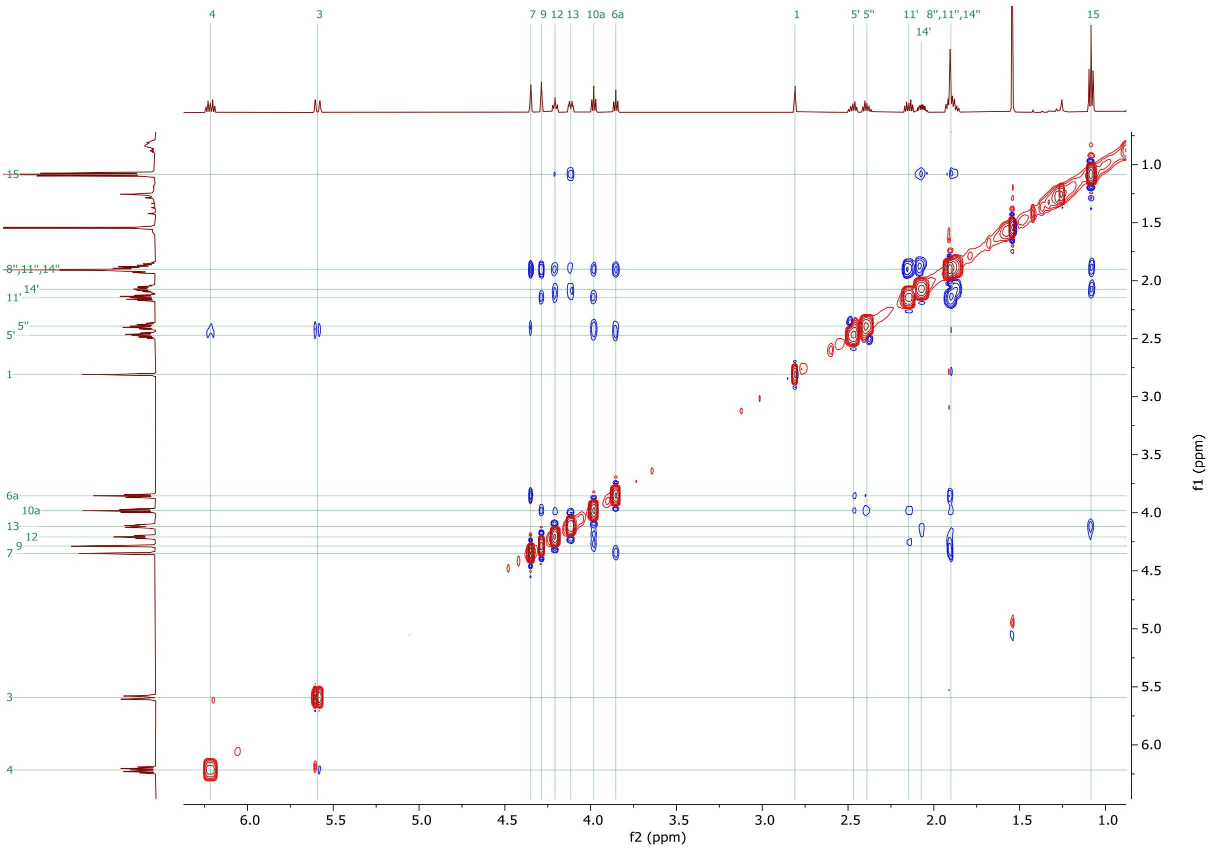

^{1}H NMR (600 MHz, C_6D_6) of (*E*)-ocellenyne (*E*)-8

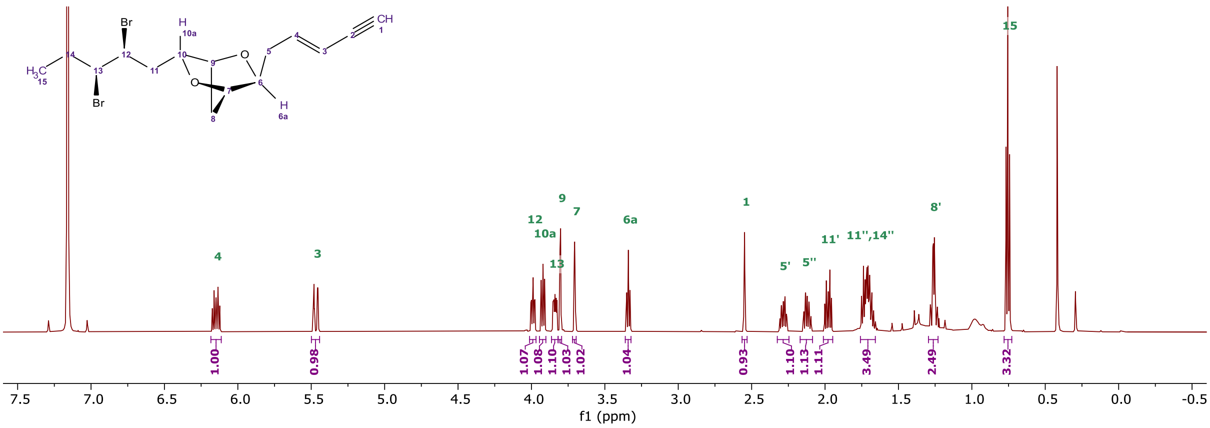

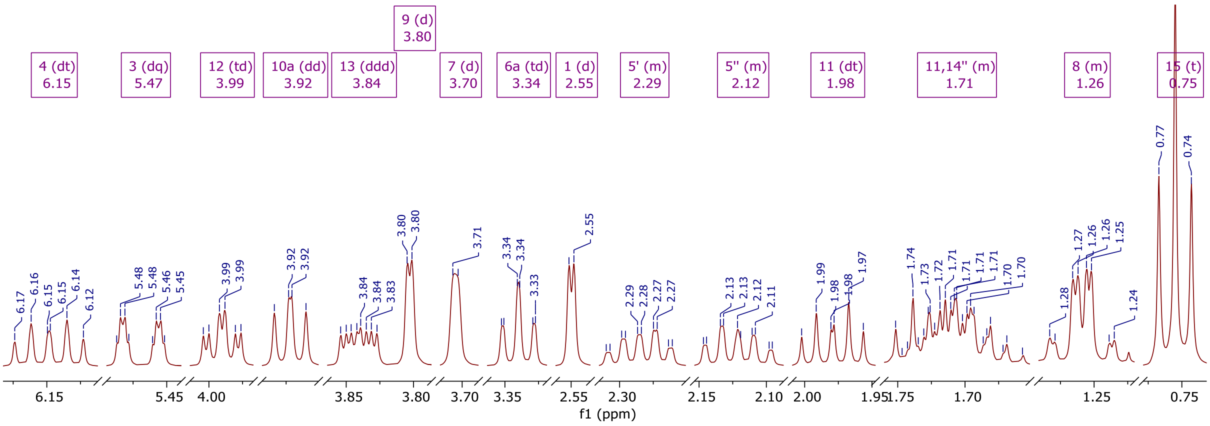

^{1}H NMR (400 MHz, C_6D_6) of (*E*)-ocellenyne (*E*)-8

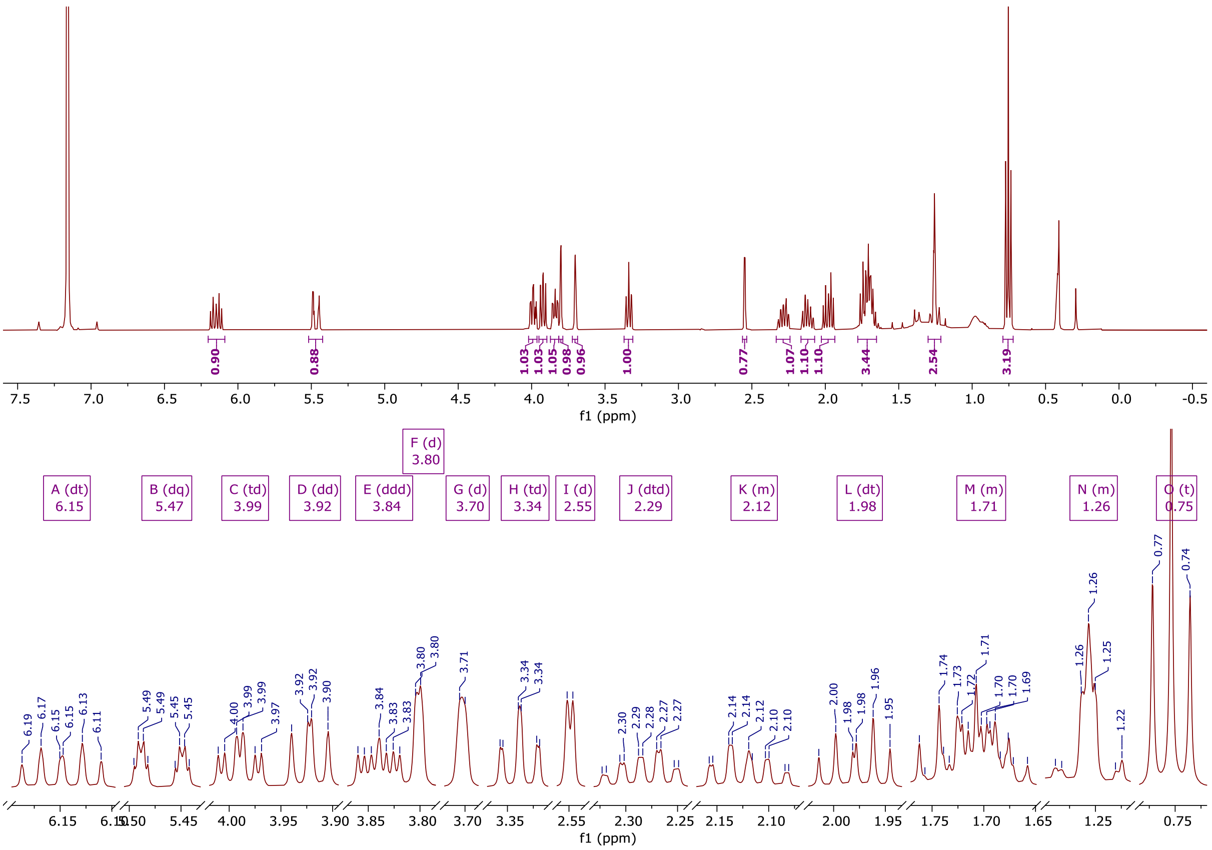

^{13}C NMR (151 MHz, C_6D_6)

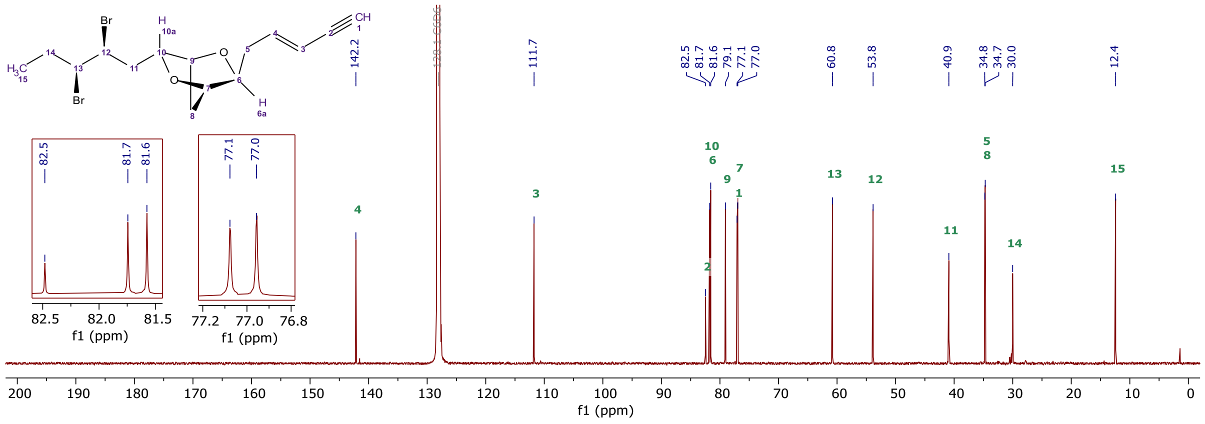

(1*S*,3*R*,4*S*,6*S*)-3-((2*R*,3*R*)-2,3-Dibromopentyl)-6-((*Z*)-3-iodoallyl)-2,5-dioxabicyclo[2.2.1]heptane (19)

^{1}H NMR (600 MHz, $CDCl_3$)

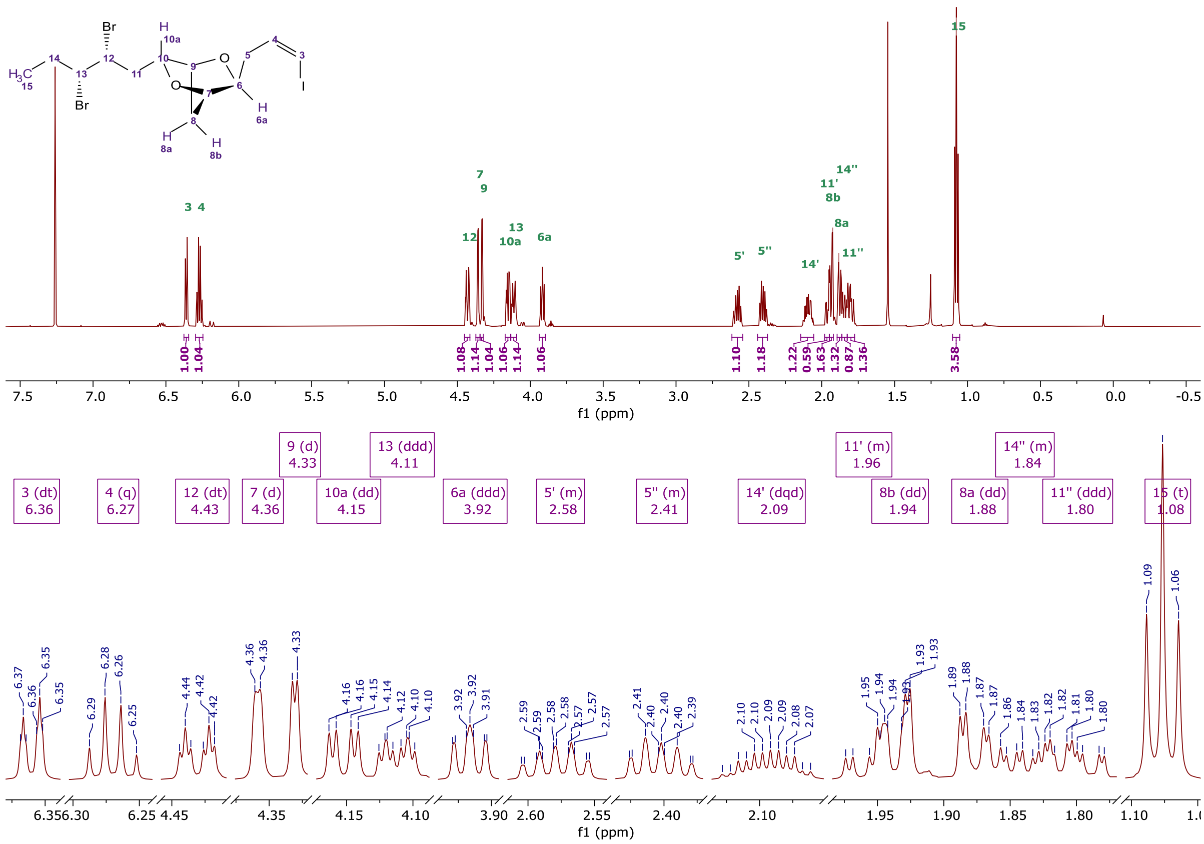

^{13}C NMR (151 MHz, $CDCl_3$)

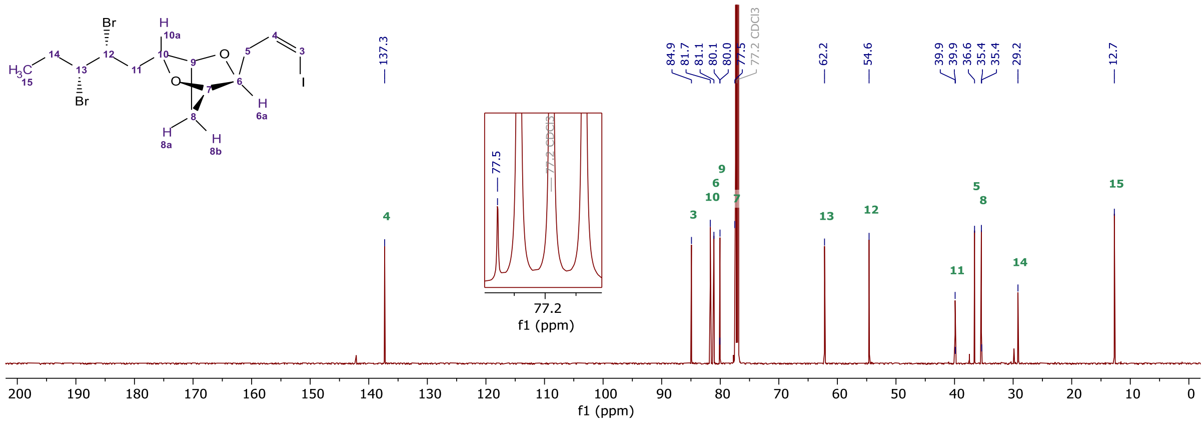

^{1}H-1H COSY of 19

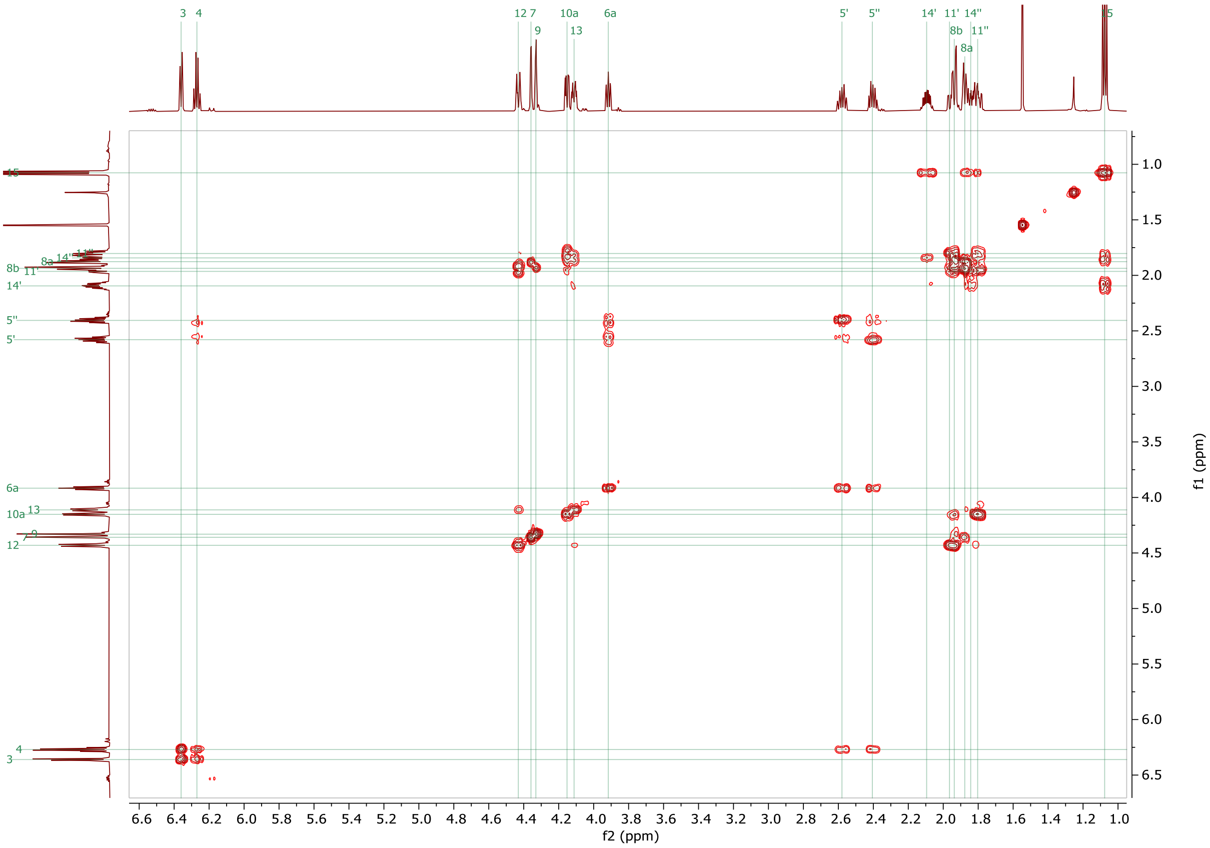

HSQC of 19

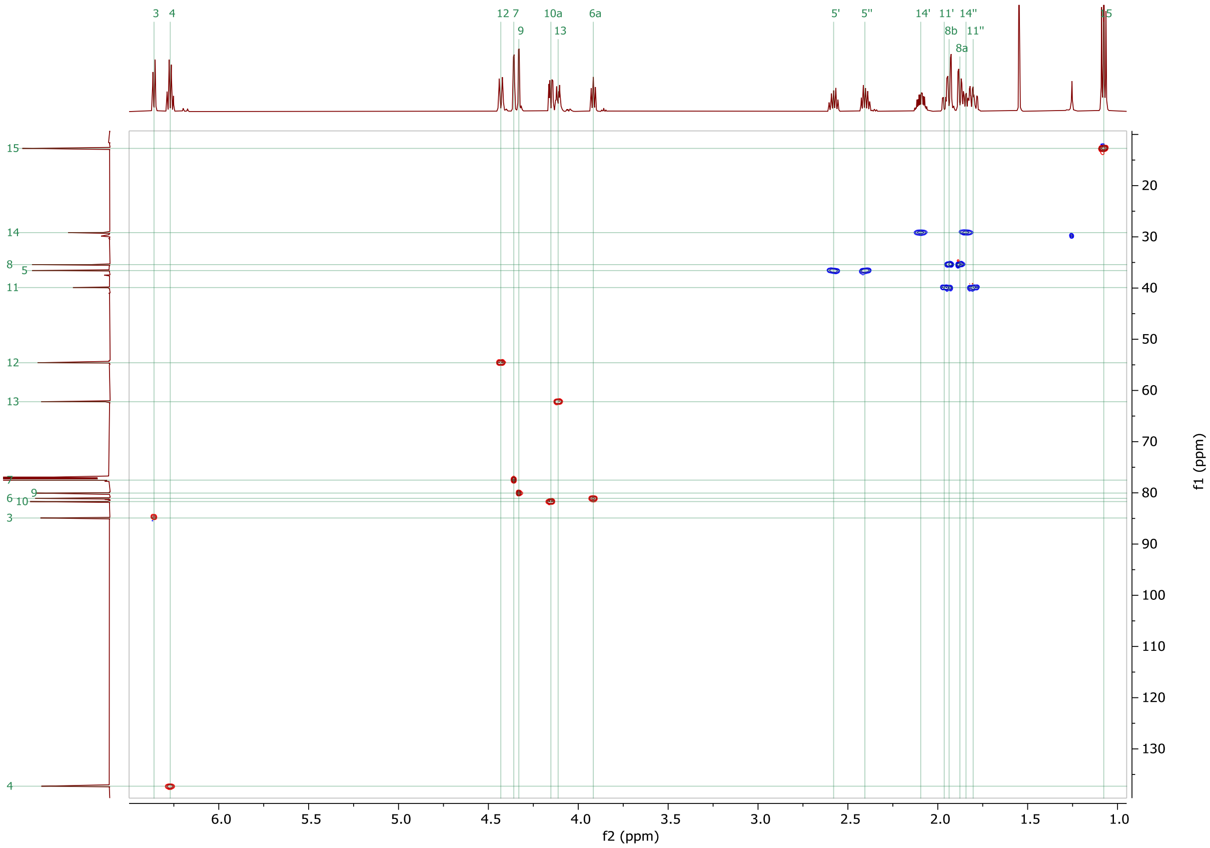

HMBC of 19

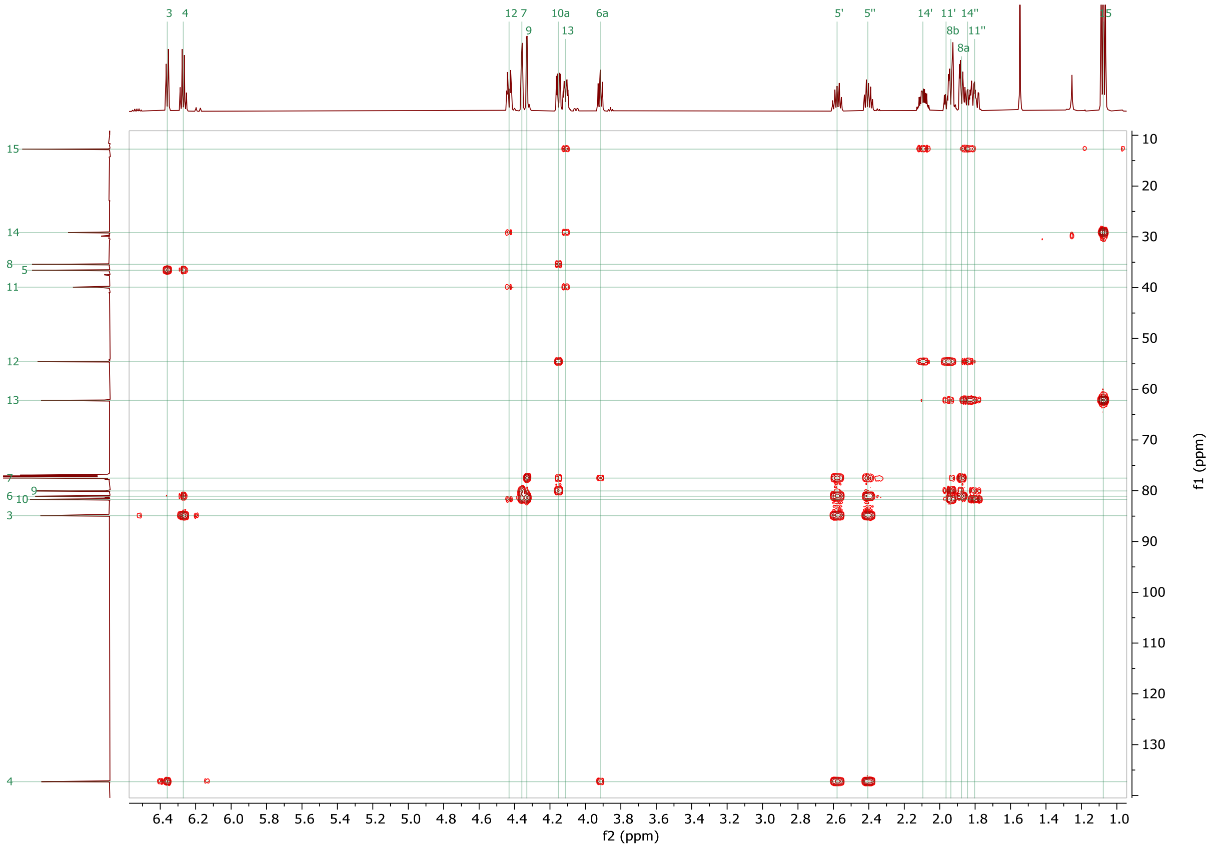

^{1}H-^{1}H NOESY of 19

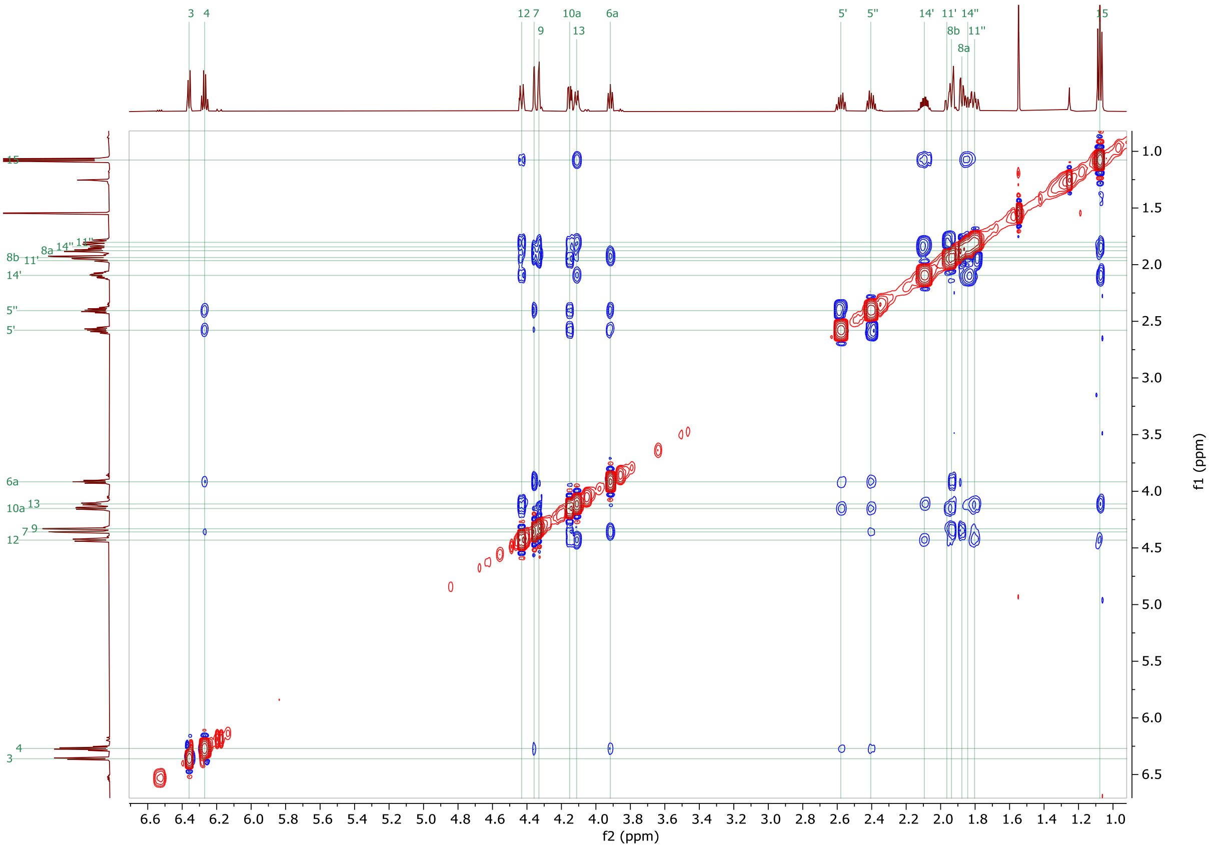

(1*S*,3*R*,4*S*,6*S*)-3-((2*S*,3*S*)-2,3-Dibromopentyl)-6-((*Z*)-3-iodoallyl)-2,5-dioxabicyclo[2.2.1]heptane (20)

^{1}H NMR (600 MHz, $CDCl_3$)

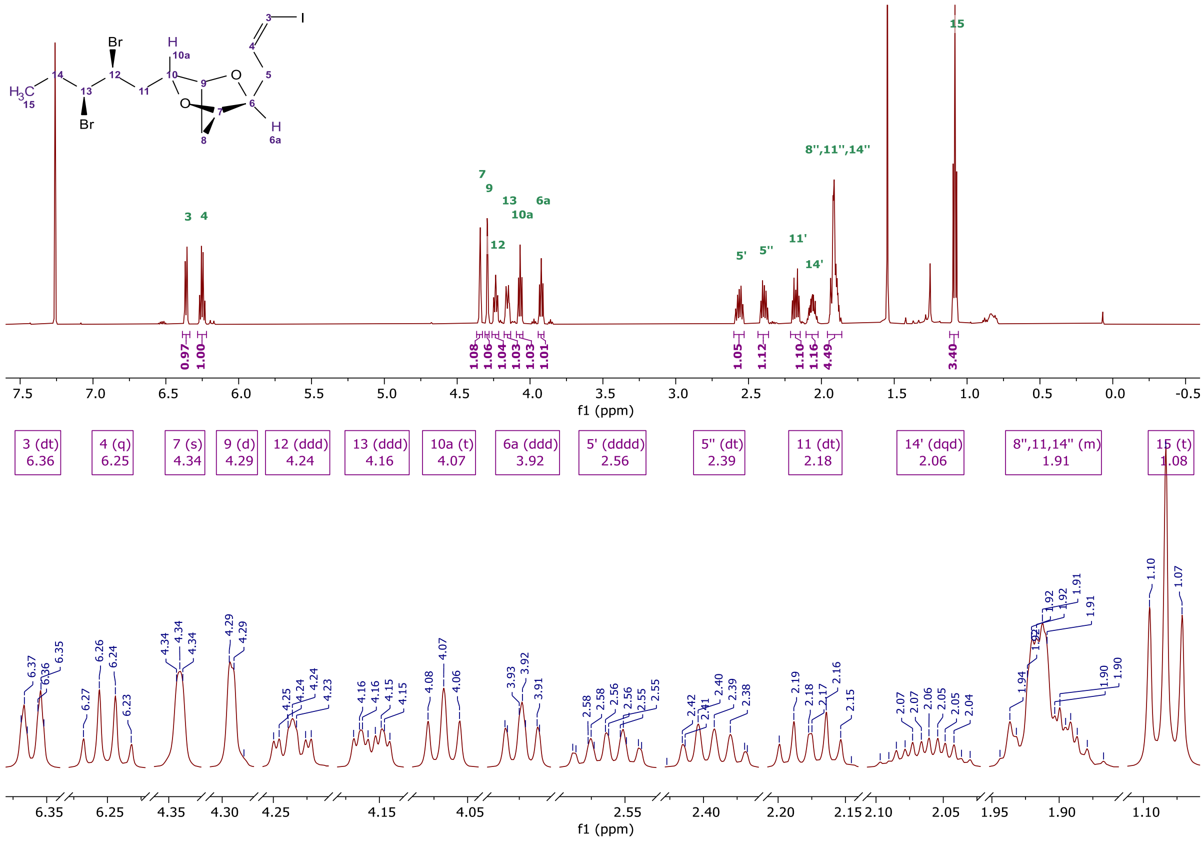

^{13}C NMR (151 MHz, $CDCl_3$)

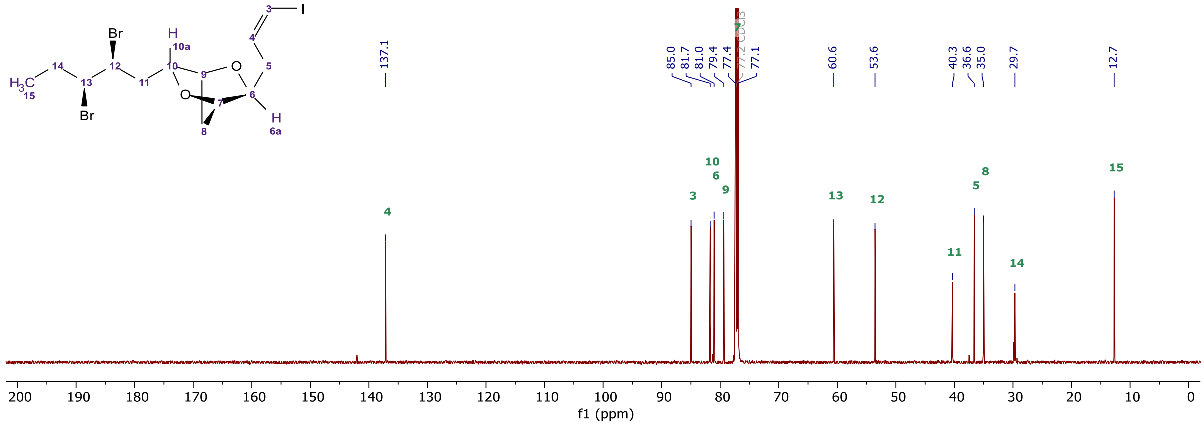

^{1}H-^{1}H COSY of 20

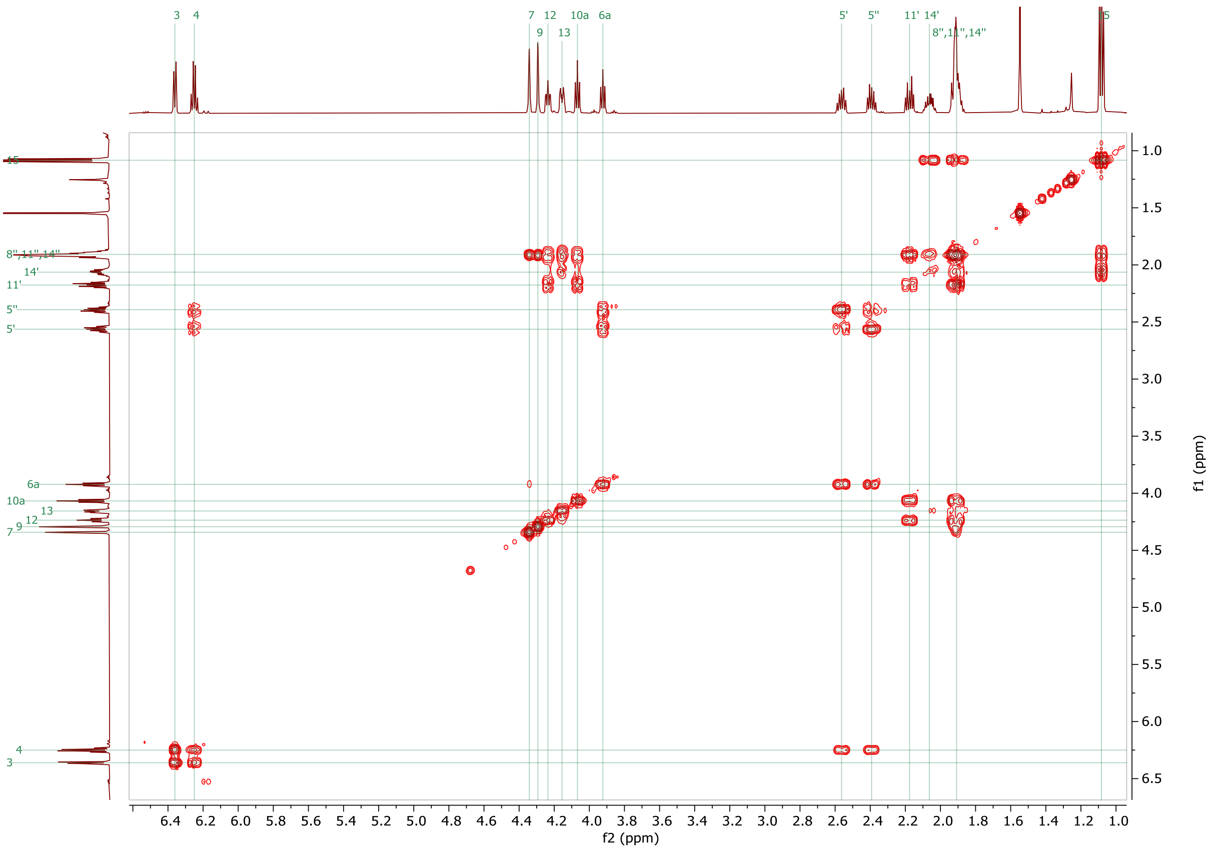

HSQC of 20

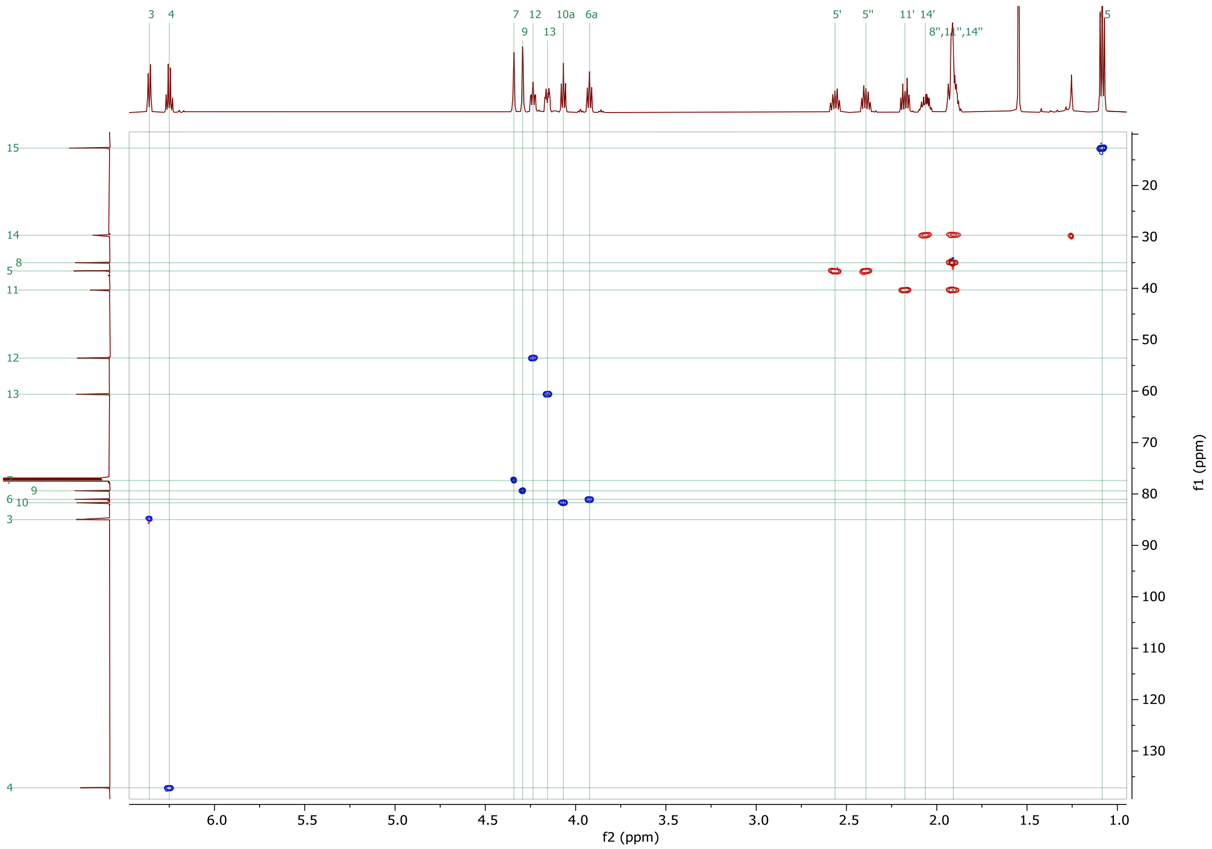

HMBC of 20

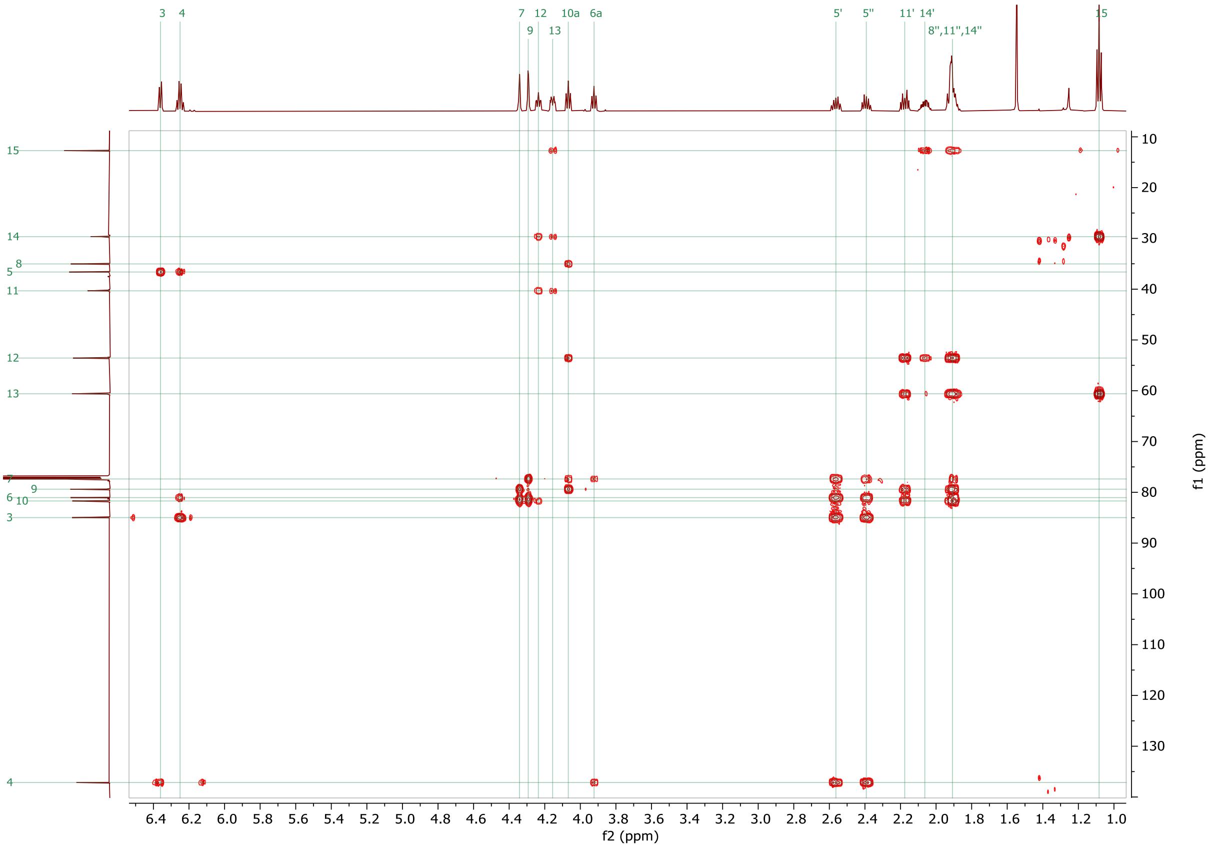

^{1}H-^{1}H NOESY of 20

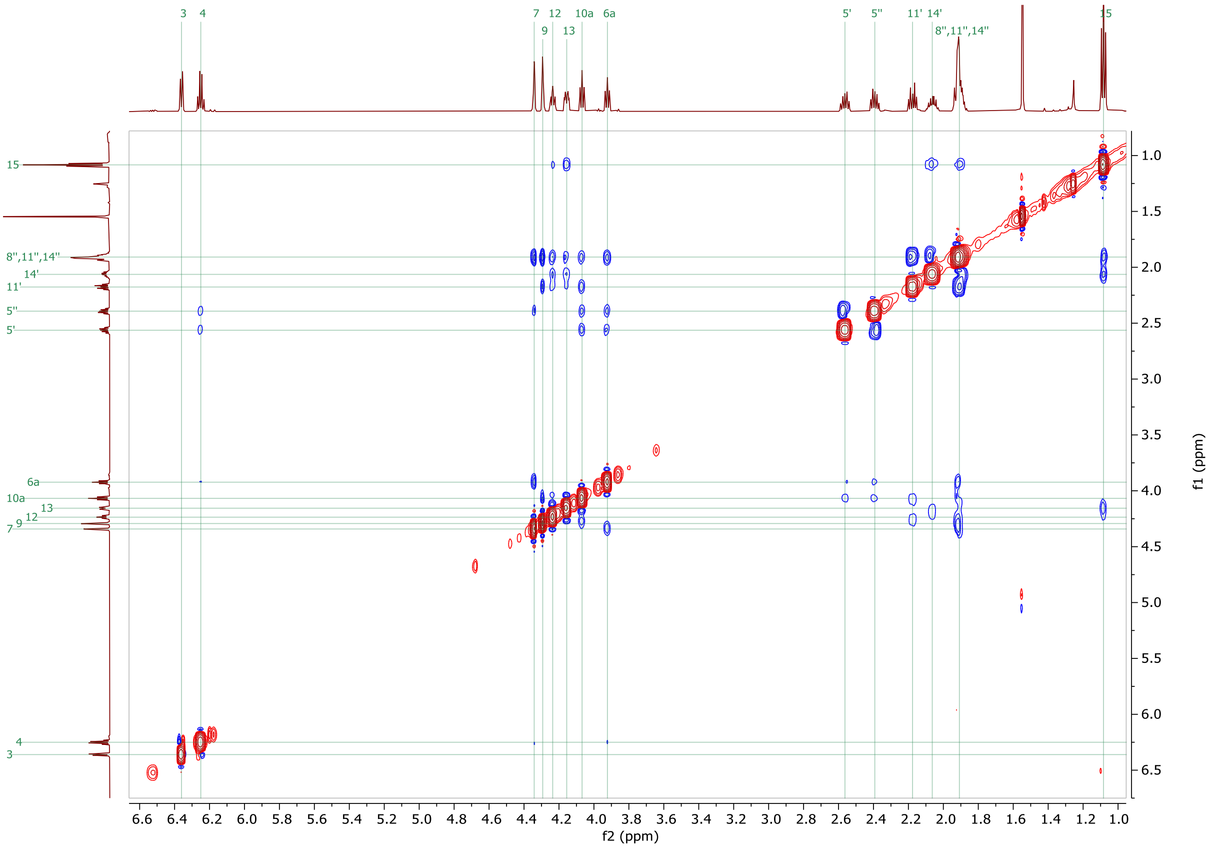

(1S,3R,4S,6S)-3-((2R,3R)-2,3-Dibromopentyl)-6-((Z)-pent-2-en-4-yn-1-yl)-2,5-dioxabicyclo[2.2.1]heptane (ent-(Z)-5)

^{1}H NMR (600 MHz, $CDCl_3$)

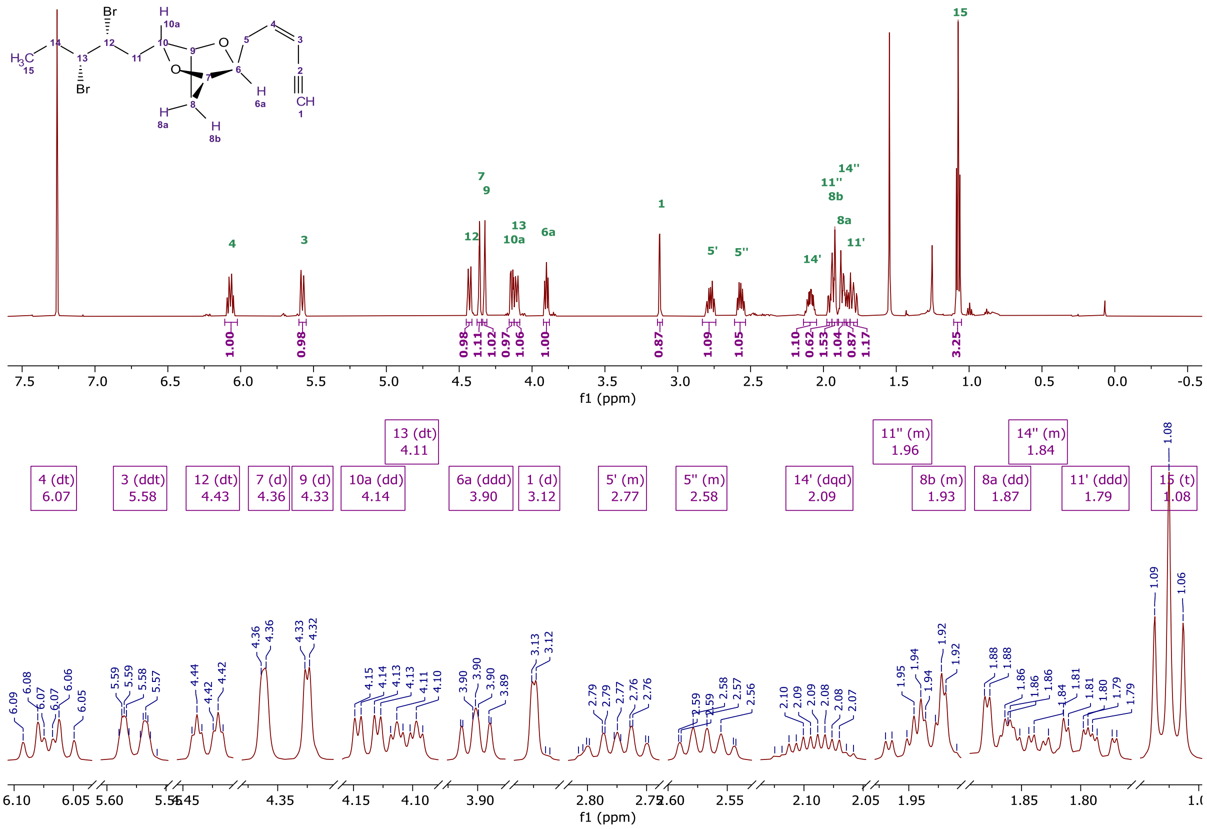

^{1}H NMR (400 MHz, $CDCl_3$) of *ent*-(*Z*)-5

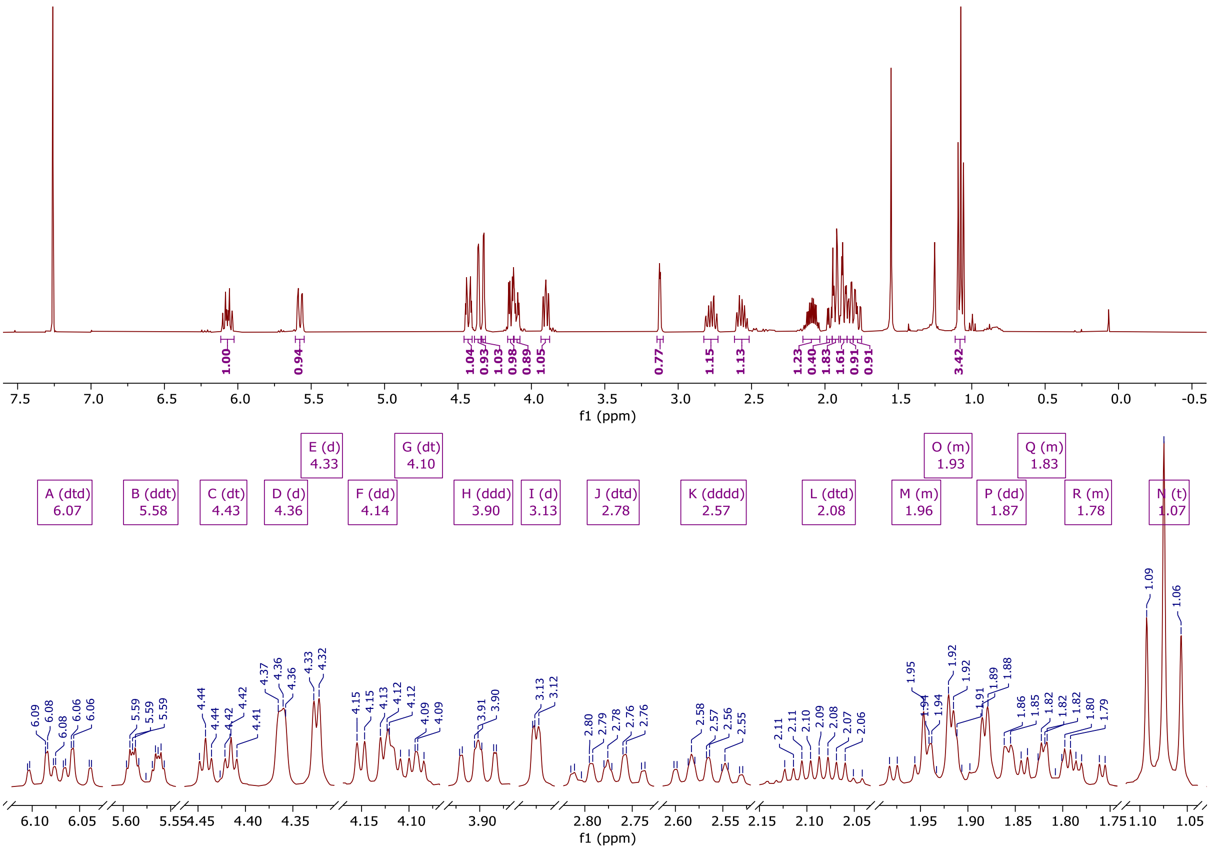

^{13}C NMR (151 MHz, $CDCl_3$)

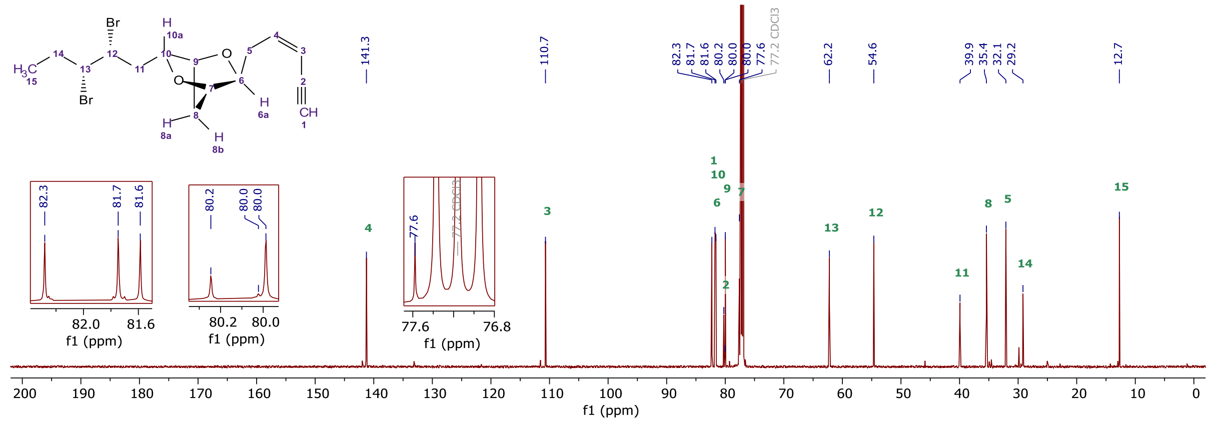

1H-1H COSY of *ent*-(*Z*)-5

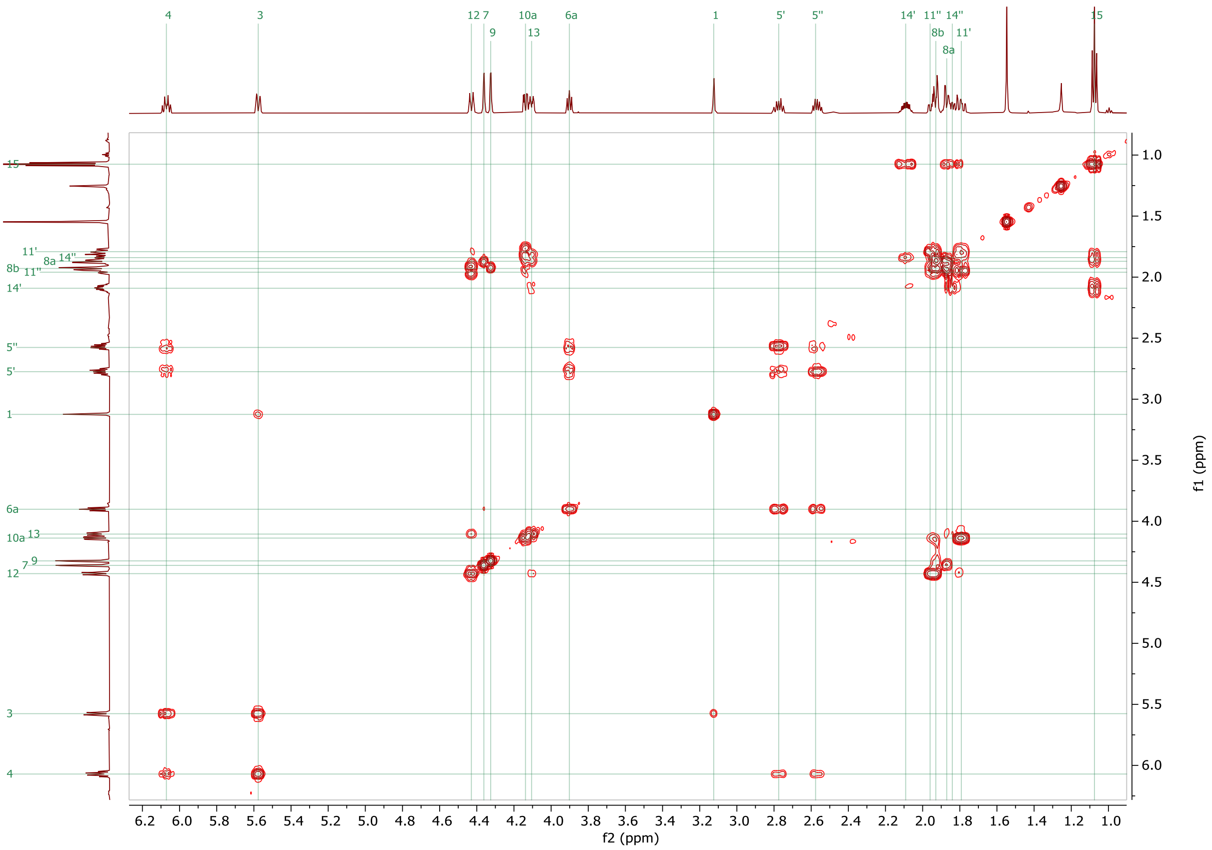

HSQC of *ent*-(*Z*)-5

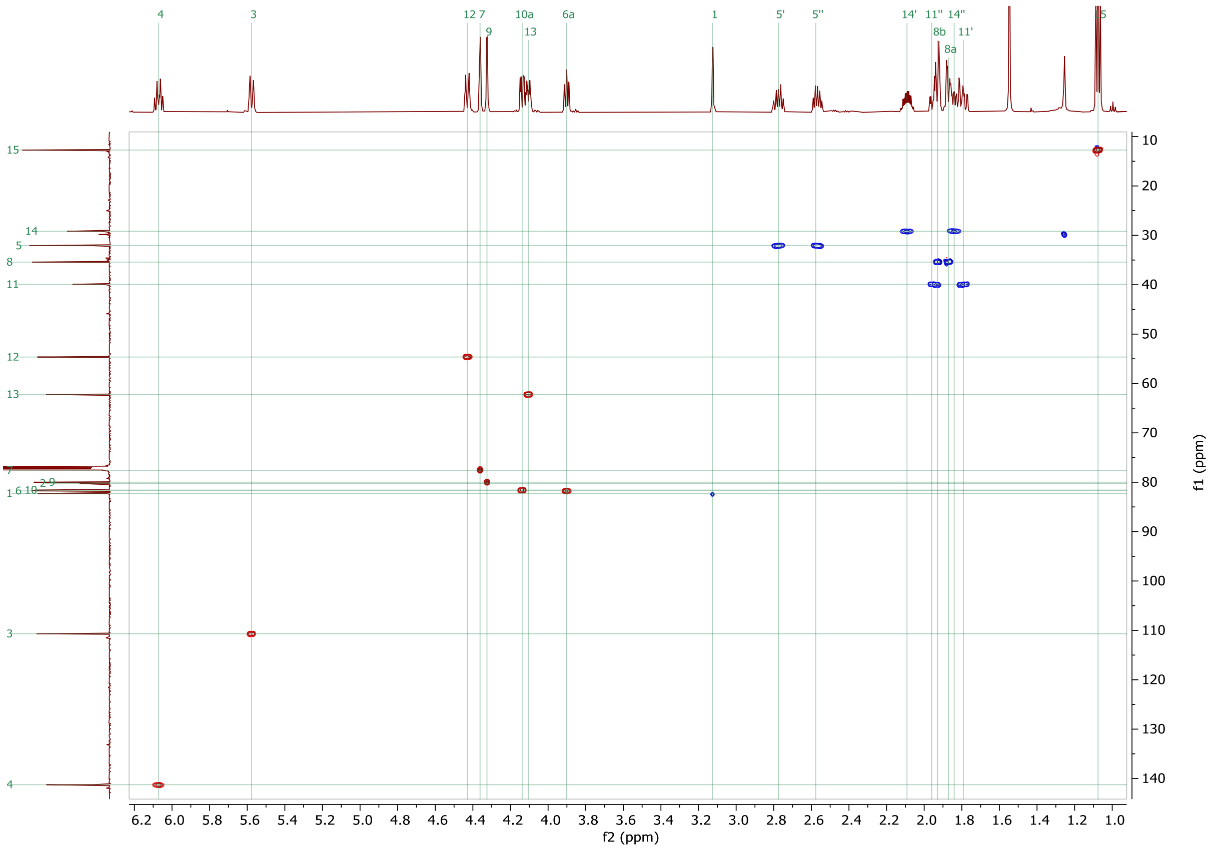

HMBC of *ent*-(*Z*)-5

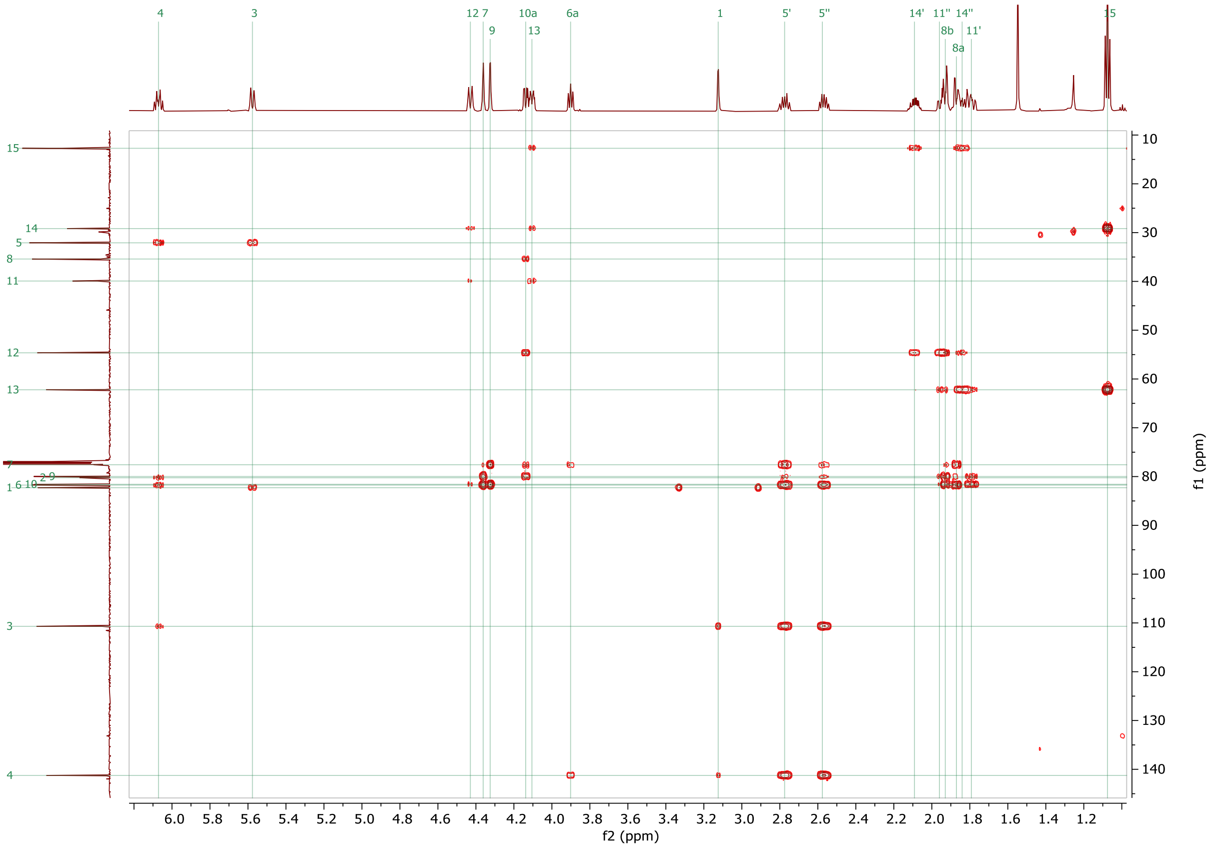

^{1}H-^{1}H NOESY of *ent*-(*Z*)-5

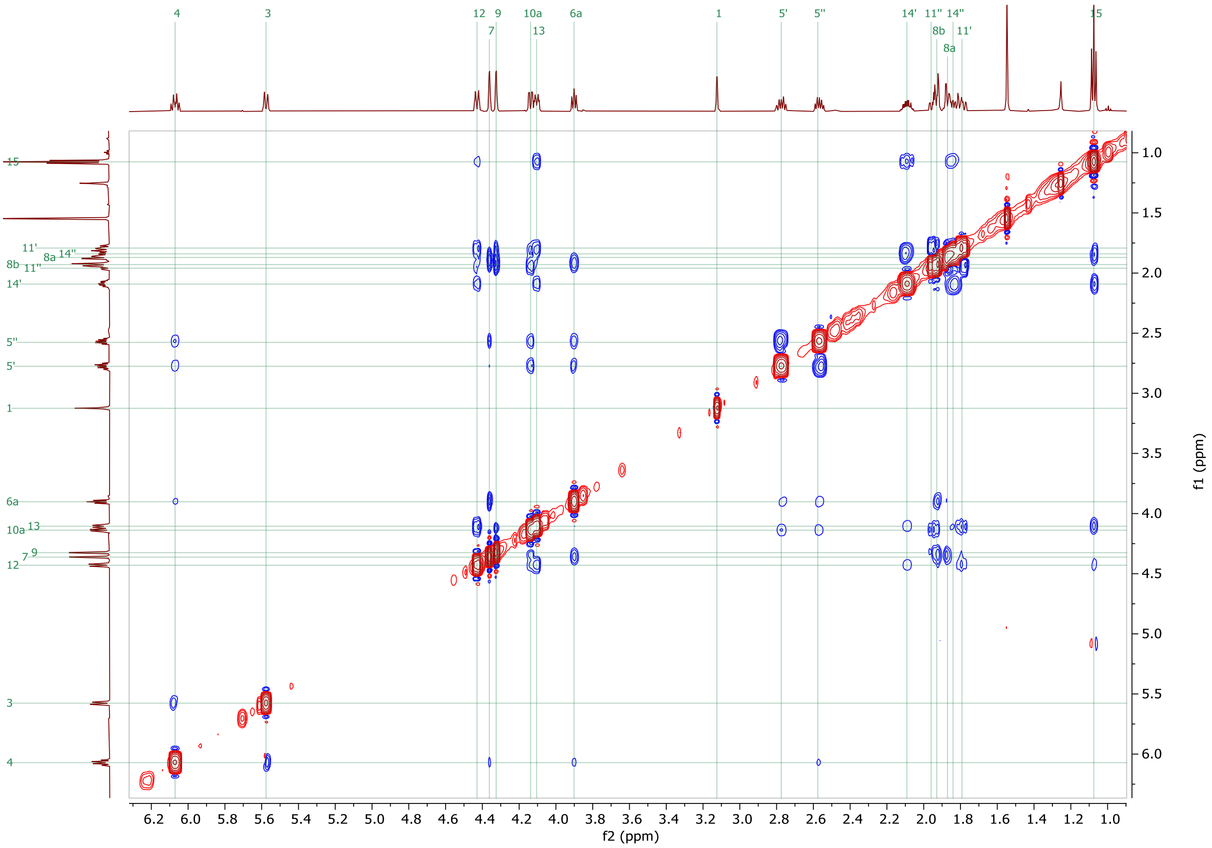

(1*S*,3*R*,4*S*,6*S*)-3-((2*S*,3*S*)-2,3-Dibromopentyl)-6-((*Z*)-pent-2-en-4-yn-1-yl)-2,5-dioxabicyclo[2.2.1]heptane, (*Z*)-ocellenyne ((*Z*)-8)

^{1}H NMR (600 MHz, $CDCl_3$)

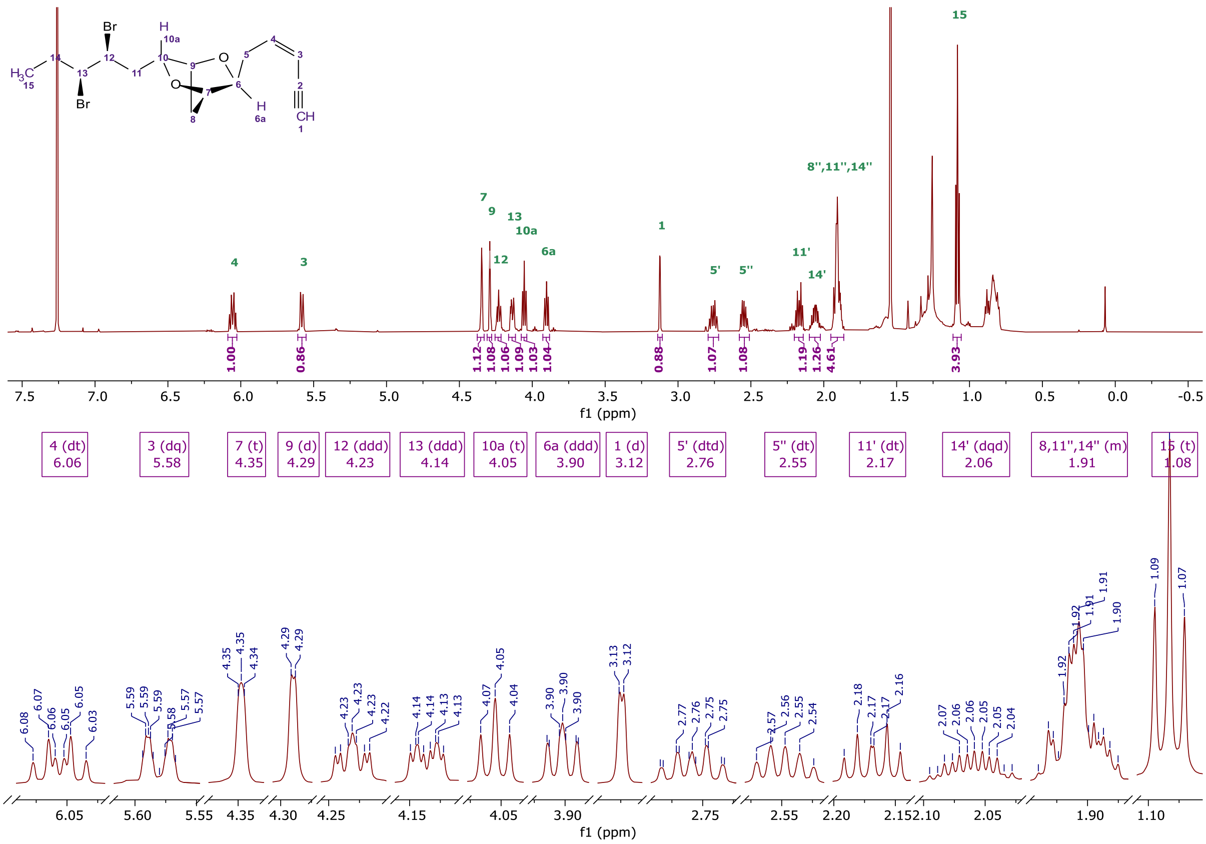

^{1}H NMR (400 MHz, $CDCl_3$) of (*Z*)-ocellenyne (*Z*)-8

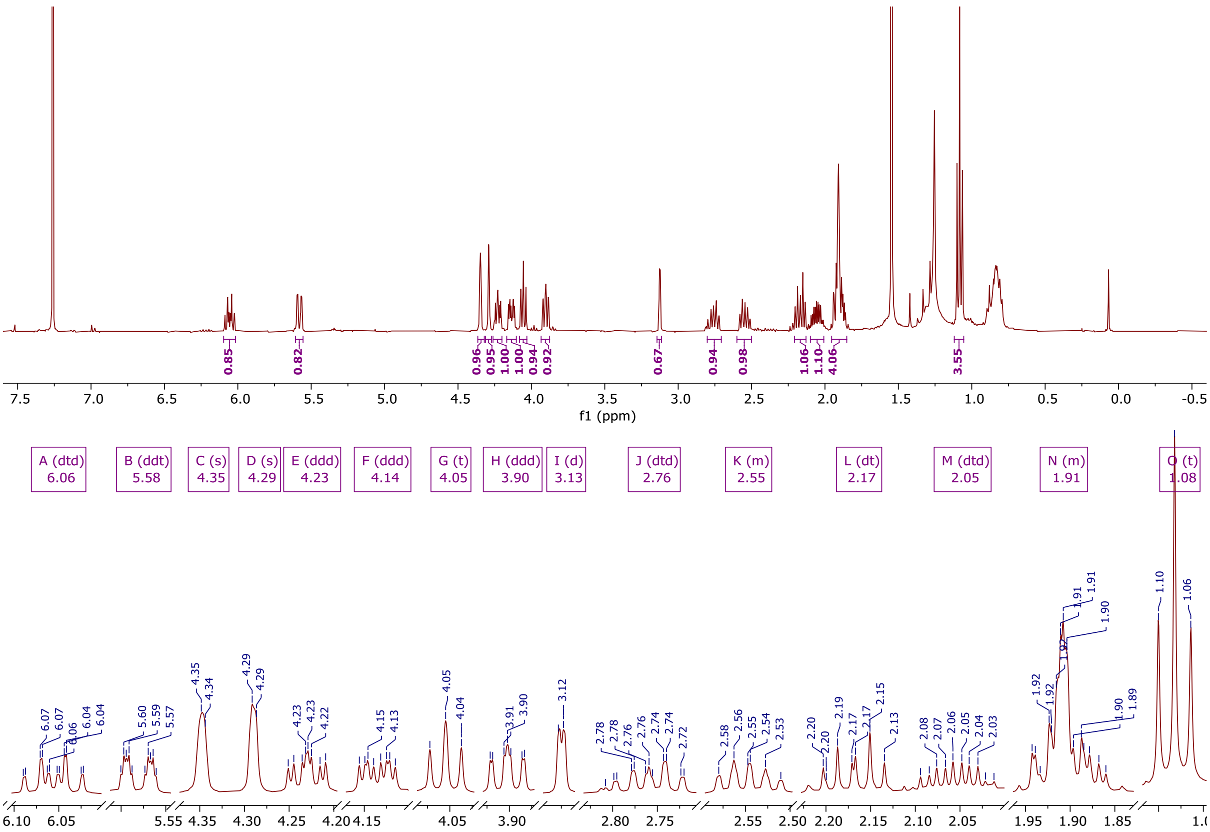

^{13}C NMR (151 MHz, $CDCl_3$)

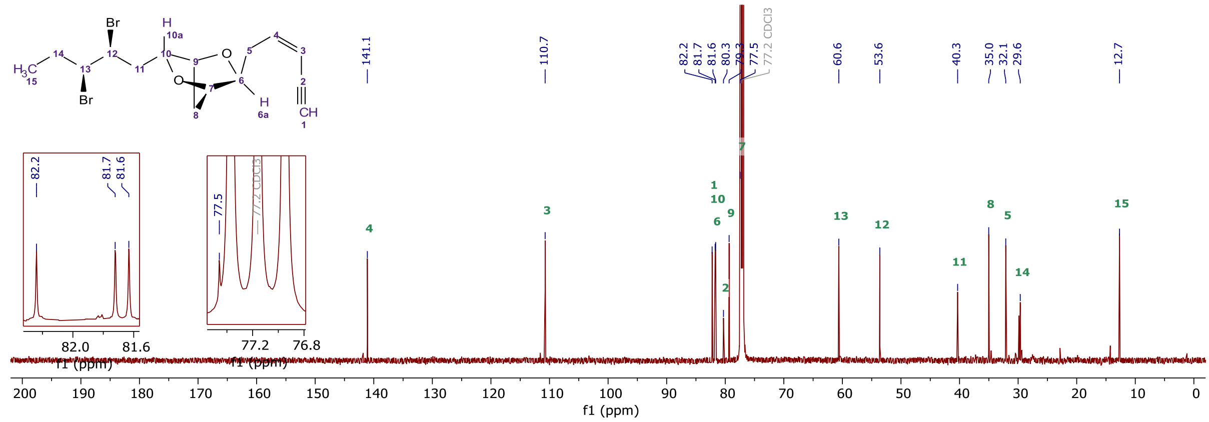

^{1}H-^{1}H COSY of (*Z*)-ocellenyne (*Z*)-8

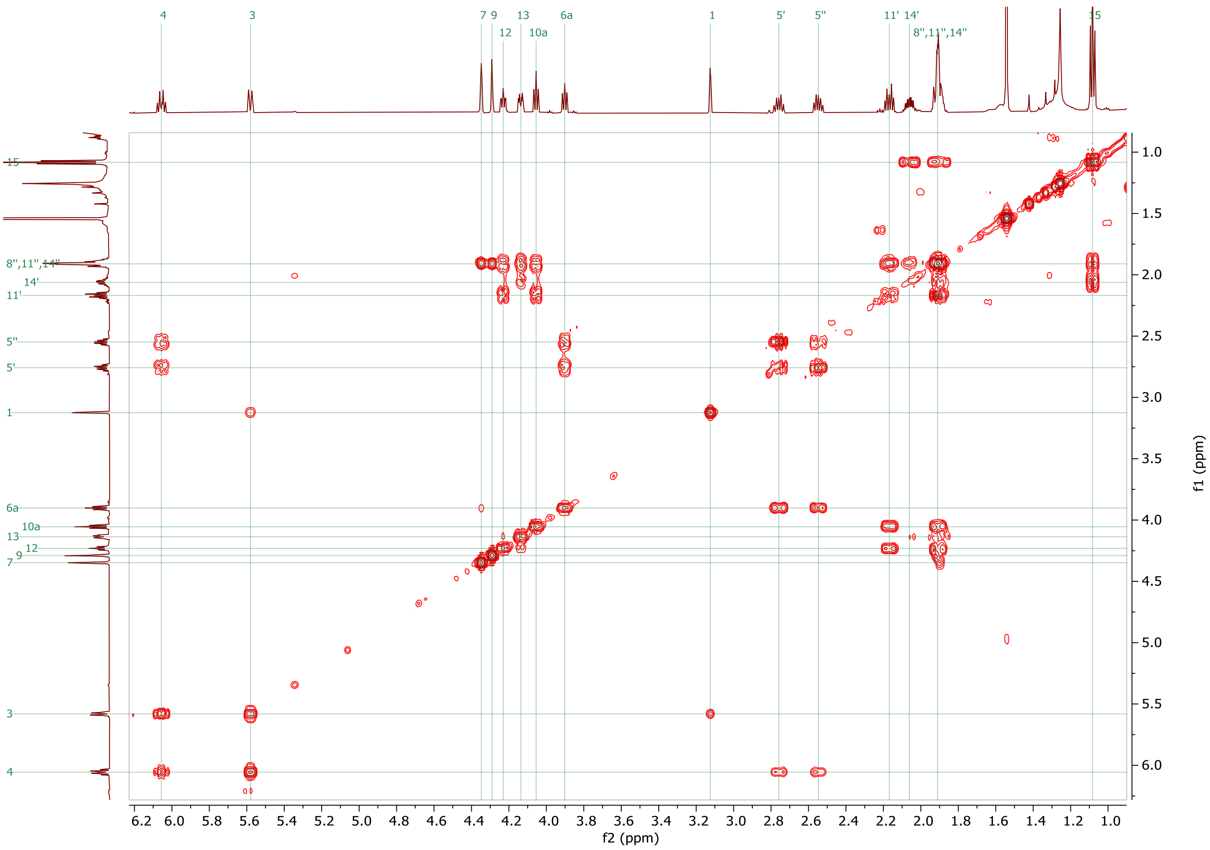

HSQC of (*Z*)-ocellenyne (*Z*)-8

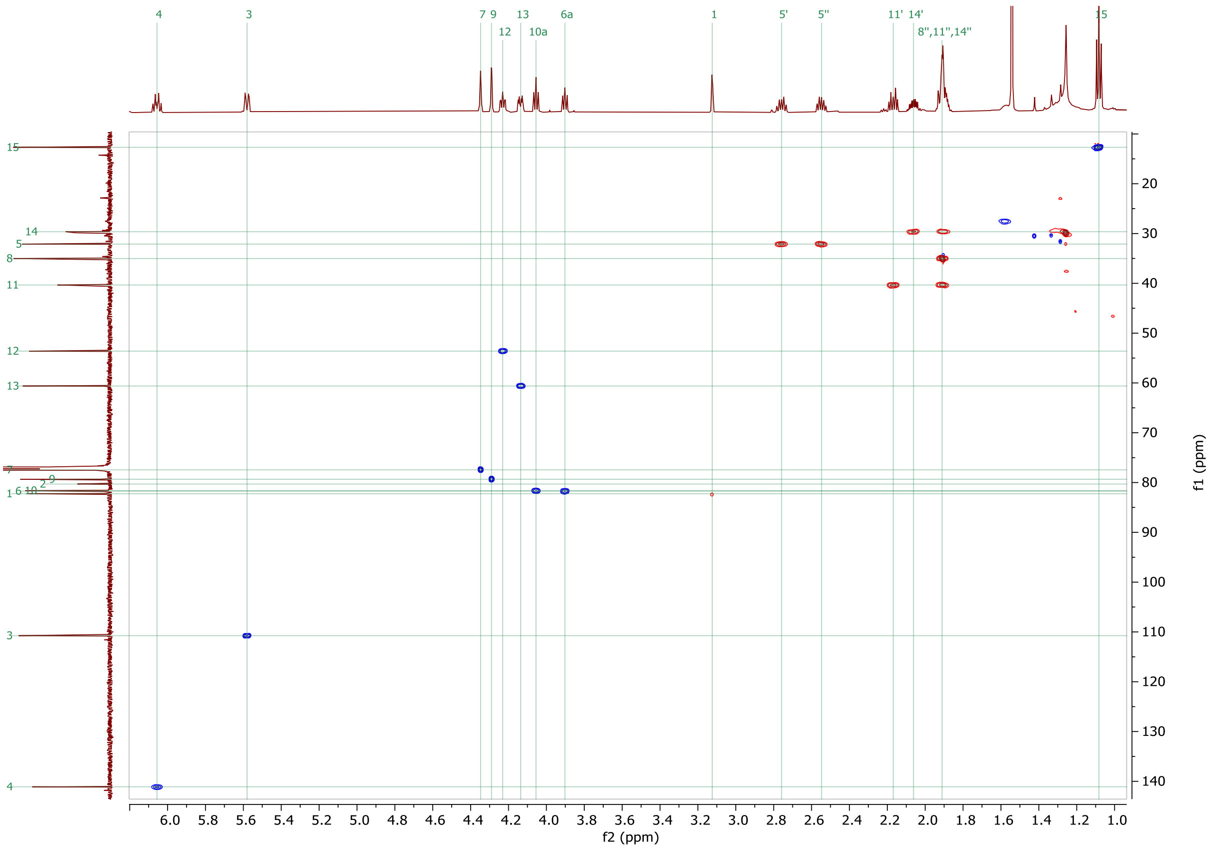

HMBC of (Z)-ocellenyne (Z)-8

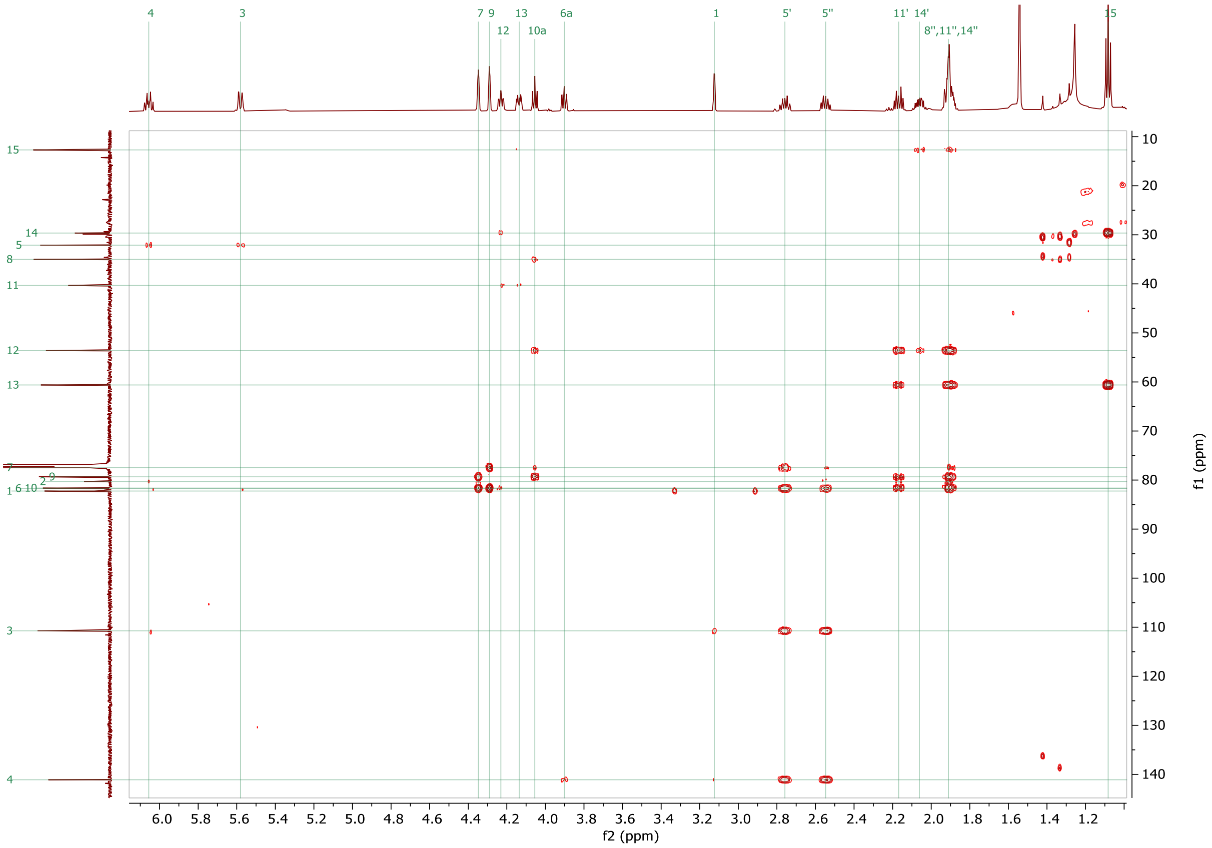

^{1}H-^{1}H NOESY of (Z)-ocellenyne (Z)-8

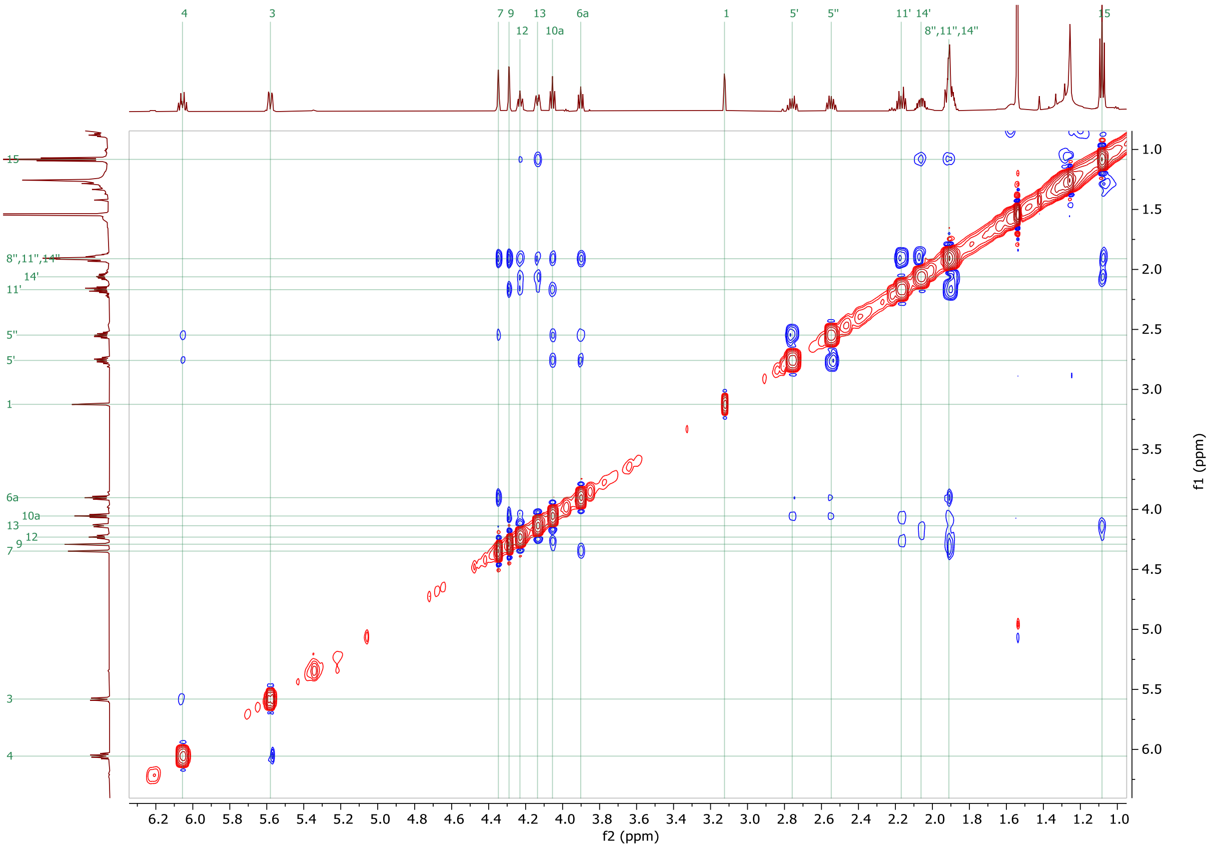

Supplement: Supplementary file 1 — ol2c03524_si_001.pdf [file ol2c03524_si_001.pdf]
